# The design, performance and organizational impact of a point-of-care ultrasound (POCUS) elective for internal medicine residents

**DOI:** 10.1186/s12909-025-06802-x

**Published:** 2025-02-18

**Authors:** Harry Kuperstein, Werda Alam, Azzam Paroya, Kinner Patel, Sahar Ahmad

**Affiliations:** 1 Renaissance School of Medicine, Stony Brook, NY USA; 2 Department of Anesthesiology, Mount Sinai West, New York, NY USA; 3 Division of Pulmonary, Critical Care and Sleep Medicine, Department of Medicine, Renaissance School of Medicine at Stony Brook University, Stony Brook, NY USA; 4 Division of Pulmonary, Allergy and Critical Care Medicine, Heersink School of Medicine, Birmingham, AL England; 5 Division of Pulmonary, Critical Care and Sleep Medicine, Department of Medicine, Stony Brook University Hospital, HSC 17, Stony Brook, NY 11794 USA

**Keywords:** Ultrasound education, Point-of-care ultrasound, Bedside ultrasound, Ultrasonography, Echocardiography, Internal medicine, Residency, Elective, Competency, Barriers to ultrasound education

## Abstract

**Background:**

An educational gap for point-of-care-ultrasound (POCUS) training exists within Internal Medicine (IM) residency programs in that there is currently no standardized training paradigm. To address this need, we designed and implemented a five-day (one work week) elective for POCUS training intending to target IM resident POCUS knowledge and skills. This course integrates self-directed learning and supervised hands-on practice to deliver effective resident education in POCUS.

**Methods:**

IM residents completed the five-day POCUS elective. Residents who took the elective were given an elective evaluation survey, written POCUS knowledge exams at a pre-course and post-course timepoint, as well as a post-course skills assessment exam.

**Results:**

45 IM residents completed the elective in total. 47% (*N* = 21) of all participating residents completed the evaluation survey. 94% of those who responded to the evaluation survey reported above average or outstanding satisfaction with all aspects of the elective, including hands-on teaching and materials provided. Written knowledge exams results showed a significant increase in POCUS knowledge scores, with pre-test and post-test scores increasing from 39 to 66%, respectively (*N* = 30, *p* < 0.001). Overall, on a skills evaluation of tested residents (*N* = 20), 45% were deemed to acquire images independently while 40% could interpret independently, with all learners deemed able to do both with some level of supervision. Overall use of POCUS by IM residents as measured by saved ultrasound studies increased after the implementation of the elective, suggesting institutional impact.

**Discussion:**

POCUS training is a recognized need for IM residency programs. While existing POCUS training programs vary in both length of course and depth of material to suit different educational objectives, we have presented a well-received and effective POCUS training paradigm aimed at achieving knowledge acquisition for clinical practice. This POCUS elective rotation, which is integrated into trainee’s patient care experiences, circumvents several known barriers to POCUS education including work-hour limitations and limited trainee hands-on experience opportunities. We propose that our elective serves as a model for IM residencies which have similar needs with respect to POCUS education.

**Supplementary Information:**

The online version contains supplementary material available at 10.1186/s12909-025-06802-x.

## Introduction

There has been increasing attention on point-of-care-ultrasound (POCUS) education in Internal Medicine (IM) residency programs [1]. A comprehensive approach to deliver such education, however, has not yet been established [2–4]. Some curricular designs aim to front-load POCUS education at the beginning of IM residency, either through a single workshop or multiple sessions over several weeks [5]. Other designs incorporate POCUS education longitudinally into the residency program [6–8]. One study found that following an ultrasound workshop at the beginning of IM residency, residents randomized to a monthly ultrasound curriculum retained image recognition skills better than those who had only attended the workshop [9]. Another model described a dedicated track to residents interested in obtaining greater POCUS training, utilizing external resources such as a national certificate program [10].

Successful retention of POCUS image recognition can be achieved through implementation of online didactics [11]. Image acquisition has effectively been taught with hands-on instruction [12]. POCUS-guided procedural training has been taught effectively through simulation [13]. Although these techniques have been effectively implemented, alone they have not met the criteria for a model POCUS program suggested by the Alliance for Academic Internal Medicine (AAIM) in a position statement. These criteria include: integration across the longitudinal training spectrum; integration several different types of skills, knowledge, and behaviors; supervised by trained faculty; and integrated into trainee’s patient care experiences [3].

Barriers to a complete POCUS education that satisfies these criteria include limited trainee hands-on experience opportunities, persistent variability in educational experiences during residency and a lack of available experts [14, 15]. There exists a body of literature that delineates barriers to POCUS education. A recent review noted similar barriers to POCUS education as noted above, adding inadequate technology, insufficient quantity of practice cases and knowledge about documentation [16]. A national survey in the Netherlands showed that despite near-universal agreement that POCUS is a useful skill for IM physicians, the majority of residents received less than 10 h of POCUS training [15]. A recent article describes a POCUS track for IM residents, using national POCUS certification programs to circumvent the known barrier of insufficient POCUS-trained faculty [10].

An initial needs assessment (Additional file 1) was conducted two years prior to the implementation of the POCUS elective at our institution. 47 residents spanning post graduate year (PGY)-1, PGY-2 and PGY-3 completed this needs assessment. This cohort represented 52% of the IM residents matriculated at the time. Needs reported included: POCUS training with a fellow or attending and POCUS training in each exam type including bedside echocardiography, vascular access and diagnostics, chest US, and abdominal US. We created and implemented our curriculum based on the needs reported by our residents.

The pedagogy of the curriculum was aligned with the results of the needs assessment and guided by the directives in the AAIM position statement POCUS for IM residency training [3] and the American College of Chest Physicians (ACCP) consensus statement on competence in critical care POCUS [17]. The curriculum encompassed all components of the indication, acquisition, interpretation, and medical decision making (I-AIM) model for POCUS training [18]. Learning points included: knowledge of ultrasound physics; machine control, setting and transducer selection and manipulation for each indicated image; knowledge of normal and abnormal POCUS findings; and knowledge of image interpretation and clinical applications. Technical and cognitive skills requirements for each exam type were also considered as per the consensus guidelines for elements required for competence and medical decision making. This elective was designed as five days of organized instruction and could be considered the first phase of a longitudinal training with the aim of achieving competence as per above referenced guidelines.

## Methods

The elective was a five-day (one work week) dedicated clinical POCUS rotation for IM residents and is being evaluated after two years of implementation at our institution. We scheduled the elective rotation from 9 A.M. until 5 P.M. for five consecutive workdays. For each of these one-week elective periods, there were two scheduled residents, hereby referred to as “POCUS residents”. During this week, our residents took part in self-directed learning and supervisor-facilitated hands-on learning at the bedside during daily patient interactions. Specifically, self-directed learning took place through reading assignments as detailed in the provided curriculum document (Additional file 2). Supervisor-facilitated learning occurred at the bedside targeting learning objectives also outlined in the curriculum.

The elective setting was our institution’s medical intensive care unit (MICU). No prerequisite knowledge was required from the residents. Supervising faculty were attending physicians who completed formal POCUS training in residency, fellowship or at the attending level. Supervisors were trained to follow a standardized method of ultrasound instruction (Additional file 3). Participating residents were provided a comprehensive resource: The Stony Brook Ultrasound (SBUS) Manual, organized for the purposes of this elective into daily reading assignments (Additional file 4) and several quick reference guides (Additional file 5).

Equipment employed was a portable ultrasound unit approved by our institution for patient care. The unit was capable of motion mode (M-mode) and brightness mode (B-mode) and came equipped with a high-frequency linear array transducer probe and a low-frequency phased array transducer probe.

### Ethical considerations

Patient interaction took place during this elective. Formal patient consent was not required by the institution’s Institutional Review Board (IRB), although patient or surrogate permission was acquired prior to ultrasound exams. Prior to the POCUS exam, the accompanying attending physician would verbalize to the patient and any present family members the intent of performing POCUS for resident education and that any clinically relevant findings would be immediately conveyed to the patient’s care team. If a patient and/or health care surrogate did not agree to participate, the intended teaching points would be covered while performing POCUS with another patient. All ultrasound imaging obtained was stored in a manner compliant with hospital patient privacy standards and human subjects policies. Some POCUS images were considered for use in patient care, after review by the supervisors and at the discretion of the MICU team. Images gathered for the purposes of education only, however, were not incorporated into patient care or the health records. Unexpected findings were promptly conveyed to the primary attending physician. A formal evaluation of this elective was approved by the Stony Brook University Institutional Review Board (IRB Reference #2018–4621).

### Daily schedule

From 9 A.M. to 12 P.M., each POCUS resident participated in morning rounds as the designated POCUS user for specific cases. After listening to the case presentation, the resident employed targeted POCUS methods relevant to the case, guided by the supervising faculty. The resident then presented findings to the team and aided in patient diagnosis and management plan development. The faculty was present in the room to ensure quality of images and to teach image acquisition skills during the patient interaction.

At the end of morning rounds, the supervising faculty selected images and videos that were targeted to the topics covered that day and questioned learners in a round-table format. Discussion took place and questions were encouraged from the learners. A lunch break was allotted from 12 P.M. to 1 P.M. From 1 P.M. to 2 P.M., POCUS residents attended their daily resident conference. Upon returning, from 2 P.M. to 5 P.M., they reviewed POCUS material associated with current and next-day learning points and wrote POCUS Consult notes into the electronic medical record (EMR) for patient interactions of that day. Each day’s relevant reading assignments were from a single comprehensive resource, the Stony Brook Ultrasound (SBUS) Manual, made available for reference in supplemental material (Additional file 4). Requirements for the POCUS Consult EMR notes and an example are provided (Additional file 6).

A detailed checklist of step-by-step daily requirements is provided (Additional file 7). This checklist was marked by the faculty to assure completion of daily activities by each POCUS resident. In this manner, we ensured consistency in learning experience.

### Daily learning points

Each day of the elective included specific learning points grouped together within associated organ systems. Full learning objectives are available in the provided curriculum document (Additional file 2).

Day 1 focused on introduction to ultrasonography, vascular access, and vascular diagnostics. Example learning objectives included understanding the basics of ultrasound (US) physics, use of the US machine, using US to obtain vascular access and submitting images for review. As part of the introductory materials, on Day 1 POCUS residents completed a written, 20 min, pre-course clinical knowledge assessment (Additional file 8). After completing this quiz, prior to starting their morning rounds at 9 A.M., the residents were given POCUS “Pocket Reference Cards” (Additional file 5) to serve as reference items during their rotation.

Day 2 focused on chest US, i.e. lung, pleura, diaphragm, and airway. Learning objectives included learning technique, image acquisition and interpretation for lung imaging, using POCUS for airway management, and identifying a safe site for pleural access. Day 3 placed focus on abdomen, retroperitoneum, hemodynamic monitoring, and abdominal scanning technique. Learning objectives included identifying abdominal anatomy, performing a Focused Assessment with Sonography in Trauma (FAST) exam, identifying a safe site for abdominal access, and interpreting inferior vena cava (IVC) measurements. Day 4 focused on bedside echocardiography. Learning objectives included identifying anatomy and the clinical utility of basic echocardiography views and evaluating left ventricular and right ventricular function. Each day’s assigned readings corresponded to the topics covered the subsequent day (Additional file 4).

Day 5 was the final day of the elective. Learning objectives included understanding protocols for diagnosis and management of respiratory failure, shock, diuresis and cardiac arrest. In addition to the usual daily schedule noted above, the POCUS resident presented at POCUS Journal Club at the end of this day. Journal club articles were selected from a list of landmark POCUS papers (Additional file 9) or a recent article that was pre-approved by the supervising faculty. This served the purpose of a robust discussion of literature related to POCUS use. At the end of journal club, POCUS residents took the same written 20 min post-course clinical knowledge assessment (Additional file 8). At the end of this day, learners were encouraged to ask to review any items of medical knowledge or hands-on skills they felt uncomfortable with. These items were addressed one-on-one with the supervising faculty.

### Comprehensive evaluation

Our evaluation of our POCUS elective for IM residents was structured by way of the Kirkpatrick four-level model framework, encompassing reaction, learning, behavior and results. Each of the four levels of the Kirkpatrick model are reported using a corresponding evaluation tool, including an evaluation survey (reaction), medical knowledge test (learning) and hands-on evaluation (behavior) which are all discussed below. Finally, the broader impacts on use of clinical POCUS at our institution (results) are also reviewed.

45 IM residents completed the elective in total. Data presented within this report is presented in cohorts, as not all POCUS residents completed all components of the evaluation. We administered an anonymous evaluation survey (Additional file 10) to POCUS residents who had participated in the elective rotation. The survey addressed organization of the rotation, quality of resources, quality of teaching, case diversity, faculty availability, balance of supervision and autonomy, and curriculum goals met.

We evaluated POCUS medical knowledge by administering a post-course written exam (Additional file 8) that tested ultrasound physics, machine control, image recognition and clinical applications of POCUS. For example, one exam question demonstrated a parasternal long axis view of the heart with an arrow pointing to the anterior leaflet of the mitral valve and required the trainee to identify the indicated structure.

For the technical skills evaluation and clinical application, the supervising faculty directly observed POCUS residents performing POCUS during rounds, taking part in management plan development where applicable, and contributing to image discussion at the end of rounds. The faculty completed a comprehensive requirements checklist (Additional file 7) that ensured each resident had successfully completed all aspects of each US technique during the week. At the end of the elective, the supervising faculty completed an end-of-course performance evaluation form which rated the residents’ ability to conduct each POCUS application by Day 5 (Additional file 7).

Additionally, resident documentation of POCUS consult notes for the week were reviewed by the supervising faculty to assess the residents’ ability to utilize POCUS in a daily clinical setting. These consult notes were similarly evaluated in the end-of-course performance evaluation form (Additional file 7). Residents also took part in a journal club at the end of the elective. During this journal club they were joined by their fellow POCUS resident and faculty supervisor. This performance was recorded in the end-of-course performance evaluation form (Additional file 7).

## Results

In total, 45 IM residents completed the elective. Of these 45 residents, 21 (46%) completed the evaluation survey, 30 (66%) completed the knowledge assessment, and 20 (44%) completed the hands-on skills assessment. Missing data was due to non-response in the case of the evaluation survey and time constraints and availability of the instructor in the case of the knowledge assessment and hands-on skills assessment, and did not relate to voluntary non-participation in the assessment. Demographics data was collected at the time of pre-course testing. Most participating residents reported less than 1 year of experience with POCUS, with remaining 4 residents reporting they had never used POCUS. No participating resident had any formal training in POCUS. All residents opted to participate in the POCUS elective as an optional elective.

### Elective evaluation

An evaluation survey (Additional file 10) was administered to assess the utility of and reaction to the rotation and was completed by IM residents who participated in the elective. 21 PGY-1, PGY- 2 and PGY-3 residents completed the survey. Survey data is provided (Table 1). 71% of residents rated the elective as outstanding at meeting curriculum goals while 29% rated it as above average. 57% of residents rated the organization of the rotation as outstanding and 33% as above average. Other domains assessed by the evaluation survey included quality of educational resources, equipment and teaching modalities.

**Table 1:** Elective evaluation survey (*N* = 21)

Evaluation statement	Outstanding	Above average
Organization of the rotation	57%	33%
Quality of simulation resources	67%	33%
Quality of other course resources	62%	19%
Quality of US machine and equipment	71%	14%
SBUS Manual	81%	14%
Hands-on teaching	95%	5%
Materials provided	81%	19%
Case diversity	81%	14%
Meeting curriculum goals	71%	29%

21 learners responded to the elective evaluation survey. Results are reported in percentage of respondents. Learners were asked to rate aspects of the curriculum as Outstanding, Above Average, Average, Below Average or Unsatisfactory

### Medical knowledge evaluation

**Fig. 1:**
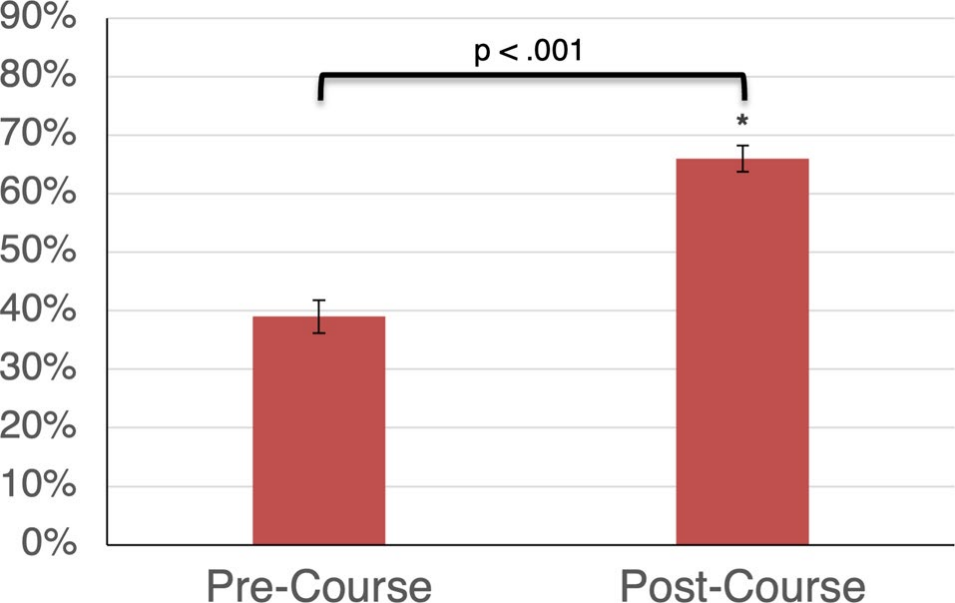
Medical Knowledge Scores at Pre-Course and Post-Course

30 PGY-1, PGY- 2 and PGY-3 residents completed both the pre-course and post-course medical knowledge exams (Additional file 6). Of the total 30 residents who completed the pre- and post-course exams, 12 (40%) were PGY-1s, 12 (40%) were PGY-2s, and 6 (20%) were PGY-3s. There was a significant increase in knowledge scores (*N* = 30, *p* < 0.001) after the US course. The pre-course mean was 39% (95% CI = 33.7–45.5%) and post-course mean was 66% (95% CI = 61.7−71.2%) (Fig. 1).

Composite scores on the medical knowledge exam are reported for PGY-1, PGY-2 and PGY-3 residents (*N* = 30). Pre-course and post-course means were compared with a paired t-test (*p* < 0.001). Error bars represent standard deviation of the data. The medical knowledge exam was previously internally validated in a population of first-year pulmonary and critical care fellow physicians. This population scored 29.9% on the pre-course exam and 69.5% on the immediate post-course exam of a year-long POCUS curriculum.

### Hands-on skill performance

20 PGY-1, PGY- 2 and PGY-3 residents completed the post-course skills exam (Additional file 9). A complete report of competency assessments and criteria for assessment level is provided (Table 2). In summary, 4 (20%) learners demonstrated an appropriate application of POCUS knowledge base under direct supervision, 6 (30%) with indirect supervision, 6 (30%) independently with 4 (20%) observed to perform in a manner compatible with an ability to supervise junior trainees. The same instructor was responsible for assessing all skills exams.

With respect to overall image acquisition capability, 7 (35%) learners could perform image acquisition accurately under direct supervision, 4 (20%) with indirect supervision, and 9 (45%) independently. With respect to image interpretation skills, 3 (15%) learners interpreted adequately under direct supervision, 8 (40%) with indirect supervision, 8 (40%) independently and 1 (5%) displayed competence at a level appropriate to supervise junior trainees.

Regarding the specific behavior of applying POCUS to performing image acquisition and interpretation within clinical context, 3 (15%) learners could perform under direct supervision, 8 (40%) could perform with indirect supervision, 7 (35%) could perform independently and 2 (10%) could supervise junior trainees.

**Table 2:** Observed skills assessment (*N* = 20)

Overall assessment	Cannot perform	Can perform under direct supervision	Can perform with indirect supervision	Can perform independently	Can supervise junior trainees
Vascular Access	0	13	6	1	0
Vascular Diagnostics	0	18	2	0	0
Chest - Lung	0	0	8	4	8
Chest - Pleura	0	2	8	6	4
Chest - Diaphragm	0	8	8	4	0
Basic Echocardiography	0	9	3	7	1
Abdomen and Retroperitoneal	0	2	7	9	2
Overall knowledge base in POCUS applications	0	4	6	6	4
Image acquisition skills	0	7	4	9	0
Image interpretation skills	0	3	8	8	1
Ability to apply POCUS knowledge, image acquisition, and interpretation within clinical context	0	3	8	7	2

20 learners were observed completing a post-course skills exam. Results are reported in absolute number of learners. Competency to supervise junior trainees was defined as being able to acquire image inclusive of appropriate gain, appropriate depth, appropriate probe positioning, and being able to identify key anatomical structures and pathologies with no verbal or physical guidance within 30 s while appropriately verbalizing their process. Competency to perform independently was defined as acquiring images and identifying structures and pathologies as above but taking longer than 30 s or not explaining their process. Competency to perform with indirect supervision was defined as acquiring images and identifying structures or pathologies with verbal guidance. Competency to perform with direct supervision was defined as acquiring images and identifying structures or pathologies with verbal and physical guidance such as co-holding the probe. A learner would be deemed unable to perform a skill if they were unable to acquire images or identify structures or pathologies with verbal and physical guidance. A single instructor was responsible for assessing all skills exams

### Institutional usage of educational resources and ultrasound use in the clinical arena

**Fig. 2:**
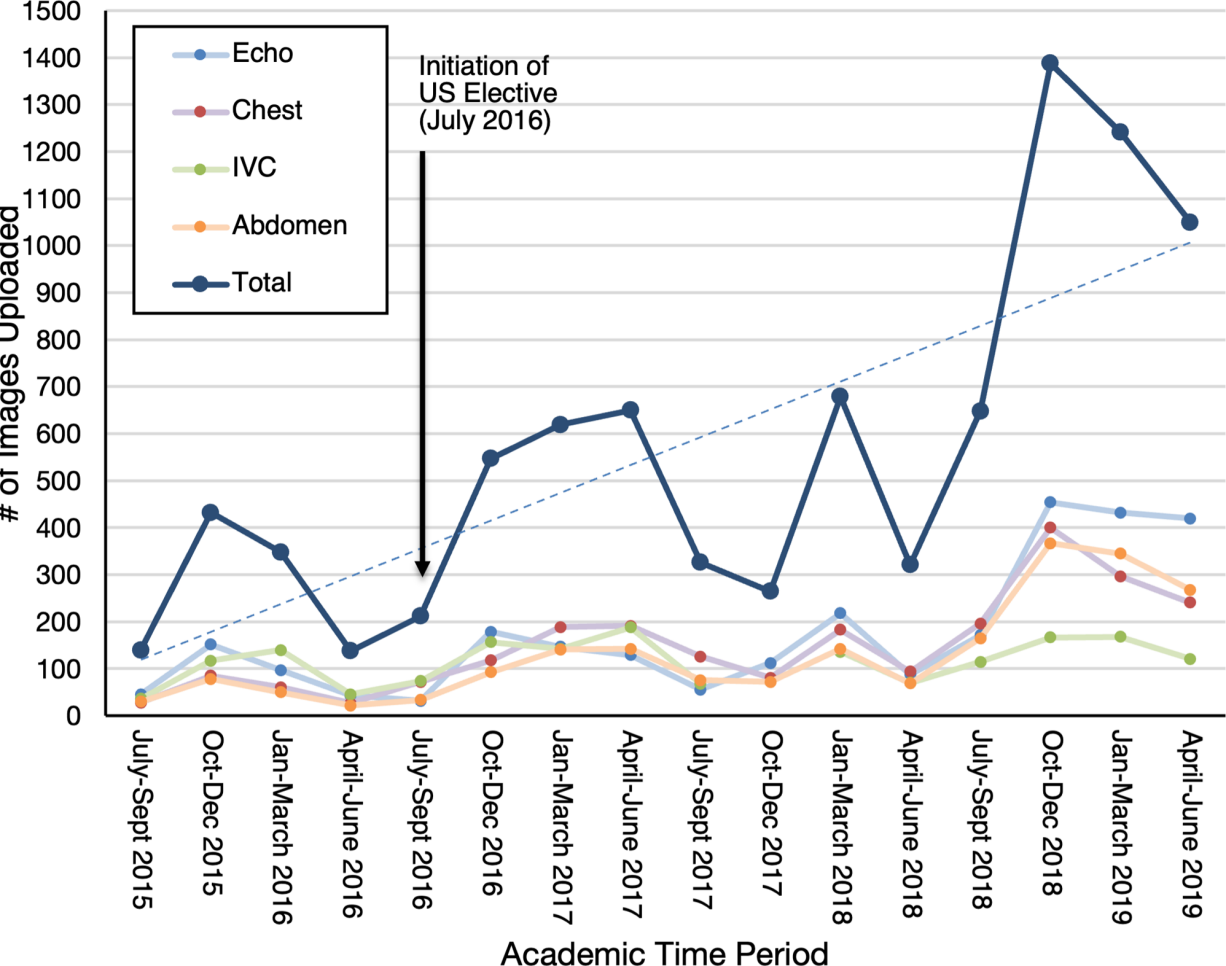
POCUS Images Saved by IM Residents from 2015–2019

All IM residents at our institution have access to POCUS and are recommended to save their images to a dedicated server where images automatically upload when saved. Quantity of images uploaded was recorded by type of scan (Fig. 2). The number of total images uploaded onto the server before the initiation of the US elective was 1058 images from July 2015 to June 2016. The US elective was first offered to all IM residents in July 2016 and every year since then. From July 2018 to June 2019, 4328 images were uploaded to the server, constituting a 409% increase in yearly images uploaded from prior to initiation of the elective. No other major clinical or educational programs in ultrasound occurred at our institution in that time period as related to the use of this image server.

POCUS images uploaded to a dedicated server by resident operators were partitioned into 4 categories of scans: echocardiography, chest, inferior vena cava (IVC) and abdomen. Datapoints represent total image studies saved over each 12 month academic year. The dashed line indicates the timepoint at which the POCUS elective was initiated. Following the initiation of the POCUS elective in July 2016, total uploaded POCUS images by residents increased at a rate of about 24 images per month while the number of residents remained minimally changed across academic years.

Additionally, recorded accesses to the SBUS Manual (Additional file 4) increased following the initiation of the elective. The elective was first offered in July 2016. In 2017, the SBUS Manual was accessed on average 97 times per month while in 2018 this figure rose to 145 times per month, constituting a 49% increase in monthly accesses to this institutional resource following the first complete year of the elective. Within our institution, faculty development initiatives arose shortly after the time period of this study. For example, a regular POCUS didactic series was started in our institution’s Division of Pulmonary and Critical Care Medicine. As an adjunct to this POCUS elective, POCUS education was later integrated into an existing simulation program at our institution’s IM residency. These impacts to clinical and educational programs at our institution are thought to be in large part a direct result of engaging the IM residents by way of this POCUS elective and represent institutional impact of our educational intervention.

## Discussion

POCUS education poses a unique challenge, requiring learners to integrate the technical aspects of machine control and image acquisition with medical knowledge and clinical reasoning to guide image interpretation. The end goal is for the trainee to become a technician-clinician. As such, we suggest that POCUS education needs to teach the trainee to efficiently navigate between the two roles, a technician and a clinician, in a clinical setting to provide quality patient care. We believe the effectiveness of this elective can be attributed to the integration of hands-on practice with didactics and multifaceted evaluation tools to ensure clinical and technical knowledge.

There remains a need for increased POCUS education at the resident level within IM residency training [15, 16]. Existing literature for POCUS education describes models inclusive of workshops that serve to introduce learners to POCUS at the start of IM residency or longitudinal curricula with learning interspersed into protected education time [5–9]. In contrast, our curricular model involves one week of focused POCUS education integrated into active clinical care. Such a design is grounded in Ericsson’s theory of deliberate practice, which has been shown to improve ability in other domains such as in music training [19, 20]. In this manner, this period of focused learning may accelerate the development of the learner [21, 22]. Despite this potential benefit to training of a dedicated POCUS elective, to further develop POCUS competency, learners must have sufficient opportunities to continue applying POCUS to clinical care to accrue experience, and must crystallize their medical knowledge through spaced repetition [9, 23]. Based on our experience, we suggest a longitudinal integrated pedagogy for POCUS training as follows: this five-day elective, followed by regular practice sessions, independent image portfolio development and maintenance, and educational opportunities to teach junior trainees culminating in summative competency testing.

Learners began with minimal POCUS knowledge with a pre-course mean score of 39% which improved to 66% (*p* < 0.001) at the end of the short elective week. Learners were observed to complete over 75% of skills with independence or with indirect supervision. These results suggest that medical knowledge was effectively gained because of the experience. Overall competency, a more comprehensive goal, was not tested and would surely need longitudinal experience to achieve, as mentioned above.

Our study is limited by participation bias, as our sample size was composed of 45 participating POCUS residents, and only subsets of the 45 total participating residents responded to the evaluation survey or completed the skills and knowledge assessments. Our study may be further limited by selection bias, as residents choosing to participate in the elective may have had a pre-existing interest in POCUS. The specific learning objectives of this elective may not be generalizable to programs where a comprehensive, longitudinal POCUS curriculum is in place, as our study assumed no prior knowledge and indeed many participants began with limited or no POCUS experience. We did not perform a follow-up assessment, thus we cannot say with certainty whether learners retained their acquired knowledge and skills.

We believe there has been impact on the institutional usage of POCUS. The usage of POCUS among the entire institution increased upon the initiation of the elective. Prior to the elective in 2015, there was only a limited use of US based on the images uploaded as depicted in Fig. 2. After the US elective was offered as an introductory course, residents became more likely to use POCUS in their daily practice. The largest increase was seen for recorded bedside echocardiography and chest US. However, our work does not explore any translation of the increase in recorded studies into improvements of patient care or workflow efficiency. Further research avenues to determining institutional impact include assessing direct patient outcomes as a result of increased POCUS utilization and assessing impact to healthcare costs and resource utilization.

Limitations to implementation include maintaining at least a small core of faculty, who must be trained in POCUS to be able to serve as POCUS faculty supervisors. There is also a need for a POCUS elective director who oversees and ensures quality work. A prior study incorporated a four-week POCUS elective as part of a dedicated POCUS track, but this was only offered to 4 IM residents, reinforcing a need for available faculty to operate a POCUS elective at scale [10]. There are national training courses for practicing attending physicians to serve these roles at their institutions. We also expect these limitations to resolve over time as the implementation of POCUS electives expand, such as ours, and as POCUS-trained residents become attending physicians who can fulfill these roles. Educational materials provided in this report may serve to attenuate a limitation to implementation due to lack of resources, which is a common limitation faced by IM electives.

Future iterations of studies assessing a POCUS elective for IM residency training could occur across multiple institutions with mandatory participation to encompass different settings and a wider and more diverse cohort of residents. Alternatively, randomizing participants to an elective or control group could further identify the benefits of the elective. Assessing the knowledge and skill retention at timepoints such as six months post-elective or one year post-elective would better assess the elective’s role of achieving long-term clinical competency. Finally, standardizing and recording both pre- and post-elective skills evaluations would better gauge technical competency of participants.

## Conclusions

POCUS training is a recognized need for IM residency programs. A model POCUS program is suggested to be supervised by trained faculty and integrated into trainee’s patient care experiences [3]. We have provided a supervised, integrated program in the form of a five-day (one work week) elective which not only meets these expected criteria, but because it is in the form of a scheduled rotation, circumvents several known barriers to US education including work-hour limitations, limited trainee hands-on experience opportunities, persistent variability in educational experiences during residency and a loss of motivation to learn. Following the Kirkpatrick model of educational experience assessment, this elective demonstrated success in all four levels of assessment. It was very well received, with course evaluations resulting as 57–95% “Outstanding” for course assessment domains (reaction). Medical knowledge increased from 29.9 to 69.5% (*p* < 0.001) during medical knowledge testing (learning). Learners demonstrated their newly acquired skills successfully, with majority able to perform image acquisition and interpretation with minimum to no supervision (behavior). Finally, there are signs of institutional impact including increased POCUS usage and subsequent educational programs in the five years following the initiation of this elective (results). For institutions looking to implement POCUS training within IM residency programs, this one-week elective model is appropriate either as a standalone intervention to provide a basis for future self-directed learning, or as a component of a longitudinal curriculum to accelerate acquisition of competencies.

## Electronic supplementary material

Below is the link to the electronic supplementary material.

Supplementary Material 1: Additional file 1 Needs Assessment

Supplementary Material 2: Additional file 2 US Elective Curriculum

Supplementary Material 3: Additional file 3 Tips for the Trainer

Supplementary Material 4: Additional file 4 Ultrasound Manual

Supplementary Material 5: Additional file 5 SBUS Quick Guides

Supplementary Material 6: Additional file 6 Consult note

Supplementary Material 7: Additional file 7 Resident evaluation and checklist

Supplementary Material 8: Additional file 8 Knowledge assessment

Supplementary Material 9: Additional file 9 JC Literature list

Supplementary Material 10: Additional file 10 Evaluation survey

## Data Availability

The dataset analyzed in this study is available from the corresponding author on reasonable request.

## Abbreviations

POCUS: Point-of-care ultrasound
IM: Internal medicine
AAIM: Alliance for Academic Internal Medicine
I-AIM: Indication, acquisition, interpretation, and medical decision making
US: Ultrasound
FAST: Focused Assessment with Sonography in Trauma
PGY: Post-graduate year
ACCP: American College of Chest Physicians
MICU: Medical intensive care unit
SBUS: Stony Brook Ultrasound
IRB: Institutional Review Board
IVC: Inferior vena cava
