# Supplementary material for: The design, performance and organizational impact of a point-of-care ultrasound (POCUS) elective for internal medicine residents
Source: BMC Med Educ. 2025 Feb 18;25:261. doi: 10.1186/s12909-025-06802-x (PMC11834687; doi:10.1186/s12909-025-06802-x)
Supplement: Supplementary file 1 — Supplementary Material 1: Additional file 1 Needs Assessment [file 12909_2025_6802_MOESM1_ESM.docx]

Ultrasound Current Use: Internal Medicine Residency Survey 1A, Survey 1B

1. What is your current position:

[ ] PGY-1 categorical [ ] PGY-1 prelim

[ ] PGY-2

[ ] PGY-3

[ ] Hospitalist

[ ] Fellow, please list type [ ] Other, please list

1. Have you completed any informal training in bedside ultrasound use? Check all that apply: [ ] Bedside training with attending

[ ] Bedside training with fellow

[ ] Bedside training with resident

1. Have you completed any formal training in bedside ultrasound use? Check all that apply [ ] Lectures

[ ] Simulation center session

[ ] Ultrasound elective

[ ] Ultrasound advanced elective

[ ] Training at a local or national conference

1. Have you completed training in the following procedures? Check all that apply [ ] FAST exam

[ ] Lung ultrasound

[ ] Abdominal ultrasound

[ ] Basic echocardiography [ ] IVC ultrasound

[ ] Ocular ultrasound for intracranial pressure assessment [ ] Renal ultrasound

[ ] Bladder ultrasound

[ ] DVT assessment using ultrasound

1. What is your experience and confidence in performing the following exams?

| Type of ultrasound procedure | Number of times performed monthly by you | If performed this procedure, your level of confidence in performing this exam  1 = Cannot perform even with direct supervision  2= Can perform with direct supervision  3= Can perform with indirect supervision  4= Can perform independently 5= Confident to perform and teach others | Your confidence level in interpreting findings of this exam  1 = Cannot interpret findings even with direct supervision 2= Can interpret findings with direct supervision  3= Can interpret findings with indirect supervision  4= Can interpret findings independently  5= Confident to interpret findings and teach others |
| --- | --- | --- | --- |
| FAST  examination |  |  |  |
| Lung  ultrasound |  |  |  |
| Abdominal  ultrasound |  |  |  |
| Basic Echocardiogra  phy |  |  |  |
| IVC  ultrasound |  |  |  |
| Ocular  ultrasound |  |  |  |
| DVT  assessment |  |  |  |
| Renal  ultrasound |  |  |  |
| Bladder  ultrasound |  |  |  |

1. Do you perform any basic procedural ultrasound examinations? Check all that apply [ ] Vascular access

[ ] Pericardiocentesis [ ] Paracentesis

[ ] Thoracentesis

[ ] Abscess drainage/localization

1. In the future, what do you expect regarding the trend in use of bedside ultrasound in the Department of Medicine?

[ ] Increased use

[ ] Same amount of use [ ] Decreased use

1. Do you think simulation training for ultrasound is beneficial? [ ] Yes

[ ] No

1. Would you like to see ultrasound training in IM residency continue? [ ] NO

[ ] Yes, the same amount of training is needed.

[ ] Yes, an increased amount of training is needed

1. Are you interested in more advanced ultrasound training?

□ Yes □ No
