# Supplementary material for: The design, performance and organizational impact of a point-of-care ultrasound (POCUS) elective for internal medicine residents
Source: BMC Med Educ. 2025 Feb 18;25:261. doi: 10.1186/s12909-025-06802-x (PMC11834687; doi:10.1186/s12909-025-06802-x)
Supplement: Supplementary file 2 — Supplementary Material 2: Additional file 2 US Elective Curriculum [file 12909_2025_6802_MOESM2_ESM.docx]

**Stony Brook Ultrasound:**

**Internal Medicine Residency Ultrasound Elective Curriculum**

**Curriculum Outline**

1. Description of Curriculum
2. Target Trainees
3. Prerequisite Knowledge and Skills
4. Reference Material
5. Elective Outline
6. Learner Objectives
7. Instructions
8. Equipment
9. End of Elective Responsibility

**Description of Curriculum**

*Course Overview*

In response to a needs assessment conducted internally, we have developed a one week ultrasound (US) elective to meet the needs of bedside US training at the Internal Medicine (IM) residency level. This is a one- week, patient and case- centered ultrasound training rotation, designed to meet the following objectives: Introduce the concepts of bedside ultrasound and its various applications for the IM practitioner; Teach image acquisition for basic applications to chest, heart, abdomen and retroperitoneum; Teach a directed strategy of bedside US application for common medical events including respiratory failure, bleeding and shock; And to teach interpretation of ultrasound images within the clinical context, and management decision making based on bedside ultrasound findings. All of these objectives are met through application- specific reading assignments, simulation and case- based hands- on teaching at the bedside. Reading and simulation assignments represent time spent in preparatory work and there is a marked emphasis on a patient- centered approach with hands- on experience at the bedside during medical rounds.

*Educational Rationale*

Bedside US use is becoming increasingly common for IM practice and is invaluable for patient care. Yet, there currently does not exist a comprehensive program that meets the US educational needs of IM residencies. Our innovative elective curriculum meets some of these training needs and has been a success at our institution. We place an emphasis on practical US application for the IM practitioner and the development of a proficient clinician-sonographer.

*Frequency of Course*

This is a one week course with daily US activities. This elective is offered approximately 20 weeks of the year for up to two residents per week- long rotation. In order to facilitate access to patients for this case- and patient- centered clinical rotation, the schedule for the rotation is oriented to match the on- service schedule of the course trainer.

**Target Trainees**

This elective is meant to serve the interests of those IM interns and residents who wish to become adept at bedside US for common IM clinical applications.

**Prerequisite Knowledge and Skills**

There are no specific requirements for students.

Qualification requirements of the trainer are: formal US training in residency or fellowship or completion of a formal national US training course. We have provided, within the online reference material, a chapter document that reviews best practices for US teaching, for the trainer’s further reference and for purposes of standardization of teaching.

**Reference Material**

Stony Brook University Internal Medicine US Manual:

*Day 1 Reading Assignment:*

1. Introduction- Prologue
2. Introduction to Ultrasound: The Basics
3. Chest- Evaluation of Pneumonia
4. Chest- Evaluation for Pleural Effusion
5. Chest- Evaluation of Pneumothorax
6. Chest- Evaluation of Pulmonary Edema
7. Confirmation of Endotracheal Tube Placement
8. Diaphragm- Evaluation of Diaphragm Function
9. Lower Extremity- Ultrasound to Rule Out DVT
10. Procedures- Internal Jugular Central Venous Catheter Insertion
11. Procedures- Ultrasound-Guided Peripheral Intravenous (PIV) Line Insertion

*Day 2 Reading Assignment:*

1. Abdomen- Ultrasound of Major Abdominal Vessels
2. Abdomen- Rule Out Abdominal Catastrophe
3. Abdomen- Evaluation of Gastric Content
4. AKI- Ultrasound Approach to Acute Renal Failure
5. Shock- IVC Examination for Fluid and Vasopressor Administration in Shock

*Day 3 Reading Assignment:*

1. Chest- Critical Care Echo

*Day 4 Reading Assignment:*

1. Respiratory Failure- Ultrasound Protocol- Based Approach to Respiratory Failure
2. Shock- Approach to Undifferentiated Shock
3. Shock- IVC Examination for Fluid and Vasopressor Administration in Shock
4. Cardiac Arrest- Ultrasound for Cardiac Arrest

**Elective Outline**

*Day 1:* **Introduction to bedside US, Vascular Access, Vascular Diagnostics**

US Introduction and Pre Course Clinical Knowledge Assessment

US rounds

*Selection of objectives based on patient cases available*

Reading Assignments: Day 1 Reading Assignments Document

US Consult Notes: *Based on patient cases available*

*Day 2:* **The Chest: Lung, Pleura, Diaphragm, Airway**

US rounds

*Selection of objectives based on patient cases available*

Reading Assignments: Day 2 Reading Assignments Document

US Consult Notes: *Based on patient cases available*

*Day 3:* **Abdomen, Retroperitoneum, Hemodynamic monitoring**

US rounds

*Selection of objectives based on patient cases available*

Reading Assignments: Day 3 Reading Assignments Document

US Consult Notes: *Based on patient cases available*

*Day 4:* **Bedside Echocardiography, US for Cardiac Arrest**

US rounds

*Selection of objectives based on patient cases available*

Reading Assignments: Day 4 Reading Assignments Document

US Consult Notes: *Based on patient cases available*

*Day 5:* **US Protocols**

US rounds

*Selection of objectives based on patient cases available*

*Review of any missed objectives (IM Resident US Elective: Evaluation Checklist must be completed)*

US Consult Notes Submission: Must submit all required cases as per the *Ultrasound Elective Consult Notes Required Image List*

US Journal Club

Post Course Clinical Knowledge Assessment

**Learner Objectives**

***Introduction to US***

***Vascular access***

***Vascular diagnostics***

Objectives:

- Understand basics of US physics
- Be able to manipulate US machine and optimize the image
- Identify clinical circumstances where US is indicated and advantageous
- Use US to enhance vascular access guidance
  - Tip Tracking
- Use US to improve safety in procedures
- Identify No-Go sites; re-orient to appropriate site for access
- Identify lower extremity deep vein anatomy
- Perform limited effective bedside DVT study
- Utilize new skill set in clinical scenario
- Submit images for review

***The Chest: Lung, Pleura, Diaphragm, Airway***

Objectives:

- Understand what characteristics of the thorax make it amenable to US
- Learn technique, image acquisition and interpretation for lung, pleura and diaphragm scanning
- Identify clinical circumstances where lung pleural and diaphragm US are highly applicable
- Understand how to use US for airway management
- Procedural control: Identifying a safe site for pleural access
- Utilize new skill set in clinical scenarios

***Abdomen, Retroperitoneum, Hemodynamic monitoring***

Objectives:

Abdominal scanning technique

- Identify anatomy
  - Hepatorenal recess
  - Splenorenal recess
  - Right and left paracolic gutters
  - Bladder
  - Kidney
- Abdominal aorta
  - Transverse plane
  - Longitudinal plane
- The FAST Exam: Understand where fluid/blood will accumulate
- Procedural control: Identify safe site for abdominal access
- Evaluation for pneumoperitoneum
- The IVC: Evaluate for fluid responsivity in shock state
- Introduction to bedside echocardiography:
  - Objectives of the bedside echo
  - The 5 basic views

***Bedside Echocardiography***

Objectives:

- Identify anatomy and clinical utility at basic echo views
- Evaluate LV, RV function
- Identify applicable views for common clinical scenarios
- Understand the role for bedside US for emergent airway control

***US Protocols***

***US for Cardiac Arrest***

- Become proficient in a protocolised approach to the role of US for diagnosis and management of:
  - Respiratory failure
  - Shock
  - Diuresis and fluid management
  - Cardiac arrest

**Instruction**

*Pre-test*

A pretest will be given before the start of the course. This multiple choice exam will contain questions about US physics, knobology, image recognition and clinical application. Images and videos tested will be particularly relevant to IM practice. In turn, these images will be recalled during practice sessions.

*Patient Scanning*

The learner attends rounds daily as the designated “ultrasound resident”. During rounds, the learner listens to case presentations and then performs targeted US during the physical exam. The learner then presents findings to the team and aids in patient diagnosis and management plan development.

The learner is expected to:

1. Introduce themselves to patients and patient care team members
2. Explain importance of procedures to patients

3) Maneuver the US machine within the room

4) Arrange the patient and room equipment safely

5) Perform US for the objectives given to them while controlling the machine and communicating effectively to patient (as needed) and family (if applicable)

6) Return the patient to their prior condition and sanitize the US machine

7) Communicate the findings to the patient and subsequently to the team

If there are junior trainees or other learners present during rounds, the resident is encouraged to verbalize techniques and reasoning to those members- acting as a teacher. The setting is a learner controlled environment but if the resident is struggling, the trainer may co-hold and guide the learner.

The trainer has a list of US objectives that the resident must complete daily in order to successfully complete the objectives checklist by the end of Day 5.

*Image Review Sessions*

The trainer will be present at the end of patient scanning sessions for discussion of US images acquired during rounds. The trainer will select images and videos that are targeted to the topics covered in that session and questions learners in a round-table format. Discussion will take place and questions will be encouraged from the learners.

**Equipment**

A portable US unit approved by the institution for patient care use will be employed. The unit will have the following capabilities: Motion-Mode (M-mode) and Brightness-Mode (B-mode). High-frequency linear array and low-frequency phased array transducer probes are also used.

**End of Elective Responsibility**

*Evaluations*

Residents are required to pass a written *Post Course Knowledge Exam* and have completed an *IM Resident US Elective: Evaluation Checklist* that spans the length of the course. This checklist is completed by the trainer and ensures each resident has successfully completed all aspects of each US technique during the week.

At the end of elective, the trainer further completes the *Ultrasound Elective Resident Evaluation Form* which rates the resident’s ability in each US application.

*Ultrasound Consults*

The resident is expected to produce US consult notes. Each note includes interpretation of US findings and recommendations to the care team if applicable. US consult notes are evaluated by the trainer in the *Ultrasound Elective Resident Evaluation Form.*

*Journal Article Review*

At the end of the course, the resident is expected to give an oral presentation on one journal article from the *Journal Club Literature List*.
