# Supplementary material for: The design, performance and organizational impact of a point-of-care ultrasound (POCUS) elective for internal medicine residents
Source: BMC Med Educ. 2025 Feb 18;25:261. doi: 10.1186/s12909-025-06802-x (PMC11834687; doi:10.1186/s12909-025-06802-x)
Supplement: Supplementary file 3 — Supplementary Material 3: Additional file 3 Tips for the Trainer [file 12909_2025_6802_MOESM3_ESM.docx]

Tips for the Trainer

*Overview*

To grow ultrasound at our institution, we focus on engaging all levels of learners and teachers. Below are some tips to learning and teaching ultrasound.

*Goals of the Trainer*

To facilitate image acquisition, image interpretation, and comprehension of findings within context.

*Principles for Effective US Skills Training*

- Be knowledgeable, be good
- Prepare beforehand: read, review cases, and scan
- Know your learners: level of medical education and training; level of US experience
  - For medical students: focus is on anatomy, physiology rather than clinical context
  - For residents, fellows: focus is on clinical applications of findings
  - For new learner: start with explanation of scan planes and introduction to machine, transducer; give a demonstration to show how to hold transducer (at its base; thenar eminence on patient), and scan technique (explaining how movements translate to changes on the screen of the field of vision)
  - For the experienced learner: initiate learner scanning immediately
- Know your assignments and learning objectives - if not allotted to you, must request ahead of time
- Read on the topic: regional anatomy, physiology, varying scan techniques
- Set up your station including machine settings, transducer, patient positioning, hand outs
- Scan the model prior to arrival of learners to the room
- Trainer hands off - Learner hands on model:
- Hold with the learner, rather than taking over the scanning (this facilitates learning probe control)
- Speak with standardized and carefully selected terms
  - Slide, rotate, rock, angulate
  - Cephalad, caudad
  - Anterior, posterior
  - Lateral, medial
- Define these terms to the learners at the start of the session
- Careful as some terms are relative to body and some to structure under investigation
- For advanced learners verbalize probe movements
- Answer question succinctly: redirect lengthy or too advanced questions
- Engage everyone in your room
  - Engage other learners with questions or discussions while one is scanning
- Identify (selectively) potential super users and new trainers.
