# Supplementary material for: The design, performance and organizational impact of a point-of-care ultrasound (POCUS) elective for internal medicine residents
Source: BMC Med Educ. 2025 Feb 18;25:261. doi: 10.1186/s12909-025-06802-x (PMC11834687; doi:10.1186/s12909-025-06802-x)

All images, videos and diagrams in this document are created by the authors.

Day 1

Introduction- Prologue

Kerim Odekon, MSIV

Putting physics back into the physical exam

Unlike many tests, which we order from a computer, bedside ultrasonography is an exercise that requires physical contact. Our sonographic findings are in the context of real-time interactions with our patients. Not only does ultrasound provide a direct window into a patient's anatomy and physiology, but the process itself allows for direct observation. As medicine transitions away from the bedside and into the laboratory via a maze of endless online orders, bedside ultrasound is a refreshing practice that re-orients modern medicine back towards the patient, the conditions they face, and highlights avenues for intervention.

Introduction to Ultrasound: The Basics

Lijo Illiparambil Chacko, MD & Sahar Ahmad, MD

The Physics of Ultrasound (The Basics)

The Piezoelectric Phenomenon

Piezoelectric elements can transmit sound when an electrical current is passed through them. This works in both directions where electrical current passed through these element crystals leads to the production of sound waves and the crystals interacting with sound waves leads to the release of electrical signals. These crystals are generally made of a quartz like material and modern US crystals are generally synthetically manufactured.

Transmission of Sound

While classically sound is pictured as a sinusoidal wave, sound is a pressure wave which leads to compression and rarefaction of particles transmitting sound energy. Without a medium of particles in close proximity, sound cannot transmit. Therefore, sound cannot be transmitted through media where the density is so low that it approaches 0 g/cm3 for example, in outer-space, there is no sound transmission at all. Similarly, US cannot be transmitted through ambient air due to the air particles being very far apart (hence the need to eliminate the layer of air between your probe and your patient's body by using gel as a coupling agent).

The speed as to which a medium transmits sound is chiefly dependent on its density, where speed of sound is higher through denser media due to the proximity of the particles participating in compression and rarefaction. For instance, air has a density 1.225 $x10^{-3}g/cm^3$, water 1 g/cm^3, and steel 8.05 g/cm^3. In comparison, blood is 1.0428 g/cm3, bone 1.75 g/cm3, adipose tissue 0.9094 g/cm3, and muscle 1.0599 g/cm3. The density affects how fast ultrasound waves move within the medium. Since sound is a progression of waves which compress and decompress as it propagates, it can achieve its speed based on how close together the molecules are and the elastic properties of the object. In air for instance it is calculated at 343 m/s or 767.269 mph. Water is 1498 m/s or 3340.5 mph, in steel sound travels at 5960 m/s or 13,290.8 mph. In soft tissue it estimated that the speed of sound is approximately 1540 m/s or 3434.2 mph. This is the speed that the US machine assumes all the sounds waves are moving.

The Interface

The interface is the moment where two media, each with its own inherent properties which affect the propagation of ultrasound waves, meet. It is the behavior of the beam(s) at the interface that

determine the US image. There are several behaviors of the US beam as it comes to the junction (interface) of two media.

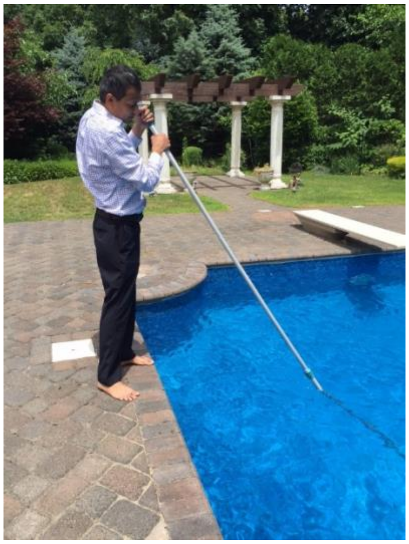

Attenuation

Attenuation refers to all of the events at an interface which result in the overall decrease in collective energy of the set of beams sent out from the transducer. Generally, it refers to all of the behaviors mentioned, other than an echo (180 degrees reflection back to the source) or transmission (where the beams continue forward unaltered, until the next interface is reached; such as is the case with blood or bodily fluid). Except for the returned echo and the soon to return transmitted beams, all of the other beams are lost, and the beam packet is attenuated.

Refraction of light beams across the air- water interface makes the pole appear bent; similar phenomena can happen with sound beams.

Acoustic Impedance

Acoustic impedance is the fundamentally connected to attenuation as it is the tissue's own impedance that determines the character of the return echo. This becomes especially important between tissues with different impedance. At these points, the boundaries themselves can create an echo with a change in the characteristics of the attenuation in the next tissue. With that there are 4 principle ways the sound waves behave at the boundaries between tissues.

First is the echo which reflects the sound waves. This is what the probe sees when it looks at the image. The second is transmission where part of the wave that continues forward through the new medium. The third is scatter, where at the boundary the sound wave moves in multiple directions from an originating point. Lastly is refraction, this is when the wave is transmitted through but at a different angle. This is can visualized when a pencil is placed in water, the slight deviation from where our mind expects the pencil to be (see above figure).

Putting all this together gives you how an ultrasound behaves with the human body. Air is a lighter substance with scattering. Fluids tend to be dark in color with because there is an echo and transmission through them. Solid organs then to be a mix of all aspects of the transmission. This leads to the heterogeneous appearance of solid organs. Bone echoes back sound or absorbs all sound, leading to a dark shadow under the surface of the bone itself.

The Creation of the Image

The images that we see on ultrasound are created via a simple mechanism occurring very fast. Simply the probe receives electricity which causes the piezoelectric material to release sound waves. This sends out a wave that meets interface of medium A, which the sound interacts with (via sending an echo, transmission of the sound through, scattering or refraction). As it passes through medium A it will have a certain level of attenuation and an impedance, as what's left of the sound waves continues it will meet with a new boundary which in turn will release a new echo.

The echo returns to the probe and causes the crystals to contract (due to the piezoelectric effect) which releases electrons which the probe sends to the base to analyze and display on the screen. What causes the appearance of the different structures is the intensity of the echo returning. It is a very simple read based on grey scaling. Where the stronger the echo the stronger the compression of the piezoelectric crystals which in turn means the more intense the electron release or larger the amplitude. The structural base reads these amplitudes and layers it layer by layer as echoes return. It can accomplish this due to the machines inherent code stating all the sound moves at 1540 m/s, so it assumes that the next wave is a deeper structure. However invariably there is a limit to how much we are sampling this limit is known as the Nyquist limit and because of it the US can demonstrate paradoxical images. This is due to the under-sampling by the probe which leads to leads to the appearance of a flow going in the direction opposite than to the real speed. An example of this is seen with helicopters (see video), and with car hub caps. Because cameras, and our eyes as well have their own sampling speed (in video terminology its frames per second), objects moving faster than the sampling rate appear to move in the opposite direction because they move so fast that the frames we see are ahead of what our minds expect.

The characteristics of the structure images on screen that the returning US beams (echoes) create is based on two variables: the frequency and the probe type. The term ultrasound means sounds above that which a human ear can hear. Normal hearing range can range from 20 hz to 20,000 hz. The frequency of the probe ranges in Megahertz or 1 million hertz. The range of these probes range from 1 Mhz to 100 Mhz. These different frequencies have different capabilities, with lower frequencies able to penetrate tissue deeper, but has lower resolution. Whereas higher frequencies allow for clearer pictures but do not penetrate the tissue as well.

Introduction to Ultrasound: The Clinician- Technician

Lijo Illiparambil Chacko, MD & Sahar Ahmad, MD

Familiarizing Oneself with the US

There are many models of US machines. Most common machine US machines contain a phased array probe and a linear array probe. They have the option of B- Mode, M- Mode, Color Doppler and PW and CW Doppler. They can also do echocardiography calculations. Some more updated machines also contain teaching videos. Further, there are portable US machines available that are laptop-like and contain a range of options. Last, there are also pocket sized portable US devices that have a phased array probe and linear array probe and contain B- Mode and Color Doppler.

Familiarizing oneself with the US machine is crucial for proper use. Below are some key functions that every beginner sonographer should know.

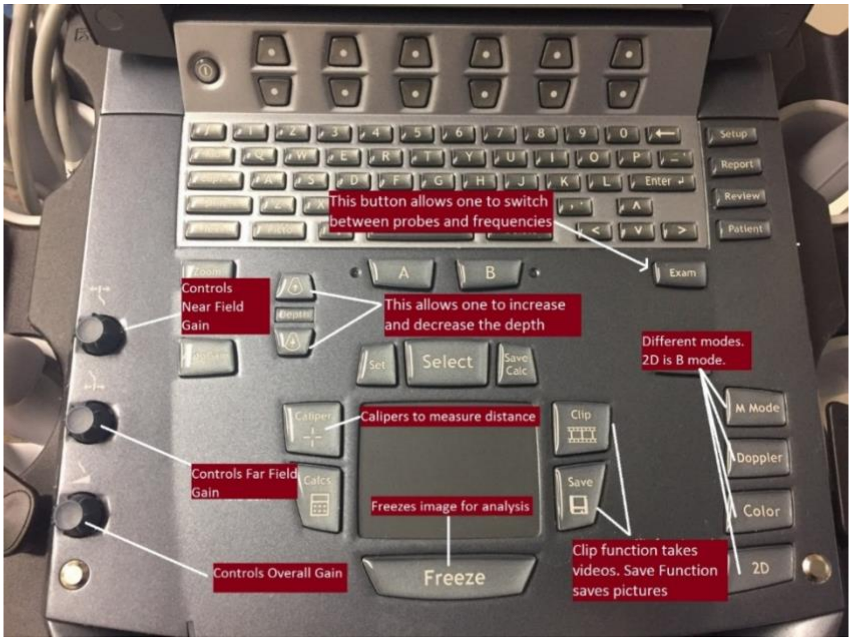

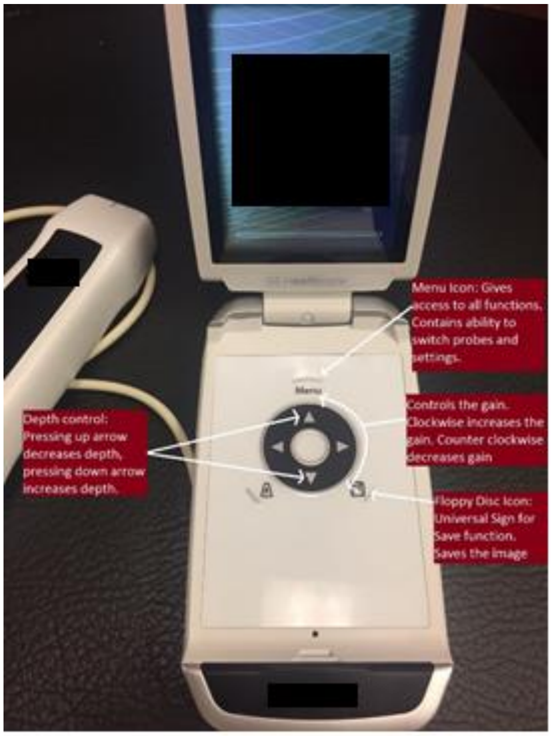

Creating an Image

<u>Transducer/Probes</u>

Linear, High Frequency Probe

This probe is best designed to look into superficial structures in great detail. It provides a rectangular image straight on both sides. It is a high frequency sound measured in Mhz (10-15 Mhz). Normal hearing range is 20 Hz to 20kHz, this ultrasound works on the order of 50,000 – 500,000x higher. Generally at Stony brook this is used for assess superficial veins, nerves, and pleural membranes.

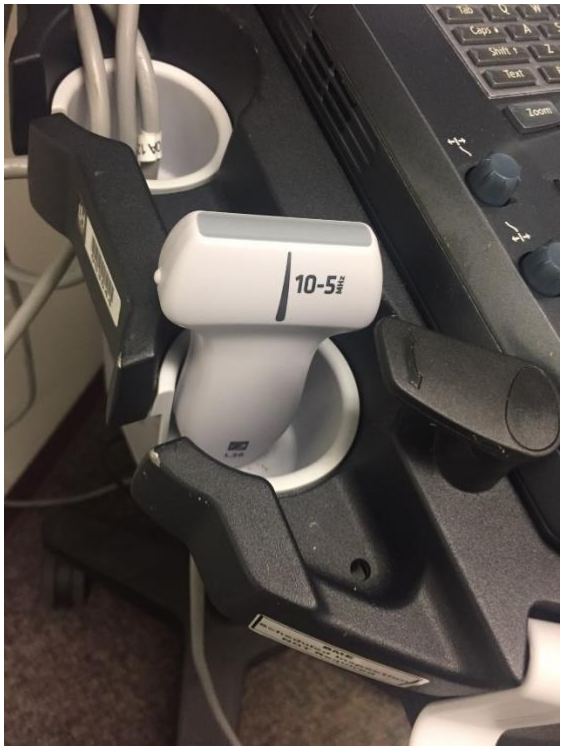

Phased array, Low frequency

The phased array probe is generally a probe that releases sound from multiple zones at the same time. Each set of beams is then phased out such that a large view is obtained through a small probe footprint that can "see" in between ribs. Additionally, the returning sets of phased beams take advantage of the principles of constructive interference and destructive interference. The constructive interference leads to an amplified signal, and the destructive interference leads to image sharpness. This is a low frequency probe generally associated with cardiac and lung imaging, especially designed for cardiac echo where the sonographer needs to visualize through the small rib space (the small footprint allows for this) and the images must be optimized for

discrete valvular and other motions (harmonics, constructive, destructive imaging is taken into account).

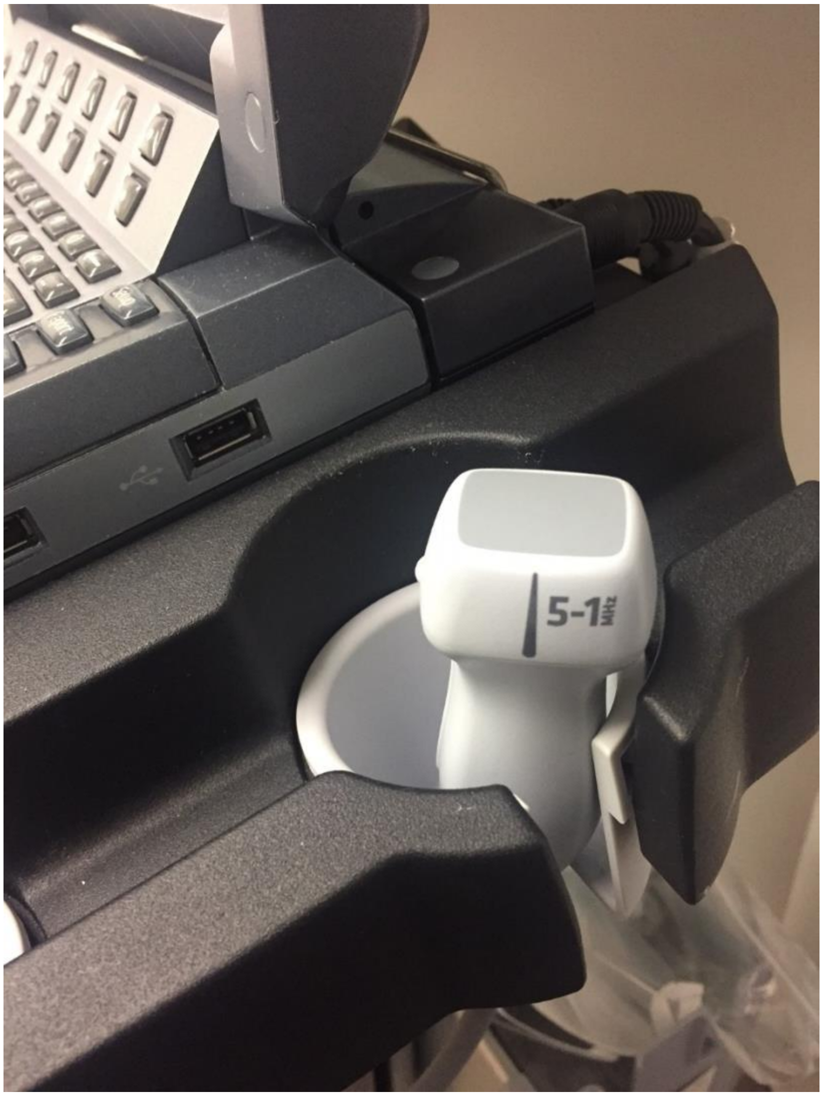

Curvilinear (mix between liner and phased).

A curvilinear probe is a mix of the two with linear in the center with phased array at the sides. Low frequency beams are sent out in linear fashion at the center and are phased at the edges of the beam set. Its best use is for abdominal and retroperitoneal organ viewing, where there are no ribs to create shadow.

Endocavitary probe

This is generally used in OB/GYN procedures and is a probe that can be placed in cavities if necessary. This is generally a low frequency probe specially designed for insertion into a body cavity with the transducer face shaped closest to that of the curvilinear.

Other probes include the Transesophageal Echocardiography (TEE probe), used to evaluate the heart from the posterior view of the esophagus; the Linear and Radial Endobronchial Ultrasound (EBUS) probes designed to look through the bronchial walls; the Endoscopic ultrasound (EUS) which views masses and lymph nodes through GI tract; and Intravascular Ultrasound (IVUS) which views plaques form within the coronary vessels. These probes are small but powerful, and are used in conjunction with invasive procedures.

The Image

The images themselves are 2D representations of 3D structures that are moving through time. These 2D images are based on the plane that the sound waves enter. These planes are based on the anatomical "cuts that a body can undergo: Coronal, sagittal, or transverse. On the ultrasound screen, one uses probe marker to orient oneself to the image. Either represented as a dot or "M" the marker gives us a reference point to work with and by convention are labeled by operator's visual orientation. As such the marker can orient use not only in direction, whether objects are cephalad (towards head), or caudal (towards tail), medial or lateral, but also we can understand whether an object is superficial or deep are relative to the probe. For proper imaging technique, the object of interest should be in the middle of the screen with good contrast. This is controlled with the depth, and gain.

Optimizing the View: Depth & Gain

Depth which is measured in centimeters, allows one to place the image in the center of the screen. This can help clinician mind of the ultra sonographer orient oneself and place the items of interest in the best view. It is adjusted with the depth buttons which depending on the machine can be located in different regions. The depth is increased and decreased simply but the time of listening that the ultrasound accepts. Listening for longer indicate that further away echoes can return to the transducer. In the table below we demonstrate the importance of depth in a case of looking at the hepatorenal recess.

Description	Image	Limitations and ways to fix
When the depth is too shallow it is difficult to identify localized regions and/or structures, for instance in the picture to the left is this a picture of a hepatized lung overlying the diaphragm or is this the liver and kidney at the level of the hepatorenal recess.	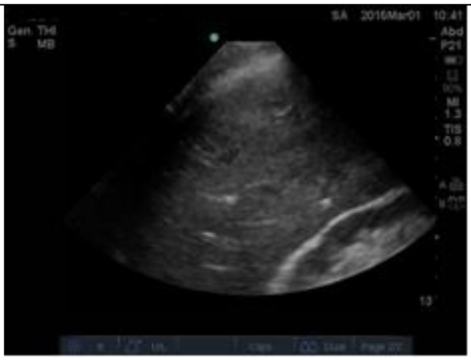	Limitations include the ability to miss important aspects of the surrounding tissues. For instance there may be a small collection of fluid in the hepatorenal recess that this image misses. Increase the depth and get a better picture.
When the depth is too large, there are extraneous and unnecessary images in view. For instance in the picture to the left there is a shadow of the vertebrae which takes up over half the screen. A distracting area when the focus may be the hepatorenal recess.	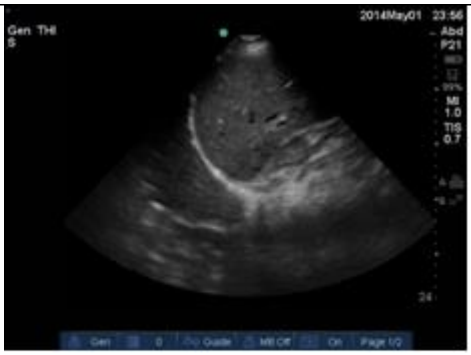	Limitations include inability to assess the structures of interest in appropriate detail. In this case the echogenicity of the kidney’s medullary region is not uniform. Does that mean it’s the kidney? Or is it a mass? Decrease depth the depth to get a better picture.
This is the appropriate depth which gives good visualization of the hepatorenal recess and adjacent structures.	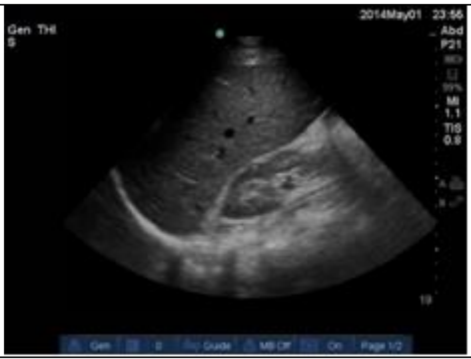	

Gain can be described as the amount of amplification applied to each signal. This means simply that the US increases the strength and intensity of the signal echo it receives to appear on the screen as brighter; simply put the signal gains strength. For gain if there is too little gain, then the image is dark and difficult to see, however if on the other hand the image is bright one can lose the distinction between interfaces. See figures below as examples of too little or too much gain.

Description	Image	Limitations and ways to fix
Near Field Gain is lower, making the top half of the image look darker.	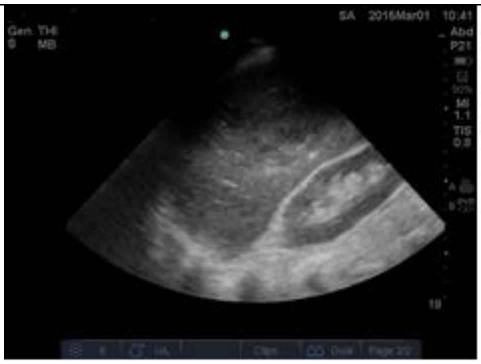	Unable to appropriately assess the closer structures. Will need to fix the near field gain first, and then once equilibrated can adjust total gain.
Far field gain is lower, making it bottom half of the image look darker	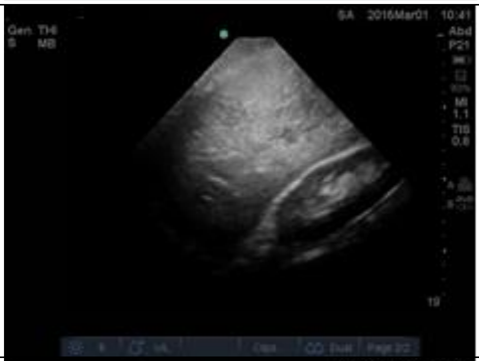	Unable to appropriately assess far structures. Will need to fix the far field gain first, and then once equilibrate can adjust total gain
Normal image with appropriate gain (may be a little too much gain overall)	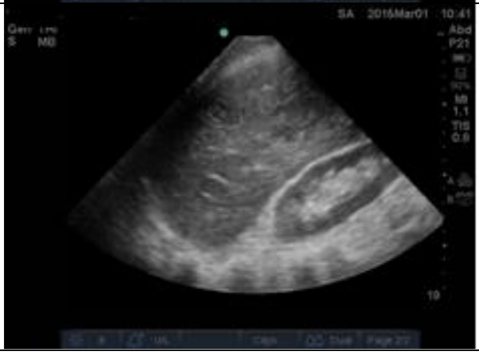	Overall gain is compatible, may adjust total gain to appropriate level

Chest- Evaluation for Pneumonia

Sahar Ahmad, MD

Pneumonia (PNA) is a clinical diagnosis. If your patient presents with the right clinical context: dyspnea, hypoxia, fever, cough, sputum and leukocytosis: your diagnosis is made. If you have imaging support of your diagnosis then this is further conclusive; however, it is not required for management. This is true for an infiltrate on Chest X-Ray (CXR) or Computed Tomography (CT) of the chest and certainly holds true for correlative findings of bedside Ultrasound (US).

There are several distinct US findings that suggest PNA and which in the correct clinical context are indicative of PNA, which will be outlined in this chapter.

Unilateral B Lines

The B line is an air artifact which arises from the inferior edge of the visceral pleura. It is an artifact created by lung parenchyma, which is edematous (e.g., pulmonary edema), fibrotic or inflamed (e.g., pneumonitis), congested (e.g., a local atelectasis), or infiltrated (e.g., PNA). The B line itself is a nonspecific artifact; the above named conditions tend to be bilateral pathologies except for PNA, which is commonly a unilateral pathology. In the right clinical context, a unilateral B-line pattern (B-lines at one anterior hemithorax and A-lines at the contralateral hemithorax, refer to page 26) seen at the anterior chest using a low-frequency transducer probe supports a diagnosis of PNA.

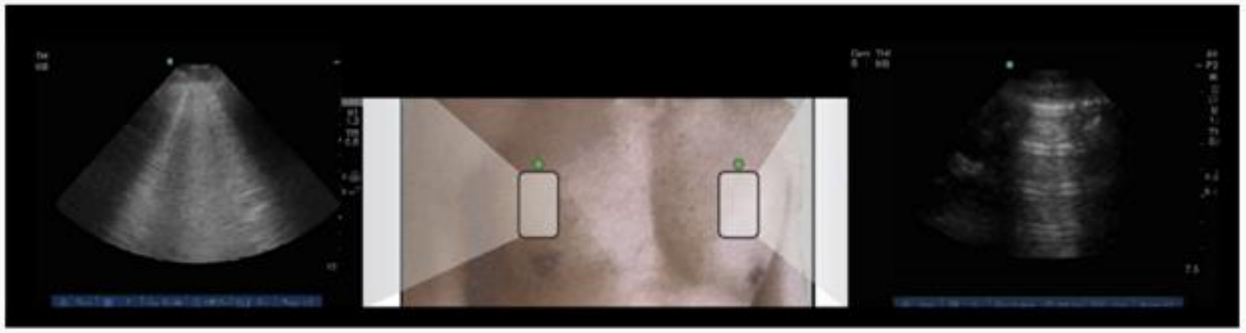

Unilateral B-Lines

- Location: Anterior Chest
- Probe: Low Frequency Phased Array
- Probe Marker: Cephalad
- Machine Setting: Abdomen or Lung
- Screen Marker: Operator-Left
- Screen depth: 8-14 cm

Subpleural Densities

Inflammation often reaches the peripheral edge of the parenchyma when there is a nearby PNA. By US this appears as subpleural densities. This is a tissue dense (dark-grey) rounded area at and immediately deep to the pleura. Locate two rib shadows and find the pleural surface as a linear echogenic (white and bright) line approximately 1-cm deep to the surface of the rib's periosteum and in between the two rib shadows. A subpleural density appears as a grey rounded area that remains immediately deep to the pleural surface and moves with respiration. It is a common but not ubiquitous finding in different forms of lung inflammation including pneumonitis, viral, and bacterial pneumonias.

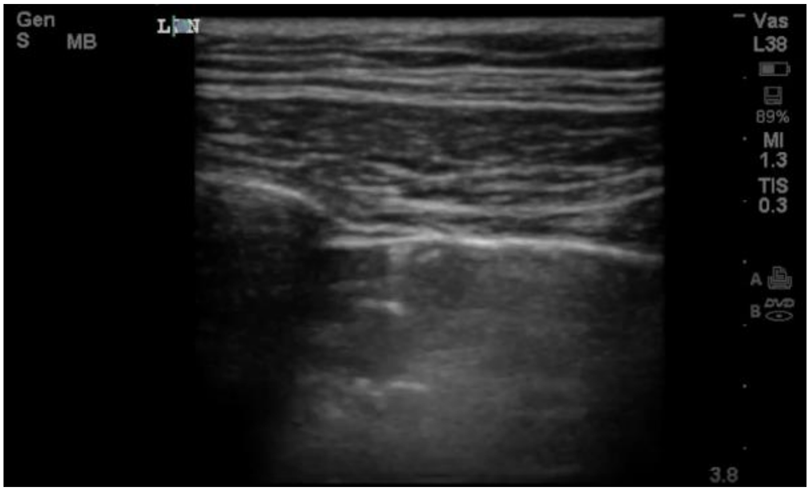

Subpleural Density

- Location: Anterior Chest
- Probe: High Frequency Linear Array
- Probe Marker: Cephalad
- Machine Setting: Abdomen or Lung
- Screen Marker: Operator-Left
- Screen Depth: 3-5 cm

Hepatization of The Lung

The term hepatization refers to a condition where the lung has taken on the echogenic appearance of liver, that is, it has become tissue-dense solidified. This is a finding associated with lobar collapse or lobar infiltration of the lung with pus (lobar PNA). A collapse is a volume-loss event of the lung and will be associated with an elevated placement of the diaphragm (often near the axilla itself when scanning at the anterior/mid axillary line), decreased ipsilateral chest wall expansion, and decreased robustness of lung sliding on the ipsilateral side. Pneumonic infiltration (consolidation) when severe and situated posteriorly can be picked up by US as a hepatized lung. It is most important that the liver itself not be misconstrued as a hepatized lung, as the terms suggest, these are similar in appearance. To avoid a common pitfall, always use the kidney as your vantage point. After first identifying the kidney, scan cephalad one or two rib

spaces viewing the liver/spleen and then the diaphragm, until the lung parenchyma can be viewed cephalad to the carefully established location of the diaphragm. A hepatized lung will look grey and solid-dense with white linear markings within it, rather than the usual aeration artifacts that we are used to seeing (A- and B-lines). The white linear markings are known as air bronchograms.

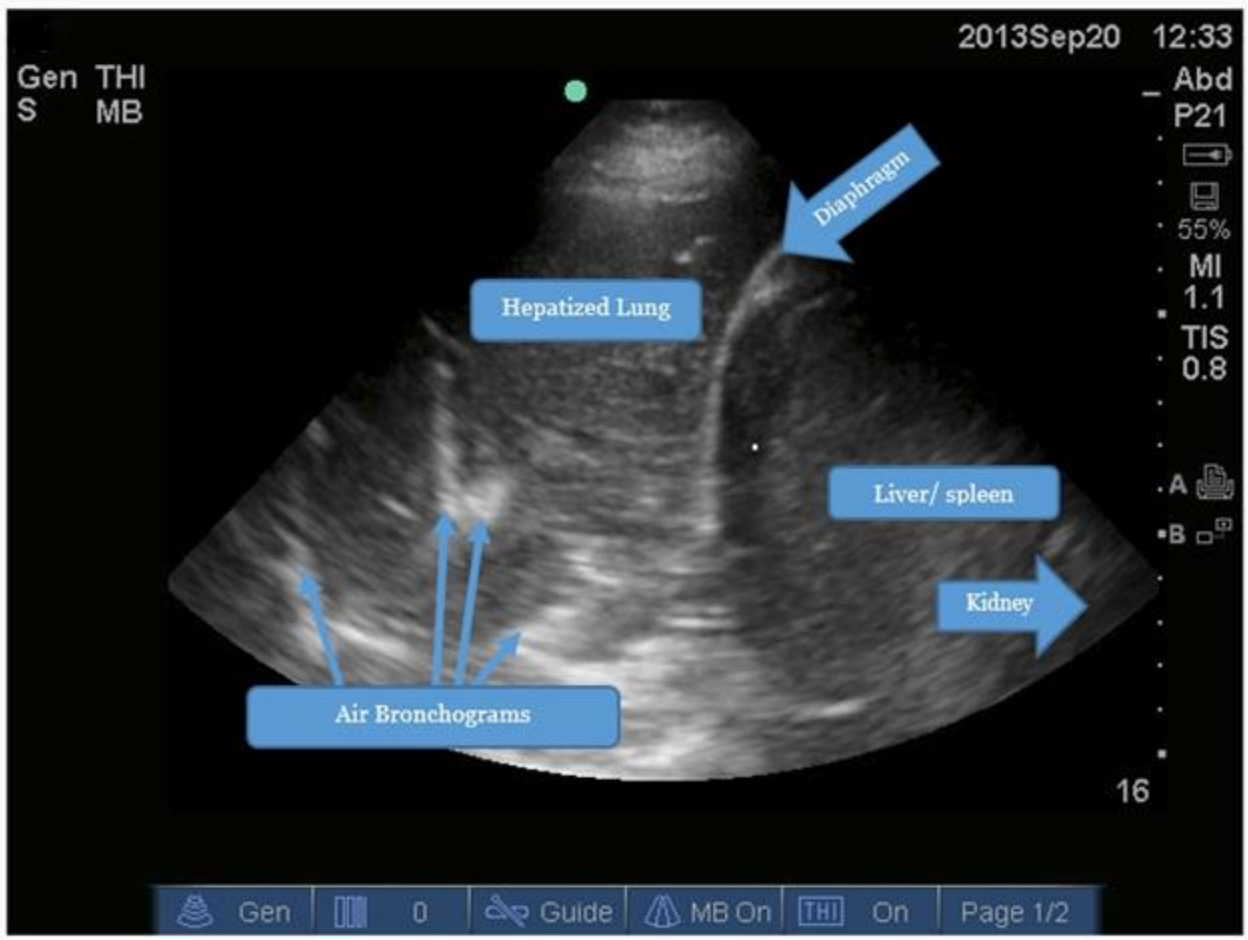

Hepatization Of Lung

- Location: Posterior-Lateral Chest
- Probe: Low Frequency Phased Array
- Probe Marker: Cephalad
- Machine setting: Abdomen or Lung
- Screen Marker: Operator-Left
- Screen Depth: 14-18 cm

Dynamic Air Bronchograms

This finding is sometimes seen in association with the hepatized lung. The white linear markings seen within the consolidated lung parenchyma, called air bronchograms, may show a speckle or bright movement within them, correlating to the patient's respirations. These dynamic air

bronchograms represent pus and air moving within very distal small bronchi in and medial to lateral as the patient breaths exhales and inhales. A hepatized lung with dynamic air bronchograms is usually indicative of a very severe dense lobar PNA.

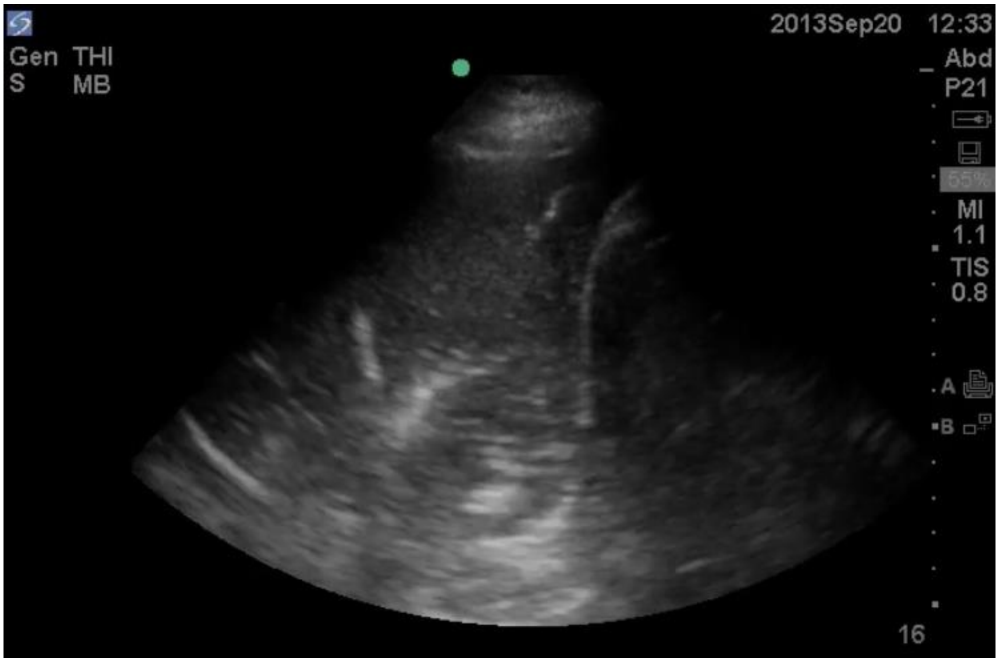

Dynamic Air Bronchogram

- Location: Posterior-Lateral Chest
- Probe: Low Frequency Phased Array
- Probe Marker: Cephalad
- Machine Setting: Abdomen or Lung
- Screen Marker: Operator-Left
- Screen Depth: 14-18 cm

Chest- Evaluation for Pleural Effusion

Lijo Illiparambil Chacko, MD & Sahar Ahmad, MD

Introduction

A common use for bedside ultrasound is evaluation of a pleural effusion. While Chest X-ray remains the initial imaging modality to identify whether a pleural effusion may exist. Proper evaluation of a pleural effusion can be done easily and accurately at the bedside by US. This will not only allow rapid evaluation and characterization of a pleural effusion but also allows for visual guidance if the pleural fluid needs be removed and analyzed.

Imaging technique:

Standard technique includes positioning of the transducer in a longitudinal orientation over the chest wall in an intercostal space with the probe marker held cranially by convention and gain and depth settings are adjusted to focus attention at the contents of the pleural space as visualized between the rib shadows (fig. 2).[17] The initial depth is set at 16-20 cm [3] and can be adjusted accordingly to the findings. M- mode is applied in assessing motion of the atelectatic lung within the pleural effusion.

This can between done with two methods with the patient sitting up or lying flat. The decision about which approach may largely depend on the patient. The acutely dyspnic patient (example during RRT) may not toelrate upright positioning. In these patient having the patient supine with arms raised above their head can allow for accurate assessement for plueral effusions (fig 1).

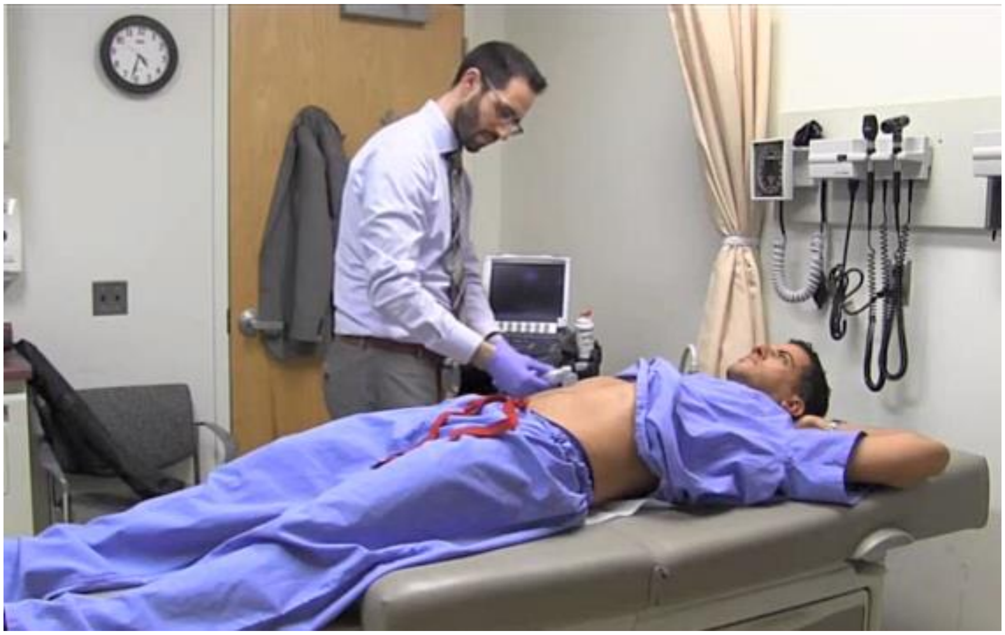

With permission: Dr. Craig Fryman (left) and Andy Ahmed are photographed

In the more stable pateint sitting upright can also allow interrogation of further posterior pleural effusions (figure 2). While the technique remains relatively unchanged it does allow easier access for procedures such as a thoracentesis if warranted.

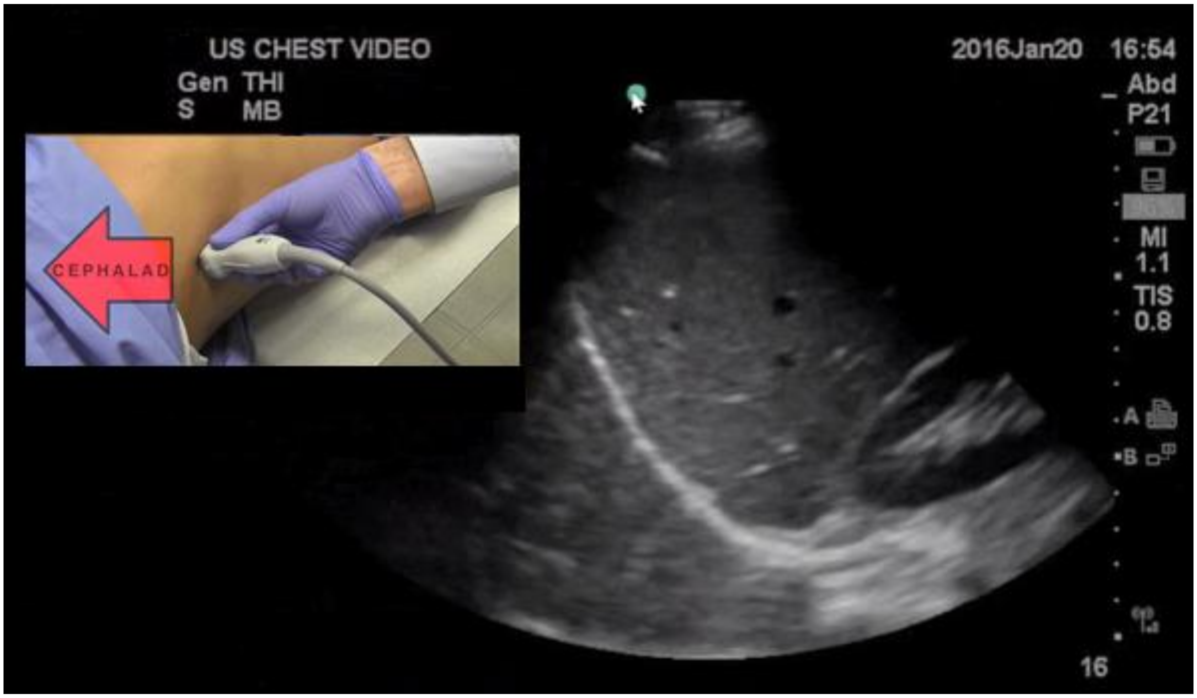

Figure 1b: Nota bene: it is very important to identify organs prior to moving forward. In certain cases without the kidney it can be difficult to assess if one is looking at liver or consolidated lung as such found in pneumonia. Additionally without a proper depth one may not see the kidney as it is a retroperitoneal organ. Increase or decrease depth until able to orient oneself.

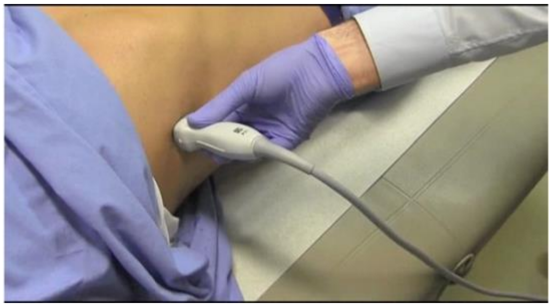

US can be performed at bedside with a handheld or portable device smaller in size than those used by technicians. Two machine modes, Brightness (B) mode and Motion (M) mode, are the most commonly utilized modes for evaluation of pleural disease. A low frequency transducer probe of 2-5 MHz (typically 3.5MHz) frequency is commonly recommended. While a curvilinear shaped probe can be utilized, we recommend a phased array, or sector shaped probe, due to a smaller footprint which allows for enhanced visualization between rib spaces (fig. 1).

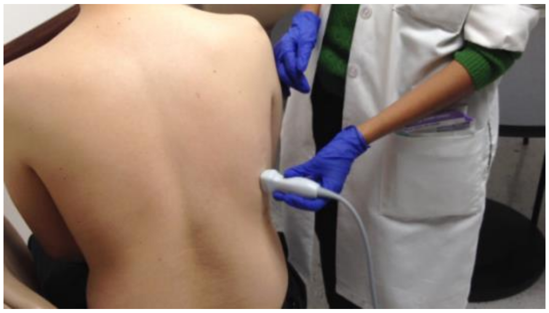

A normal lung US, one without an effusion present, would be demonstrated by the curtain sign. This occurs when an aerated lung, of which is differentiated from the diaphragm on full expiration with full visualization of the sub- diaphragmatic organs, obscures the diaphragm and sub- diaphragmatic organs on full inspiration.[3] (fig. 3)This is an artifact of dynamic movement of the aerated lungs and shows that there is no pleural fluid present.

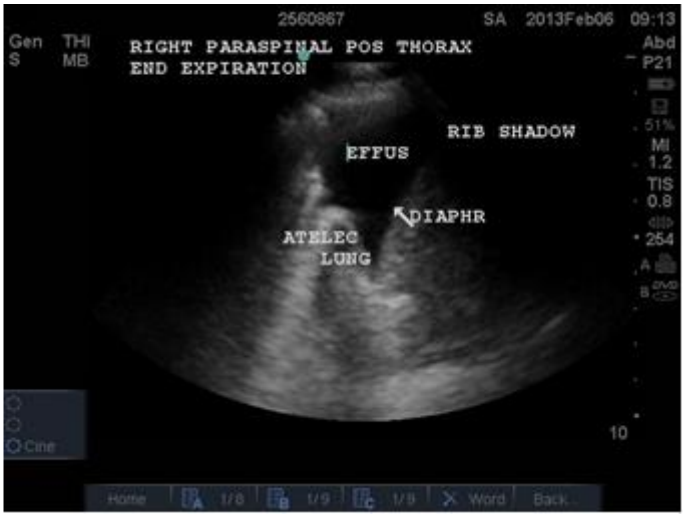

If a pleural effusion is present, there is a term for the atelectatic lung called jelly fish sign or flapping lung sign.[4,5] (fig 4). This shows that there is some movement of the lung within the pleural fluid and will be more pronounced in transudative and newer effusions. In small effusions, atelectasis may not be present and this sign would not be visible.[3] Further discussion of differentiating the etiology of pleural effusions based on US will be discussed below.

Pleural Effusion Size

Ultrasound can distinguish between small and large (clinically irrelevant and clinically significant) effusions:

US Features	Example	Notes
Inflated lung is visible to chest wall during inspiration	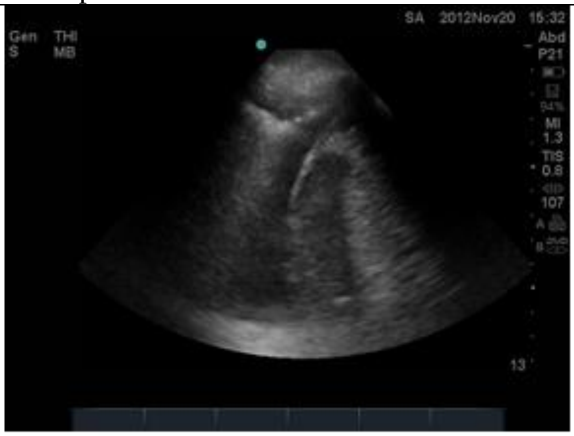	Trivial Pleural effusion- not etiology of symptoms

Small anechoic area separating lung from diaphragm, however lung still expanded to chest wall during inspiration	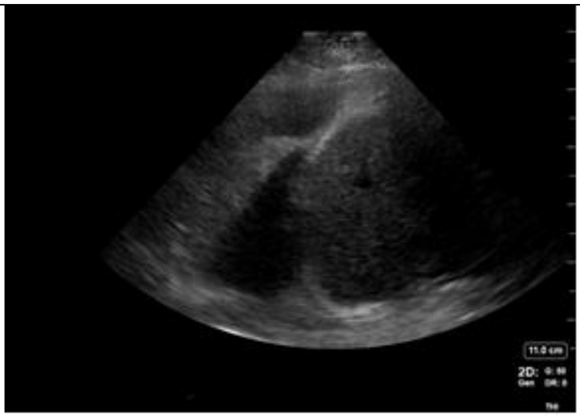	Small pleural effusion- unlikely symptomatic
Larger anechoic area, with atlectatic lung visible. Diaphragm retains dome shape.	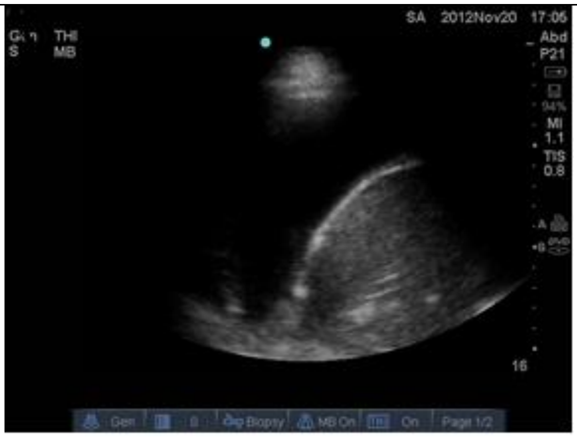	Moderate to large- symptomatic
Atlectatic lung no longer visible; Diaphragm is flattened or everted.	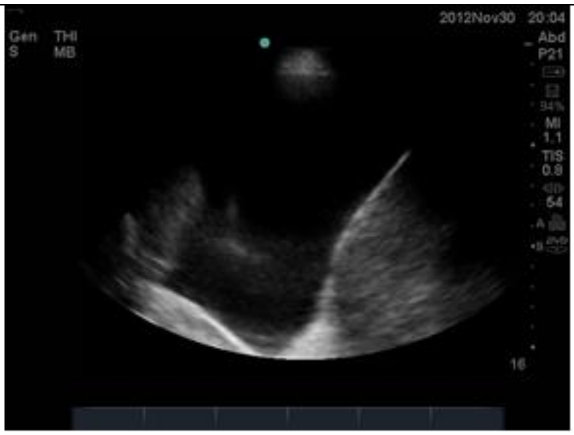	Massive pleural effusion- very symptomatic; avoid laying patient flat; consider chest tube drainage

Pleural effusion etiology

Ultrasound can differentiate between simple and complex effusion, and predict pleural effusion etiology.

US Features	Example	Suspected diagnosis & Notes
Simple anechoic fluid	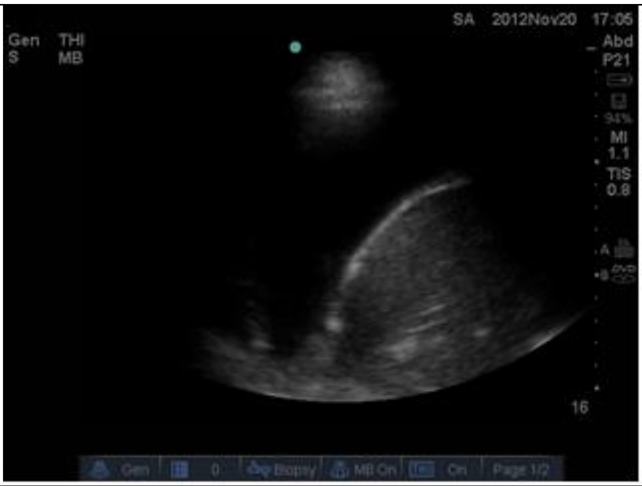	Likely a simple effusion but may still be exudative. If simple, depending on clinical context nan be seen in CHF, Hepatic Hydrothorax, Pancreatitis, other volume overload, hypoalbuminemia .
Fibrin, loculations	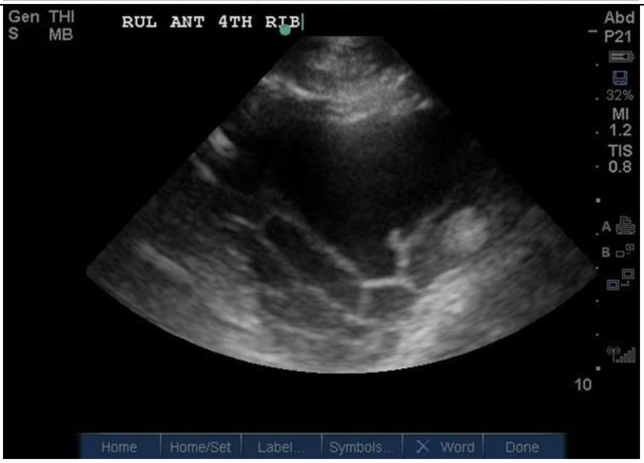	Empyema is suspected
Hematocrit sign (layering)<br>With plankton sign (swirling)	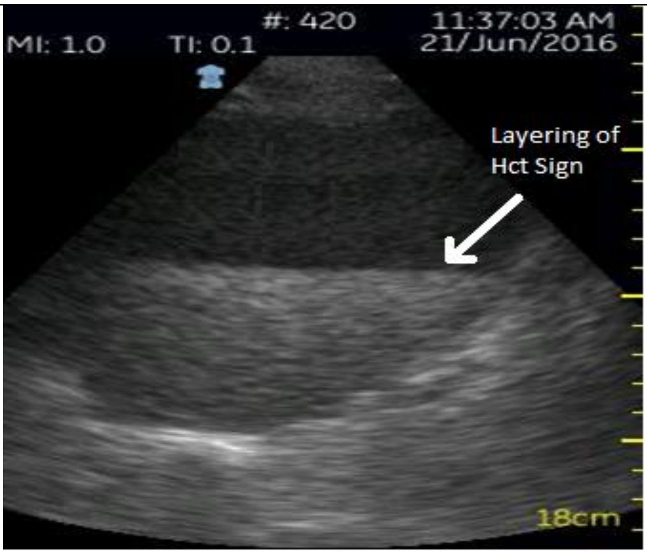	Hemothorax is suspected

Pleural based mass<br>Fibrin, loculations	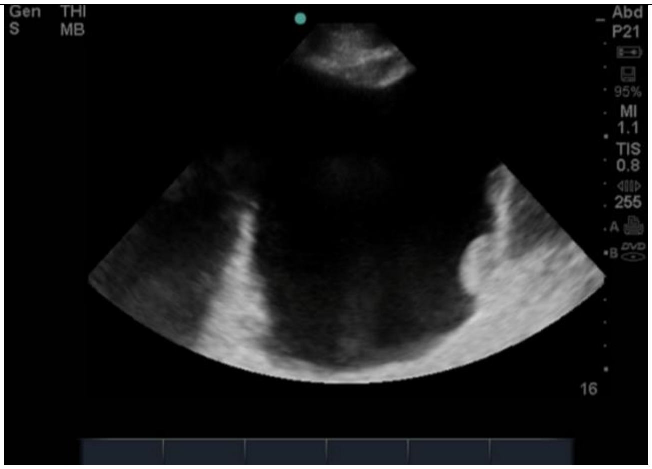	Malignancy is expected

Pearls & Pitfalls:

You must identify kidney then move/look cephalad to identify the diaphragm. Kidney is your vantage point, so that you can avoid mistaking ascites for pleural fluid.

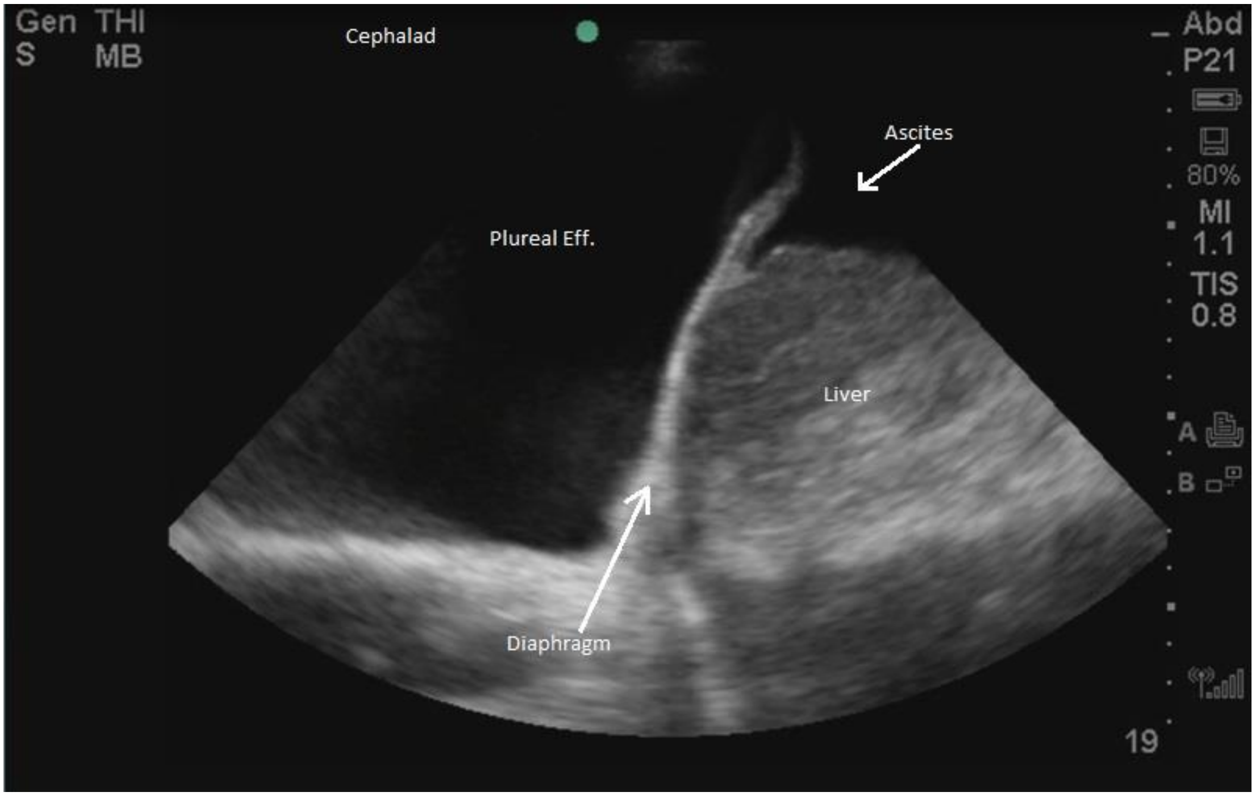

Further Reading:

Doelken P, Eisen LA., Ahmad S. Ultrasound Evaluation of the Pleura. In: Levitov A, Mayo, P., Slonim, A., ed. Critical Care Ultrasonography. 2nd ed. New York, NY: McGraw Hill; 2014:197-206.

Lichtenstein DA. Lung ultrasound in the critically ill. Annals of intensive care. Jan 09 2014;4(1):1.

Rambhia SH, D'Agostino CA, Noor A, Villani R, Naidich JJ, Pellerito JS. Thoracic Ultrasound: Technique, Applications, and Interpretation. Current problems in diagnostic radiology. Dec 15 2016.

Bouhemad B, Zhang M, Lu Q, Rouby JJ. Clinical review: Bedside lung ultrasound in critical care practice. Critical care (London, England). 2007;11(1):205.

Chest- Evaluation of Pneumothorax

Craig Fryman, MD and Sahar Ahmad, MD

Introduction

Pneumothorax (PTX) is a commonly encountered and potentially life-threatening condition. Although CT is the gold standard for diagnosis, it is costly, can delay diagnosis, and requires large doses of radiation. Chest radiography (CR) has been conventionally used to rapidly detect PTX despite low sensitivity compared to CT [1]. Moreover, it is particularly challenging to detect PTX with CR in critically ill patients who are often imaged while supine.

Ultrasound (US) offers a noninvasive, non-ionizing, cost-effective, and rapid means of detecting PTX with similar specificity but far superior sensitivity to that of CR. Two meta-analyses evaluating the sensitivities and specificities of US in detection of PTX found pooled sensitivities and specificities ranging from 0.88 to 0.91 and 0.99 to 0.98, respectively. This is in contrast to pooled sensitivities and specificities of CR ranging from 0.50 to 0.52 and 0.99 to 1.00, respectively [2,3].

Ultrasound

Examine the patient in the supine position using either a high-frequency linear or a low-frequency phased array probe in B-mode. The probe marker should be pointed cephalad and positioned longitudinally over the bilateral chest wall anteriorly between two adjacent ribs. Visualize the rib shadow on either side of the screen as dark vertical shadows. The goal is to achieve maximum extension of the visible pleural line, a bright hyperechoic horizontal linear structure located approximately one centimeter deep to the upper edge of the rib.

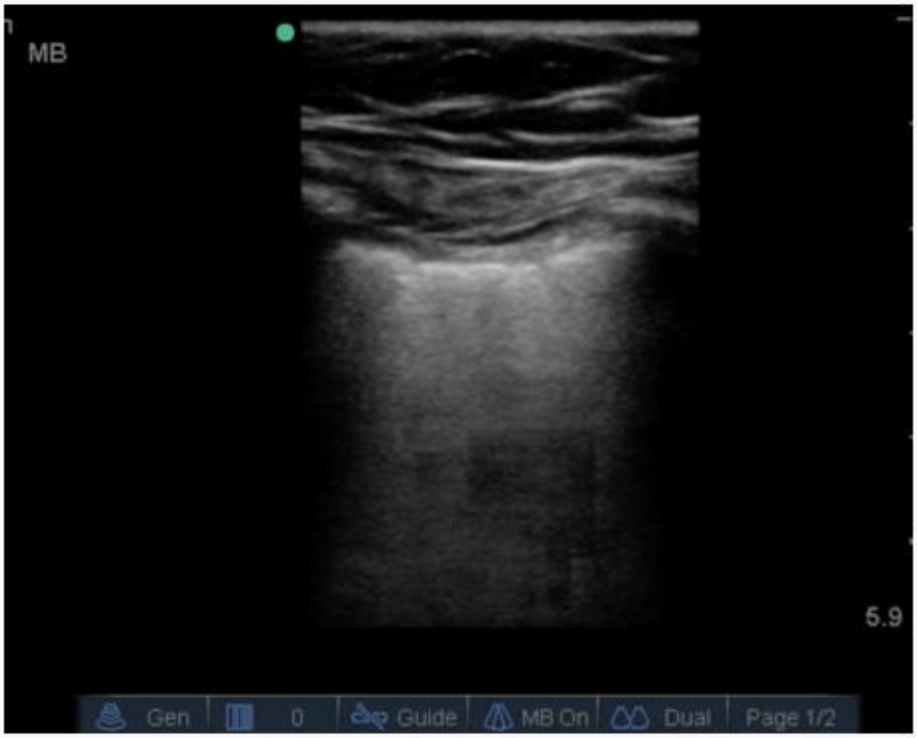

Lung Exam with Linear Array Probe

In a normal lung, the parietal and visceral pleura are separated by a virtual space. The movement or sliding of these two layers against one another, referred to as lung sliding, may be observed with US. During respiration, this manifests as a shimmering of the bright hyperechoic horizontal

structure that represents the pleural line. In the setting of PTX, in which air separates the parietal from visceral pleura, US only detects the parietal pleural line. This is due to the inability of US waves to propagate through the layer air present beneath the parietal pleura. Consequently, lung sliding that is normally observed during a respiratory cycle is absent.

Alternatively, one may use M-mode to diagnosis PTX, particularly in cases when lung sliding is difficult to identify on US. Obtain the same image as described above and run M-mode through the center of the screen, plotting movement of the pleural line against time. Sliding of the parietal and visceral pleura during a respiratory cycle produces a characteristic image consisting of grainy horizontal lines known as "sea-shore sign" (bottom left). This sign is notably absent in the presence of PTX as one can only visualize the parietal pleura. The consequent M-mode image projected in the case of PTX is characterized by flat horizontal lines known as "bar-code sign," signaling the absence of movement of parietal against visceral pleura (bottom right).

View	Identify	Transducer Probe Marker	Machine Setting	Location
Lung	Rib shadows<br>Pleural line<br>Lung sliding	Low frequency (phased aray) →<br>OR<br>High frequency →<br>(linear array)<br><br>Probe marker cephalad (both transducers)	Abdominal (phased)<br>OR<br>Vascular (linear)<br><br>Screen marker operator left (both settings)	Bilateral anterior chest wall between 2 adjacent ribs (check 4 locations - 2 on either side of chest wall

Pearls and Pitfalls

- Examine at least four non-gravity dependent regions of the chest wall – two locations on either side
- If lung sliding is not apparent with B-mode, use M-mode by directing the beam perpendicular to the pleural line
- The presence of B lines effectively rules out PTX
- The presence of lung sliding or "sea-shore sign" rules out PTX in the location on which the probe is placed – be aware that the patient may still have a PTX elsewhere

<u>References</u>

1. Azad A, Juma SA, Bhatti JA, Dankoff J. Validity of ultrasonography to diagnosing pneumothorax: a critical appraisal of two meta-analyses. CJEM 2015;17:199-201.
2. Alrajhi K, Woo MY, Vaillancourt C. Test characteristics of ultrasonography for the detection of pneumothorax: a systematic review and meta-analysis. Chest 2012;141:703-8.
3. Ding W, Shen Y, Yang J, He X, Zhang M. Diagnosis of pneumothorax by radiography and ultrasonography: a meta-analysis. Chest 2011;140:859-66.

Chest- Evaluation of Pulmonary Edema

Peter Hong, MD and Sahar Ahmad, MD

Introduction

Bedside ultrasound (US) allows the physician to quickly evaluate for pulmonary edema as a cause of respiratory distress in real-time without having to wait for other less specific imaging modalities such as chest x-ray. Rapidly recognizing findings suggestive of pulmonary edema can help guide diagnosis and management.

Terminology

Sonographic artifacts are caused by air-tissue interfaces arising from the pleural line. An A-line is a bright and hyperechoic horizontal line parallel to the chest wall. It is a reverberation of the pleural line. Several parallel A-lines can be visualized in regular intervals given an adequate depth setting. The A-line dominant pattern, in the presence of lung sliding, suggests a "normal" and "dry" lung parenchyma. It has been correlated with low pulmonary capillary wedge pressure, which further supports the evidence for low likelihood of pulmonary edema [1]. A B-line is a comet tail artifact that is hyperechoic and vertical, extending from the inferior aspect of the pleural line to lower edge of the screen without fading [2]. There are seven criteria that must be met to qualify as a B line [3]:

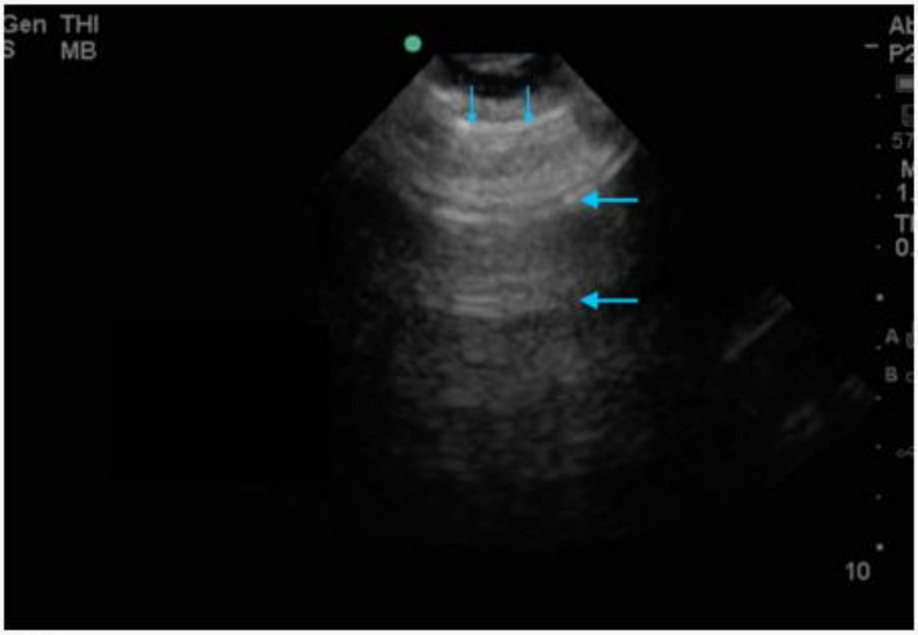

A-Lines

1. Comet-tail artifact
2. Arises from the pleural line
3. Well defined and laser-like
4. It is hyperechoic
5. Long, spreading out without fading to the edge of the screen
6. Erases, or obliterates, A-line

7. Moves with lung sliding

B-lines fan out, move synchronously with the lung during respiration, and obliterate visualization of A-lines. They are due to thickened interlobular septa and extravascular lung fluid. The presence of multiple and diffuse comet tail B-lines suggests pulmonary edema in the appropriate clinical context. Without context, B-lines are non-specific. It is important to note that single vertical lines that do not meet the above criteria are artifacts that do not have any clinical value.

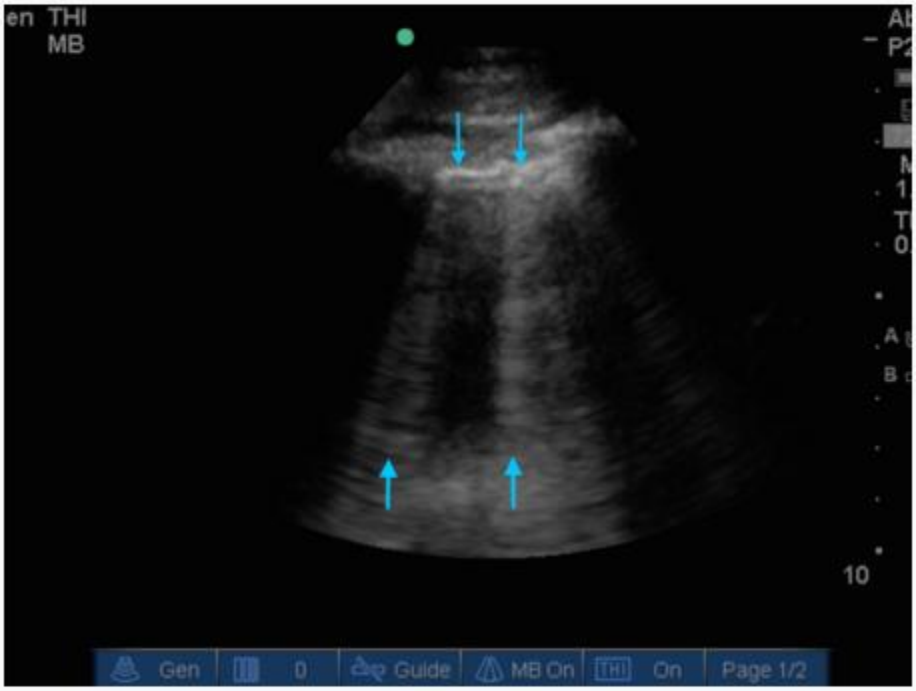

B-Lines

Ultrasound

Use a low frequency transducer (3.5 to 5.0 MHz phased or curvilinear array) in abdomen setting with the marker pointed cephalad and positioned longitudinally over the bilateral chest wall between two adjacent ribs in the area of least lung mass (upper anterior lung field in the supine patient). Visualize the rib shadow on either side of the screen as dark vertical shadows. The goal is to achieve maximum extension of the visible pleural line, a bright hyperechoic horizontal linear structure located approximately one centimeter deep to the upper edge of rib.

View	Identify	Transducer Probe Marker	Machine Setting	Location
Lung	Rib shadows<br>Pleural line<br>A-lines<br>B-lines	Low frequency (phased array)<br><br>Probe marker cephalad	Abdominal<br><br>Operator left	Bilateral anterior chest wall between 2 adjacent ribs

Pearls and Pitfalls

- A single B-line in isolation is unlikely to have any clinical value.
- B-line should be differentiated from E-line, which is often seen in emphysema and described as a comet tail artifact that extends from an area of subcutaneous emphysema that arises from a layer superficial to the pleural line.
- A B-line is present only when it meets the full criteria as listed above

- Presence of B-lines during cardiac arrest is helpful in that it effectively rules out pneumothorax at the location of the transducer. B-lines originate from the inferior aspect of the visceral pleura and would not be visualized if a pneumothorax were present.

<u>*References*</u>

1. Lichtenstein D, Meziere GA, Lagouyete J-F, et al. A-Lines and B-Lines. Lung ultrasound as a bedside tool for predicting pulmonary artery occlusion pressure in the critically ill. CHEST 2009; 136(4):1014-20
2. Lichtenstein D, Meziere G. A lung ultrasound sign allowing bedside distinction between pulmonary edema and COPD: the comet-tail artifact. Intensive Care Med. 1998; 24:1331–1334.
3. Lichtenstein D: Classification of artifacts. In Whole Body Ultrasonography in the Critically Ill. Edited by. Heidelberg, Berlin, New York: Springer-Verlag; 2010:185–188.

Diaphragm- Evaluation of Diaphragm Function

Craig Fryman, MD and Sahar Ahmad, MD

Introduction

Ultrasonographic evaluation of the diaphragm is a relatively new modality and facilitates a means of assessing its structure and function under a variety of circumstances. Moreover, its low cost as well as its non-ionizing and non-invasive nature make it a preferred tool for many for studying the diaphragm in a dynamic setting over other widely used imaging modalities, such as fluoroscopy and electromyography.

The diaphragm can be visualized globally as it demarcates thorax from abdomen. In such a view, its excursion direction (normal vs. paradoxical) and its distance during the respiratory cycle can be visually assessed. The diaphragm can be visualized more discretely as well to assess the thickness of the muscle itself (on the order of micrometers to millimeters) and the changes in this thickness during the respiratory cycle.

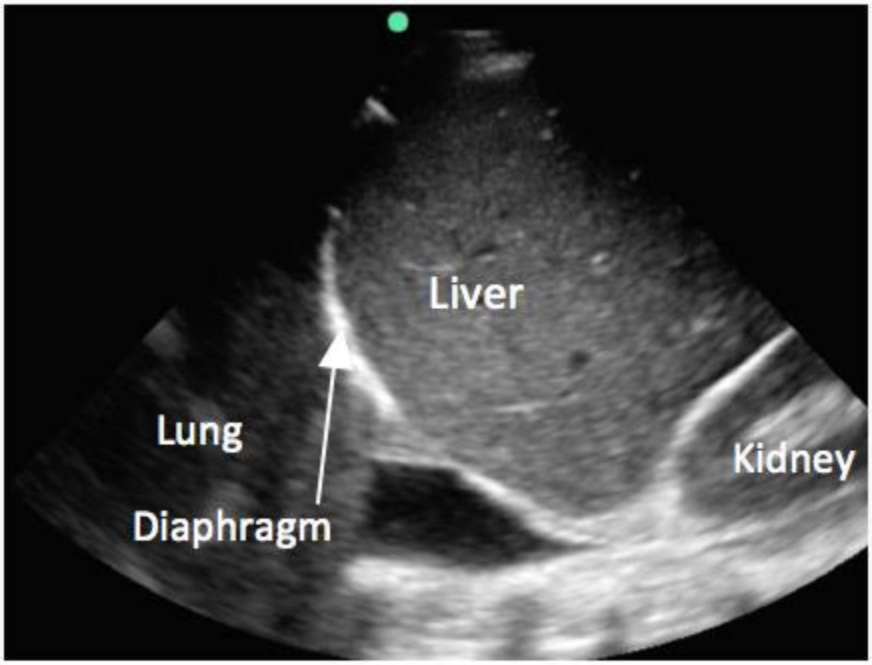

Global view of diaphragm and surrounding structures (low frequency, phased array probe)

Measuring diaphragm thickness (Tdi) has proven accurate with the use of ultrasound (US) [1] and has been employed in several studies evaluating healthy volunteers [2], patients with suspected diaphragm dysfunction [3,4], and those undergoing either non-invasive positive pressure ventilation (NIPPV) [5] or mechanical ventilation (MV) [6].

Assessment of Tdi and specifically its relative changes during the respiratory cycle, represented by the thickening fraction (TF), can be used as a measure to assess diaphragm function in some patients [7]. It has also been employed as a predictor of successful extubation in those who are mechanically ventilated [8,9].

Ultrasound

Examine the patient in the supine position using a high-frequency linear array transducer probe. In B-mode, using the liver as an acoustic window, visualize the right hemidiaphragm at the zone of apposition (ZOA) at a right anterior to mid-axillary scan plan between the 8th and 10th intercostal spaces. It will appear as a group of three horizontal stripes. These stripes, from near-screen to far-screen, represent: the parietal layer, continuous from the parietal layer of the pleura; the muscular layer; and the peritoneal layer, continuous with the abdominal peritoneum.

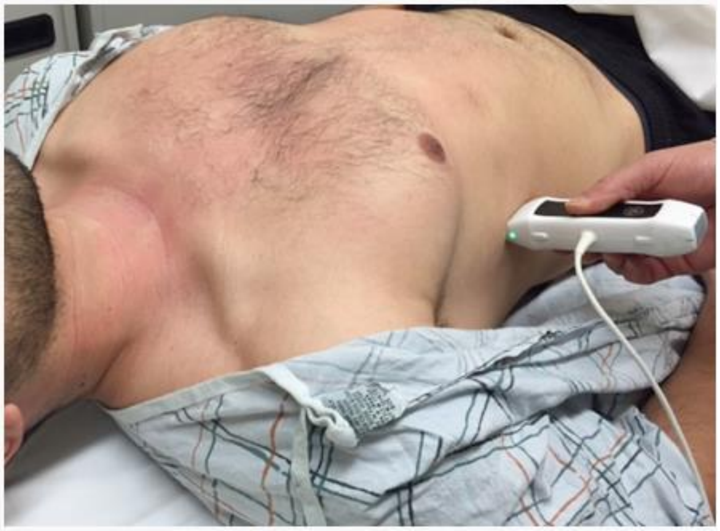

Proper placement of probe

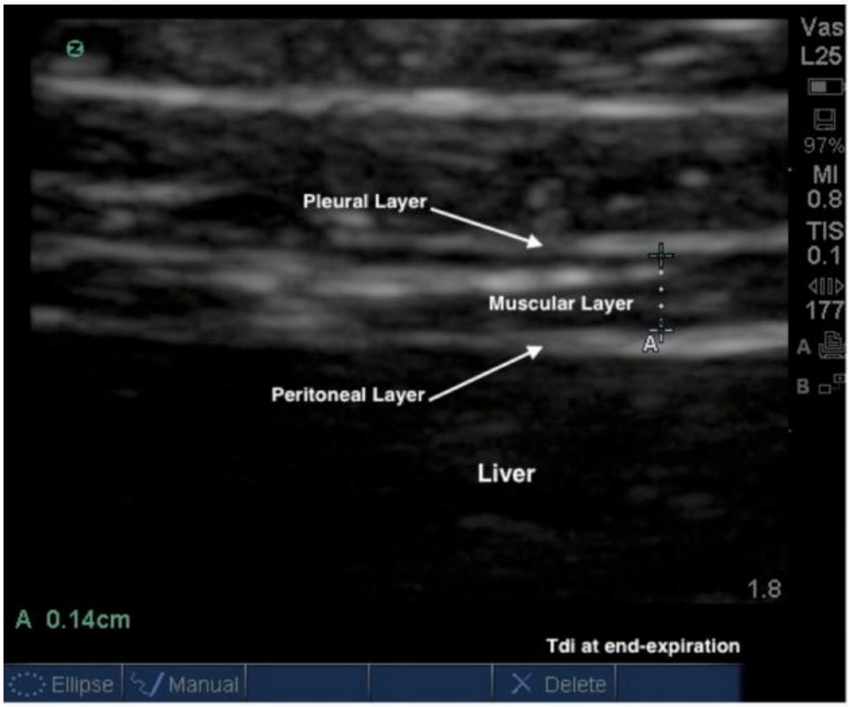

Using zoom functionality for optimal visualization, Tdi is measured on still frames from the inner edge of the peritoneal and parietal layers flanking the muscular diaphragm [1].

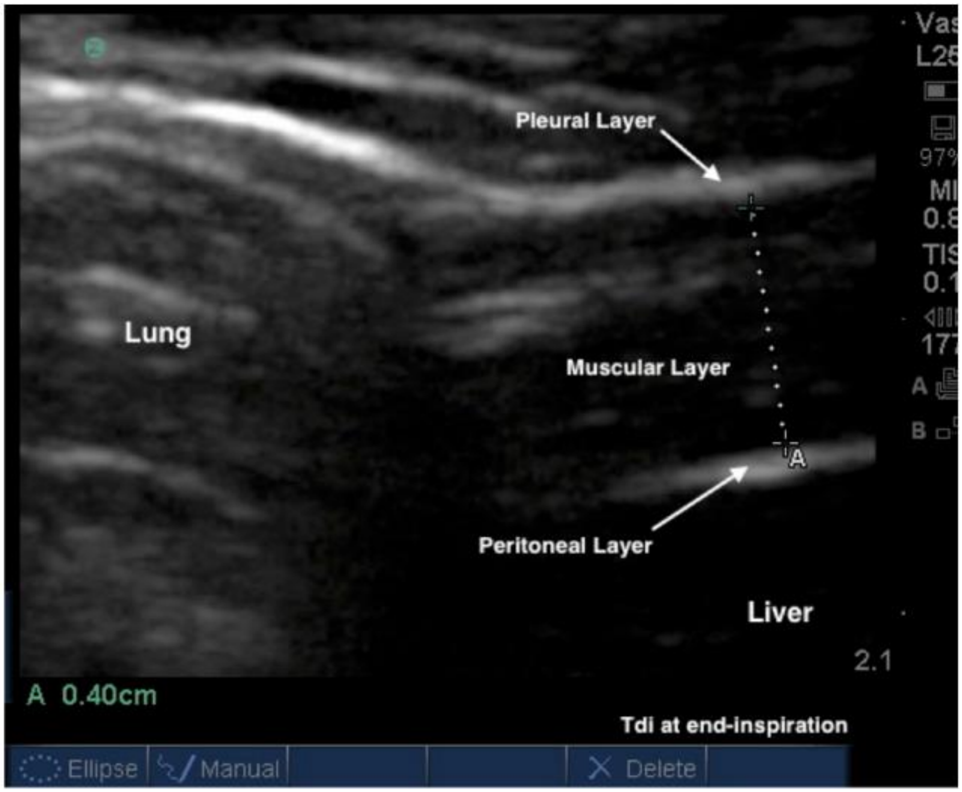

The TF, a relative measure of diaphragm function, can be calculated for each delivered tidal volume (TV) as follows:

$$\mathbf{TF} = \frac{\mathbf{Tdi}\mathit{max} - \mathbf{Tdi}\mathit{min}}{\mathbf{Tdi}\mathit{min}}$$

Image Interpretation

In normal subjects, as reported by Boon et al. (2013), Tdi in the ZOA at resting end-expiration is at least 0.15 cm and thickens by at least 20% at maximal inspiration [7]. DiNino et al. (2014) found that a TF > 30 during a pressure support (PS) or spontaneous breathing trial (SBT) was associated with extubation success, comparable to RSBI > 105 [8].

Another useful clinical finding to assess diaphragmatic breathing is the diaphragmatic excursion test. Generally done as a clinical exam with percussion, with US one is able to see the diaphragm move. This is measurable in M-mode and can provide a good assessment of diaphragm movement especially in cases where diaphragmatic paralysis is a concern. The patient remains in the same position as prior however instead of the high frequency linear array probe, one should select the low frequency phased array probe. After visualizing the diaphragm, place the ultrasound in M mode, and measure the movement of the diaphragm. One should get a wave form similar to the one below. Measuring the low and high points of the wave indicate exhalation and inhalation phase of the diaphragm respectively. Taking the difference gives you the diaphragmatic excursion. A good benchmark for normal breathing would be between 3-5 cm.

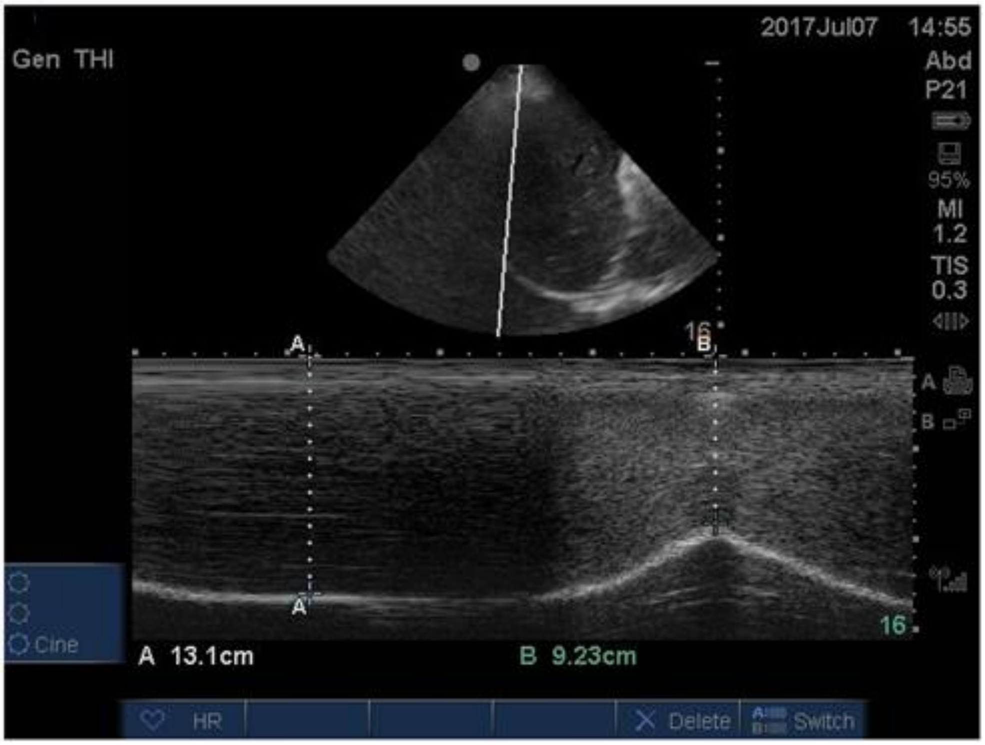

Pearls and Pitfalls

- Tdi can be accurately assessed with US
- The TF may be used to assess diaphragm function
- Using zoom function, measure Tdi from the internal edges of the subtending layers of the diaphragm
- Assessment of left hemidiaphragm is difficult as the spleen does not offer an optimal acoustic window
- Poor patient effort will prevent maximal thickening of the diaphragm [7]
- Variability in positioning of the probe can result in significant variability in measurements [7]
- B-mode is a preferred modality over M-mode as it allows for better visualization of diaphragm anatomy and adjacent structures

References

1. Cohn D, Benditt JO, Eveloff S, McCool FD. Diaphragm thickening during inspiration. Journal of applied physiology (Bethesda, Md : 1985) 1997;83:291-6.
2. Ueki J, De Bruin PF, Pride NB. In vivo assessment of diaphragm contraction by ultrasound in normal subjects. Thorax 1995;50:1157-61.

3. Gottesman E, McCool FD. Ultrasound evaluation of the paralyzed diaphragm. American journal of respiratory and critical care medicine 1997;155:1570-4.
4. Summerhill EM, El-Sameed YA, Glidden TJ, McCool FD. Monitoring recovery from diaphragm paralysis with ultrasound. Chest 2008;133:737-43.
5. Vivier E, MekontsoDessap A, Dimassi S, et al. Diaphragm ultrasonography to estimate the work of breathing during non-invasive ventilation. Intensive care medicine 2012;38:796-803.
6. Grosu HB, Lee YI, Lee J, Eden E, Eikermann M, Rose KM. Diaphragm muscle thinning in patients who are mechanically ventilated. Chest 2012;142:1455-60.
7. Boon AJ, Harper CJ, Ghahfarokhi LS, Strommen JA, Watson JC, Sorenson EJ. Two-dimensional ultrasound imaging of the diaphragm: quantitative values in normal subjects. Muscle & nerve 2013;47:884-9.
8. DiNino E, Gartman EJ, Sethi JM, McCool FD. Diaphragm ultrasound as a predictor of successful extubation from mechanical ventilation. Thorax 2014;69:423-7.
9. Ferrari G, De Filippi G, Elia F, Panero F, Volpicelli G, Apra F. Diaphragm ultrasound as a new index of discontinuation from mechanical ventilation. Critical ultrasound journal 2014;6:8.

Lower Extremity- Ultrasound to Rule Out DVT

Nick Pakzad, MD and Sahar Ahmad, MD

Introduction

Venous thromboembolisms (VTEs) can cause significant morbidity and mortality. Over 90% of acute pulmonary embolism (PE) cases are consequence of emboli originating from the proximal veins of the lower extremities. It is therefore essential to quickly and safely diagnose lower extremity DVT [1].

There are several invasive and noninvasive testing modalities available to work up DVT, including impedance plethysmography, D-dimer, magnetic resonance venography (MRV), computed tomography (CT), and contrast venography. Venography is the gold standard, however, due to patient discomfort, contrast load, as well as myriad contraindications and technical factors, venography is not recommended as the first-line diagnostic test [2]. Alternatively, compression ultrasonography (US) has become the primary noninvasive approach of choice given its point of care availability and high efficiency.

By using a succinct 5-point compression protocol, examinations can be rapidly performed by trained personnel at the bedside with high sensitivity and specificity for proximal vein thrombosis [3]. Moreover, the amount of training required to become proficient in bedside US evaluation for DVT diagnosis is reasonable [4,5]. We describe a focused US protocol for this purpose.

Patient Positioning

Position the patient supine or semi-recumbent with the lower extremity rotated laterally and slightly bent at the knee. This orientation creates venous distention, allowing for optimal viewing windows. Begin the examination on the right leg, placing the transducer at the patient's inguinal crease.

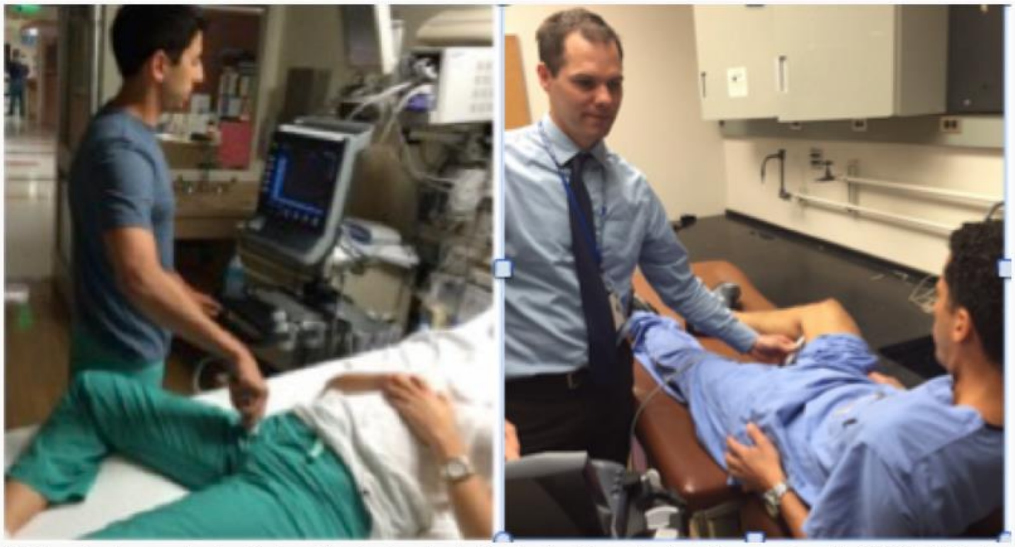

Initial probe placement and patient positioning in a standardized patient for evaluation of lower extremity DVT (Left). Probe placement and patient positioning for evaluation of popliteal vein DVT in a standardized patient (Right).

With permission: Dr. Nick Pakzad (right), Dr. Bjorn Flora (center) and Andy Ahmed (right) are photographed.

Ultrasound

In vascular mode using a high frequency transducer probe, visualize the superficial structures where clinically significant DVTs commonly form. The transducer marker should be operator left at all times. All visualizations and compressions are performed in the transverse orientation. With the transducer at a 90-degree angle, compress the vein until the walls appose each other and the lumen is completely obliterated. This signifies the absence of a DVT. If the lumen does not obliterate with compression, consider that a DVT is present. The majority of DVTs will be located on the posterior aspect of the vein.

Ultrasound Protocol

Two anechoic structures will be visualized: the common femoral vein (CFV) medial to the common femoral artery (CFA) (image 1). This is the initial point of compression. Next, locate

View	Identify	Transducer Probe Marker
Lower extremity vasculature	1. CFV<br>2. CFV at junction with great saphenous vein<br>3. CFV distal to division of CFA to SFA and DFA<br>4. FV and DFV<br>5. Popliteal artery and vein	High frequency (linear array)<br><br>Probe marker operator left

the sapheno-femoral junction (SFJ) by sliding the probe distal, which reveals the great saphenous vein arising from the CFV anteriorly and medially (image 2). This is the second point of compression. Move the probe caudal, tracking the previously visualized CFV and CFA down the lower extremity until the CFA begins to branch, forming the superficial femoral artery (SFA) and the deep femoral artery (DFA) (image 3). This is the location of the third compression point. Caudally, the CFV then divides into the femoral vein (FV) and deep femoral vein (DFV) (image 4). This is the fourth compression site. The fifth and final compression location is the popliteal vein, for which we place the probe in the popliteal fossa of the laterally rotated and slightly bent lower extremity, and point the probe towards the anterior aspect of the knee. Two anechoic structures are visualized: the popliteal vein (near-field) and the popliteal artery (far-field) (image 5). This is the final point of compression. The same examination is then performed on the contralateral lower extremity.

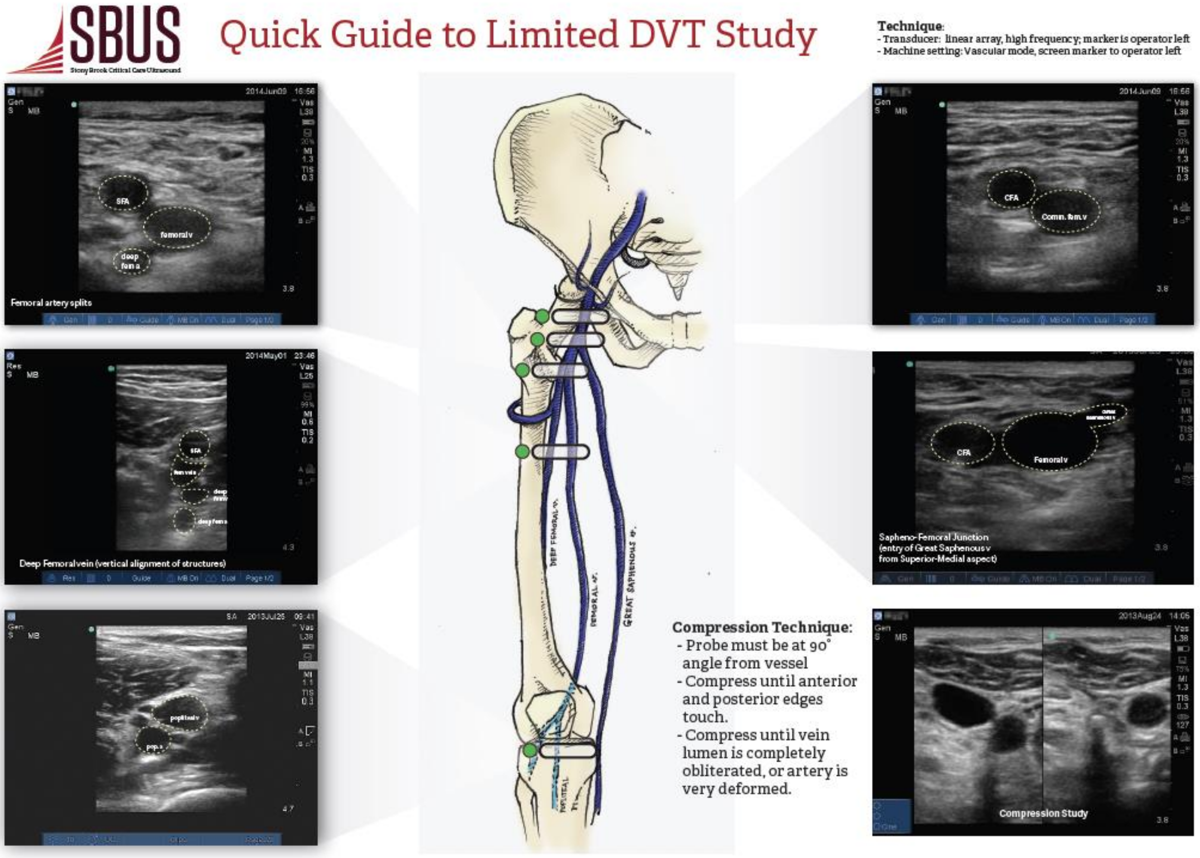

Pearls and Pitfalls

- In cases where the sapheno-femoral junction is high above the inguinal ligament, use angulation technique to look proximally.
- At times DFA takeoff is proximal or at the SFJ, in which case a third compression site should be performed distal to SFJ and proximal to CFV division.
- To visualize the deep vein takeoff and the popliteal vein (both easily compressed structures) keep a very light touch, barely making contact with the skin.
- Popliteal vein examination easier if the leg is rotated laterally and slightly bent at knee.
- Limited results in patients with deformities, pelvic neoplasms, abscesses, or recurrent DVTs.
- If initial study is negative but clinical suspicion is high, a repeat study should be performed in 5 to 7 days.

<u>References</u>

1. Galanaud JP, et al. Comparative Study on Risk Factors and Early Outcome of Symptomatic Distal Versus Proximal Deep Vein Thrombosis: Results from the OPTIMEV Study. ThrombHaemost. 2009; 102(3):493.

2. Heijboer H, et al. Detection of Deep Vein Thrombosis with Impedance Plethysmography and Real-Time Compression Ultrasonography in Hospitalized Patients. Arch Intern Med. 1992; 152(9):1901.
3. Lensing AW, et al. Detection of Deep-Vein Thrombosis by Real-Time B-Mode Ultrasonography. N Engl J Med. 1989; 320(6):342.
4. Kory, PD, et al. Accuracy of Ultrasonography Performed by Critical Care Physician for the Diagnosis of DVT. CHEST. 2011;139:538-542.
5. Crisp, JG, et al. Compression Ultrasonography of the Lower Extremity with Portable Vascular Ultrasonography can Accurately Detect Deep Venous Thrombosis in the Emergency Department. Ann Emerg Med. 2010; 56(6):601-610.

Procedure- Internal Jugular Central Venous Catheter Insertion

Andrew Lyu, MD and Sahar Ahmad, MD

Introduction

Central venous catheters (CVCs) are instrumental in the management of critical ill patients, allowing the delivery of medications such as vasopressors or nutrition (e.g., TPN) and providing vascular access when peripheral venous access is insufficient. Over 5 million CVCs are placed each year, with roughly 8% of hospitalized patients undergoing such a procedure [1].

Adverse events related to placement of CVCs include arterial injury, puncture of nearby structures, and complications such as hemothorax or pneumothorax. The use of ultrasound (US) has reduced such adverse events and has also been useful for pre- and post-procedural monitoring. Compared with anatomic landmark-guided placement or the "cut-down method" whereby an incision is made to directly visualize the vein, peri-procedural 2-D or Doppler US significantly increases operator success in venous catheterization [2]. US reduces the rate of complications, such as carotid puncture, hemothorax, and pneumothorax when compared to the landmark-guided technique [3]. US-guided placement of CVC has therefore become the standard of care as recommended by the US Agency for Healthcare Research and Quality (AHRQ) and UK National Institute of Clinical Excellence [4].

Ultrasound - Protocol Peri-procedure

Please note that this chapter describes only the US components of the CVC procedure. Refer to your other simulation training materials for the entirety of the procedure itself.

Pre-procedure:

Place the patient in Trendelenberg position to engorge the veins of the neck. Rotate the head 15-45 degrees opposite the side of examination/catheterization. Position the US machine such that the screen is in direct line of sight of the operator during the entire procedure.

1. Identify Anatomy

Using a high-frequency linear array probe with the probe marker operator left, examine the mid-neck using the transverse "short" access view (probe perpendicular to course of vessels) to identify the sternocleidomastoid muscle, the internal jugular vein, and the internal carotid artery. Veins and arteries will appear black (anechoic), with veins being more easily compressed and with thinner walls. Arteries nearly always perfectly round whereas veins are typically not so.

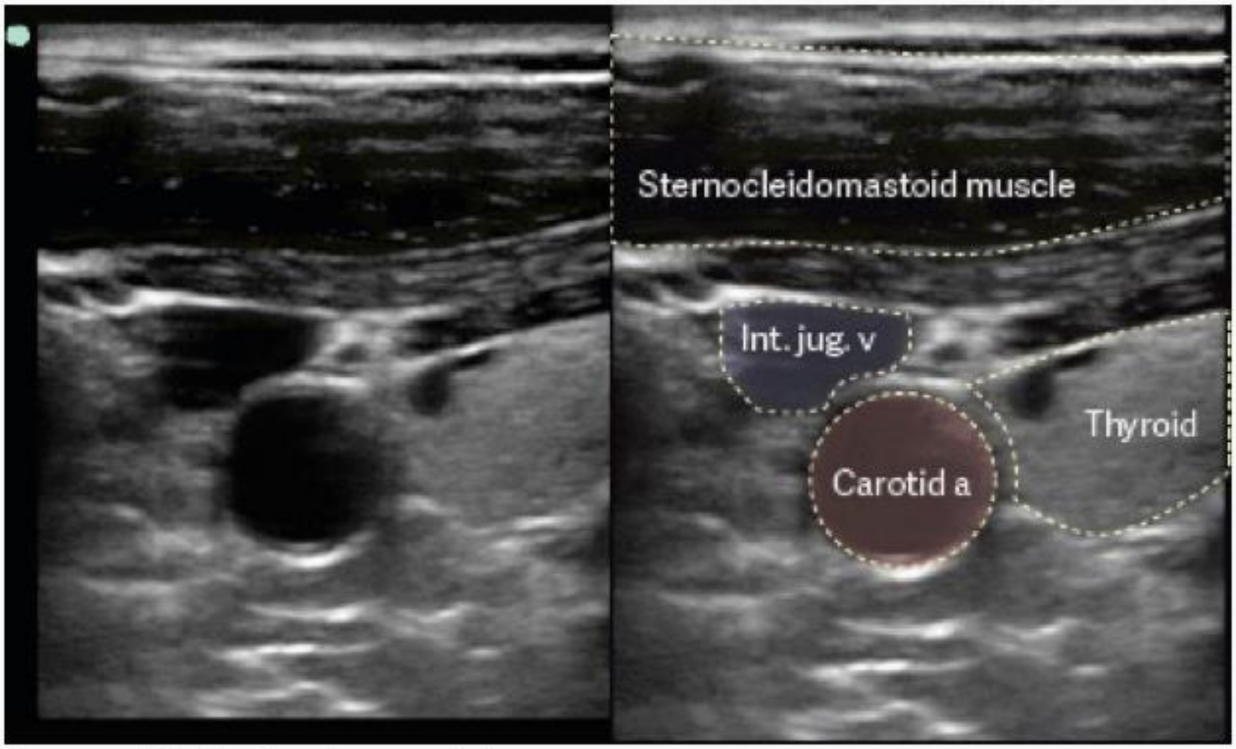

Anatomy of the IJ vein and surrounded structures

2. Rule out vessel thrombosis and stenosis.

Slide the probe along the course of the vessels (up and down from base of neck to clavicle while holding the probe in the same transverse view) to assess for stenosis or thrombosis – if either is present, an alternative site should be considered. Thrombosis is suggested when during a 90 degrees compression of the vessel you ae unable to view the anterior and posterior vessel walls juxtapose, or you see an obvious echo- dense thrombus within the vessel lume.

Stenosis is suggested when the vein's wall is thick, round often echogenic, and there is a sudden change in caliber of the vessel as you track up and down the neck. Note that this can only be picked up by allowing full recoil of the vessel after you compress; and note also that in normals, the Right IJ vein is larger caliber than the left IJ vein, and all vessels decrease slowly in caliber as you move distally cephalad.

3. Assess for lung sliding

Assess the anterior lung fields for lung sliding and pre-procedural pneumothorax (see chapter on pneumothorax). This will help you pick up occult pneumothorax, other pleural/ parenchymal abnormalities (which assist in deciding which side of neck to place the CVC at) as well as serve as the baseline comparator for re- checking this finding post- procedure when you need to rule in or rule out pneumothorax secondary to your procedure.

4. Optimizing Access View

Ensure that the target vessel is in the center of the screen and the appropriate gain is set. In the transverse “short” axis view, create a scan plane which produces a view where the vessels are side by side rather than on top of each other. This usually requires two moves of the probe: few centimeters medially (not up and down the neck, but slide the probe towards the front of the neck) followed by a tilt of the probe such that the scan plane aims outwards (tail of the probe tilts towards the chin). With this simple maneuver, a safe site for access can be achieved, as we have now reduced the possibility of entering the carotid artery in case the posterior wall of the IJ vein is punctured through. If this optimization cannot be achieved, an alternative site should be considered or a repositioning of the patient can be tried.

Intra-procedure Ultrasound

Prep the patient in the usual sterile fashion. Anesthetize the prepared area, and optimize the vessels, again as above such that the IJ is to the side of the artery.

1. Optimize view

Center the IJ vein on your screen, both vertical and lateral centering using sliding and depth, respectively.

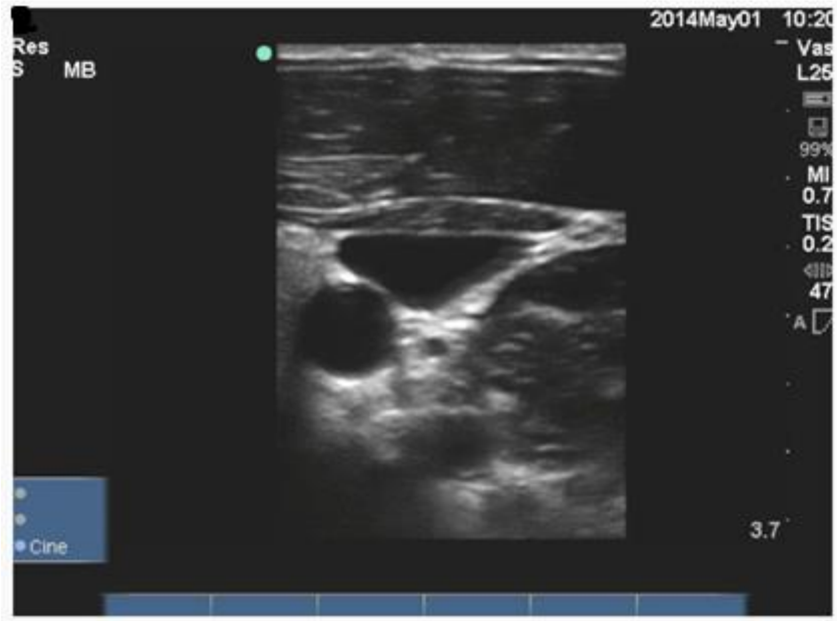

Optimal view - target vessel is centered and positioned at the side of the artery

The needle angle should nearly match that of the probe’s scan plane in steepness as well as in directionality. Insert the needle at a steep angle (about 15 degrees off from the probe itself,

almost parallel to the US beam). You will proceed to enter the skin then begin to hold suction by pulling back on the syringe.

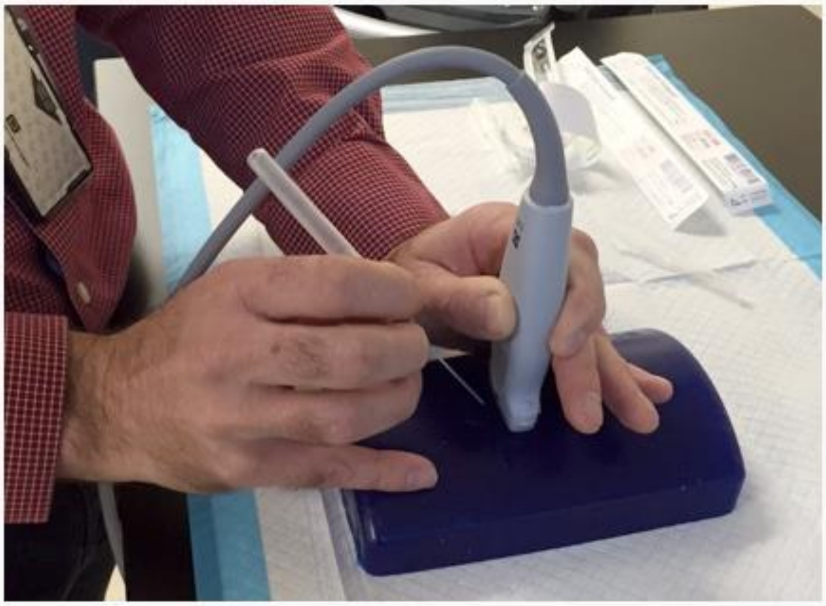
Angle of needle nearly match that of the probe's scan plane

2. Real-time guidance, tracking your needle tip

To be able to visualize your needle tip entering the anterior wall of the vessel, keep the steep angle discussed in the last step, and make very minor angle changes of the probe to track the tip of the needle as it approaches the vessel.

3. Confirmation of guidewire

Once there is flashback in the attached syringe, place the guidewire through the needle. Once the guidewire is inserted, remove the needle as per usual procedure protocol.

Once the needle is out of the neck and the guidewire remains (you are holding the guidewire the entire time) you will confirm placement with US by rotating the probe in the short axis and long axis views. The short axis view is obtained by the same probe -hold you used to insert the needle, scanning right at the skin entry site. You will see the guidewire appear as a bright dot or blur at the anterior wall of the vessel as well as within the vessel itself.

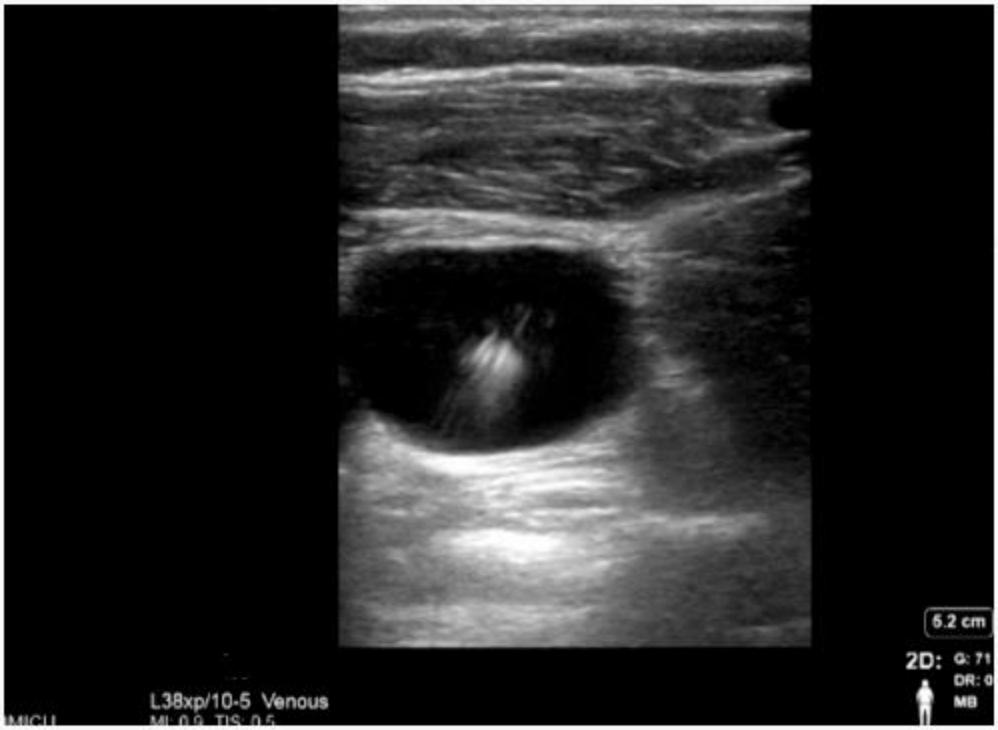

Short axis view of guidewire within the IJ vein

The longitudinal view of the guidewire within the vessel is obtained by a 90 degrees rotation of the probe to obtain the longitudinal “long” axis view of the vessel, never letting go of the guidewire. Ensure that you first identify the target vessel (IJ vein) in short axis, keeping it centered as you perform this rotation, this way ensuring you have rotated open the IJ vein and not the nearby carotid. In a successfully placed guidewire you will see the guidewire sitting along the posterior (far) wall of the vessel, and not exiting out of the vessel.

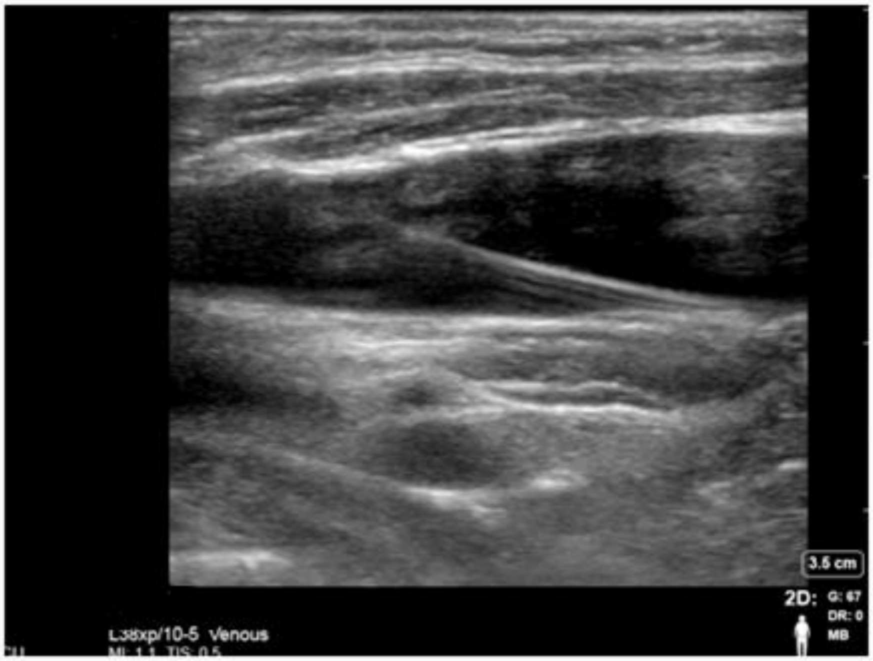

Longitudinal view of guidewire within IJ vein

Once guidewire placement is confirmed, continue placement of the catheter using the standard Seldinger technique.

Post-procedure

1. Rule out post procedure pneumothorax

Before leaving the room after your procedure, re-assess the anterior lung fields, using the same high frequency linear array ("vascular") probe that you used for the procedure, for lung sliding in order to rule out post-procedure pneumothorax.

Present lung sliding prior to the procedure and present lung sliding post procedure is expected. Loss of lung sliding on your post- procedure US check, particularly if the procedure was difficult and if the patient is symptomatic (hypoxic, respiratory distress, hypotension, absent breath sounds, higher peak pressures), should alert you to the development of pneumothorax. In this case, the fellow should be immediately notified to help evaluate for pneumothorax and aid in treatment.

2. Check catheter direction

Confirm catheter direction using the US probe in the transverse "short" axis view by scanning superiorly and inferiorly to the site of placement.

View	Identify	Transducer	Machine Setting	Location
Vascular	SCM<br>IJV<br>ICA<br>Lung sliding	High frequency (linear array)<br>Probe marker cephalad or operator left	Vascular<br>Screen marker operator left	Neck (bilalteral)

SCM - sternocleidomastoid; IJV - internal jugular vein; ICA - internal carotid artery

Pearls and Pitfalls

- Examine the lungs before and after catheterization to check for lung sliding. If you have not checked before the procedure, you will have no comparison for post procedure. Keep in mind that many patients have abnormal reduced or absent lung sliding at baseline due to underlying lung diseases other than pneumothorax.
- Slide the probe medial or lateral to obtain the optimal positioning of vein and artery such that the carotid artery does not underlies the target vein. This will prevent accidental entry through the vein into the artery.
- Squeeze out any air bubbles when placing the sterile US probe cover; air bubbles impede US wave transmission and decrease image quality.
- Apply minimal pressure while using the US probe as too much pressure can collapse the vein making catheterization difficult.

<u>References</u>

1. McGee, D. C., & Gould, M. K. (2003). Preventing complications of central venous catheterization. New England Journal of Medicine, 348(12), 1123-1133.
2. Hind, D., Calvert, N., McWilliams, R., Davidson, A., Paisley, S., Beverley, C., & Thomas, S. (2003). Ultrasonic locating devices for central venous cannulation: meta-analysis. Bmj, 327(7411), 361.
3. Karakitsos, D., Labropoulos, N., De Groot, E., Patrianakos, A. P., Kouraklis, G., Poularas, J., ...&Karabinis, A. (2006). Real-time ultrasound-guided catheterisation of the internal jugular

vein: a prospective comparison with the landmark technique in critical care patients. Critical Care, 10(6), 1.
4. Feller-Kopman, D. (2007). Ultrasound-guided internal jugular access: a proposed standardized approach and implications for training and practice. CHEST Journal, 132(1), 302-309.

Procedures- Ultrasound-Guided Peripheral Intravenous (PIV) Line Insertion

Tejas Shah, MD and Sahar Ahmad, MD

Introduction

Ultrasound-guided PIV insertion is a basic and extremely useful skill set for any physician, particularly when working with the infamous "hard sticks," such as in patients who have edema, are dehydrated, or have difficult body habitus. Familiarity with this technique will prevent delays in patient care.

Preparation

Materials

- Ultrasound (US)
- Sterile US gel
- Tourniquet
- Alcohol pads / chlorhexidine swab
- Intravenous catheter (pay attention to length of catheter, depth of identified vein may necessitate a longer catheter)
- Gauze
- Intravenous connectors/tubing
- Saline syringe

Support

- Consider having nurse at bedside with you to support and set up hep-lock catheter is advanced

Patient Positioning

Upper extremity should be fully supinated and then positioned such that it is perpendicular to the operator.

Ultrasound Settings

Use the linear probe and set to vascular setting. When operating the probe, ensure that the probe marker is oriented operator left when operating in short-axis/transverse view, or oriented cephalad when operating in longitudinal view.

Technique

1. Verify that the probe and screen marker matches up by placing gel on the probe face and tapping one edge.
2. Identify a vessel of interest by scanning the patient's arm in the transverse axis (probe marker oriented operator left) for a circular, hypoechoic structure.
3. When a structure of interest is found, gently compress with the US probe. Although not always the case, a compressible hypoechoic structure denotes a vein. Hold compression for 5 – 10 seconds to ensure that structure is non-pulsatile.
4. Once a vein has been found, move probe proximally and distally to determine the vessel's path. It is generally ideal to find a vein that is less tortuous to maximize the chances of successful PIV placement.
5. Sterilize region of interest with alcohol or chlorhexidine.
6. There are two techniques: transverse and longitudinal axis view approaches

Short/Transverse Axis Approach

This method employs the technique of "tip tracking," in which the operator tracks the tip of the catheter needle with the US by fanning the probe distally and/or proximally. The needle tip should be in view at all times to allow for successful venous cannulation.

1. Steady the probe on the patient's arm with the vessel of interest on the screen.
2. With the other hand, position the catheter at a 30 degree angle to the patient's arm, with the tip of the needle approximately adjacent (can be up to 1-2 cm distal) to the center mark of the probe.
3. Insert the needle through the skin. Fan the US probe in distal/proximal directions until the needle tip is visualized.
4. Continue to advance the needle, taking care to track the tip at all times.
5. Cannulate the vein with the needle with tip tracking technique. At this point, flash should have been achieved and there should be visual confirmation that the tip is within the lumen of the vessel.
6. Carefully drop the angle of the catheter until the catheter is flush with the skin.
7. Drop the probe and advance the catheter until it is fully inserted.
8. Confirm that the PIV is functioning by drawing back and flushing the catheter with saline.
9. Untie the tourniquet and attach the IV connector/tubing to the catheter.

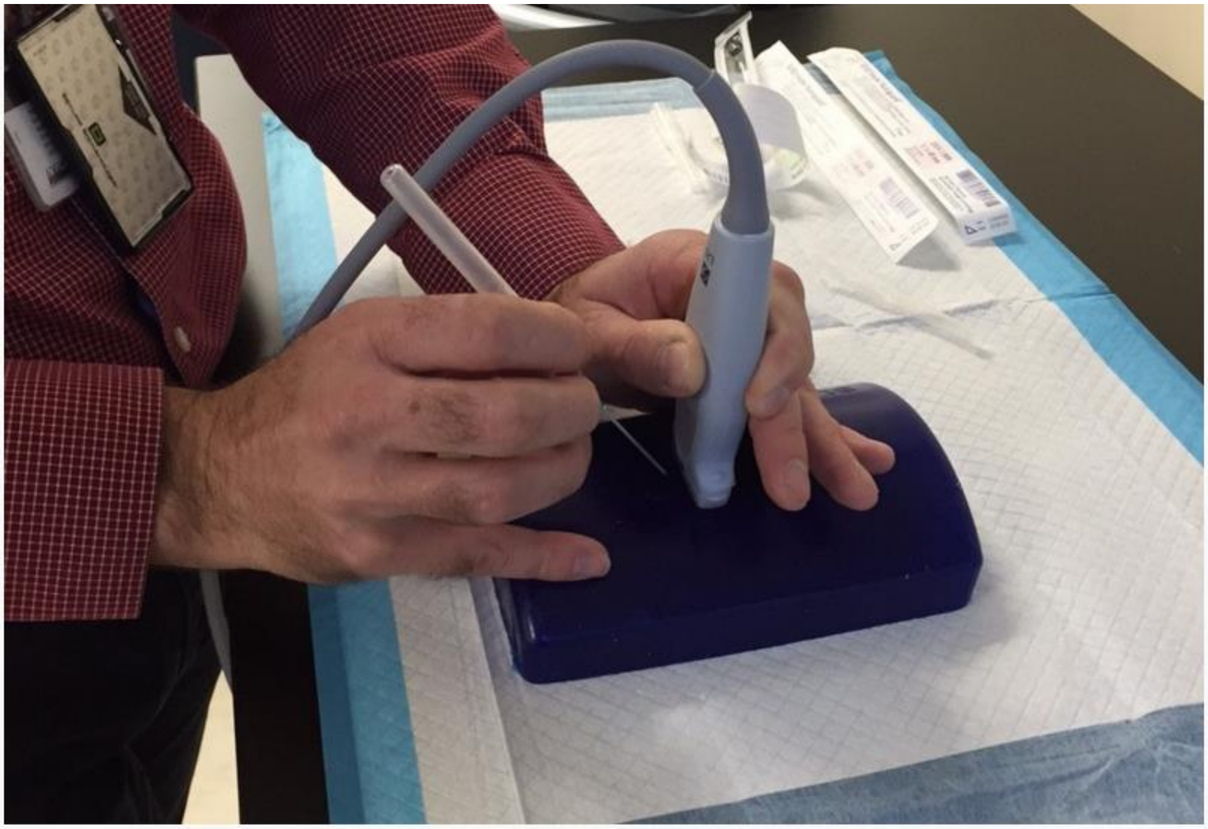

Short axis approach - note positioning of the probe and the needle relative to the probe

Longitudinal Approach

1. Center a short axis view of the vessel of interest on the screen and then rotate the probe 90 degrees, taking care that the probe does not slide. Probe marker should be oriented cephalad. At this point, the circular shape of the vein becomes a tube that is coplanar with the probe.
2. Proceed as described above

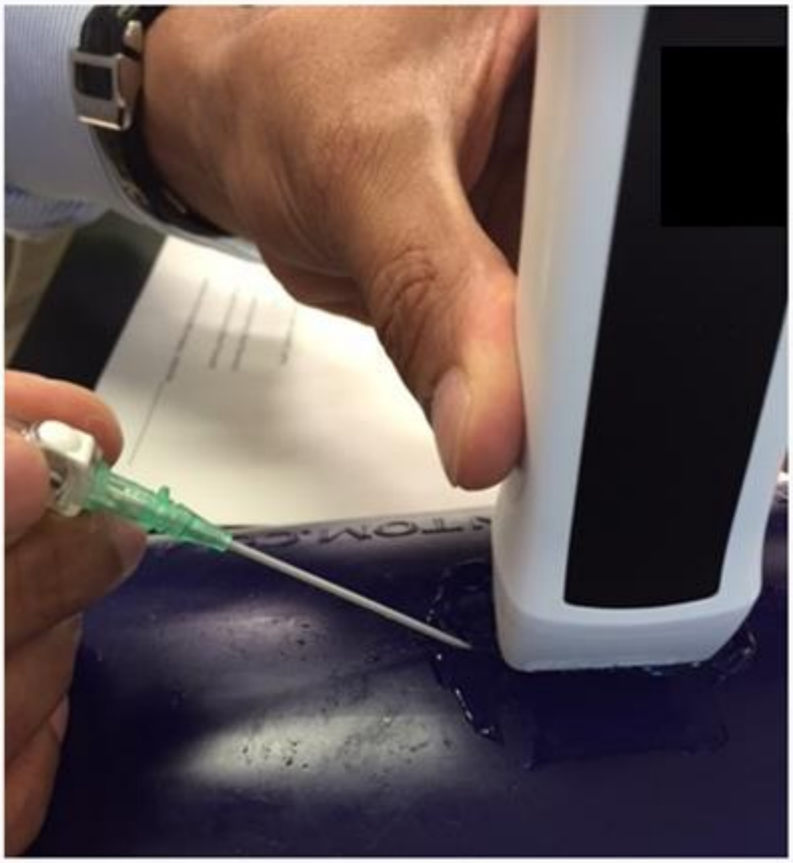

Long axis approach - note positioning of the probe and the needle relative to the probe

Pearls and Pitfalls

- When first identifying a vein, note of its depth. This will dictate the length of the catheter required for insertion.
- When attempting a PIV insertion in longitudinal view, fan the probe laterally and medially and/or rotate the probe in order to ensure that the entire vessel is coplanar with the probe.
- If it is unclear whether a hypoechoic structure of interest is a vessel, Doppler mode can be used to determine whether there is flow through the structure.
- Ensure that a vein and artery are oriented side-by-side one another to avoid cannulating an artery in the event that the catheter tip crosses through the vein.

Day 2

Abdomen- Ultrasound of Major Abdominal Vessels

Andrey Pavlov, MD and Sahar Ahmad, MD

Introduction

Ultrasound (US) is utilized for rapid assessment of the major vessels in the abdominal cavity in the critically ill patient and for screening purposes in the less acute settings. Major vessels – aorta and inferior vena cava (IVC) in the abdominal cavity are retroperitoneal structures thus low frequency probes, either phased array or curvilinear one, are utilized. Abdominal exam setting can be used for any of the abdominal structures including all scan planes of the aorta and IVC, though cardiac setting for longitudinal scanning of the IVC for fluid responsivity is recommended.

Ultrasound

The probe marker is directed to the patient's right (during the short axis, or transverse, exam) or cephalad (during the longitudinal exam). The patient should be supine with abdominal wall exposed from xiphoid process to pubic symphysis. US of the aorta is performed to screen for or diagnose abdominal aortic aneurysm. Rupture of AAA in out-of-hospital setting has 80-90% mortality. Incidence of AAA is 5% in men over 50, 10% in men over 65 with positive smoking history, resulting in ~15,000 deaths/year.

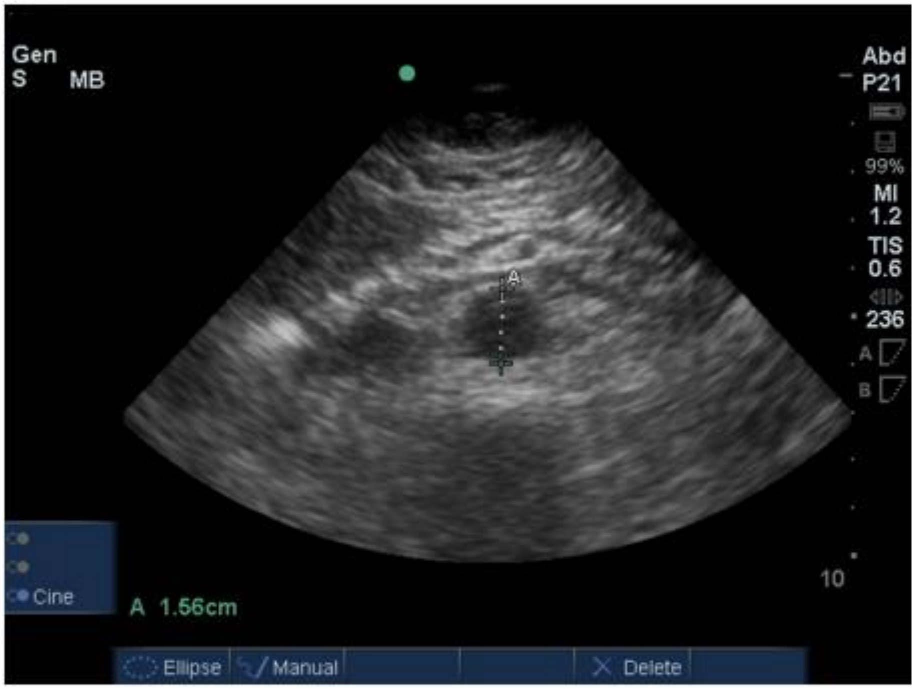

The aorta is retroperitoneal, left of the midline, and anterior to the vertebral bodies. Three scan levels are utilized on the transverse scan: (1) subxiphoid, (2) just above umbilicus (prior to aortic bifurcation into iliac arteries which corresponds to ~L4 level) and (3) midway between the prior levels. Normal infrarenal aortic diameter in men is ~1.7cm, in women ~1.5 cm, aneurysmal dilation is present if maximum diameter is 3cm or more. Aneurysm should be imaged transversely and longitudinally with maximal diameter measured at the point of the maximal dilatation from outer wall to outer wall, not to miss, dissection flap or thrombus.

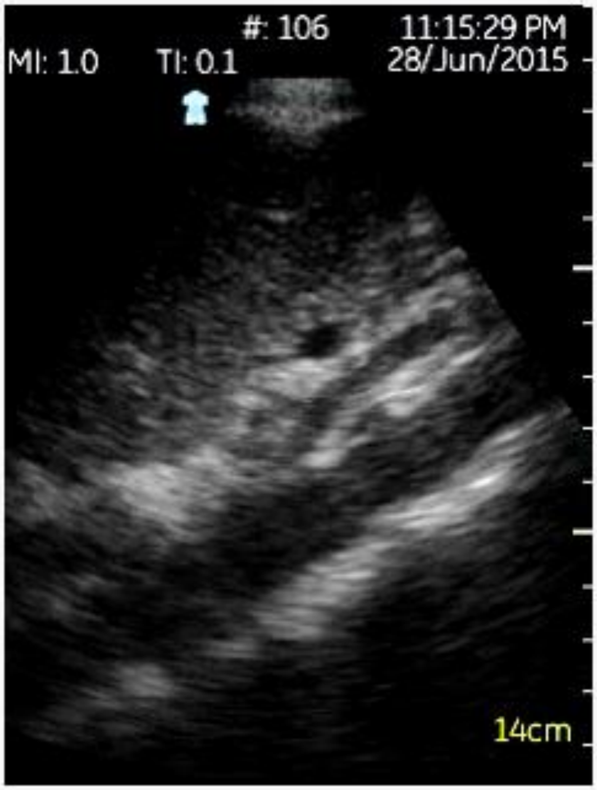

Image of the Aorta in Longitudinal view with SMA

On the longitudinal view at the subxiphoid level following structures can be easily identified: celiac trunk with early trifurcation (usually only the hepatic artery and splenic artery takeoffs are visible), takeoff of superior mesenteric artery (easily identified as running parallel and anterior to aorta), crossing of the left renal vein over the aorta under the SMA.

US of the IVC is performed to assess volume responsiveness of a patient in a shock state; IVC diameter varies with changes in intrathoracic pressure. In a non-ventilated patient it increases with expiration and decreases with inspiration. Inversely, in a ventilated patient (positive pressure ventilation), it increases with inspiration and decreases with expiration

On the longitudinal subxiphoid view proximal IVC, cavo-atrial junction, and hepatic vein can be identified. M-mode is used to obtain temporal freeze frame for measurements of maximal and minimal IVC internal diameters during one respiratory cycle. For further information please

refer to IVC and shock evaluation chapters. If limited by body habitus, right lateral approach at the level of diaphragm can be utilized to visualize IVC.

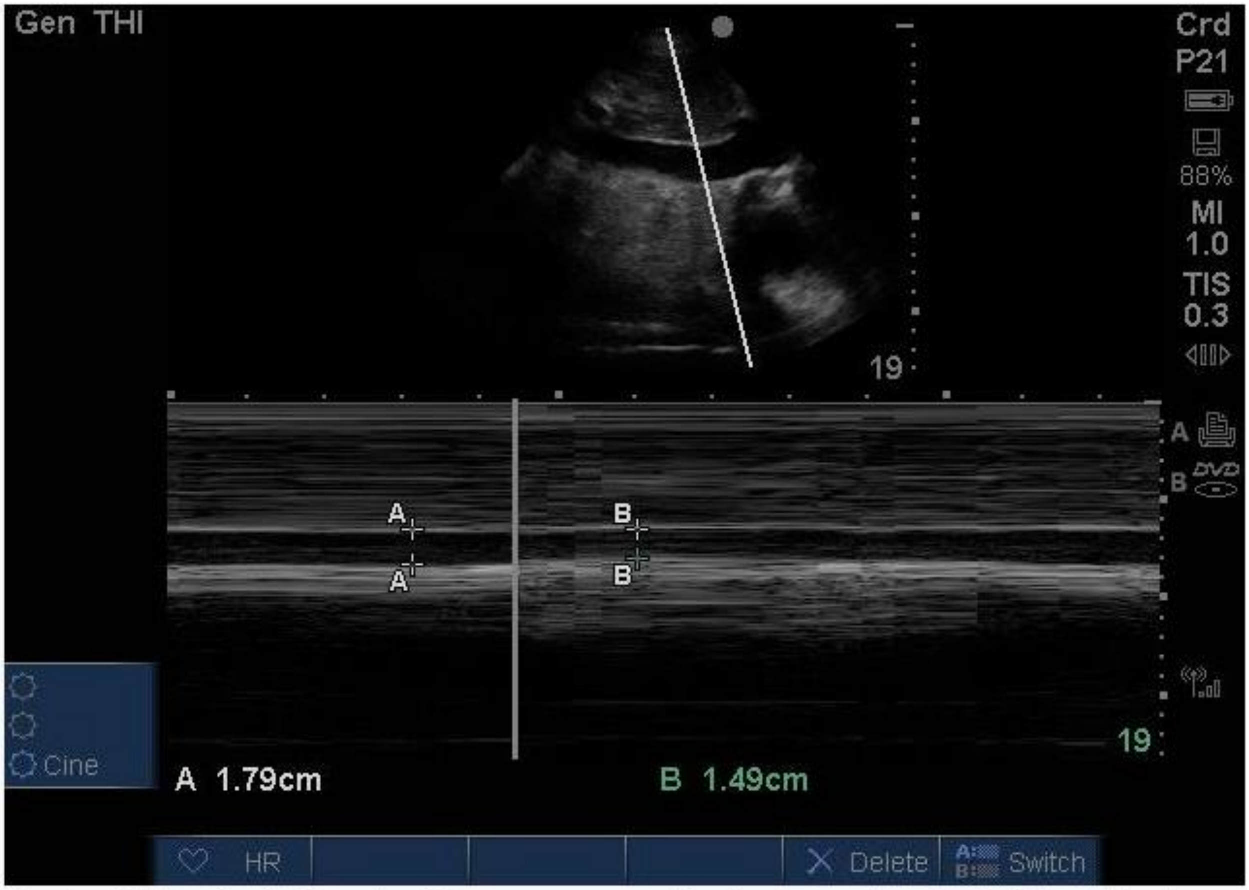

Caval-atrial junction in M mode with measurements. (See further explanation in IVC and shock evaluation chapters)

Aorta	IVC
Left of midline	Right of midline
Muscular thick wall	Thin wall
Round	Elliptical tear shaped
Pulsatile<br>Non-compressible	Compressible
Plaques may be present	No plaques
No respiratory variation	Respiratory variation
Pulsatile high velocity flow	Low velocity phasic flow

Abdomen- Rule Out Abdominal Catastrophe

Sahar Ahmad, MD

Introduction

Bedside ultrasonography (US) can be used as a rapid and dynamic imaging modality to evaluate for certain life-threatening abdominal conditions. It is often used in the setting of concern for internal bleeding in the abdomen and retroperitoneum (i.e., FAST exam) or to assess the gallbladder and biliary tree in the setting of right upper quadrant pain. We describe a protocol to rule out abdominal catastrophe or emergent surgical abdomen from any cause including pneumoperitoneum, peritonitis, or ischemic bowel. We describe also some US findings which specifically point to perforated viscous (pneumoperitoneum).

Ultrasound

The visualization of three normal sonographic findings can be used to rule out abdominal catastrophe including pneumoperitoneum. These findings are: splanchnogram, gut sliding and peristalsis.

Splanchnogram

Splanchnogram refers to the ability to visualize, with US, abdominal structures such as the liver, mesenteric fat, abdominal vessels, and bowel loops. Air will not allow propagation of US beams and therefore free air (located between the abdominal wall and the underlying organs) as in the setting of a perforated viscus, will not allow for visualization of these organs. Therefore, if free air is present at the location of the US probe, the splanchnogram sign is absent.

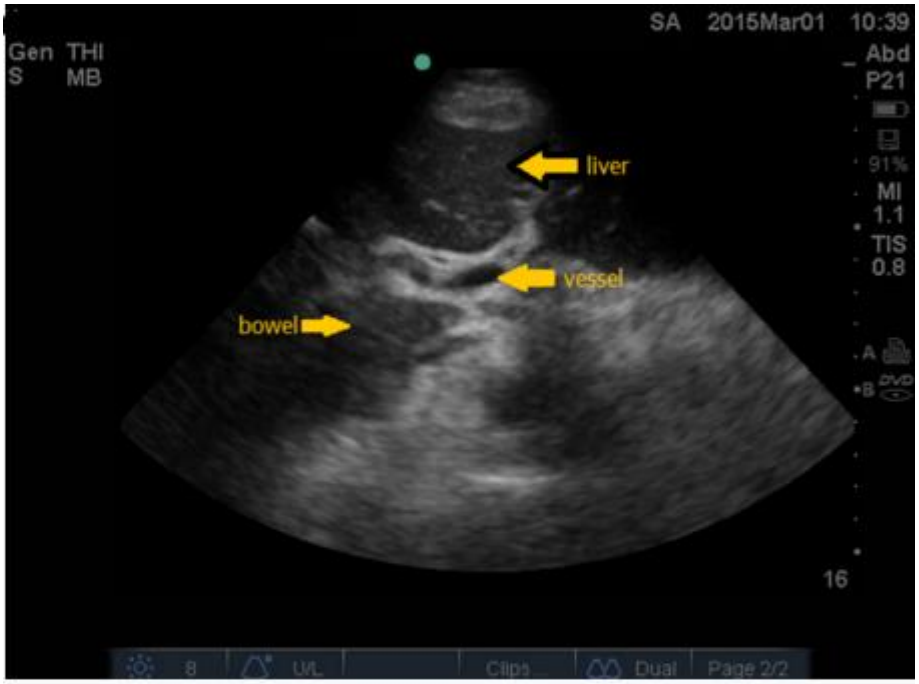

Splanchnogram

Gut Sliding

The abdominal peritoneum consists of two layers, the inner visceral peritoneum, which is wrapped around internal organs, and the parietal peritoneum, which is at the abdominal wall. Gut sliding refers to the dynamic gliding of the visceral layer during respiration against the more stationary parietal layer.

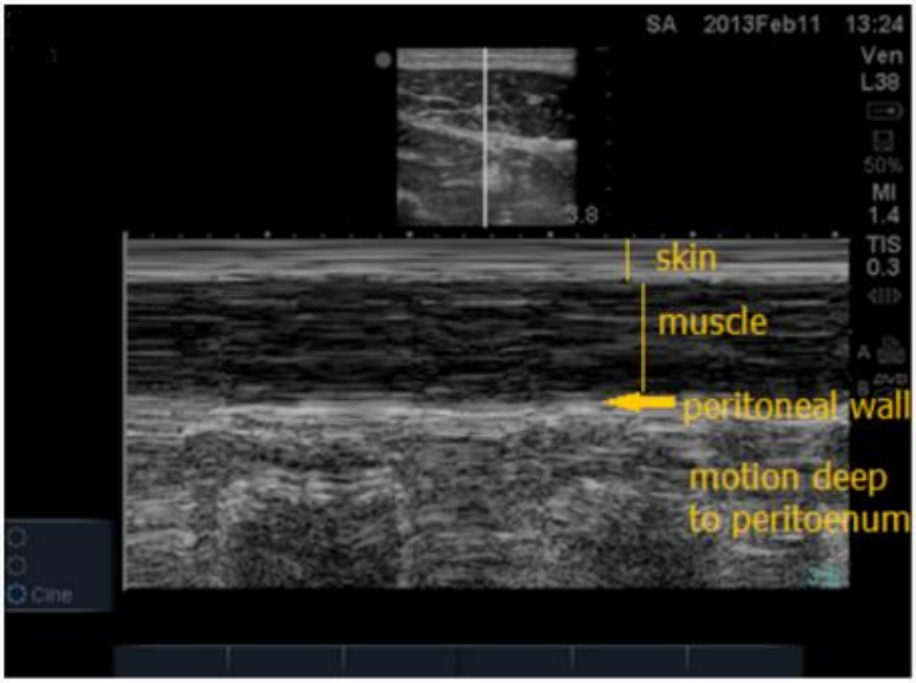

Gut Sliding - M-Mode

Analogous to the shimmering of the parietal and visceral pleura of lung sliding in pulmonary US, this sign will produce an M-mode pattern similar to the "seashore sign" seen on normal lung US. This sign will be absent when air is interposed between the two layers, as in pneumoperitoneum

Peristalsis

Intestinal peristalsis refers to the coordinated contractions of the intestinal smooth muscles. Visualized using bedside US, peristalsis appear as a circular churning motion of bowel loops. In disease processes such as mesenteric infarction or bowel perforation, there is generalized peritonitis and an accompanying ileus. These pathologies result in the absence of peristalsis.

The presence of these three findings in each hemi-abdomen effectively rules out pneumoperitoneum an abdominal catastrophe that requires immediate surgical intervention.

Pneumoperitoneum

Traditionally, pneumoperitoneum is diagnosed with an upright chest plain film. Sonography, however, has been shown to be superior to upright plain films in detecting pneumoperitoneum. In addition to the absence of gut-sliding, splanchnogram, and gut peristalsis mentioned previously, there are several characteristic sonographic findings present in patients with pneumoperitoneum. These include: (1) the Enhanced Peritoneal Stripe Sign (EPSS) phenomenon (arrow), characterized by a sudden brightening of the peritoneal line representing where the line is enhanced due to presence of air; (2) reverberation artifacts of the peritoneal line, also seen with presence of air - these enhancement artifacts may shift when the patient is placed in lateral decubitus since air follows an anti-gravity pattern; and (3) echogenic free peritoneal fluid when associated with bowel perforation - usually very small pockets and cannot always can be visualized.

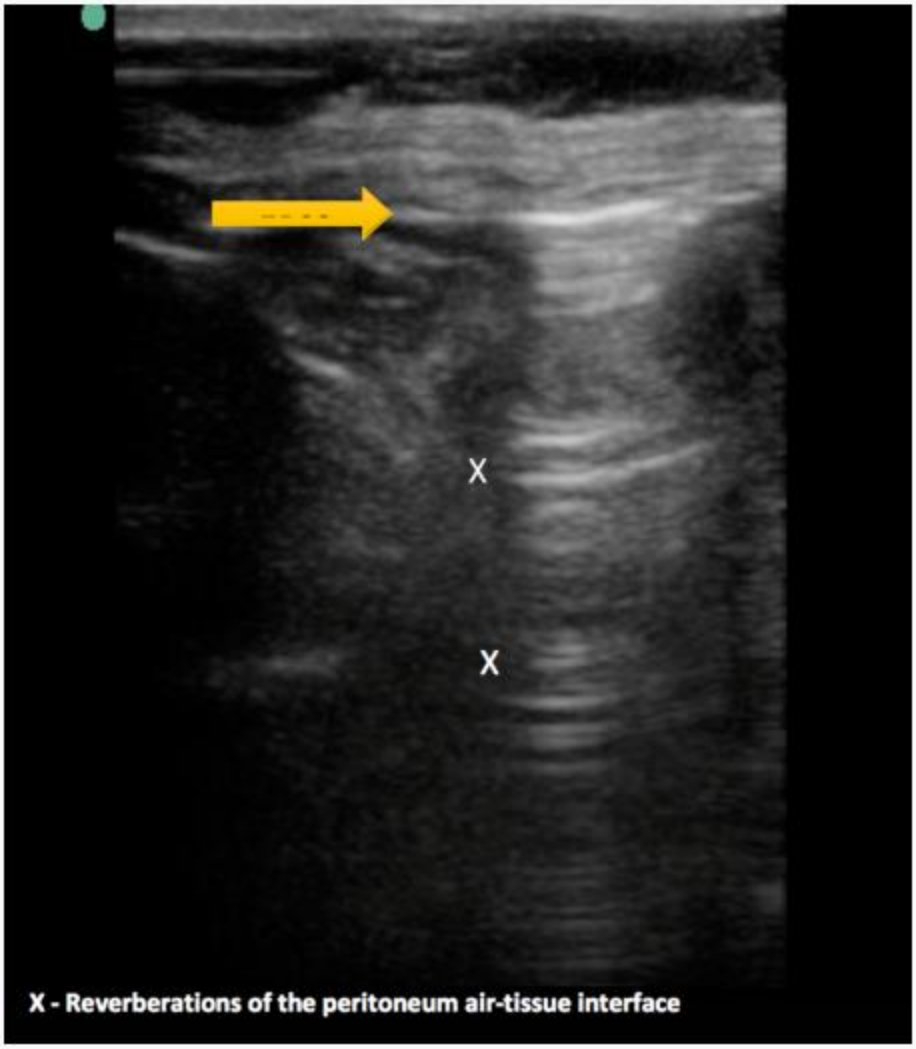

EPSS

To detect these signs, patients should be scanned in a supine position in the epigastric and right hypochondrium area superficial to the liver as this area helps remove mistaking intraluminal bowel air from free peritoneal air. To accentuate the amount of air superficial to the liver, the patient can be placed in a left lateral decubitus position.

View	Identify / Rule Out	Transducer	Machine Setting	Location
Gut Sliding	Pneumoperitoneum	Low frequency (phased array) OR Curvilinear probe<br><br>Marker cephalad	Abdominal<br><br>Screen marker operator left	Bilateral hemi-abdomen x 4 quadrants Peri-umbilical (supine)
Splanchnogram				
Peristalsis	Pneumoperitoneum Peritonitis Mesenteric infarction			Bilateral hemi-abdomen Lower quadrants

Pearls and Pitfalls

- The presence of gut-sliding, splanchnogram, and intestinal peristalsis are useful sonographic findings to help rule out pneumoperitoneum and the need for acute surgical intervention.
- Enhanced peritoneal stripe sign (EPSS), ring-down artifacts, and echogenic free peritoneal fluid are suggestive sonographic signs of pneumoperitoneum.

References

1. Chen SC, Yen ZS, Wang HP et al. Ultrasonography is superior to plain radiography in the diagnosis of pneumoperitoneum. Brit J Surg 2002;89: 351-4.
2. Hoffman, B. et al. Focus on abnormal air: diagnostic ultrasonography for the acute abdomen. EJEM 2012, 19:284-291. Lichtenstein. Whole Body Ultrasonography in the Critically Ill. Springer, 2010.

Abdomen- Evaluation of Gastric Contents

Danielle O'Hara and Sahar Ahmad, MD

Introduction

Bedside ultrasound (US) is a portable, reliable, and noninvasive diagnostic tool that has proven its utility in both emergent and critical care settings. One of the most unique aspects of bedside US is its ability to provide real-time physiologic and pathologic data that can immediately alter a patient's course of treatment. Gastric US is an example of this capability, as it exhibits its strongest utility in determining gastric contents and confirming gastric tube placement (see related chapter: Abdomen – Confirmation of Gastric Tube Placement). This modality is particularly useful prior to sedation for a procedure or during intubation because it allows the clinician to drain stomach contents thereby reducing the risk of life-threatening aspiration of GI contents into the airway.

Critically ill patients are particularly susceptible to life-threatening complications during intubation and endoscopic procedures. Both the positioning and the sedation required for these maneuvers increase the risk of aspirating blood or other gastric contents, especially when a large volume is contained within the stomach. Such aspiration can lead to cardiopulmonary complications in up to 20% of cases in the Intensive Care Unit (ICU) setting and are responsible for 50-60% of deaths in this population [1]. It is imperative, then, to identify patients that have excess gastric volume and to drain the contents prior to intubation or procedures.

It may be necessary to intubate patients in the Medical Intensive Care Unit (MICU) for a variety of reasons. This procedure requires the patient be laid flat and administration of sedatives and paralytics which reduce their ability to protect their airway. Although many of these patients are on intake restrictions, it is a well-known fact that gastric contents may remain and aspiration is possible. For those patients who are prescribed bi-level positive airway pressure (BiPAP) therapy, the risk of aspiration is even greater. This therapy supplies positive pressure during both inspiratory and expiratory efforts, thereby insufflating of the stomach and increasing the risk of rapid efflux during intubation. Gastric US has the ability to detect a full stomach before initiating intubation and thereby avoid life-threatening complications.

Upper gastrointestinal bleeding (UGIB) is also commonly encountered in the MICU and carries an overall mortality rate of 10% [2]. Large volumes of blood may accumulate in the stomach, thereby imposing a challenge in the course of treatment for UGIB patients. This collection of blood increases aspiration risk during sedation and impairs visualization during esophagogastroduodenoscopy (EGD). Incorporating gastric US into pre-operative practices can preemptively identify large gastric volumes and therefore improve perioperative patient safety.

Bedside US of the stomach may easily identify large gastric volumes, signaling the need for preventative measures such as draining stomach contents prior to intubation or procedures. Nasogastric (NG) drainage is one technique that is used to remove excess volume from the

stomach, and has shown to alleviate aspiration risk and improve endoscopic visualization when preformed before EGD [3]. This procedure presents a reliable method of draining large gastric volumes that are detected by US. By draining the full volume, the patient's risk of aspiration is eliminated. Clinicians commonly forego NG drainage to avoid inducing mucosal trauma in patients with known variceal lesions, although evidence shows that large-bore lavage tubes do not impose such trauma in these patients [3]. The procedure, however, is extremely painful for patients and should not be done with haste [4]. Gastric US therefore provides a quick and reliable method of assessing a patient's need for NG drainage at the bedside.

Ultrasound

Using a low frequency, phased array probe in the abdominal setting, gastric contents may be visualized from a left upper quadrant view. With the probe marker operator left, the probe should be placed in the mid-clavicular line tucked under the ribcage margin and angled slightly cephalad. By scanning cephalad to caudate the transverse plane, a fluid-filled stomach will be seen with its characteristic "starry night" appearance [5].

If the stomach does not have any contents, it will not be visualized due to the anatomic position of the spleen in this setting. Therefore, bedside US has a strong utility during the evaluation of acute UGIB to determine the need for NG drainage and thus augment periprocedural patient safety.

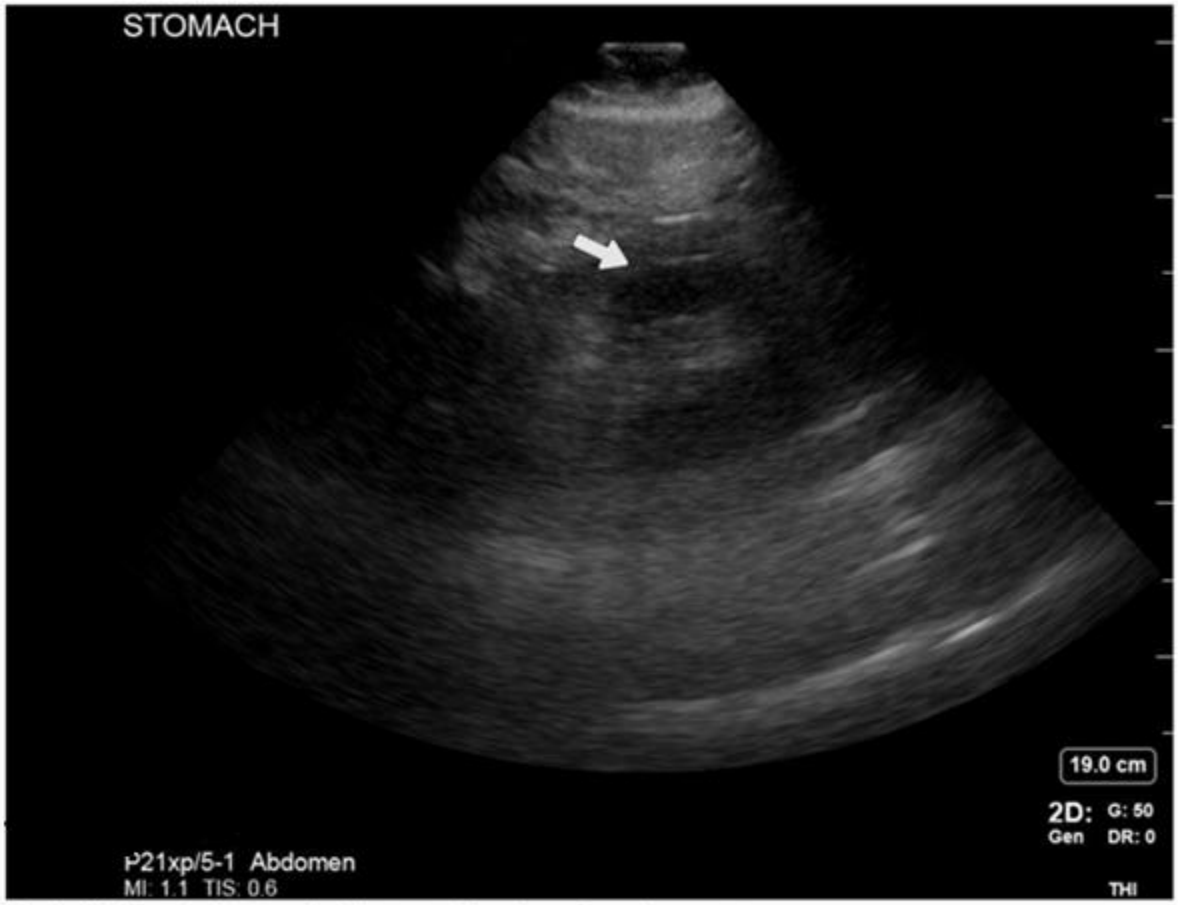

Gastric US of a patient's stomach without a notable volume of gastric contents

View	Identify	Transducer Probe Marker	Machine Setting	Location
Stomach	Body of Stomach	Low frequency (phased array)<br><br>Probe marker operator left	Abdominal<br><br>Probe marker operator left	Left mid clavicular line tucked under the rib margin with probe flat against stomach, angled slightly cephalad. Scan cephalad to caudate to identify presence or absence of gastric contents

Protocolized approach to bedside US for evaluating gastric contents:

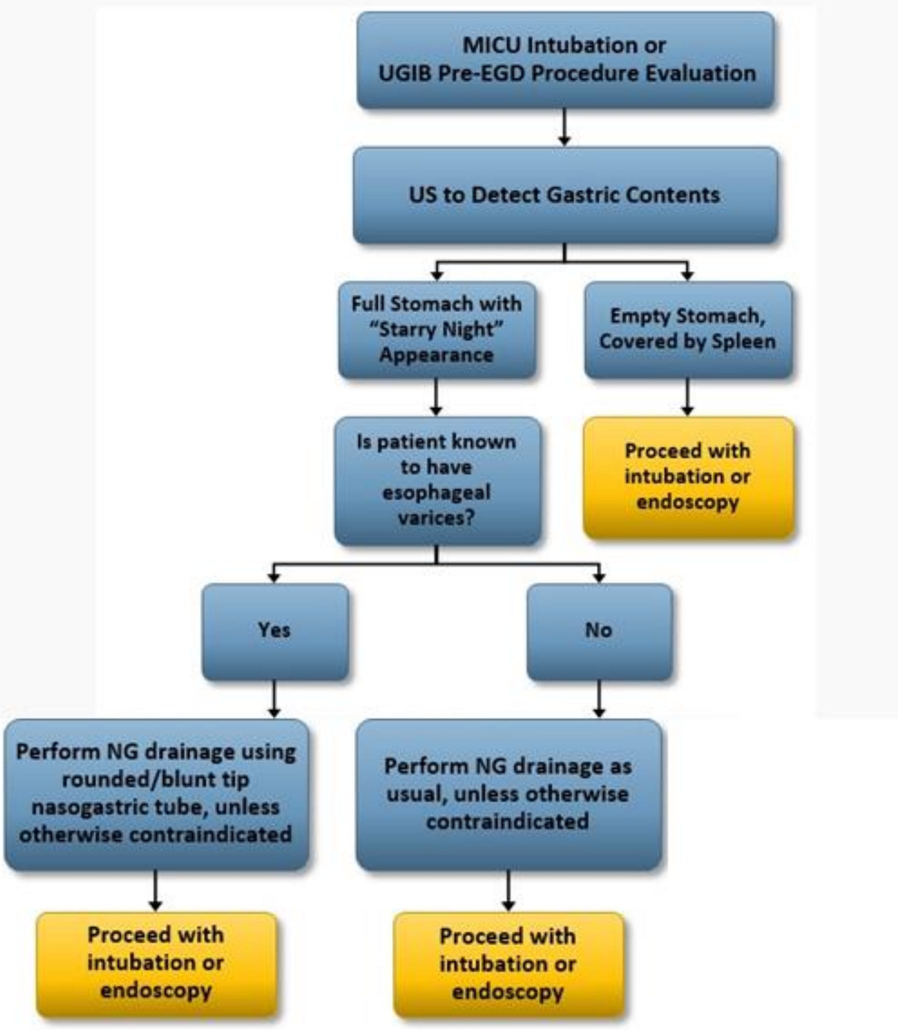

Pearls and pitfalls

- Identifying fluid-filled stomach prior to EGD or intubation procedure can prevent fatal complications
- If the stomach is not seen on US then the stomach is unlikely filled with fluid

References

1. Allescher HD. Prophylactic endotracheal intubation for emergency endoscopy in critically ill patients? Gastroenterology 2010;138:1627-9.
2. Aljebreen AM, Fallone CA, Barkun AN. Nasogastric aspirate predicts high-risk endoscopic lesions in patients with acute upper-GI bleeding. GastrointestEndosc 2004;59:172-8.
3. Lee SD, Kearney DJ. A randomized controlled trial of gastric lavage prior to endoscopy for acute upper gastrointestinal bleeding. J ClinGastroenterol 2004;38:861-5.

4. Pallin DJ, Saltzman JR. Is nasogastric tube lavage in patients with acute upper GI bleeding indicated or antiquated? GastrointestEndosc 2011;74:981-4.
5. Van de Putte P, Perlas A. Ultrasound assessment of gastric content and volume. Br J Anaesth 2014;113:12-22.

AKI- Ultrasound Approach to Acute Renal Failure

Elixabeth L. Sullivan, MD; Mark F. Sullivan, MD; Danielle O'Hara; and Sahar Ahmad, MD

Introduction

Portable renal ultrasound (US) is a valuable imaging modality for the comprehensive assessment of acute kidney injury (AKI). Bedside US is an extension of the physical examination and therefore has the potential to improve diagnostic accuracy, expedite proper treatment, and increase safe outcomes for patients with renal disease.

In this chapter, we demonstrate how renal US can help distinguish acute versus chronic kidney disease by examining renal size, echogenicity, and cortical diameter. We also review how US can be used as an adjunct tool to differentiate prerenal-, intrarenal-, and post-renal-associated AKI.

Ultrasound technique and imaging

Examine the patient in the supine or semi-recumbent position using a low frequency transducer probe in abdominal mode to evaluate the kidneys and bladder. Each kidney may also be examined in the lateral decubitus position. One may raise the ipsilateral arm above the patient's head to increase the space between adjacent ribs for better visualization of the kidney.

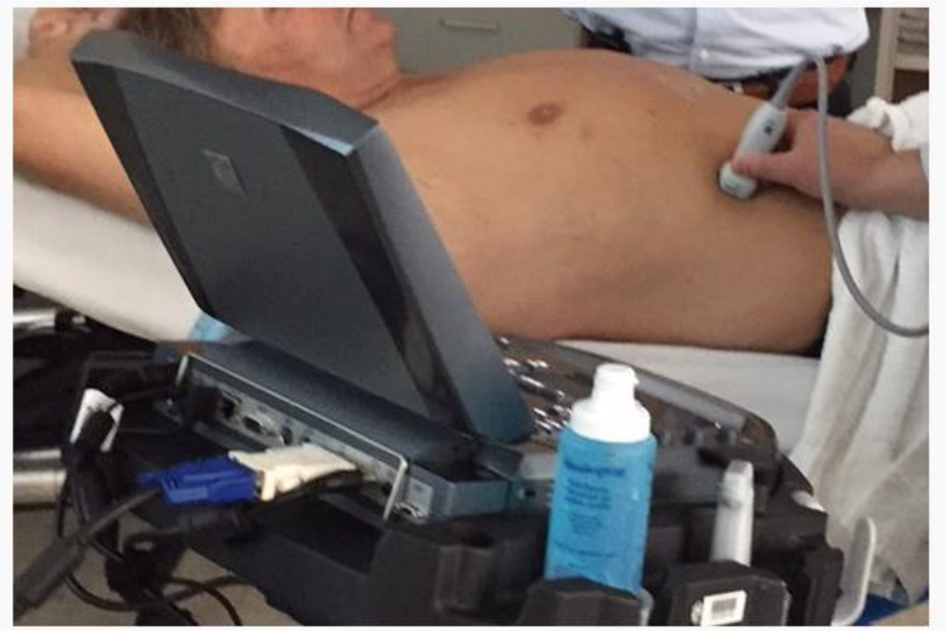

Right Kidney

Obtain a longitudinal (long axis) view by placing the transducer in the right lower intercostal space in the mid-axillary line with the probe marker facing cephalad. Use the liver as your acoustic window and angle the transducer slightly posterior (towards the kidney). Tilt the scan-plane superiorly and inferiorly in order to view the superior and inferior poles.
Obtain a transverse view (short axis) by rotating the transducer 90 degrees, such that the probe marker is now directed anteriorly. In both longitudinal and transverse views, use the freeze and calipers function to measure the diameters (length and width) of the kidney.

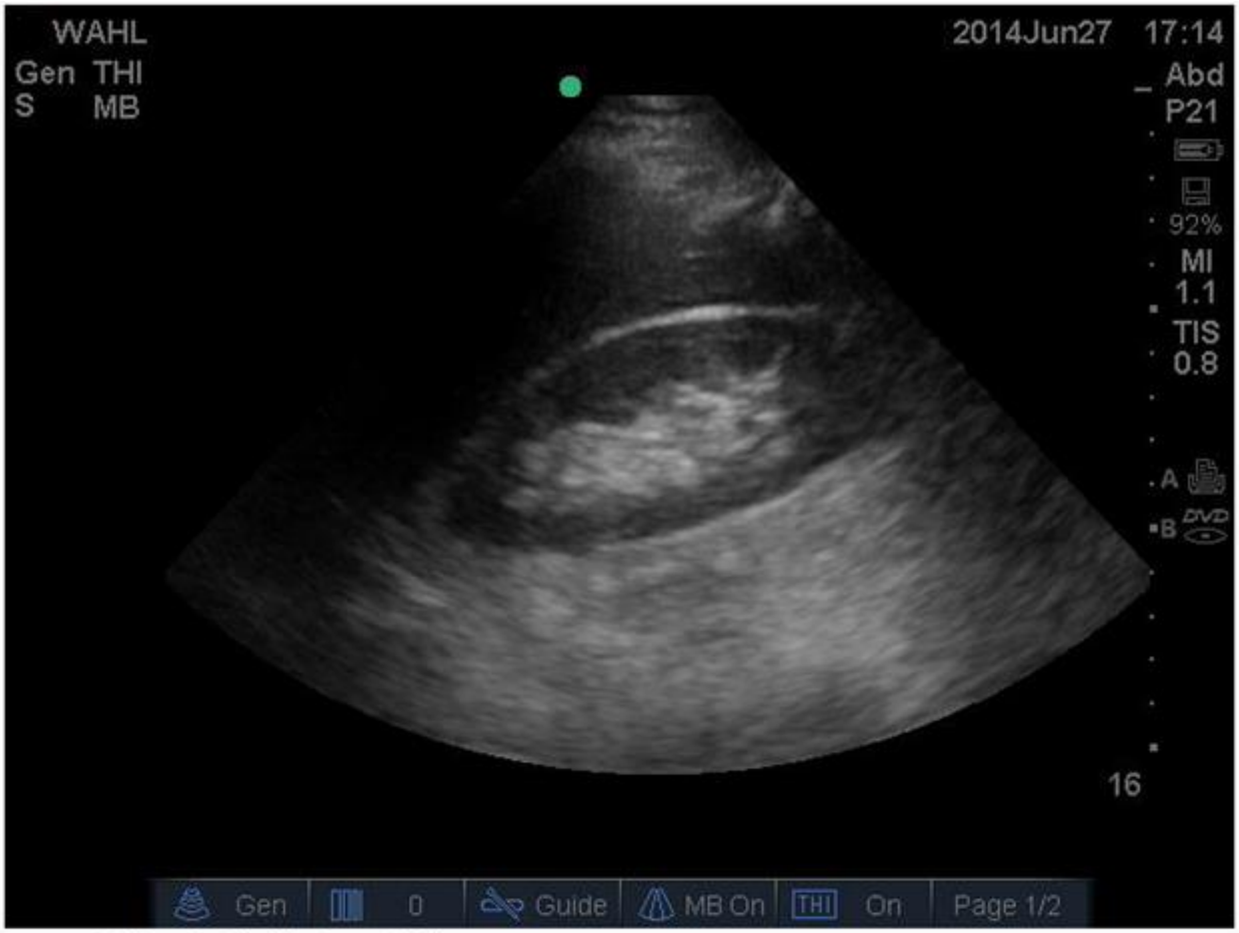

Long axis (longitudinal) view of kidney

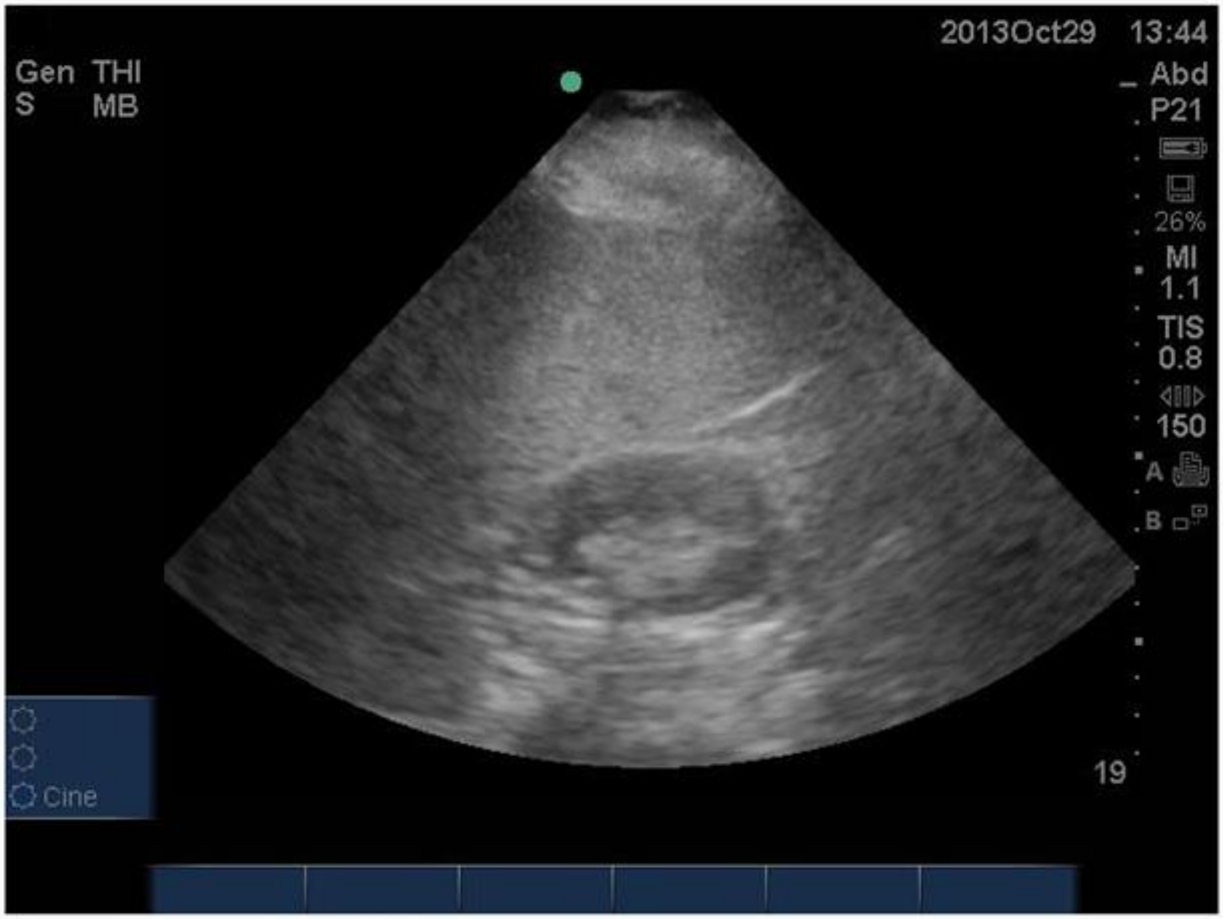

Short axis (transverse) view of kidney

On a longitudinal view, the normal kidney will appear football-shaped and will typically be 10-14 cm in length and 4-5 cm in width. Tall males are expected to have a kidney size at the larger end of the normal spectrum; petite females at the lower end of this range. On a transverse view, the kidney appears C-shaped at its end and oblong centrally.

Left Kidney

Place the transducer in the lowest intercostal space along the posterior-axillary line with the probe marker facing cephalad (probe placement is more cephalad and posterior than when examining the right kidney) to obtain a longitudinal view. Tilt the scan-plane superiorly and inferiorly to view the superior and inferior poles.

Obtain a transverse (short axis) view by rotating the transducer 90 degrees. Take measurements as described above.

Ureters

Ureters may appear as tubular structures extending inferiorly from the hilum of the kidney when they are distended.

Bladder

Position the probe marker of the transducer operator left. Place the transducer midline on the anterior abdomen just superior to the pubis. A full bladder will be square or rectangular in shape. Visualize all four walls and note whether all the fluid is contained within the bladder (normal) or there is extra-vesicular fluid present (abnormal). Often, a collapsed or unfilled bladder will not be clearly visible since fluid within the bladder is what sonographically demarcates the bladder walls on US.

A Closer Look at Renal Anatomy

The kidneys, normally measuring 10-14 cm x 5-7 cm x 4 cm, are retroperitoneal structures located between the 12th thoracic and 4th lumbar vertebrae, lateral to the psoas muscles. In the setting of specific pathology, the clinician may readily identify major renal structures using renal US including the hilum, pelvis, calyces, cortex, and medulla. When identifying and describing various renal structures it is helpful to use varying echogenicity as a guide. The renal capsule surrounding the kidney is hyperechoic, nearly white. The cortex is slightly echogenic but not as bright as the capsule. It is a thin layer and often not distinctly discernible from the rest of the renal parenchyma which is slightly hypoechoic (dark grey).

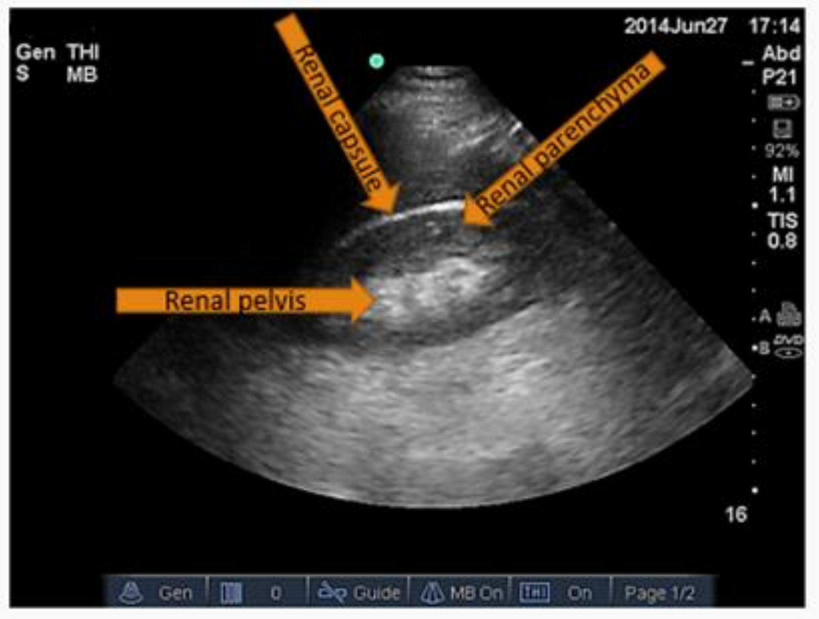

The medullary pyramids are hypoechoic, triangular structures between the cortex and the renal sinus. The pyramids are only distinctly visible when they are full of urine and enlarged (as in cases of hydronephrosis). The renal parenchyma, therefore, including the calyces and the cortex normally appear homogenously hypoechoic. Finally, the renal sinus is echogenic due to high fat content, and becomes anechoic when it fills with urine.

Evaluation of Acute versus Chronic Kidney Disease

Size and echogenicity are two helpful clues that can help distinguish AKI and chronic kidney disease (CKD). In AKI, kidneys are usually normal or slightly increased in size, whereas in CKD, the kidneys are small, thin, and demonstrate irregular borders. An increase in echogenicity of the renal parenchyma occurs when there is an increase in material that can reflect sound waves back, thus increasing its brightness on the US image. Thus, CKD is typically associated with increased echogenicity since fibrous tissue is increased in states such as interstitial fibrosis and glomerulosclerosis. Increased echogenicity was reported to have 96% specificity (and 67% positive predictive value) for the presence of parenchymal kidney disease.[1] It is unusual for renal echogenicity to be less than that of the liver in patients with CKD. In one study, only 11% of patients with CKD (serum creatinine 8 mg/dl) had kidney echogenicity that was less than liver echogenicity.[2] Furthermore, when US characteristics were directly compared with renal histopathology, the combination of small size and increased cortical echogenicity was strongly correlated with chronic, untreatable kidney disease.

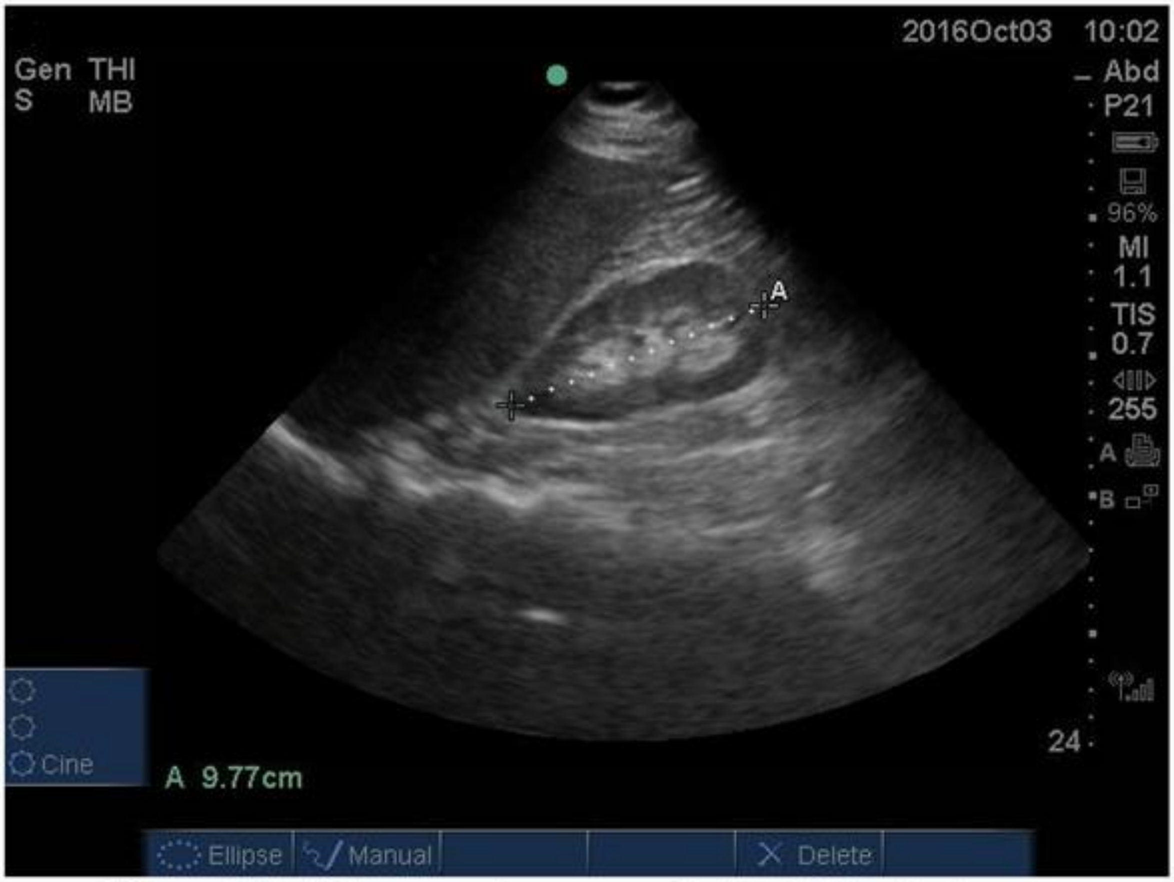

CKD: a small sized kidney, as seen here, or one with distorted/blurred and unrecognizable sonographic anatomy, as shown below, suggests CKD.

The recognition of CKD is the most reliable utility of bedside US during the AKI workup, and so kidney size and general appearance should be paid attention to, as detailed above.

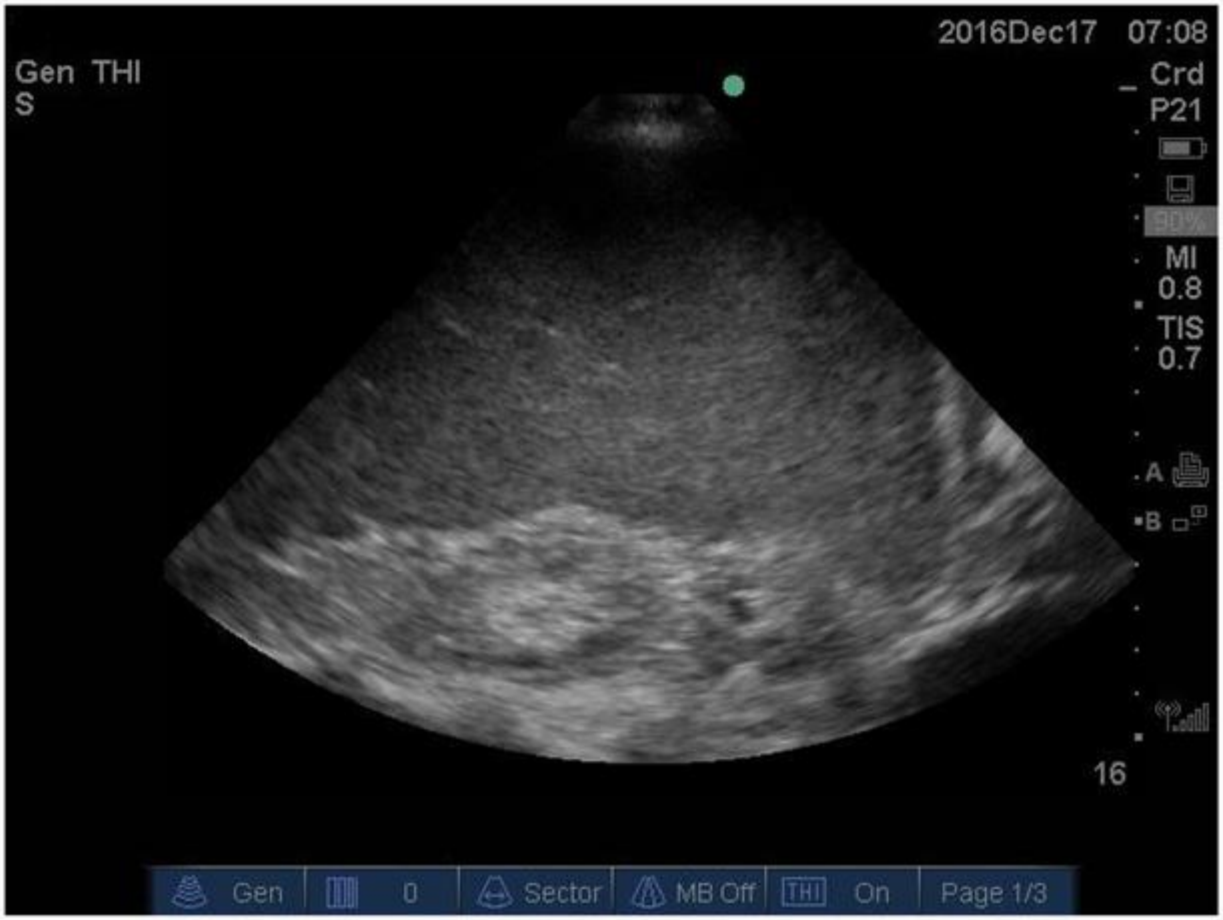

CKD: blurring of the usual sonographically distinct layers is a feature commonly seen in CKD of medical-renal disease including chronic hypertension or diabetes mellitus.

Pre-renal AKI: Assessment of the IVC

The use of portable US to estimate intravascular volume status and extravascular lung water has been validated in the management of acute respiratory failure and heart failure.[3] These parameters can also provide objective data in determining volume status in patients with pre-renal AKI (please refer to chapter on IVC measurement). The lack of radiation, convenience of a bedside procedure, and accessibility make US an affordable and safe image modality which can be learned and interpreted easily.[2] Barbier and others described the correlation between IVC calibers and fluid responsiveness during shock.[4] In pre-renal states patients are usually volume depleted secondary to either intra- (e.g., diuretics) or extra-renal (e.g., dehydration, diarrhea, vomiting, insensible fluid loss) causes. By properly assessing volume status, a physician may be able to estimate the degree of volume depletion and expedite management decisions.

Intra-renal diseases: Evaluation of the renal cortex and parenchyma

<u>Pyelonephritis</u>

An acute kidney process such as acute pyelonephritis results in normal or slightly enlarged, hypoechoic kidneys due to the presence of edema. There often is loss of differentiation between the cortex and the medullary pyramids. There can also be micro-abscess formation that appear as hypoechoic areas with smooth or irregular margins and internal echoes. In chronic pyelonephritis, kidneys appear small with evidence of linear, hyperechoic scars from the capsule to the renal cortex.

Glomerulonephritis

US findings of acute glomerulonephritis include enlarged kidneys due to parenchymal swelling with increased echogenicity and increased corticomedullary differentiation. In chronic glomerulonephritis, kidneys are atrophic with increased echogenicity and a loss of cortical thickness.

Renal Cysts

Simple renal cysts can be found in approximately 50% of the population and have an increased incidence with age. Renal cysts appear as anechoic structures with a thin capsule, lack of internal vascularity and posterior enhancement. Cysts are usually benign. Multiple cysts raise suspicion for polycystic kidney disease, which involves both kidneys in 83% of cases. Complex cysts usually have thickened walls, septations, and can contain echogenic material.

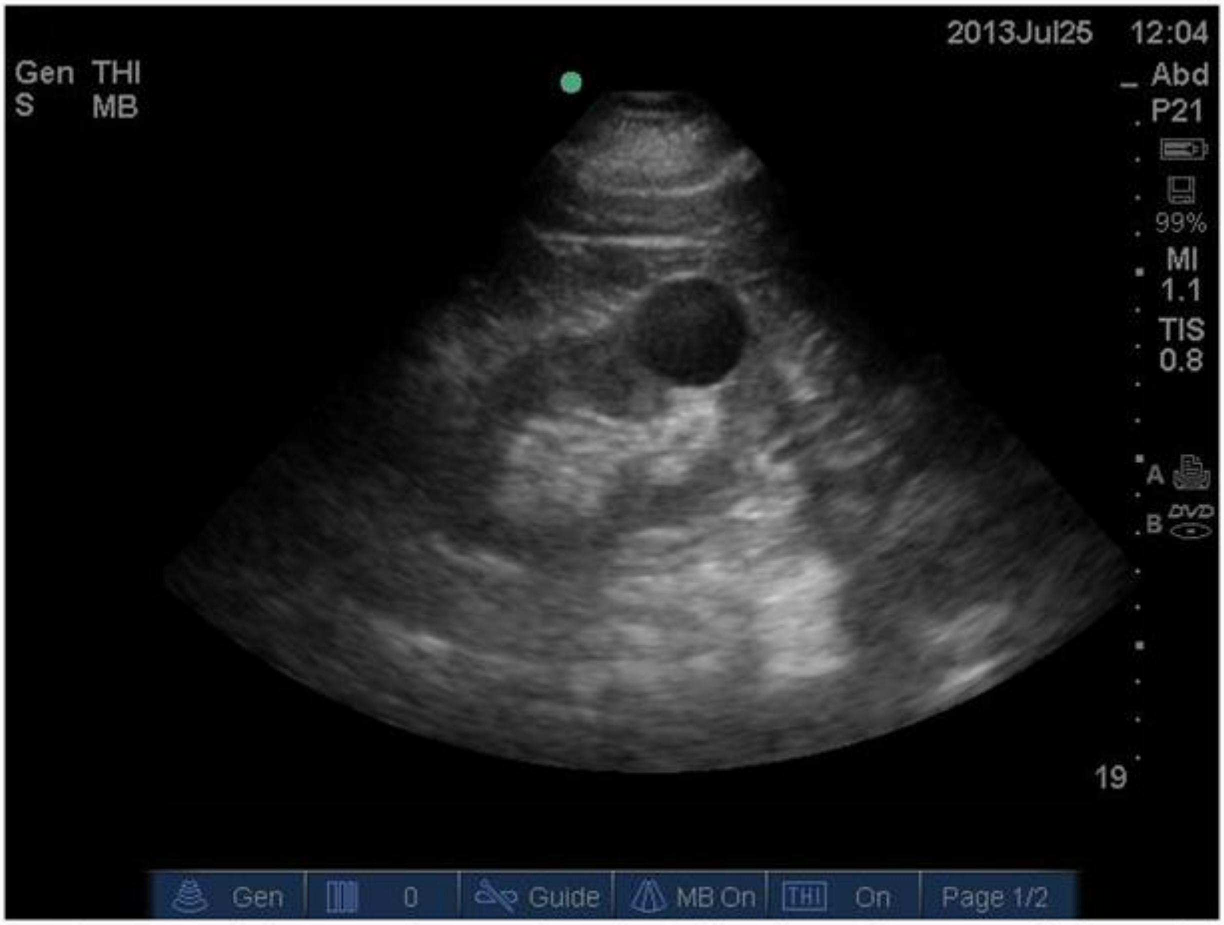

Simple cyst: Simple benign renal cysts are located typically at the periphery, are perfectly round in shape and have a purely anechoic and homogenous appearance. They are common, and when found, they are unlikely to contributory to AKI.

Please note:

In general, intrinsic renal disease will require additional work up, and bedside US alone cannot be relied upon for a specific diagnosis. Identification of AKI on a background of CKD can be established if there is clinical context and undersized kidneys (see earlier in this document descriptors of normal size parameters).

Post-renal diseases: Evaluation of renal pelvis, calyces and bladder

<u>Hydronephrosis</u>

Hydronephrosis on US appears as dilation of the renal pelvis and calyces. Maintaining a broad differential is important which includes obstruction (e.g., stone, clot, strictures, etc.), chronic infection, benign prostatic hyperplasia (BPH), ureter pathology, neurogenic bladder, and pregnancy. If hydronephrosis is identified, the contralateral kidney and the bladder should be

viewed as well. Unilateral hydronephrosis suggests a unilateral upper genitourinary tract obstructive process such as nephrolithiasis or unilateral ureteral compression. Bilateral hydronephrosis with an enlarged urine filled bladder suggests a distal lower genitourinary obstruction at the level of the urethra or neurogenic bladder. In the case of an indwelling foley, misplacement or obstruction of the foley would be the etiology. Bilateral hydronephrosis with a collapsed bladder may represent either bilateral nephrolithiasis, usually in the setting of a systemic disease that promotes stone formation, or a compressive abdominal process such as abdominal carcinomatosis, retroperitoneal fibrosis, or abdominal compartment syndrome.

In later stages of severity, the major then minor calyces fill up with fluid, creating finger- like protrusions, and the kidney becomes enlarged in size and distorted in shape as seen here:

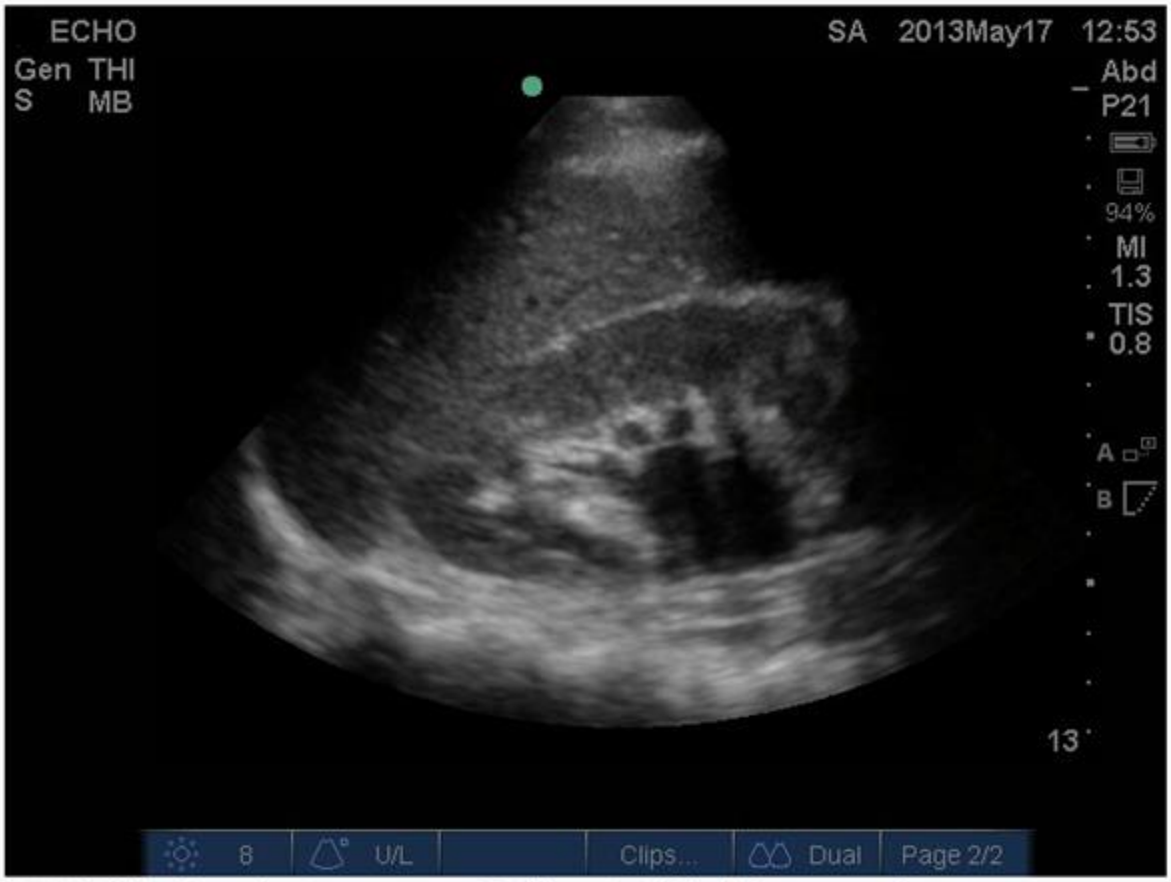

Early (mild) hydronephrosis appears as a fluid (anechoic) filled hilum as seen here.

<u>Nephrolithiasis</u>

Renal stones are either incidental findings or are discovered in the setting of obstruction of the ipsilateral renal collecting system. Most renal stones are calcified, located in the renal collecting system and display distal shadowing. Differentiating stones from the renal sinus can be challenging due to their similar echogenicity (increase frequency to better visualize the stone).

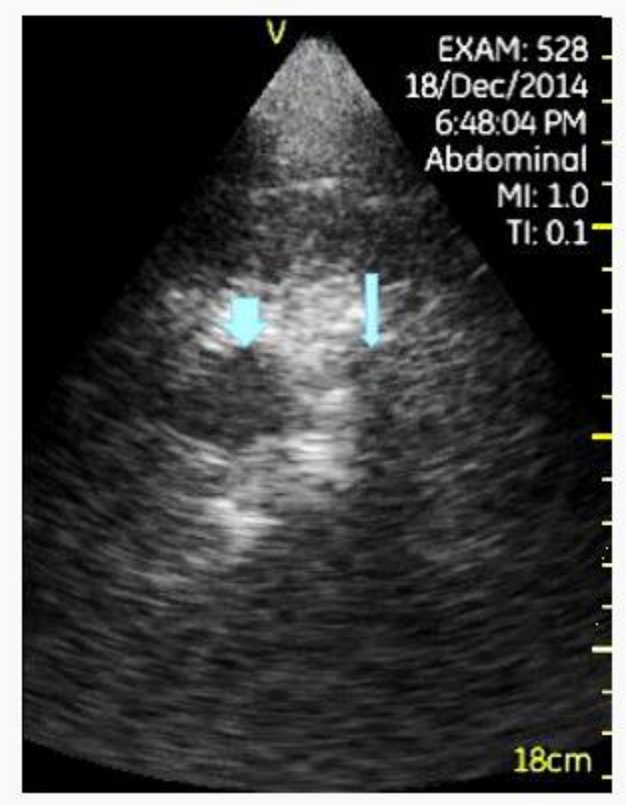

Seen here is an example of cyst and stone in the kidney. Note that while the cyst (thick arrow), which has fluid all around, has a visible back- wall (posterior edge) the stone (thin arrow) has a shadowing effect at the posterior edge and only the anterior edge of the stone can be visualized sonographically. This is because stone, like bone, will reflect beams from the anterior edge of the structure but absorb remaining beams that pass through this initial edge, creating a shadowing effect due to absence of data returned to the beam source transducer.

Evaluation of the bladder

As described above, US of the bladder to determine the presence of urine is part of the AKI workup, especially when obstructive AKI is suspected. Further evaluation of the bladder can yield additional information regarding renal pathology.

Ideally, the bladder should be full to achieve optimal wall delineation. In men, the prostate gland can be seen under the bladder neck. In women, the uterus may be seen in the midline, posterior and superior to the bladder. Bladder wall thickening is defined as width of > 0.4 cm. Focal thickening can be found in cystitis, endometriosis, tumors, and BPH. Diffuse thickening can be seen in inflammation, infection, prolonged catheterization, neurogenic bladder, and drug toxicity.

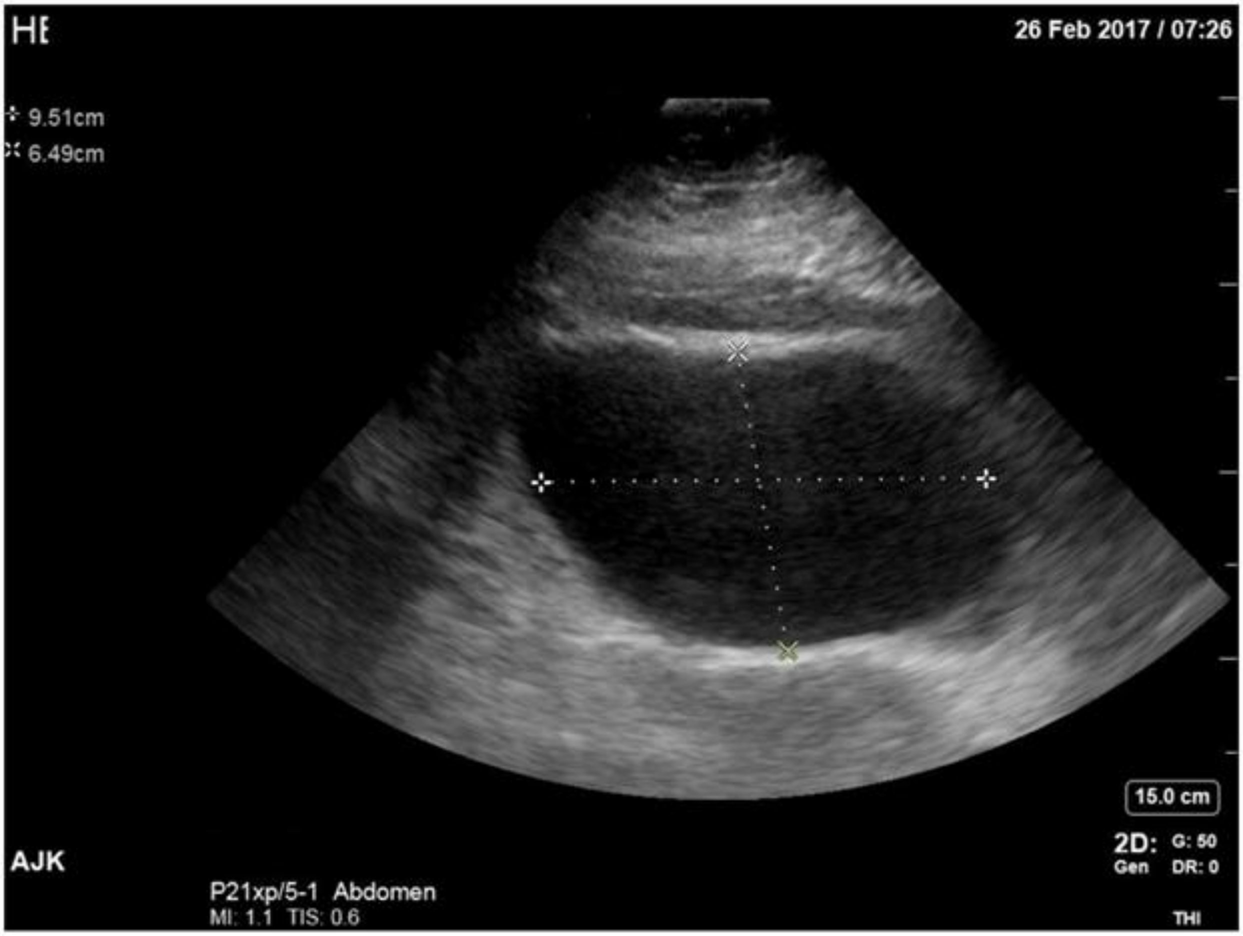

A full bladder as shown here amongst the caliper hash marks, can be seen in post- obstructive AKI, commonly due to neurogenic bladder (or other primary bladder retention), urethral obstruction including prostatic hypertrophy or urethral stone, or misplaced foley. Note, in the above image there is no foley balloon present, however if this were the case, one would suspect an appropriately placed foley with a distal foley catheter obstruction due to clog or tubing kink.

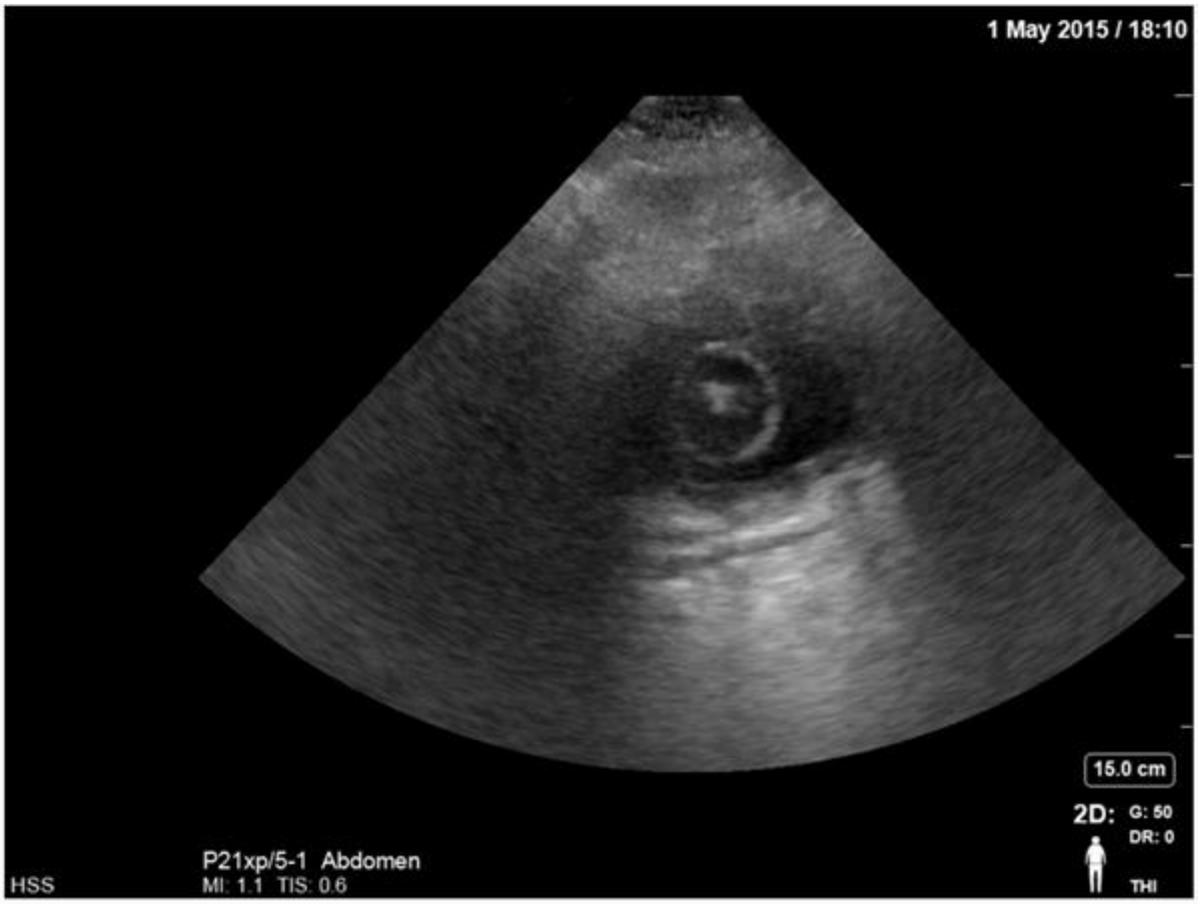

Foley Catheter in Bladder

Finally, the presence of ureteral jets, delineating the presence of urine flow into the posterior edge of the bladder, can be interrogated. Color flow doppler is used to identify these flows, by placing a color doppler interrogation box over the posterior-lateral aspects of the bladder as seen on a transverse US view taken from the anterior suprapubic region.

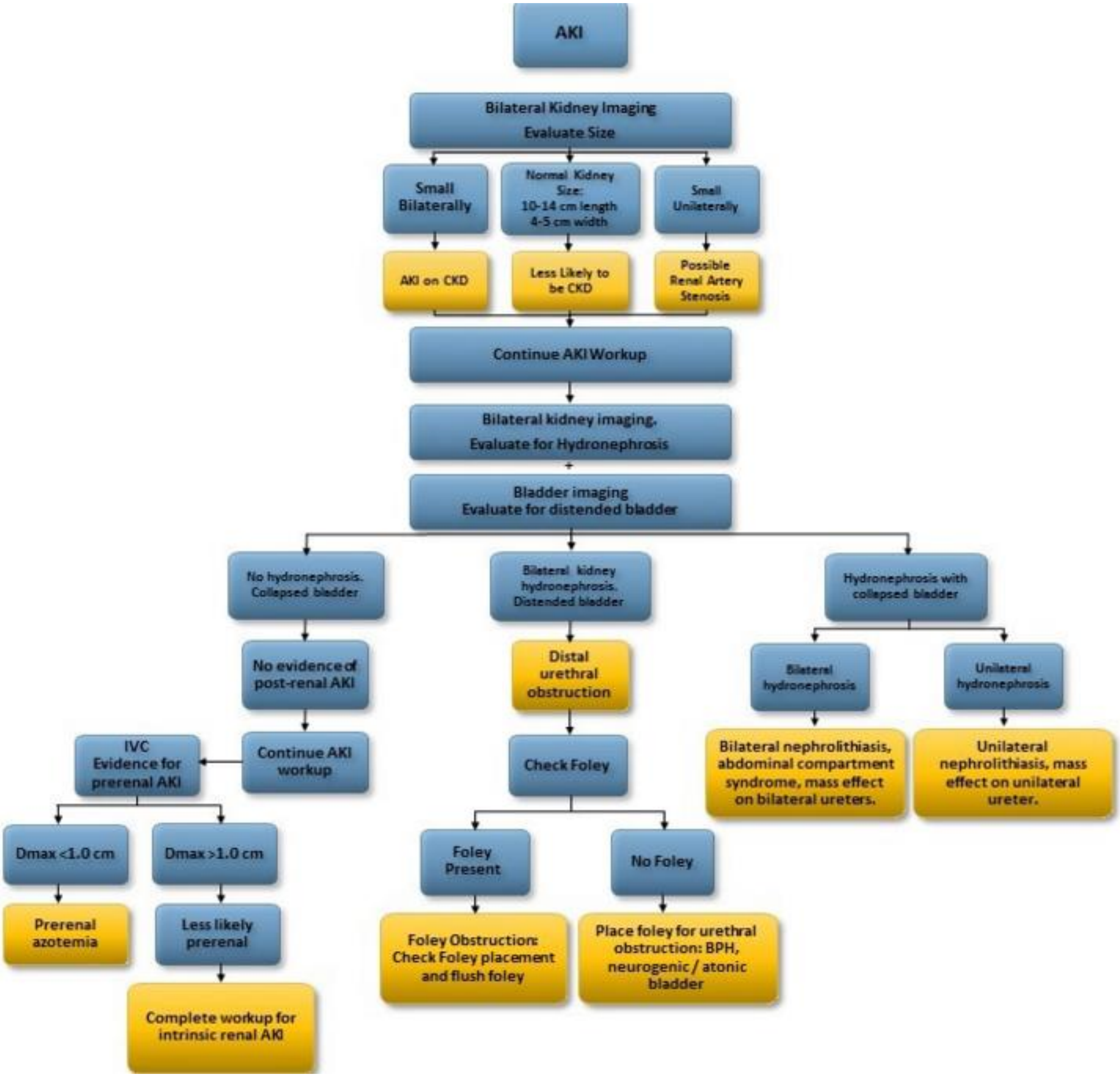
AKI
Bilateral Kidney Imaging
Evaluate Size
Small Bilaterally
Normal Kidney Size: 10-14 cm length 4-5 cm width
Small Unilaterally
AKI on CKD
Less Likely to be CKD
Possible Renal Artery Stenosis
Continue AKI Workup
Bilateral kidney imaging. Evaluate for Hydronephrosis
Bladder imaging
Evaluate for distended bladder
No hydronephrosis. Collapsed bladder
Bilateral kidney hydronephrosis. Distended bladder
Hydronephrosis with collapsed bladder
No evidence of post-renal AKI
Distal urethral obstruction
Bilateral hydronephrosis
Unilateral hydronephrosis
IVC Evidence for prerenal AKI
Continue AKI workup
Check Foley
Bilateral nephrolithiasis, abdominal compartment syndrome, mass effect on bilateral ureters.
Unilateral nephrolithiasis, mass effect on unilateral ureter.
Dmax <1.0 cm
Dmax >1.0 cm
Foley Present
No Foley
Prerenal azotemia
Less likely prerenal
Foley Obstruction: Check Foley placement and flush foley
Place foley for urethral obstruction: BPH, neurogenic / atonic bladder
Complete workup for intrinsic renal AKI

Pearls and Pitfalls

- The workup of AKI including oliguria/anuria should include, and is often initiated by bedside US. Pre-renal azotemia due to reduced preload is suggested when the IVC is small in caliber (Dmax<1.0cm) as these findings suggest a low CVP and reduced preload hemodynamics.

- Suspected intrinsic kidney disease deserves workup beyond bedside US.

- Post-renal obstruction findings on ultrasound include hydronephrosis and/or a distended bladder.

References

1. 1. Platt JF, Rubin JM, Bowerman RA, Marn CS. The inability to detect kidney disease on the basis of echogenicity. Am J Roentgenol 151: 317-319, 1988
2. Ozmen CA, Akin D, Bilek SU, Bayrak AH, Senturk S, Nazorglu H. Ultrasound as a diagnostic tool to differentiate acute from chronic renal failure. ClinNephrol 74: 46-52, 2010.
3. Moore, C.L. and J.A. Copel, Point-of-care ultrasonography. N Engl J Med, 2011. 364(8): p. 749-57.
4. Barbier, C., et al., Respiratory changes in inferior vena cava diameter are helpful in predicting fluid responsiveness in ventilated septic patients. Intensive Care Med, 2004. 30(9): p. 1740-6.

Shock- IVC Examination for Fluid and Vasopressor Administration in Shock

Rian Shah, MD and Sahar Ahmad, MD

Introduction

Shock State is one of the most common conditions encountered in Critical Care Medicine. It can be understood as a low cardiac output state usually accompanied by systemic hypotension due to either reduced cardiac contractility (cardiogenic shock), or reduced circulating blood volume (either total intravascular blood volume is low in case of hypovolemic shock; or effective circulating blood volume is low in the case of septic/distributive shock) [1].

Stabilization of these patients depends on first identifying the etiology of shock then directing therapy for treatment:

- In the case of cardiogenic shock: increase cardiac contractility by use of inotropes
- In the case of septic/distributive shock, increase SVR by use of vasopressors.
- In the case of hypovolemic shock, increase total circulating intravascular volume by administration of fluids.

Not only does efficient delineation of these forms of shock result in efficient management, it allows the physician to avoid harmful therapies such as:

- Inappropriate administration of vasopressors or inotropes to the hypovolemic patient which will lead to worsened tissue hypoperfusion.
- Inappropriate administration of fluids to a patient with reduced cardiac contractility which can lead to worsening of heart failure by distention of myocardial fibers off of the Starling Curve, leading to acute worsening of heart failure and/or pulmonary edema [2].

To this end, ultrasonographic assessment of the respiratory changes in inferior vena cava (IVC) diameter has arisen as a reliable and effective metric to predict which shock patients will have a cardiac output which is responsive to fluid bolus, thus allowing us to avoid any deleterious effects caused by inappropriate fluid or pressor administration.

Key concepts

To understand the value and limitations of this technique lets first begin with a brief review of how the diameter of the IVC responds to changes in preload and intrathoracic pressure variations during (1) negative pressure ventilation (Physiologic and non-intubated patients) vs. (2) mechanical ventilation (positive pressure ventilation), and (3) the relationship between preload and stroke volume by way of frank starling physiology.

In non-intubated patients the intrathoracic pressure decreases during inspiration, thus causing a transient decrease in right atrial pressure and increase in venous return or "preload." This then causes a collapse of the IVC as blood is drained into the heart [3] and we can measure a collapsibility index of the IVC [4]:

$$cIVC = \frac{Dmax - Dmin}{Dmax}$$

Conversely, in mechanically ventilated patients the intrathoracic pressure transiently increases during inspiration as the ventilator forces air into the lungs. This increase in intrathoracic pressure causes an increase in the right atrial pressure and a decrease in venous return or "preload" which then causes the IVC to distend as blood temporarily pools on the venous side of circulation [3] and we can measure the distensibility index of the IVC [2]:

$$dIVC = \frac{Dmax - Dmin}{Dmin}$$

Finally, let's put it all together by looking at the following figure which plots both IVC variability (blue) as a function of preload, and the frank starling curve (red). On the left of the figure we can see that states of low preload correlate to those of high IVC variability and the steep part of the frank-starling curve. Thus indicating that the patient is fluid responsive, meaning that the administration of fluids will increase cardiac output. Conversely, the right of the figure demonstrates that states of high preload correspond to low IVC variability and the flat to down trending parts of the frank starling curve. This indicates that the administration of fluids to increase preload will not increase cardiac output. In fact the administration of excessive amounts of fluid may even be deleterious, as the excess preload will lead to excessive cardiac stretch and failure as shown in the far right of the figure.

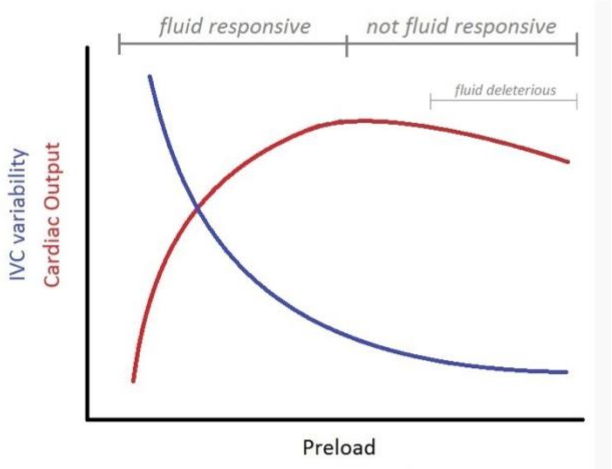

1. Ensure that the non-intubated patient is breathing calmly (tidal breathing) and that the intubated mechanically vented patient is adequately sedated and in synchrony with the ventilator.
2. Begin the exam by selecting the phased array - low frequency probe.
3. With the marker cephalad, place the probe at a 90° angle to the patient in the sub-xiphoid region.
4. Visualize the Cavo-atrial junction and the proximal IVC in
5. Identify aorta and IVC both by angling the probe back and forth from medial and right lateral.
6. Select M-Mode and choose a beam corresponding to a near orthogonal section of the IVC 2-4 cm distal to the cavo-atrial junction or just posterior to the hepatic vein (image 1)
7. Using the caliper tool measure the maximum (Dmax) and minimum (Dmin(anterior-posterior diameters of the IVC lumen over one complete respiratory cycle (image 2).

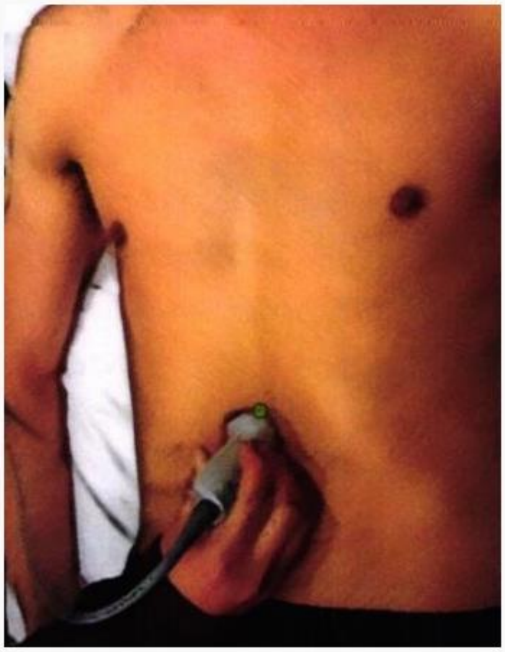

Hold probe with marker cephalad

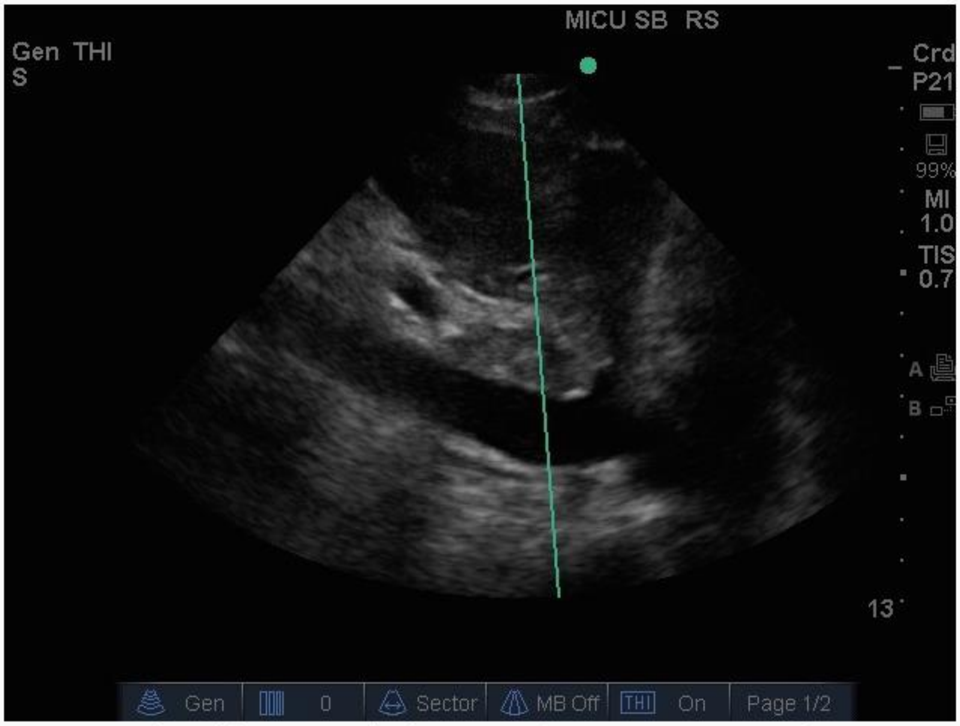

Image 1: M-Mode with beam 2-4 cm distal to cavo-atrial junction just posterior to hepatic vein

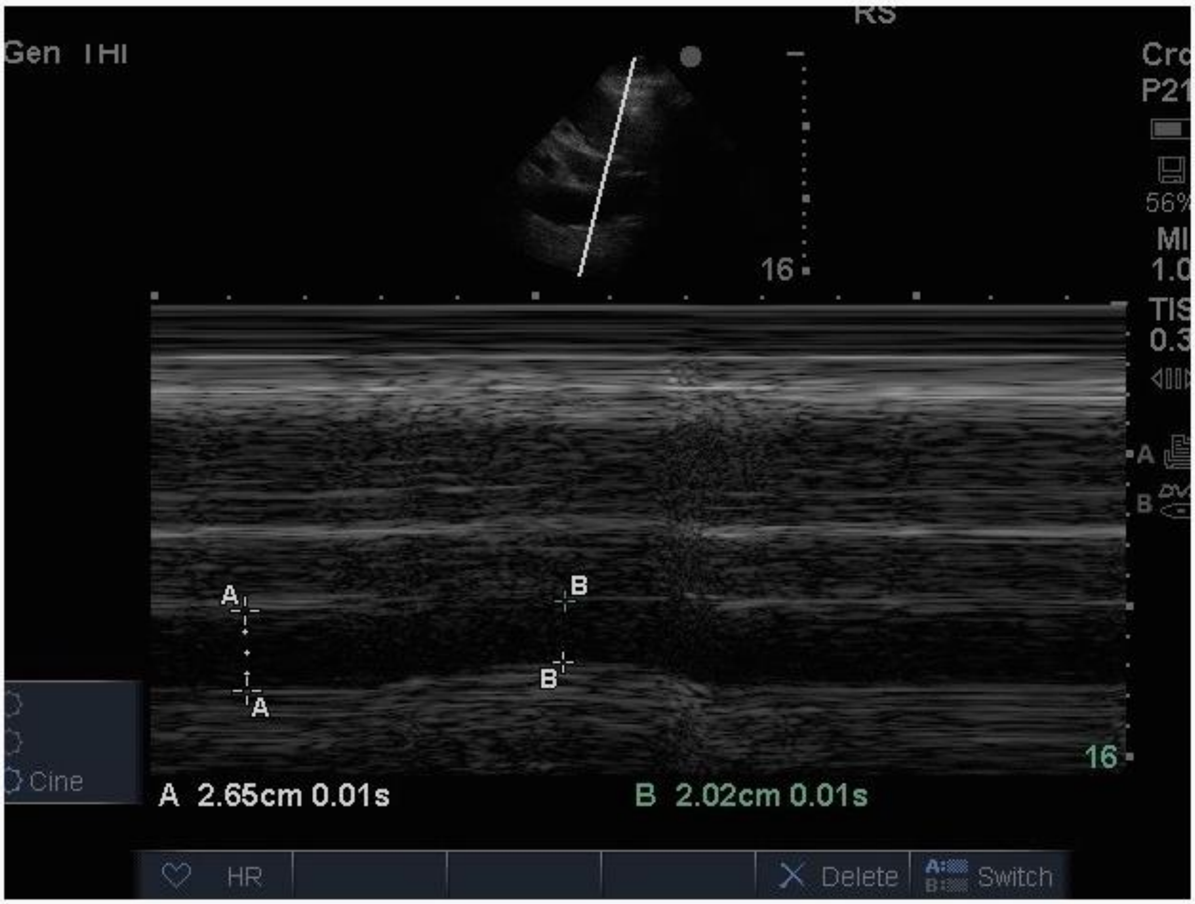

Image 2: Measure D-min and D-max with calipers

Interpretation and clinical application

- For non-intubated patients record the Dmax or calculate the cIVC.
- For mechanically ventilated patients calculate the dIVC.

Non-Intubated patients:

In non-intubated patients maximum IVC diameters < 1cm or cIVC values > 40% have been shown indicate fluid responsivity, meaning that the infusion of fluids will lead to an increase in cardiac output. However, maximum diameters >1cm or cIVC values <40% do not exclude fluid responsiveness and thus the decision to administer fluids vs. pressors must be based on a more comprehensive clinical picture of the patient [4,5].

Mechanically Ventilated Patients:

Because the physiology of mechanically ventilated patients is more controlled than their non-intubated counterparts, the distinction between fluid responsivity and non-responsivity is more

clearly cut. In these patients dIVC values >18% have been shown to be both 90% sensitive and 90% specific for fluid responsivity, whereas dIVC values <18% indicate a low probability of fluid responsivity, and thus one should pursue the administration of pressors/inotropes [2].

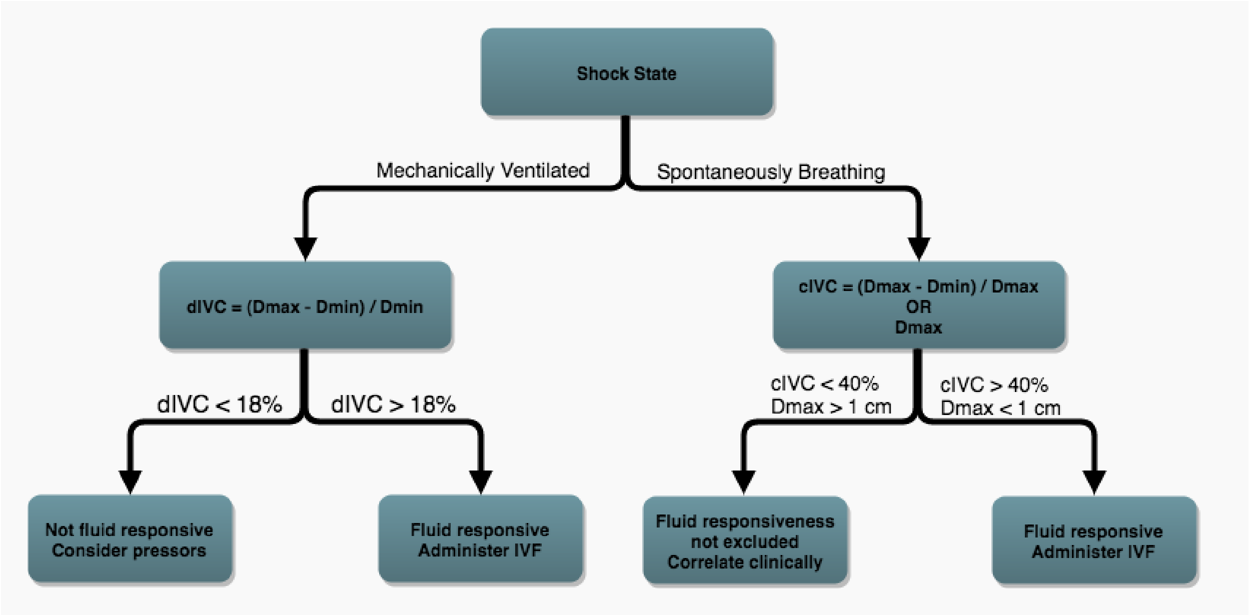

Examples

<u>Case 1a: Mechanically Ventilated Patient</u>

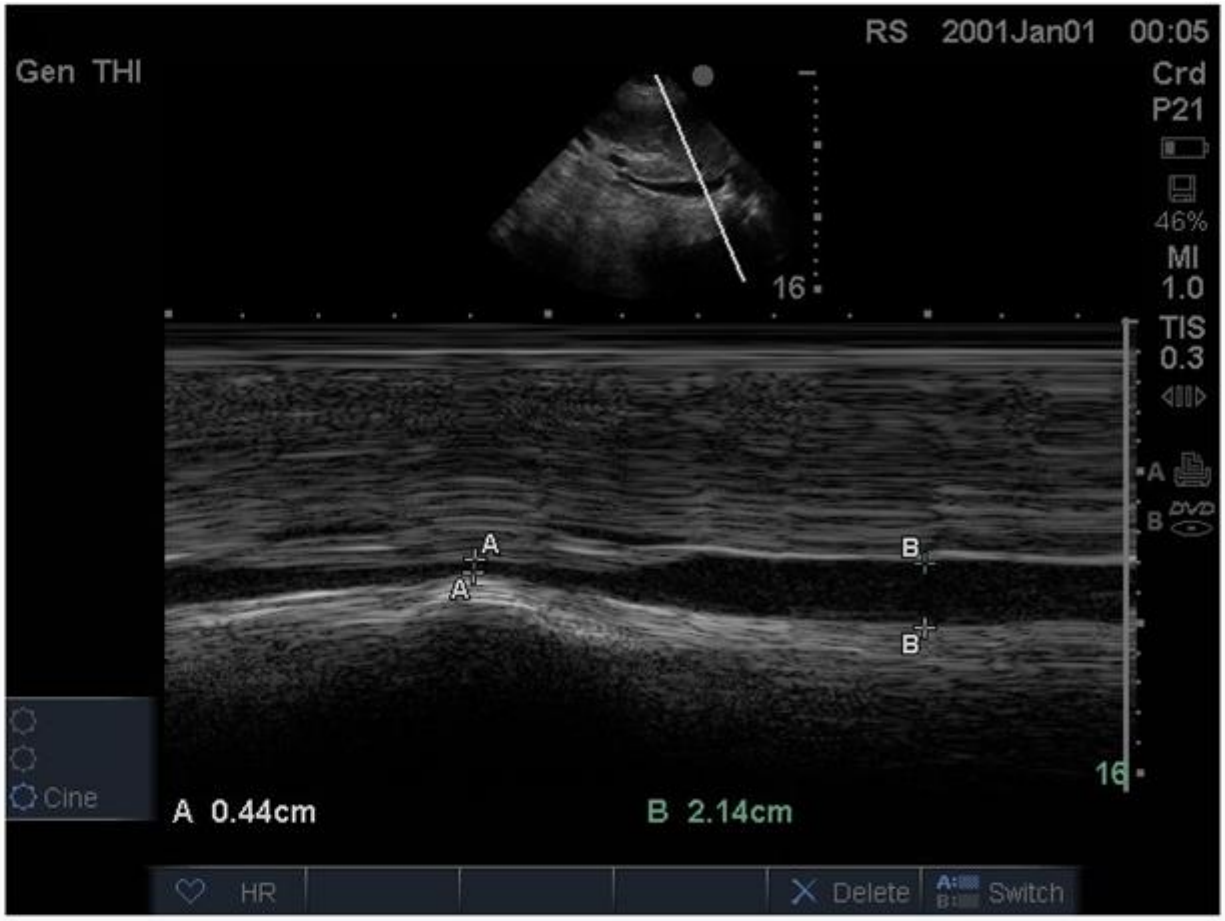

1A

dIVC = (2.14-0.44)/0.44 = 386%

dIVC> 18%: pt is fluid responsive, begin fluid resuscitation

Case 1b: Mechanically Ventilated Patient

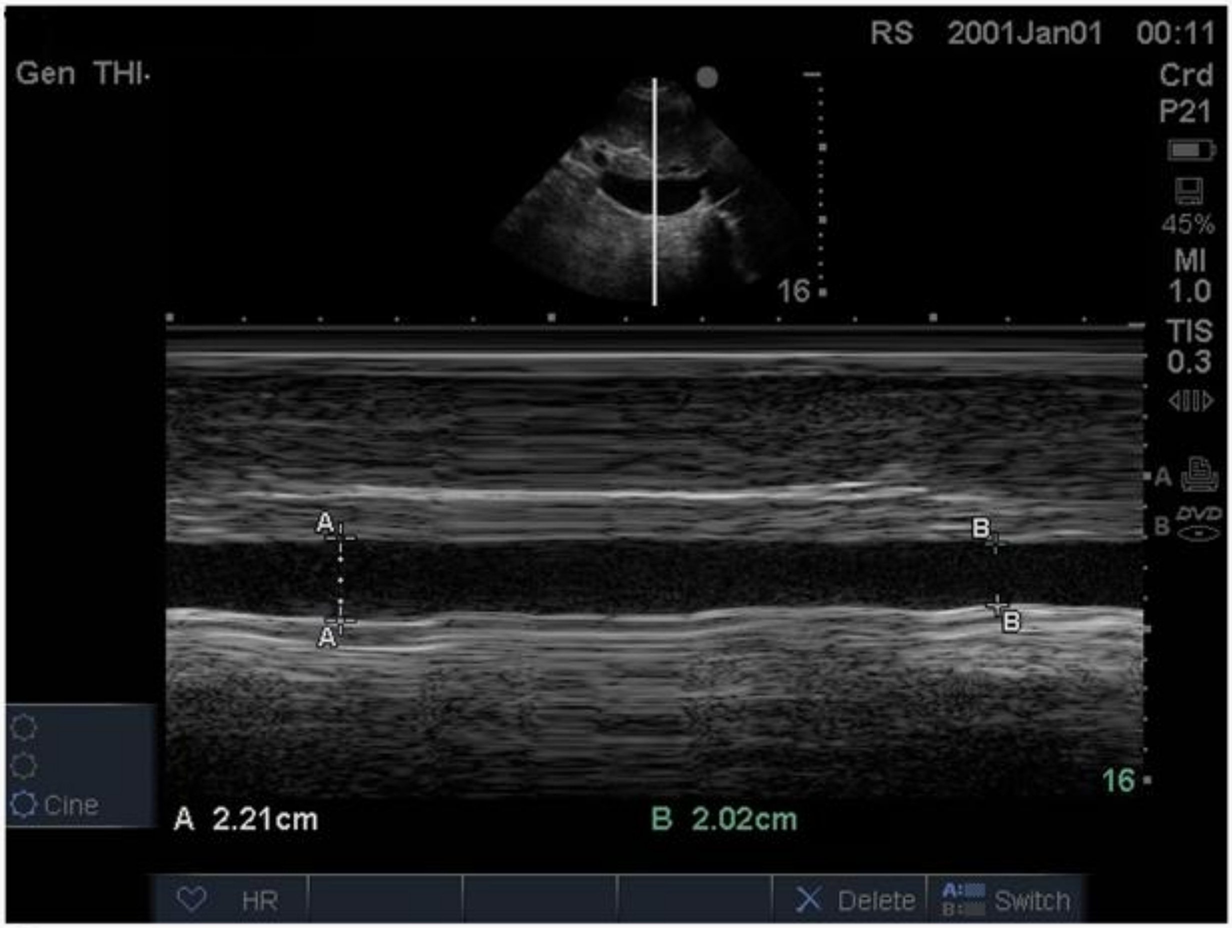

1B

dIVC = (2.21-2.02)/2.02 = 9%

dIVC<18%: pt is not fluid responsive: begin pressors

Case 2a: Non-Intubated Patient

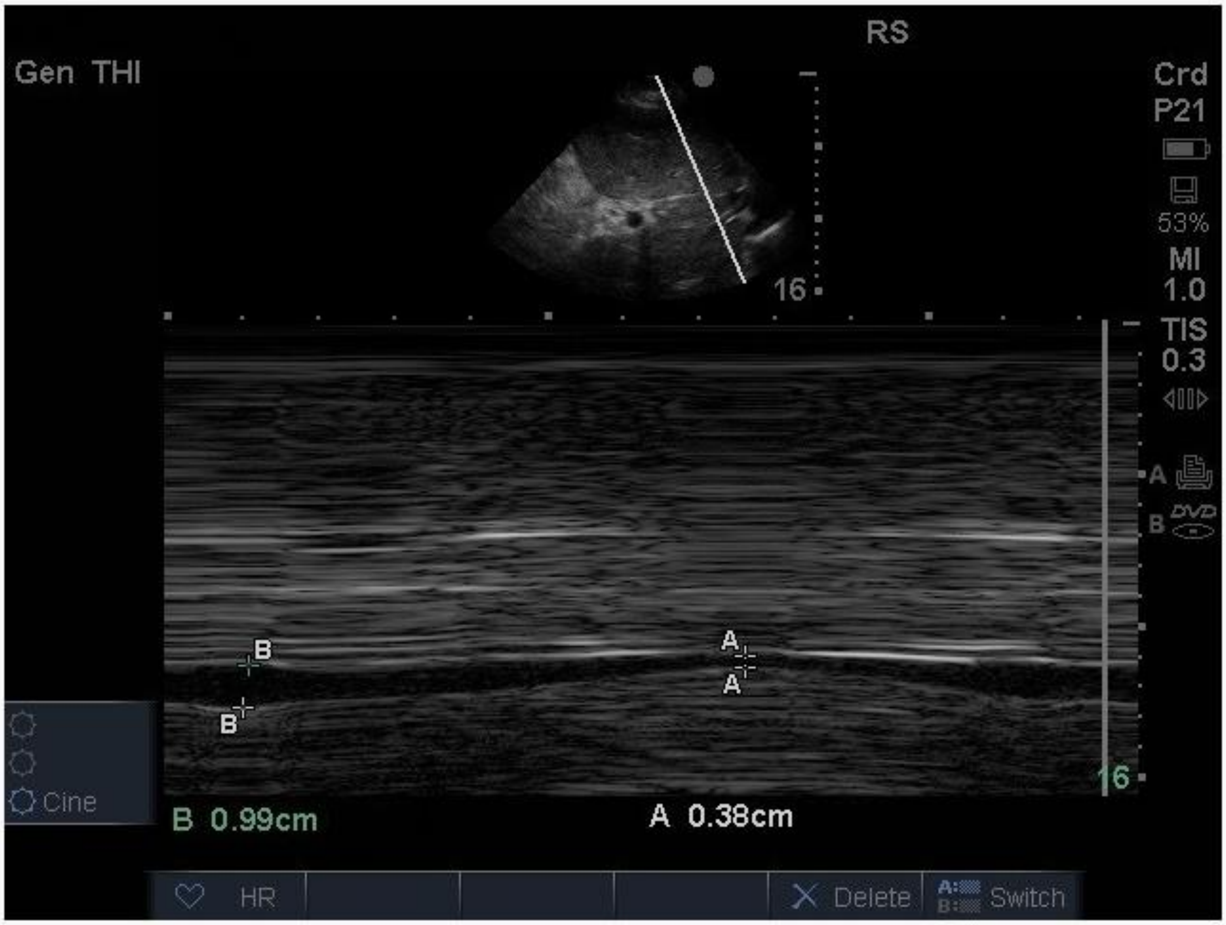

2A

cIVC = (0.99-0.38)/0.99 = 62%

CIVC > 40% or Dmax<1cm: pt is fluid responsive: begin fluid resuscitation

Case 2b: Non-Intubated Patient

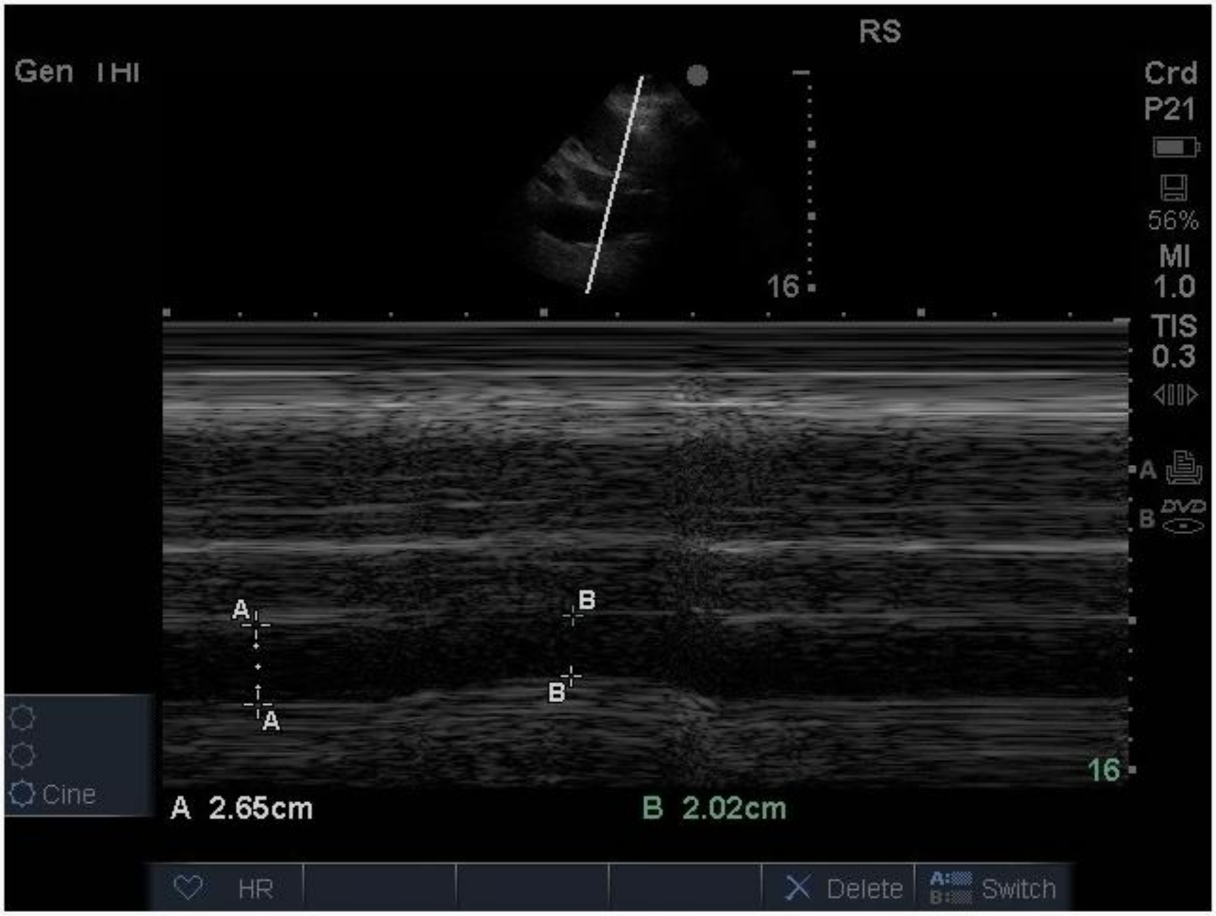

2B

cIVC = (2.65-2.02)/2.65 = 24%

cIVC<40% or Dmax>1cm: pt unlikely fluid responsive: correlate clinically and consider pressors

Pearls and Pitfalls

- These concepts apply only to patients in the shock state.
- Be aware of the criteria that the patient has to meet before can use these calculations.
- If patient does not meet all criteria, can still use extreme findings, such as minimal to no respiratory variability, 100% variability, an unmeasurably small IVC or a large IVC caliber (approximately 2.5 cm)
- Use the phased array probe in the cardiac setting
- Use anterior (subcostal) view as described in this document; if anterior abdomen not obtainable can look at the IVC from the right- lateral view however the calculations are unreliable.
- Measure the IVC just past the hepatic vein which is commonly seen entering IVC just by the cavo- atrial junction.
- Non Intubated patients → cIVC, or Dmax
- cIVC> 40%, or Dmax<1cm: administer fluids cIVC< 40%, or Dmax>1cm: clinical correlation! (Use of lung US may help guide the decision)
- Mechanically vented patients → dIVC
- dIVC = (Dmax – Dmin)/Dmin
- dIVC>18% → administer fluids
- dIVC< 18% → administer pressors
- If close to 18% correlate clinically, consider starting both fluids and pressors.
- Do not mistake aorta for IVC: be sure to identify both vessels from that anterior (subcostal) view

References

1. Robbins, S.L. and V. Kumar, *Robbins and Cotran pathologic basis of disease*. 8th ed. 2010, Philadelphia, PA: Saunders/Elsevier. xiv, 1450 p.
2. Barbier, C., et al., *Respiratory changes in inferior vena cava diameter are helpful in predicting fluid responsiveness in ventilated septic patients.* Intensive Care Med, 2004. **30**(9): p. 1740-6.
3. Daniel De Backer, B.P.C., Michel Slama, Antoine Vieillard-Baron, PhilipeVignon, ed. *Hemodynamic Monitoring Using Echocardiography in the Critically Ill*. 2011, Springer.
4. Muller, L., et al., *Respiratory variations of inferior vena cava diameter to predict fluid responsiveness in spontaneously breathing patients with acute circulatory failure: need for a cautious use.* Crit Care, 2012. **16**(5): p. R188.
5. Ahmad, S., *Stony Brook Critical Care Ultrasound*. 2014.

Day 3

Chest- Critical Care Echo

Vlad Radulescu, MD; Roshini Malaney, DO; and Sahar Ahmad, MD

Introduction

Echocardiography is a fast and effective way to evaluate basic cardiac function in an unstable patient. The information obtained from a bedside echo can be used in conjunction with the patient's clinical status to make real time decisions and guide management. In contrast, the complete echo that is done by a technician and interpreted by a cardiologist is usually done when a patient is stable with the interpreter removed from the clinical scenario. The process of a complete echo is also time consuming and can delay critical decision-making.

View	Identify	Transducer & Probe Marker	Machine Setting Screen Marker	Location	Image
PSLA	RV, LV, aortic root, aortic valve, mitral valve, pericardium	Low frequency<br>Phased array<br>Marker towards patient's right supraclavicular fossa	Cardiac setting<br>Operator right	Parasternal left, 3rd or 4th intercostal space	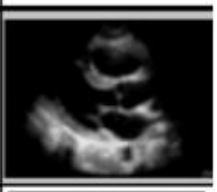
PSSA	RV, interventricular septic, LV, papillary muscles, chordae, inferior wall LV, anterior wall LV, pericardium	Low frequency<br>Phased array<br>Marker toward left shoulder	Cardiac setting<br>Operator right	Parasternal left, 3rd or 4th intercostal space	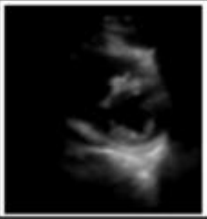
A4C	LV, RV, LA, RA, mitral valve, tricuspid valve	Low frequency<br>Phased array<br>Marker towards left shoulder	Cardiac setting<br>Operator right	Lateral or inferior to left nipple, at the PMI (varies by patient and position)	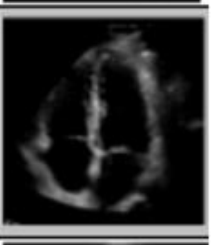
Subcostal	RV, LV, LA, Ra, mitral valve, tricuspid valve, pericardium	Low frequency<br>Phased array<br>Marker facing patient's left	Cardiac setting<br>Operator right	2-3 cm below the xiphoid process	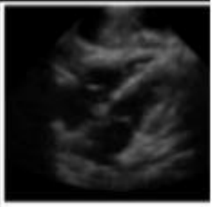
IVC	IVC, hepatic vein, RA	Low frequency<br>Phased array<br>Marker facing cephalad	Cardiac setting<br>Operator right	2-3 cm below the xiphoid process	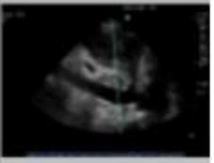

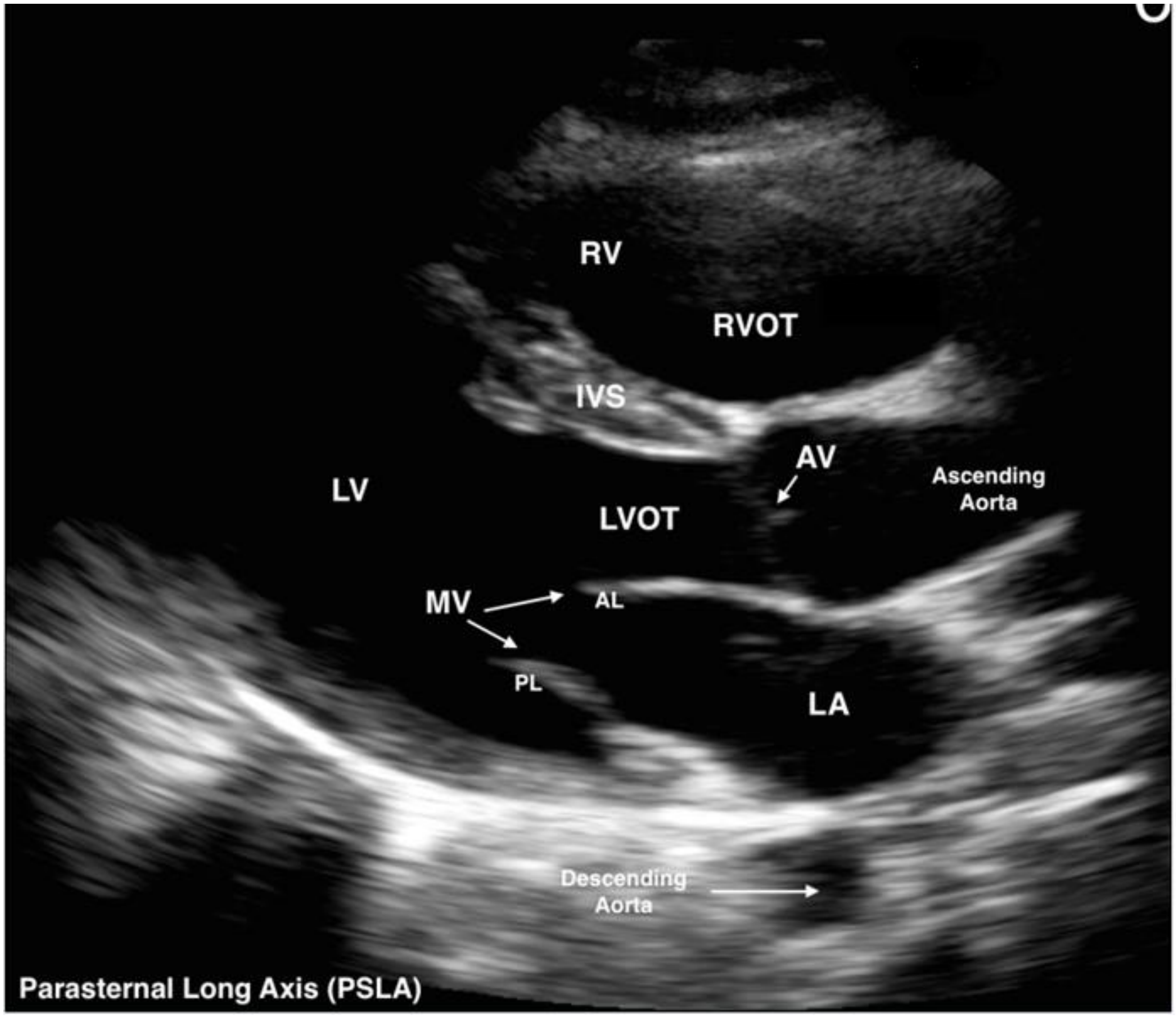
RV
RVOT
IVS
AV
Ascending Aorta
LV
LVOT
MV
AL
PL
LA
Descending Aorta
Parasternal Long Axis (PSLA)

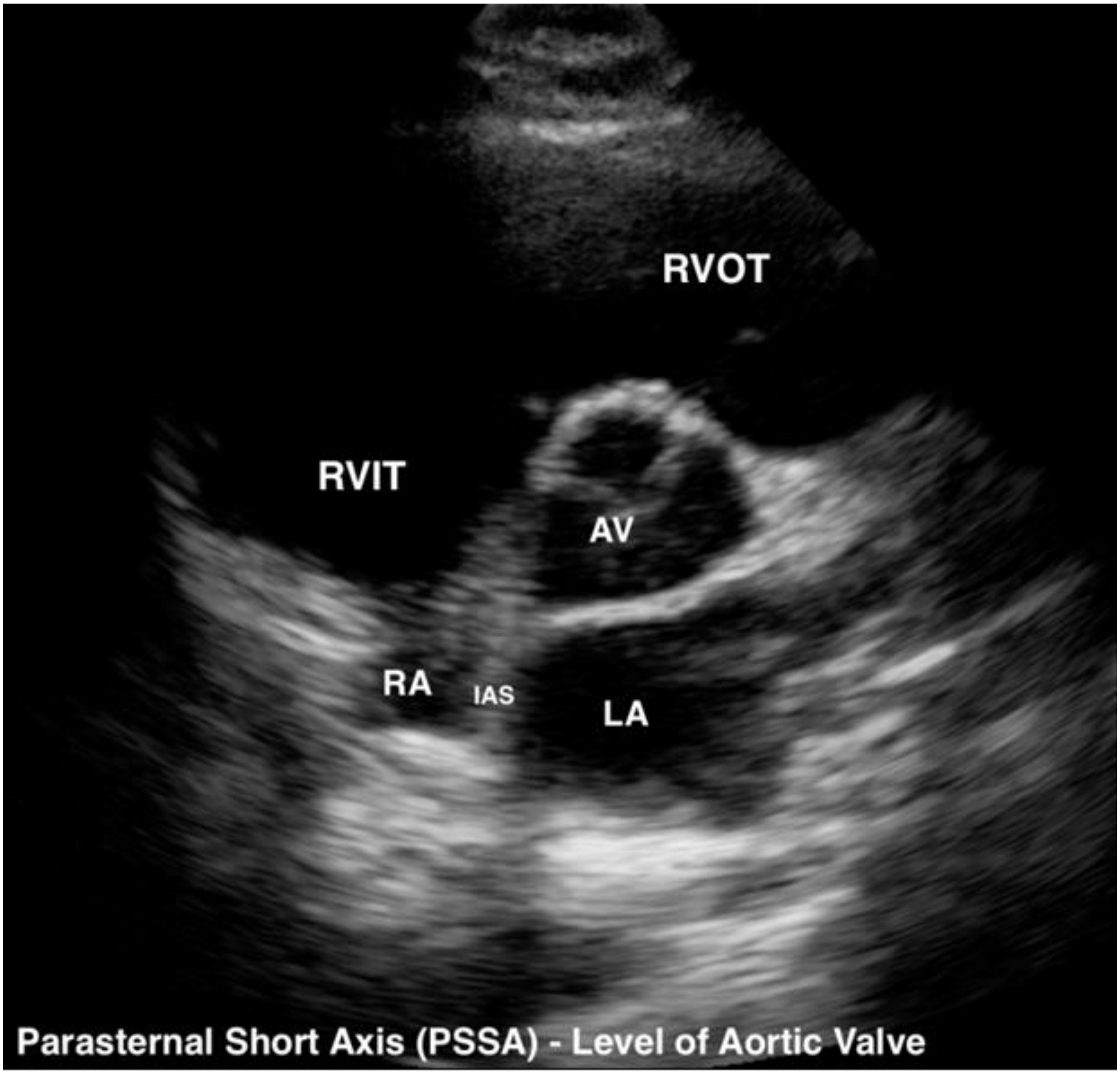
RVOT
RVIT
AV
RA
IAS
LA
Parasternal Short Axis (PSSA) - Level of Aortic Valve

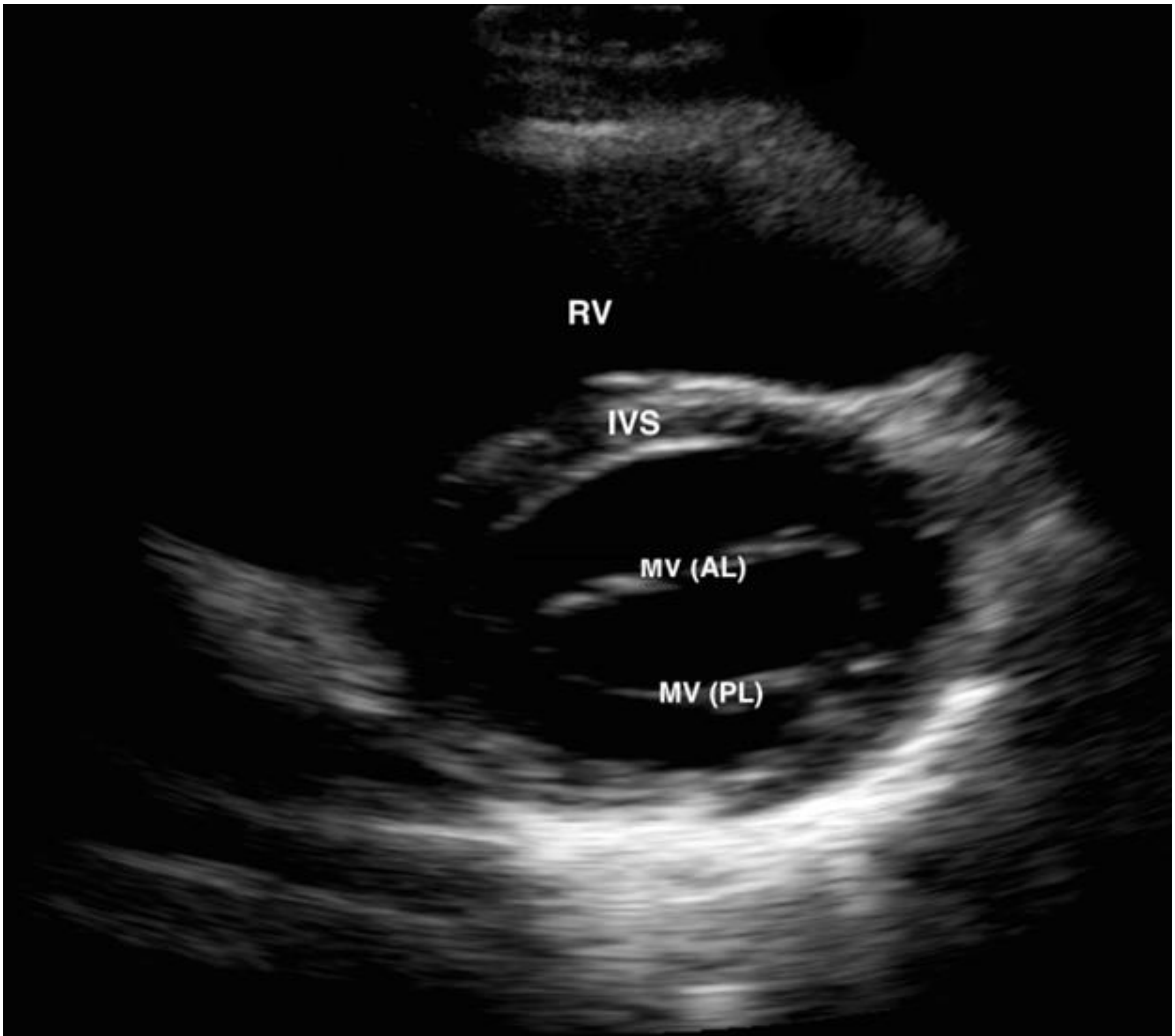

Parasternal Short Axis (PSSA) - Level of Mitral Valve

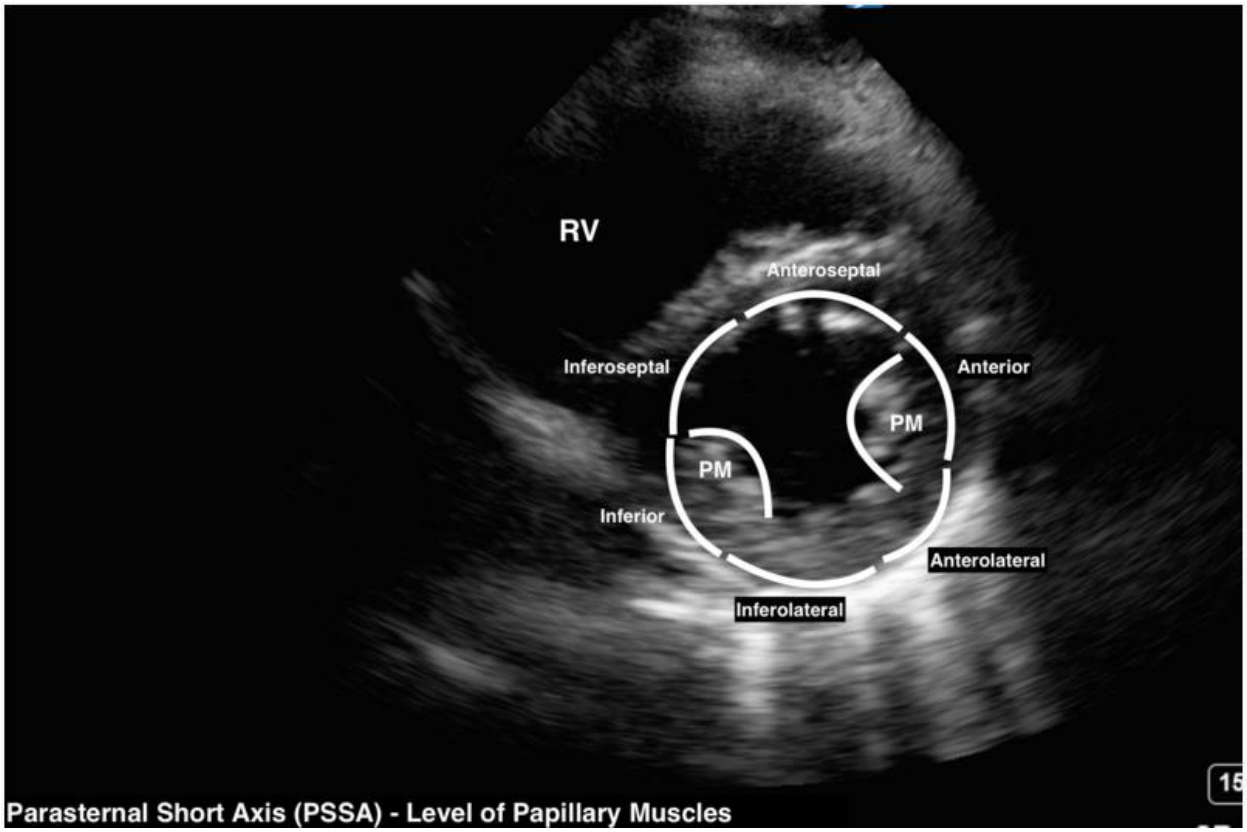

Parasternal Short Axis (PSSA) - Level of Papillary Muscles

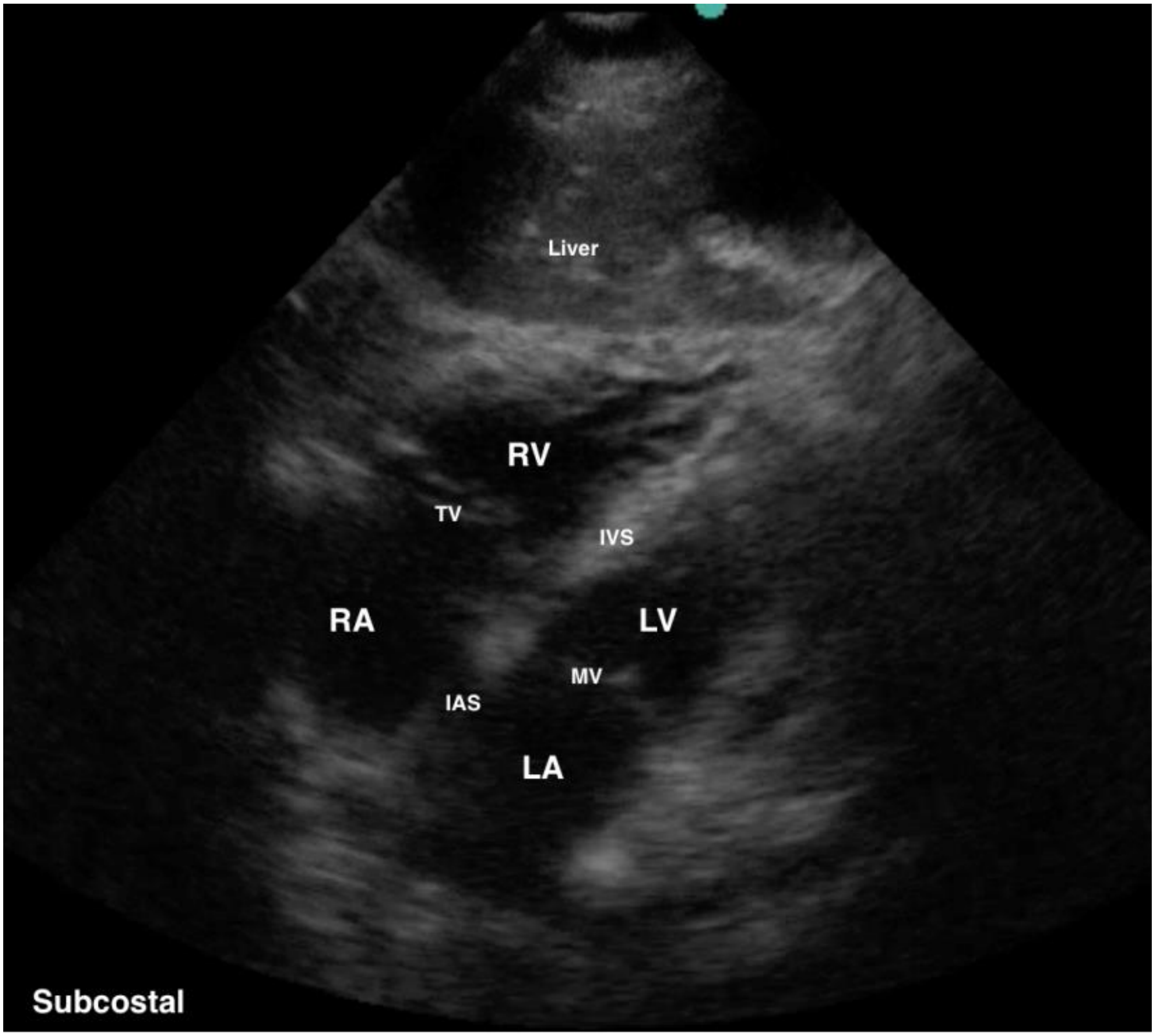

Liver
RV
TV
IVS
RA
LV
MV
IAS
LA
Subcostal

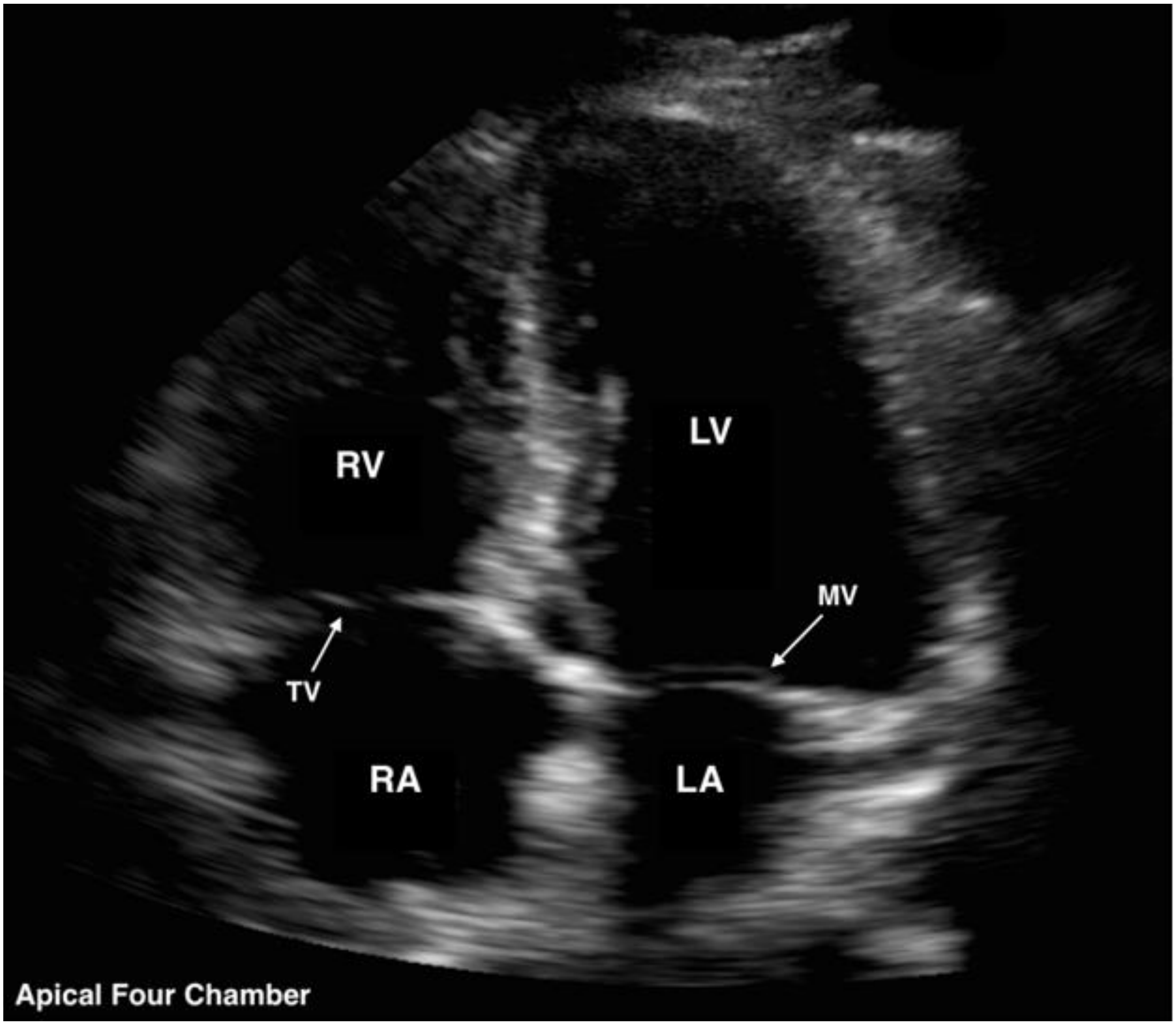

Pearls and Pitfalls

- PSLA: The mitral valve should be positioned at the center of the field with LV horizontally across the screen.
- PSSA: The papillary muscles should be symmetric.
- A4C: The ventricles should be elongated.
- A4C, subcostal view: The leaflets of the mitral and tricuspid valve should be visible.

- In patients with COPD, the PSLA and PSSA views are obstructed due to the presence of retrosternal air. Hyperinflation also causes the heart to be pushed distally toward the abdomen making the subcostal view more ideal and attainable in these patients.
- The parasternal view can sometimes be obscured by air artifact. In cooperative patients, instructing the patient to exhale and hold may minimize this. In ventilated patients, an expiratory hold can be used to create the same effect.
- LV function cannot be assessed within a single scan plane. It functions in multiple planes and at least two views are needed in ordered to determine function. This is also true for pericardial effusions, which can be mimicked by ascites in the subcostal view [1-8].
- A4C – most challenging view

Three methods to obtain an Apical 4 Chamber view

1. Put-Down Method – place the probe directly over the apical impulse (inferior lateral margin of the pectoralis muscle)
2. Migrate Method – from the PSSA view, migrate the probe towards the inferior lateral margin of the pectoralis muscle while keeping some image of the heart on the center of the screen. Once at or beyond the nipple line and the image has reached a view of the LV apex, angulate the probe such that the scan plane views anteriorly and the tail of the probe moves towards the ground.
3. Spleen-Up Method – identify the spleen along the left mid-axillary plane and migrate the probe cephalad towards the apical impulse

References

1. Via, G., et al., International evidence-based recommendations for focused cardiac ultrasound. J Am SocEchocardiogr, 2014. 27(7): p. 683 e1-683 e33.
2. Narasimhan, M., S.J. Koenig, and P.H. Mayo, Advanced echocardiography for the critical care physician: part 2. Chest, 2014. 145(1): p. 135-42.
3. Mayo, P.H., Critical care ultrasonography: the Italian approach. Intensive Care Med, 2013. 39(10): p. 1849-50.
4. Mayo, P.H., Training in critical care echocardiography. Ann Intensive Care, 2011. 1: p. 36.
5. Labovitz, A.J., et al., Focused cardiac ultrasound in the emergent setting: a consensus statement of the American Society of Echocardiography and American College of Emergency Physicians. J Am SocEchocardiogr, 2010. 23(12): p. 1225-30.
6. Kaplan, A. and P.H. Mayo, Echocardiography performed by the pulmonary/critical care medicine physician. Chest, 2009. 135(2): p. 529-35.
7. Marik, P.E. and P. Mayo, Certification and training in critical care ultrasound. Intensive Care Med, 2008. 34(2): p. 215-7.
8. Anne-Sophie Beraud, M., Toni Burkett. Introduction to transthoracic echocardiography. Available from:http://www.acep.org/uploadedFiles/ACEP/Clinical_and_Practice_Management/innovatED/Innovation/Philips Healthcare - Intro to TTE Tutorial_short_FNL.PDF

Day 4

Cardiac Arrest- Ultrasound for Cardiac Arrest

Shreyas Ravishankar, MD and Sahar Ahmad, MD

Introduction

Management of patients in cardiac arrest starts with basics of circulation, airway and breathing. Analysis of cardiac rhythm and the administration of drugs and defibrillation is an integral part of advanced cardiac life support (ACLS). After these initial management goals are met, it is important to identify and treat specific reversible causes of cardiac arrest during the early resuscitation period. These reversible causes are often summarized as the "Hs and Ts."

<u>Hs &Ts</u>

H: Hypovolemia, Hypoxia, Hydrogen ion (acidosis), Hyperkalemia, Hypothermia

T: Toxins, Tamponade, Tension pneumothorax, Thrombosis (coronary and pulmonary)

This search for reversible causes takes place through a targeted review of history and lab work along with bedside ultrasound. We will describe a focused ultrasound protocol for this purpose.

Ultrasound

The benefit of ultrasound in cardiac arrest is the ability to quickly look for many of these reversible causes at bedside during the arrest. What follows is an algorithm for a systematic approach to quickly assess for many of these causes. Critical management decisions can be made with information from this protocol including: The identification of tension pneumothorax, intra-abdominal or retroperitoneal bleeding, volume depletion, right ventricular failure, and cardiac tamponade. Airway management can be guided with verification of endotracheal tube placement and efficient ventilation with the benefit that ultrasound findings are not hindered by ambient noise and the efficiency to identify a misplaced endotracheal tube without the delay of X-ray imaging. Performing echocardiography during the resuscitation process can determine fine ventricular fibrillation, which on rhythm analysis appears as asystole (two very different management decisions here: fine V-fib needs defibrillation, whereas for asystole, we administer epinephrine or vasopressin), or to visualize return of spontaneous circulation (ROSC). Intra-arrest echocardiography is also considered by many authors to hold predictive value for the likelihood of achieving ROSC. In the post-arrest management period, lung, IVC and cardiac ultrasound will guide administration of fluids, vasopressors and inotropes. With practice, it should be possible to perform these maneuvers rapidly and obtain valuable information for management decisions in a reliable and efficient manner. Any positive findings must immediately be communicated to the Code Team Leader.

Tension Pneumothorax

The first thing to do is to check for pneumothorax. This can either be done with the high frequency linear array *or* the low frequency phased array. Check in 2–3 rib spaces bilaterally in the least dependent portions (the anterior lung fields in a supine patient). The presence of lung sliding and/or B-lines in the anterior chest wall (see Chest/Lung ultrasound chapter for definitions) will immediately rule out a hemodynamically significant pneumothorax. If you see a lack of lung sliding at any location (or "stratosphere sign" in M-mode), pneumothorax should be considered.

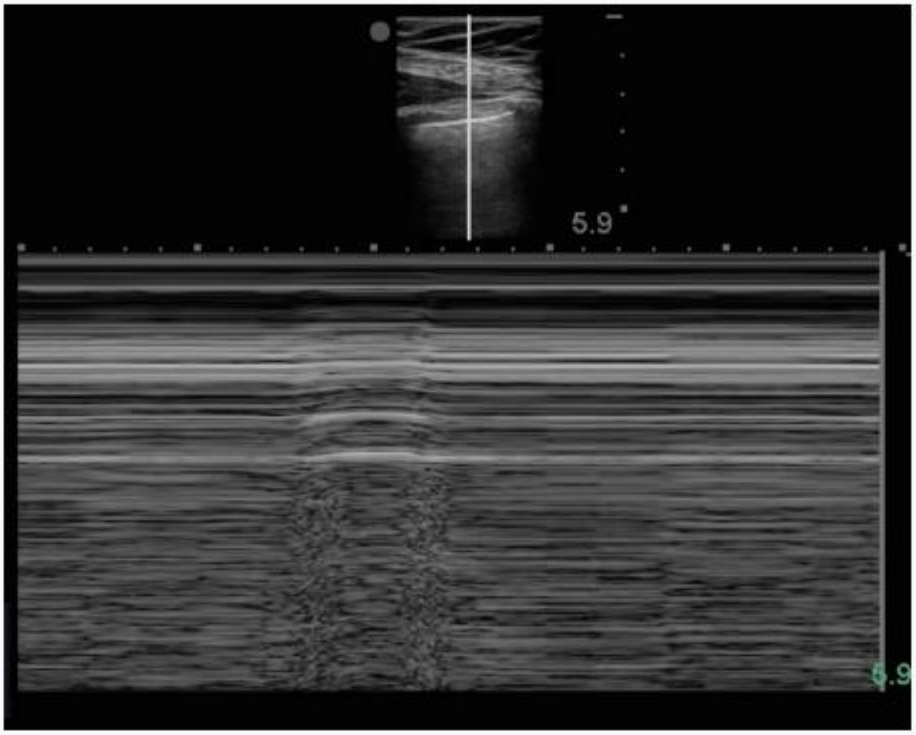

Rule out PTX (absent lung sliding or bar-code sign (stratosphere sign)

Hypovolemia & Hemorrhage

There are multiple causes for hypovolemia. One of the easily assessed is hypovolemia due to hemorrhage. Using the low frequency phased array with the marker pointed cephalad, look for the kidney on the right side. You should be able to see the hepatorenal recess and check for an anechoic collection, which could represent blood or ascites.

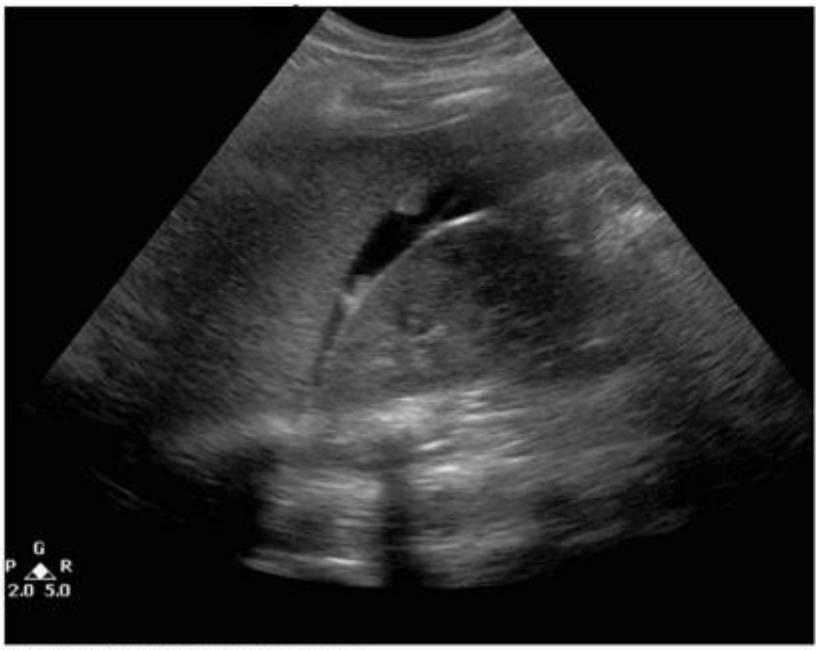

Rule out hemorrhage (collection at hepato-renal recess)

While in the abdomen, it is useful to also check for inferior vena cava (IVC) diameter. This can be useful to evaluate intravascular volume status. An absolute dimension of IVC of <1 cm can be a useful indicator of intravascular volume depletion in the setting of cardiac arrest. A diameter of 2.5cm or larger suggests against the need for immediate volume expansion.

Thrombosis and Tamponade

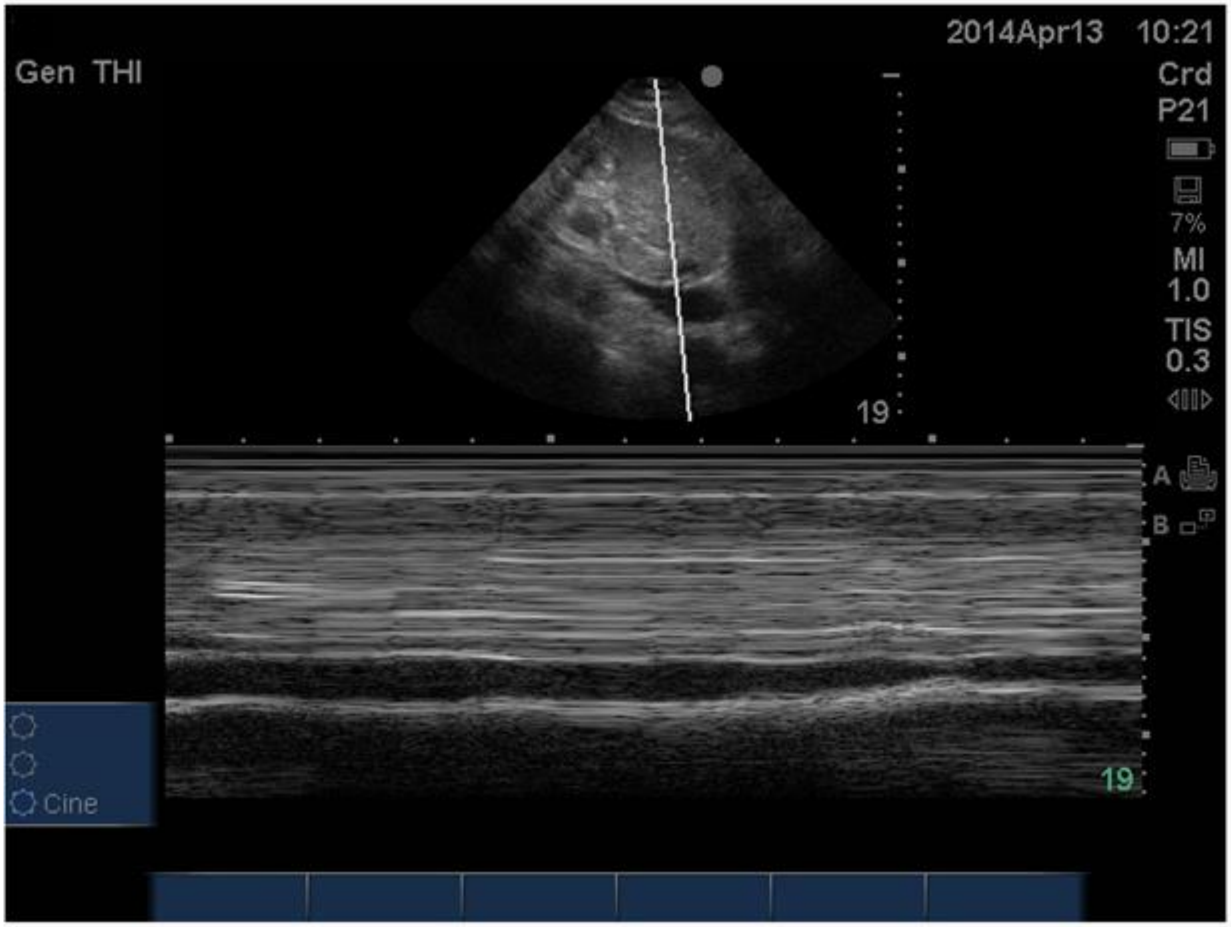

Rule out hypovolemia (IVC < 1 cm)

Using the low frequency phased array probe, look at the heart using a subcostal 4-chamber view. You should be able to see if RV is significantly larger than the LV, which could be evidence for pulmonary embolus. You can also look for evidence of cardiac tamponade.

Cardiac views can also be used during the code to assess cardiac function. Evidence of cardiac standstill would be a sign of poor overall outcome. One can also see poor contractility consistent with ventricular fibrillation, which may be useful in determining whether the rhythm is shockable or not. Evidence of poor LV contractility or regional wall motion abnormalities may be useful when deciding whether the etiology of the arrest is purely cardiac in nature, and may help in determining whether the patient should undergo an emergent cardiac catheterization. All echo views should be obtained during the “pulse check” phase of ACLS.

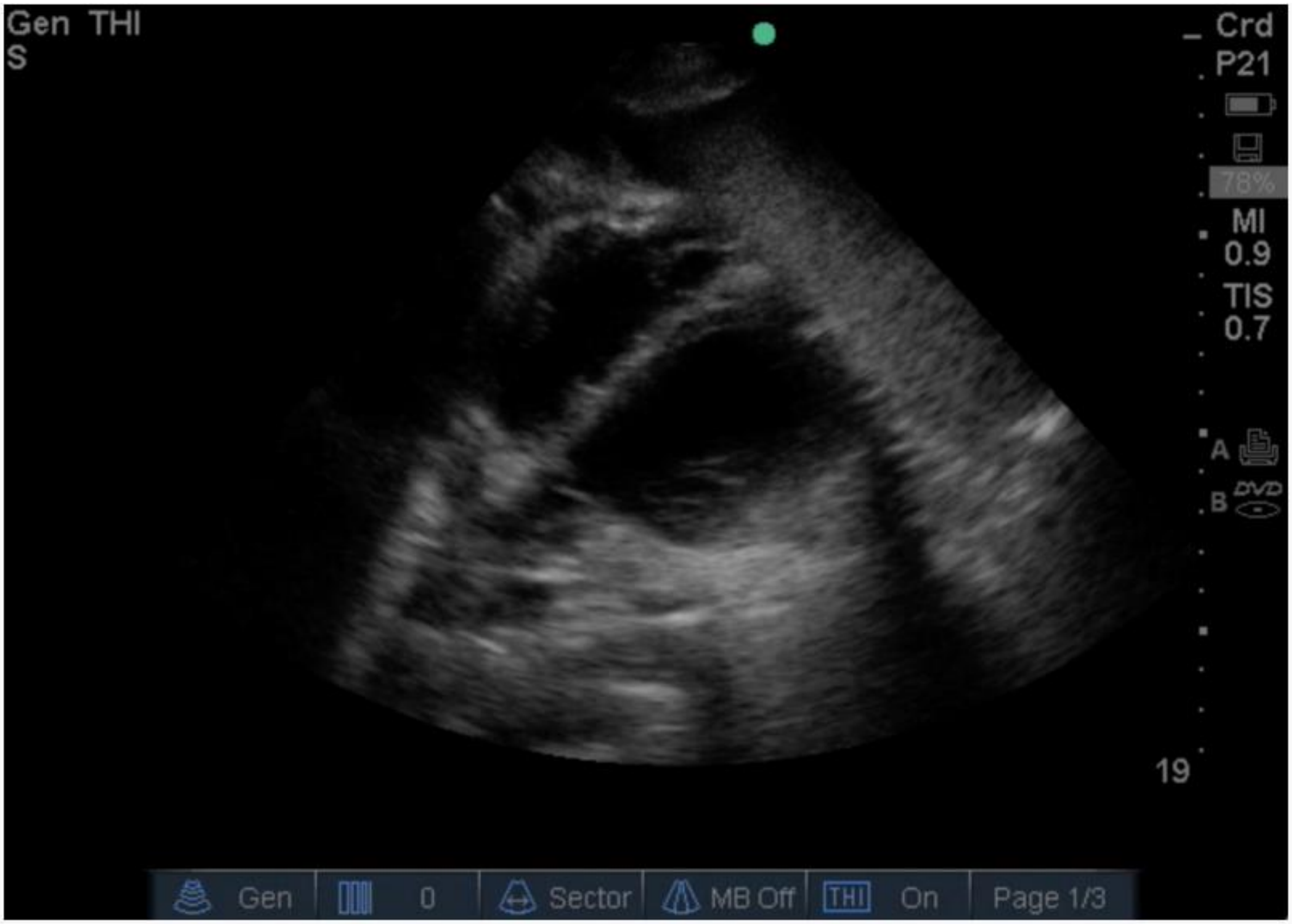

Rule out acute cor pulmonale (RV:LV > 1)

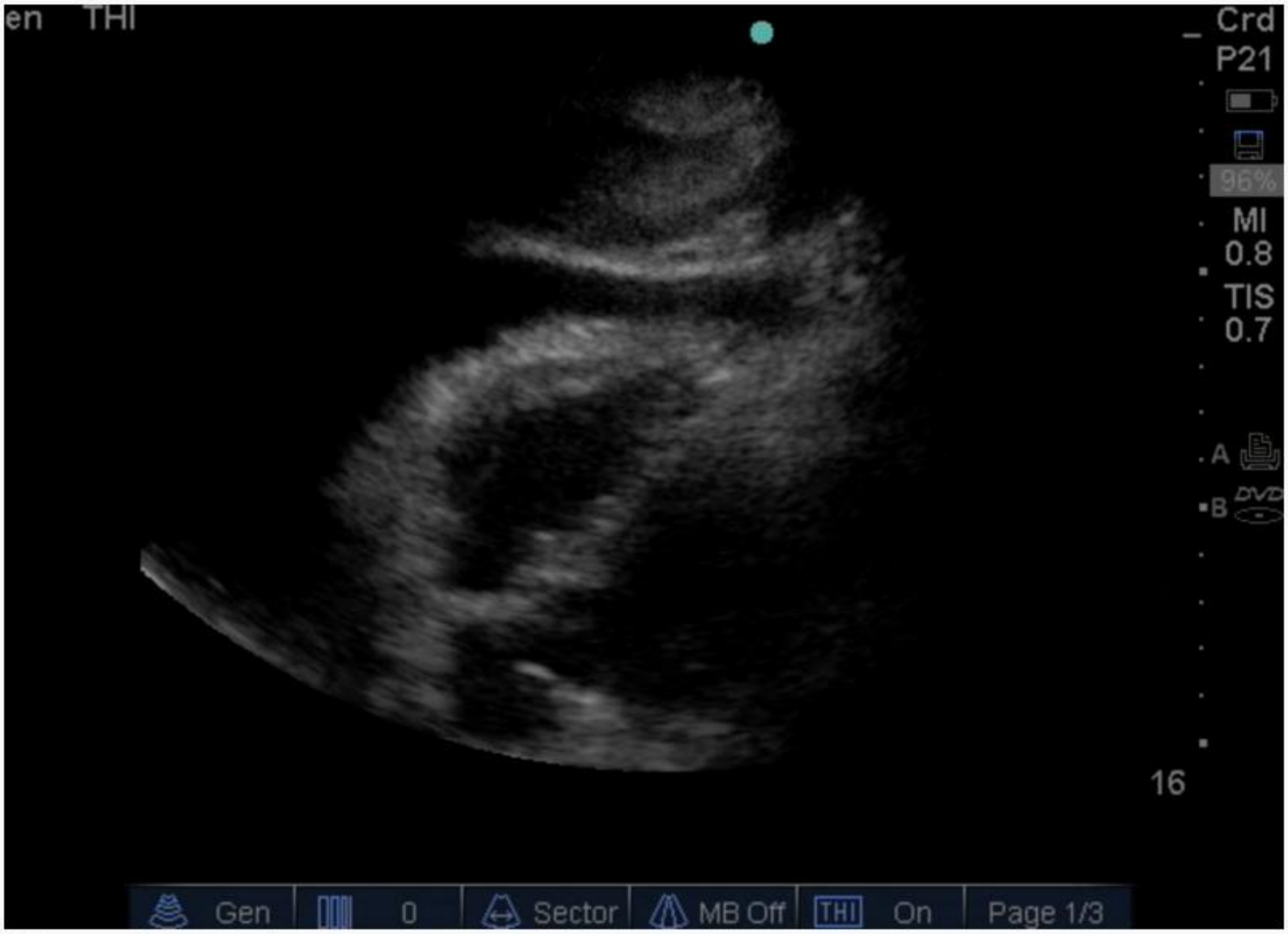

Rule out cardiac tamponade

ETT Placement

Intubation during a cardiac arrest is challenging and has to be performed in synchrony with a ten-second pulse check. Esophageal intubation, due to these challenges, is not uncommon and can be a reason why ROSC is delayed. Therefore, after intubation, it is important to confirm that the endotracheal tube is in the airway, rather than in the esophagus. Capnography can initially be unreliable, as ambu-bagging prior to intubation has usually insufflated the stomach, and auscultation for lung sounds can be unreliable in noisy environments, especially in the presence of automated CPR. Ultrasound technique for airway verification does not have these limitations. A quick way to confirm proper placement is to use the high frequency linear array probe and place it at the neck. You should be able to see a single air shadow in the anterior neck, which represents the trachea (with the endotracheal tube within; the presence of the ETT does not change the trachea's air shadow because the ETT is much smaller diameter). Normally the esophagus, which is typically flat and not air-filled, will not be visible. If, on the other hand, the endotracheal tube is in the esophagus, you will see two air shadows in the anterior neck, which represent both the trachea and the ETT in the esophagus. The linear array high frequency transducer should then be placed at the anterior lung fields to look for lung sliding (or movement of B-lines). The shimmering of lung sliding (and/or the movement of the B-lines when present)

should correspond to the ambu-bag ventilation. If two air shadows at the neck and an absence of lung sliding/ B-line movement with ambu-bag are detected, in appropriate clinical condition (especially inability to achieve ROSC and low EtCO2), removing the ETT, returning to ambu-bag, and re-intubating when appropriate should be discussed with the Code Leader and Airway Team. All this can take place without interruption of ACLS for CXR.

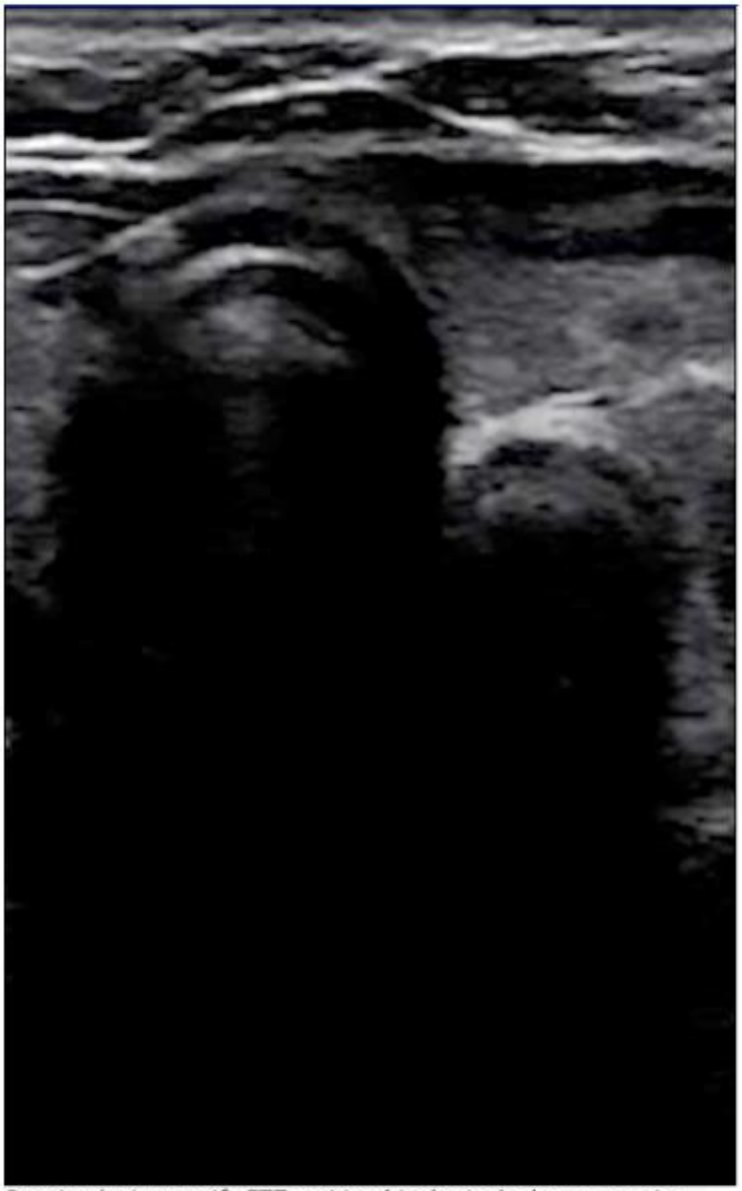

Post-intubation: verify ETT position (single air shadow at anterior neck). Image here shows esophageal intubation (double air column).

Pearls and Pitfalls

- B-lines are visceral pleural artifacts that, if seen, rules out pneumothorax
- The absence of lung sliding does not always mean there is a tension pneumothorax. Other possibilities include right mainstem intubation or inadequate intubation.
- The presence of lung pulse can differentiate between right mainstem intubation and pneumothorax during ROSC. Lung pulse is the shimmering effect of the pleural surface in response to transmitted cardiac motion. A right mainstem intubation will display lung sliding at the right anterior chest and lung pulse at the left anterior chest.

<u>Referenes</u>

1. Michael B. Stone, MD; Wilma Chan, MD;
Focused ultrasound for airway management, Philips tutorial. (ACEP.org)*http://www.acep.org/uploadedFiles/ACEP/Clinical_and_Practice_Management/innovatED/Innovation/Philips%20Healthcare%20-%20Airway_Tutorial_FNL_short.pdf*

Shock- Approach to Undifferentiated Shock

Glen Granati, MD; Craig Fryman, MD; and Sahar Ahmad, MD

Introduction

Shock states are categorized into four major categories - hypovolemic, cardiogenic, obstructive, and distributive. Each form of shock may present similarly (i.e., hypotension and end-organ damage); however, management strategies of each type are distinct from one another. Ultrasound (US) provides a method to quickly and accurately differentiate shock types, and in the right hands leads to more efficient management of patients in shock.

This chapter describes US findings associated with each type of shock. Through pairing of signs, symptoms, and sonographic findings, the medical resident can better evaluate the etiology of or *contributing factors to shock state.*

View	Cardiogenic	Obstructive			Hypovolemic		Septic
		Tampondade	Pneumothorax	Cor Pulmonale	Third Spacing	Hemorrhahic	
Internal Jugular	Distended IVC with minimal respiratory variation				Collapsed IVC with high respiratory variation		
Heart	Hypocontractile<br>Poor MV excursion<br>Enlarged LV<br>Wall motion abnormalities	Percardial effusion<br>RA or RV collapse		Enlarged RV<br>Septal bowing (D-Sign)<br>McConnel's sign<br>Right-sided thrombus	Hypercontractile		Hypercontractile (early)<br>Hypocontractile (late)
Lungs and Pleura	B-lines		Absent lung sliding<br>Lung point<br>Lack of B-lines		Pleural effusions	A-lines	A-lines (initially)
Miscellaneous				Positive DVT study	Ascites	Fluid in RUQ/LUQ<br>Aorta >5cm<br>(Ruptured AAA)	

Ultrasound in Cardiogenic Shock

All of the following views utilize (unless otherwise stated) these standards: a phased array low-frequency transducer probe and machine set to cardiac setting with screen marker Operator - Right.

Left ventricular (LV) function may be rapidly assessed with a PSLA view. Normal LV function is characterized by adequate excursion of the mitral leaflets (discussed in more detail below), an approximately 40% thickening of the LV wall during systole, and a 30% reduction in LV cavity size during systole.

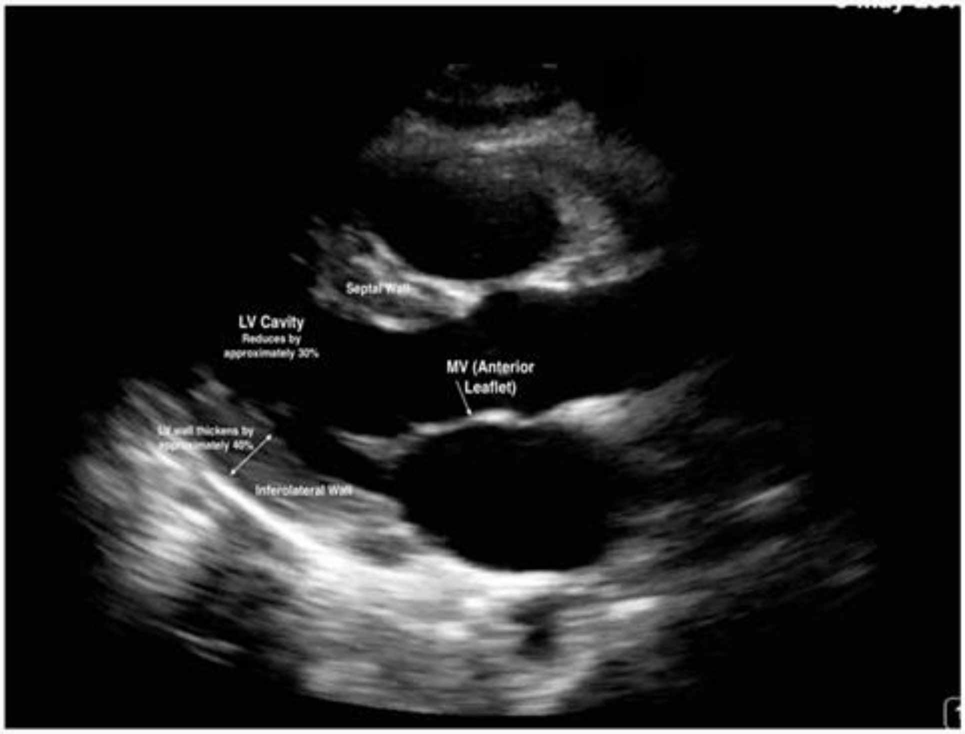

PSLA View - Normal Function

Normal example is shown. Note that the septal wall and inferolateral wall of the LV thicken by approximately 40% during systole. Note the LV cavity reduces in size by approximately 30% visually.

Note the anterior leaflet of the mitral valve excurses to within 1 cm of the ventricular septum.

In the setting of cardiogenic shock, however, note that the septal wall and inferolateral wall of the LV do not thicken by approximately 40% during systole. Note that the LV cavity does not reduce in size by approximately 30% visually. Finally, note that the anterior leaflet of the mitral valve does not excurse to within 1 cm of the ventricular septum (see Critical Care Echo chapter for further discussion of mitral valve leaflet excursion).

Inadequate mitral leaflet excursion may best be depicted by way of E-Point Septal Separation (EPSS), which is visualized in the parasternal long (PSLA) or apical four chamber (A4C) views. EPSS is measured in M-Mode as the distance between the anterior mitral valve leaflet and the ventricular septum in early diastole (E). MV excursion will be decreased in those with reduced ejection fraction and cardiogenic shock due to a blunted gradient between the left atrium and left ventricle during diastole. Consequently, EPSS is decreased.

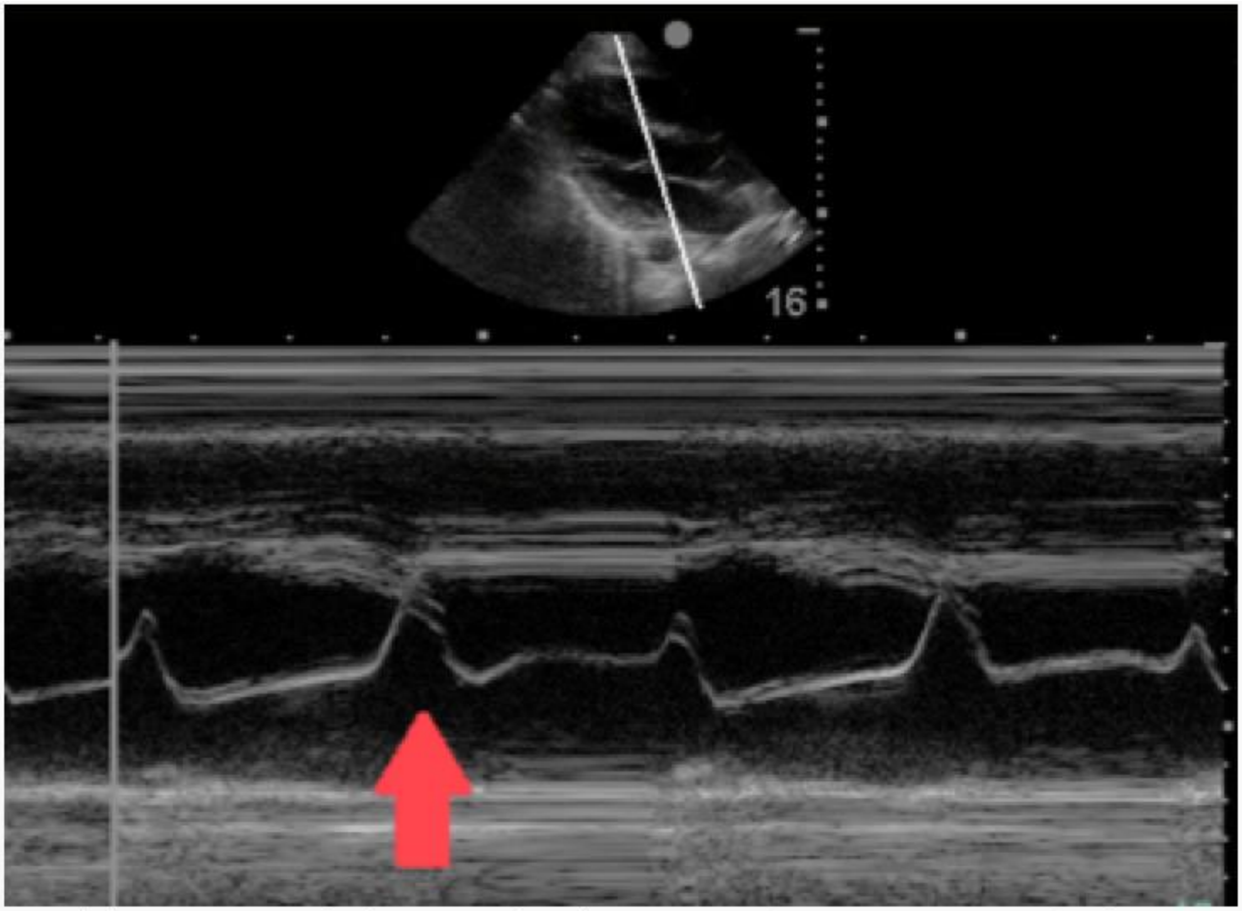

M-Mode demonstrating mitral valve opening. The red arrow points to the E-wave. EPSS is normal in this image.

Due to increased back-pressure, a distended inferior vena cava (IVC) with minimal respiratory variability, as well as distended internal jugular (IJ) veins, are findings consistent, but not limited, cardiogenic shock. Similarly, the presence of pulmonary edema is manifested as a B-line predominant pattern on lung US.

An A-line predominant pattern, on the other hand, should prompt an alternative diagnosis to pulmonary edema, and similarly would be unusual to see in the setting of cardiogenic shock.

Ultrasound in Obstructive Shock

Obstructive shock, like cardiogenic, is characterized by a distended IVC with minimal respiratory variability. The three most common types of obstructive shock encountered in

medical clinical practice are cardiac tamponade, tension pneumothorax (PTX), and massive pulmonary embolism. Each condition has distinct management strategies and therefore requires careful diagnostic decision making.

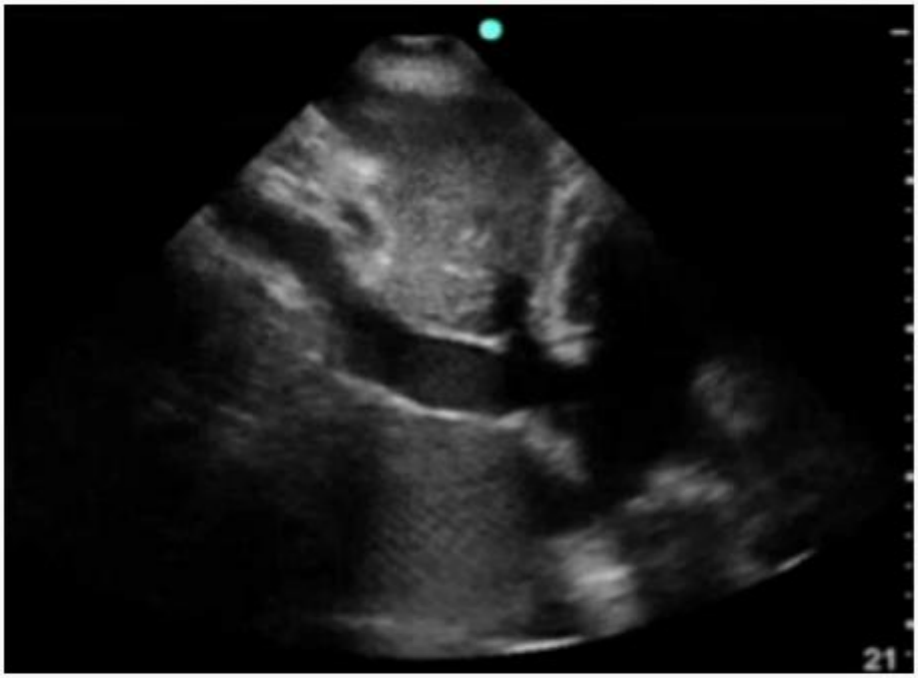

A distended IVC (as seen in cardiogenic and obstructive shock)

Cardiac Tamponade

A pericardial effusion can best be seen in the subcostal four chamber (SC4) or the PSLA view as an anechoic collection surrounding the free walls of the heart. While not all pericardial effusions are manifested by tamponade physiology, the presence of shock and hypotension in the setting of a pericardial effusion suggest tamponade, as tamponade is a clinical diagnosis. Bedside echo findings that are consistent with tamponade physiology include right atrial (RA) and right ventricular (RV) diastolic collapse, which may be observed with the S4C and PSLA views in either B-mode or M-mode.

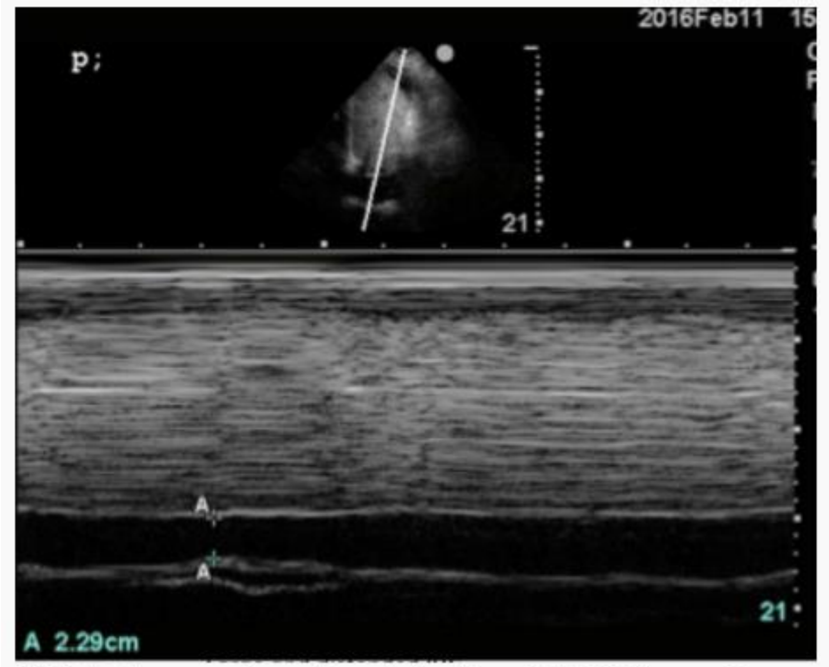

M-Mode demonstrating a distended and invariable IVC (as seen in

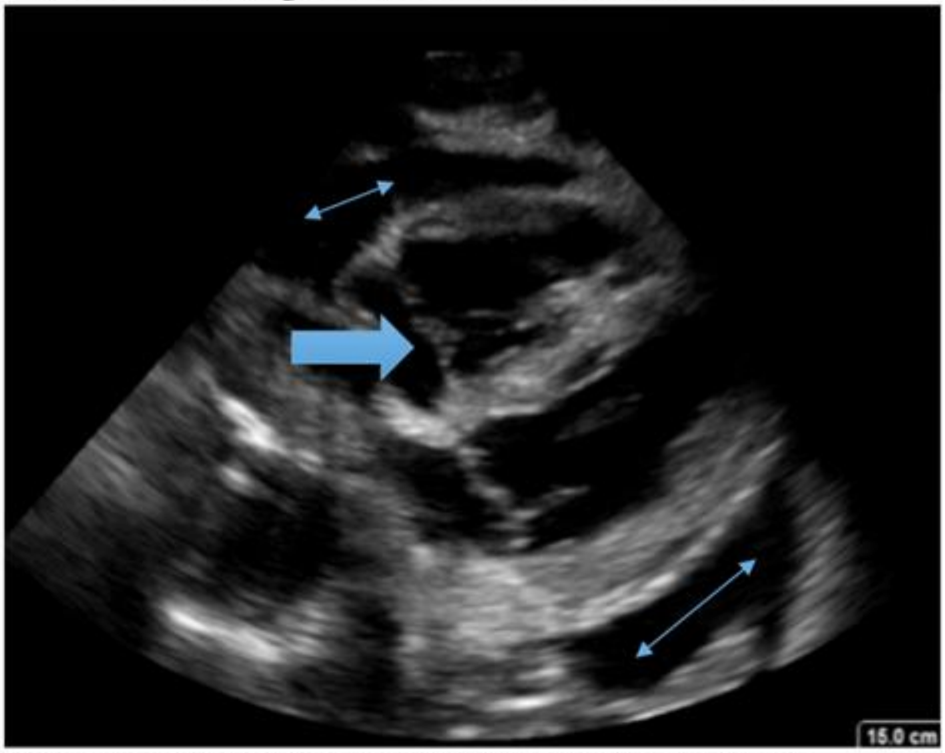

Right atrial collapse (thick blue arrow) with pericardial effusion (thin double arrows) in cardiac tamponade

Recording a clip on the machine (6 – 10 seconds) and then playing back at ½ or ¼ speed can help identify collapsing cavities. M-mode can be used to delineate collapse if it is not readily apparent. A simple use of M mode for this purpose is described here:

- Start with PSLA view
- Select M-mode and scroll to make appropriate selection
- Select M-mode again and freeze the screen to evaluate for collapse (figure shown)

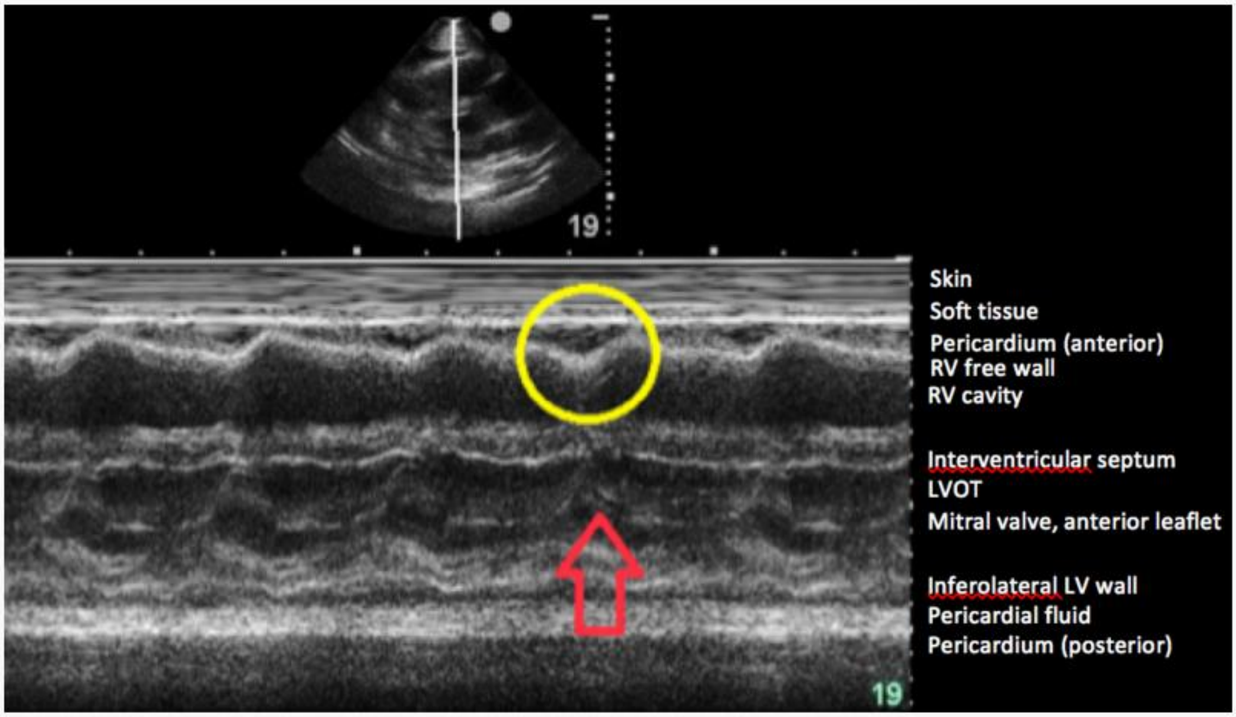

M-Mode of RV collapse during cardiac tamponade. Yellow circle indicates RV collapse while the red arrow points out the E-wave (mitral valve opening). Compare this image to the above demonstration of EPSS

Tension Pneumothorax

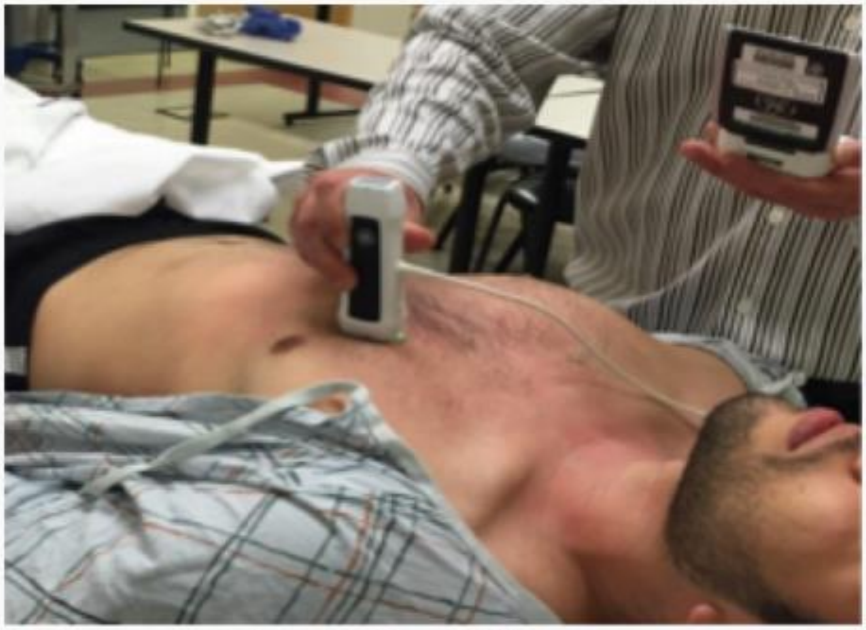

Probe placement in the evaluation of PTX

A PTX that is large enough to cause obstructive shock can be rapidly identified in an anti-gravity position at the anterior chest along the mid-clavicular line in a supine patient. If PTX is clinically suspected, check for lung sliding bilaterally in the second and third intercostal space with a high frequency linear probe. Lung Sliding is the ultrasound finding which is generated when the two layers of pleural are juxtaposed and gliding against each other. This finding is abolished in the presence of a pneumothorax. Similarly, because B- lines are a visceral pleural artifact, they cannot be visualized when a pocket of air (ie PTX) intervenes between the two layers of pleura. In summary, the presence of lung sliding or the presence of B-lines definitively rules out PTX at those specific sites.

Absence of lung sliding associated with an A-line pattern does not confirm a diagnosis of PTX; however, these findings in the appropriate clinical context (e.g., post-central venous catheterization; post-pacemaker implantation; following multiple rounds of chest compressions) are highly specific for PTX. It is also of benefit to confirm the presence of lung sliding prior to such procedures because if lung sliding is lost post-procedure, then the clinical suspicion that absent lung sliding (with A-line type pattern) is due to PTX is essentially 100% in clinical context of respiratory compromise and shock.

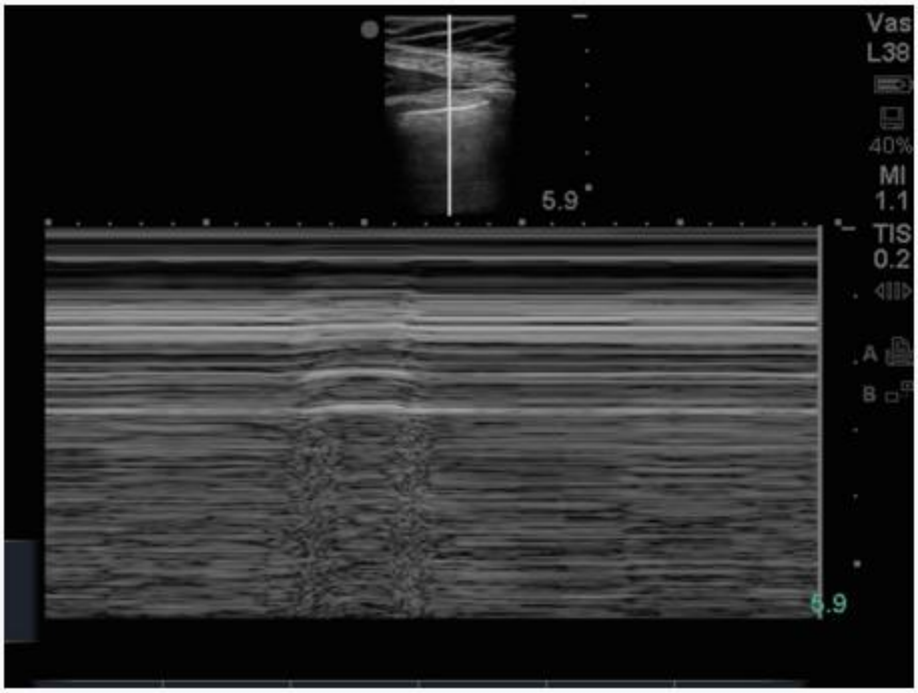

M-Mode demonstrating a “barcode” or “stratosphere sign” which is consistent with PTX

An absence of lung sliding produces “stratosphere sign,” also known as ”barcode sign,” with the M-Mode function. If pneumothorax is suspected, one may attempt to find the “lung point” using B-mode, which represents the interface of inflated lung and the area of pneumothorax; this can confirm the diagnosis. Lung point can be located by finding the section of lung where lung sliding and an absence of lung sliding are both observed in the same field of view.

Acute CorPulmonale

A normal RV cavity diameter is usually half that of the LV or smaller. In clinical practice we accept up to 60 – 75% of LV size. An RV that is near equal to or even larger that the LV is of serious concern during shock state, especially if this finding is thought to be new onset or secondary to PE.

Visual estimation of the RV in an A4C or a SC4 view is performed using a phased array low frequency transducer probe. Visual estimation of RV size usually suffices without any formal measurement. During acquisition of an A4C view, be careful not to (1) foreshorten or (2) inappropriately rotate the image when assessing the right ventricle as this may lead you to over- or underestimate true RV size. This can be avoided by (1) achieving the view from as lateral and inferior and possible and (2) rotating the probe until a view which contains all four chambers and both valves are visible. In particular, the two valves must be symmetrically viewed – meaning leaflets of both valves are visible. Avoid views which display the annulus of one valve while the leaflets of the other.

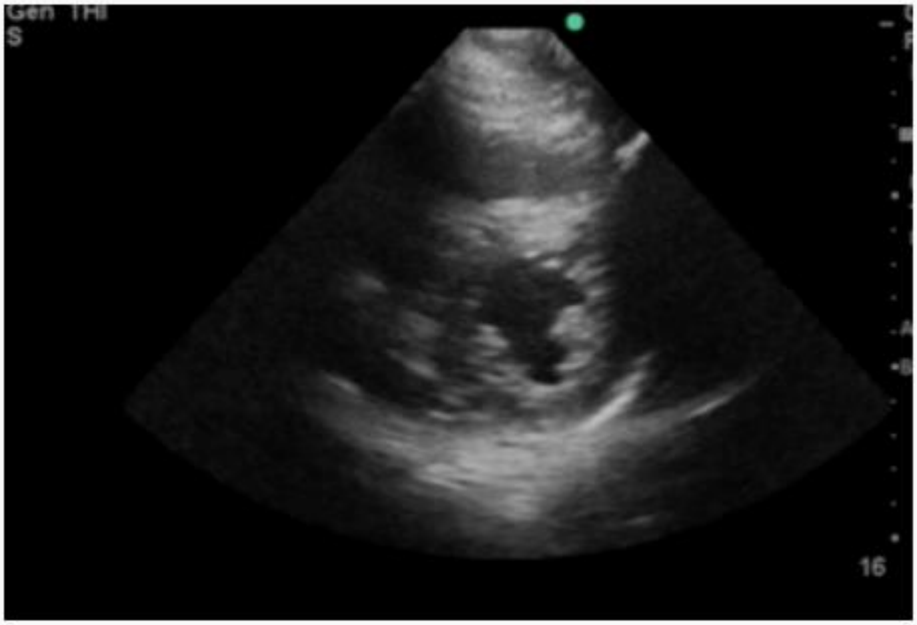

Normal PSSA View - Note the circular LV

RV pressure overload can be seen as interventricular septal bowing, which appears as “D sign” on a PSSA view. Obtain a parasternal short axis (PSSA) view at the level of the papillary muscles to detect bowing of the interventricular septum into the LV, this causes the normally circular LV to take on the shape of the letter D (known as D-sign), a sign of RV strain.

McConnell's sign is an US finding nonspecific to acute PE, but seen in acute corpulmonale and RV failure, which demonstrates relative RV apex hypercontractility and RV free wall hypokinesia; it can also be viewed in the A4C view.

If PE is suspected, one may also perform a lower extremity venous US examination to identify DVT, as over 90% of PE originate from DVT When identifying a new DVT, in the appropriate clinical setting, once can begin anticoagulation and bring clinical suspicion for PE to the very top of the differential (see DVT chapter).

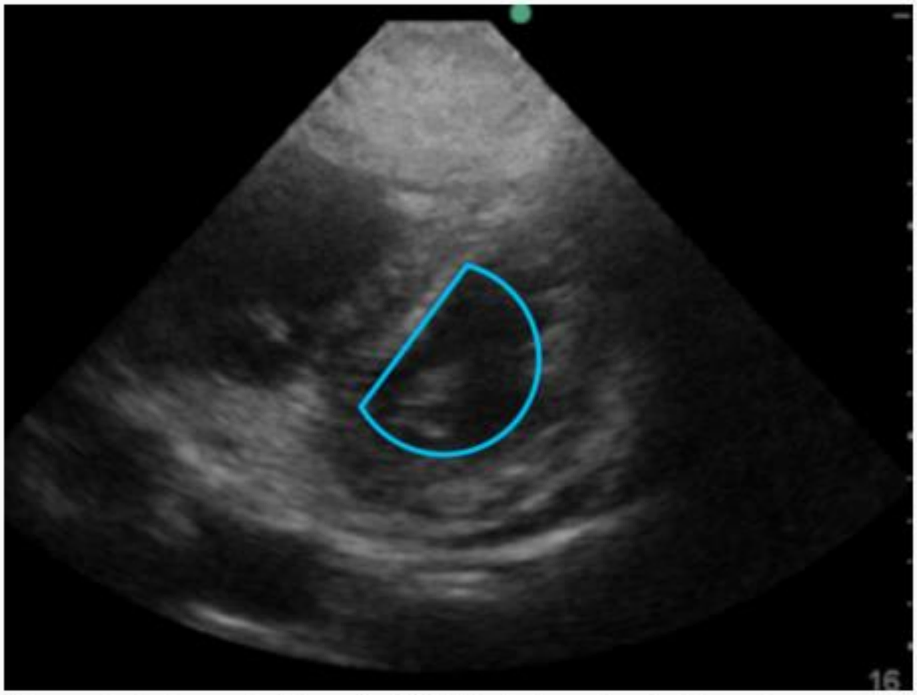

Interventricular septal bowing (D Sign) after a large PE and associated cor pulmonale. The LV cavity is outlined to illustrate the D shape of the LV in short axis during systole. Compare to normal (above figure).

Ultrasound in Hypovolemic Shock

One should suspect hypovolemic shock in hypotensive patients who are bleeding, have had recent surgeries or catheterizations, and all other patients prone to intravascular volume depletion. The IVC should be visualized using standard technique (see IVC in Shock chapter). A small IVC with a high respiratory variability suggests that fluid administration to increase preload would be beneficial.

Bedside echo may reveal hyperdynamic ventricular function. This may be seen in most views. One may observe "kissing ventricles" in the PSSA view, in which the ventricular walls nearly touch during peak systole. Lung US findings typically reveal an A-line predominant pattern.

Ultrasound in Septic Shock

Septic shock is the most common form of distributive shock. Due to systemic peripheral vasodilation, central venous pressure is reduced. A collapsed or small IVC with a high degree of respiratory variability suggests central venous intravascular depletion, a finding consistent with both hypovolemic (due to true loss of preload) and septic (due to loss of SVR) shock. Differentiating these two types of shock is difficult. Bedside echo, however, may aid in making the distinction. While hyperdynamic characteristics suggest hypovolemia or early sepsis, one may see hypodynamic behavior in late sepsis. Lung US may initially reveal an A-line pattern but

as sepsis progresses and vascular permeability worsens, an A-line predominant pattern may give way to B-lines.

US guides management in septic shock by: (1) guiding fluid administration and predicting a need for vasopressor therapy (IVC) and (2) aiding in identification of an infected source. Many sources of infection can be rapidly identified using bedside US, including pneumonia, empyema, abscess, nephrolithiasis, and cholecystitis.

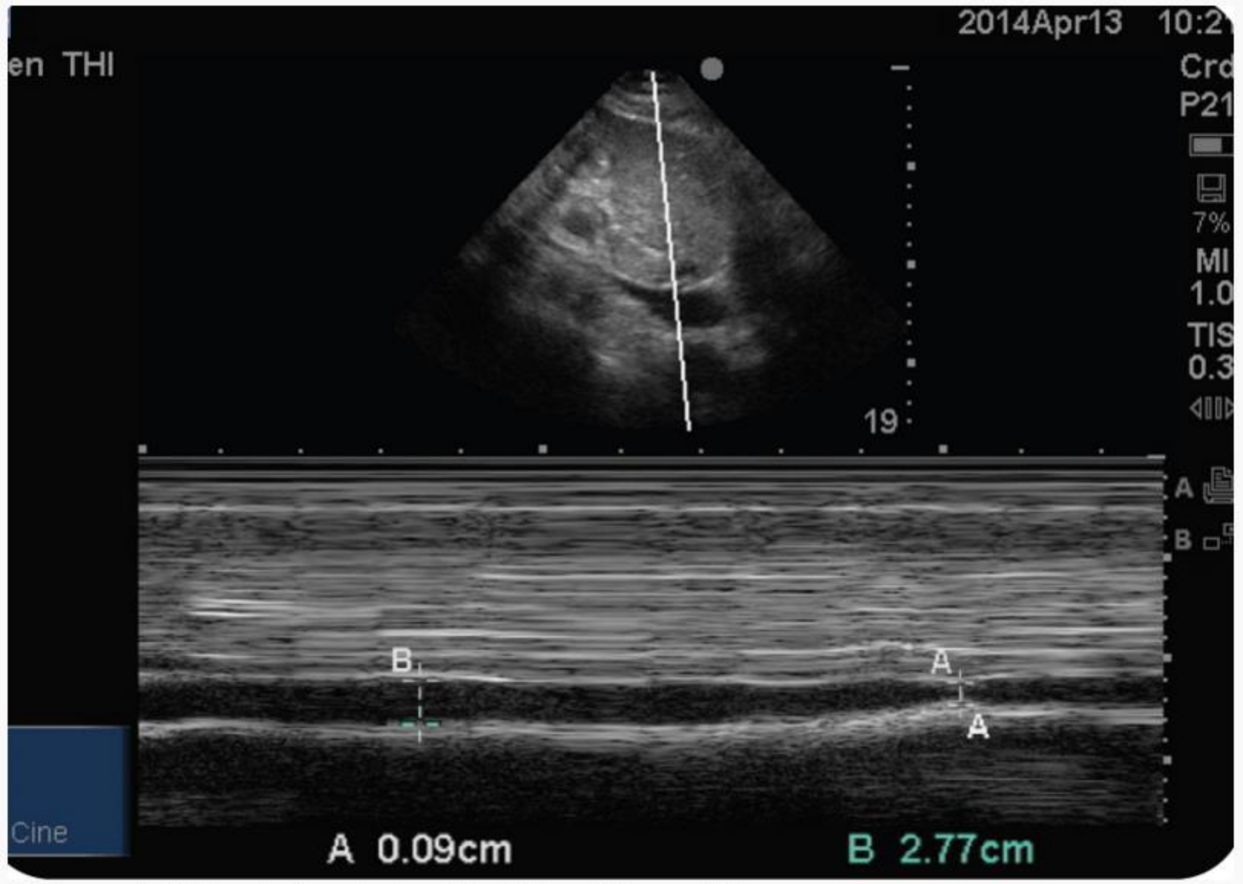

In this scenario this hypotensive patient could benefit from fluid resuscitation

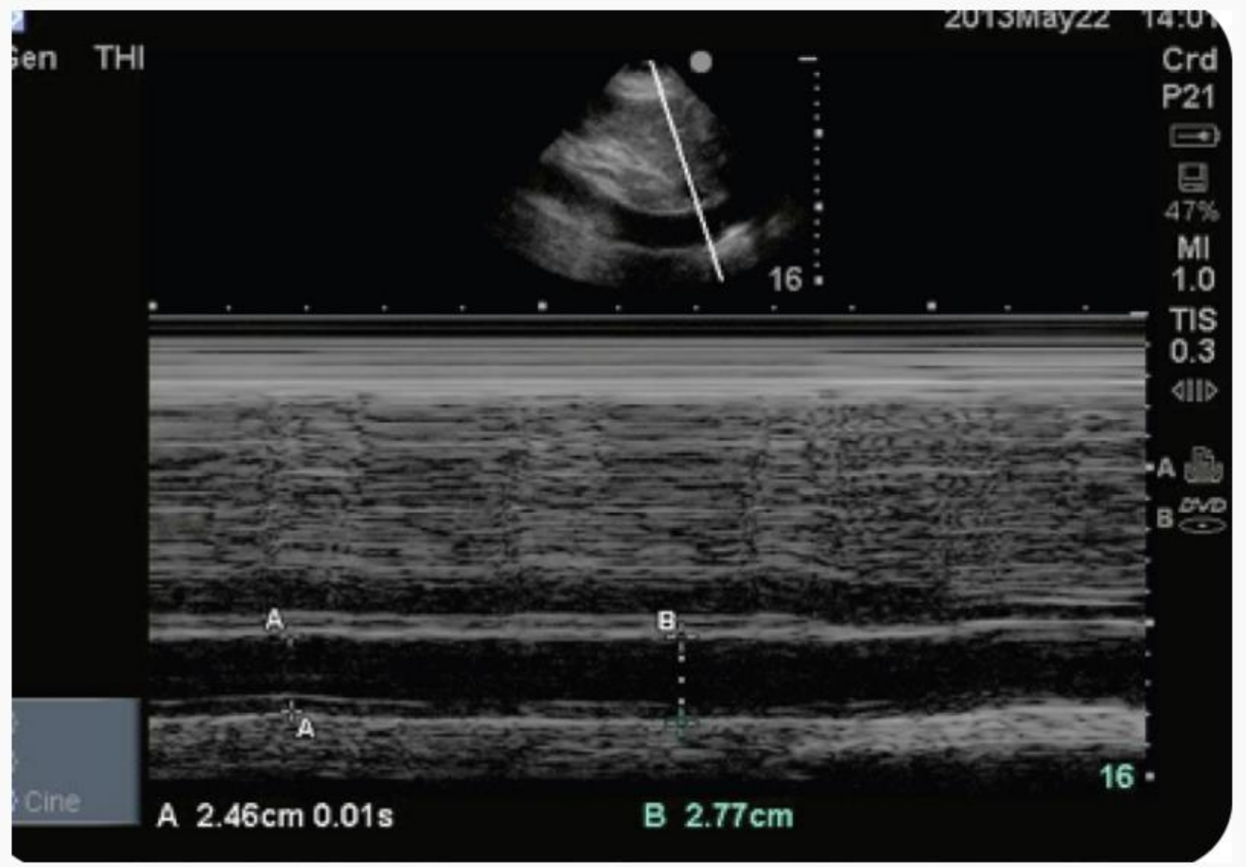

In this hypotensive patient, fluid resuscitation may not be beneficial, starting pressers given the large IVC state is indicated

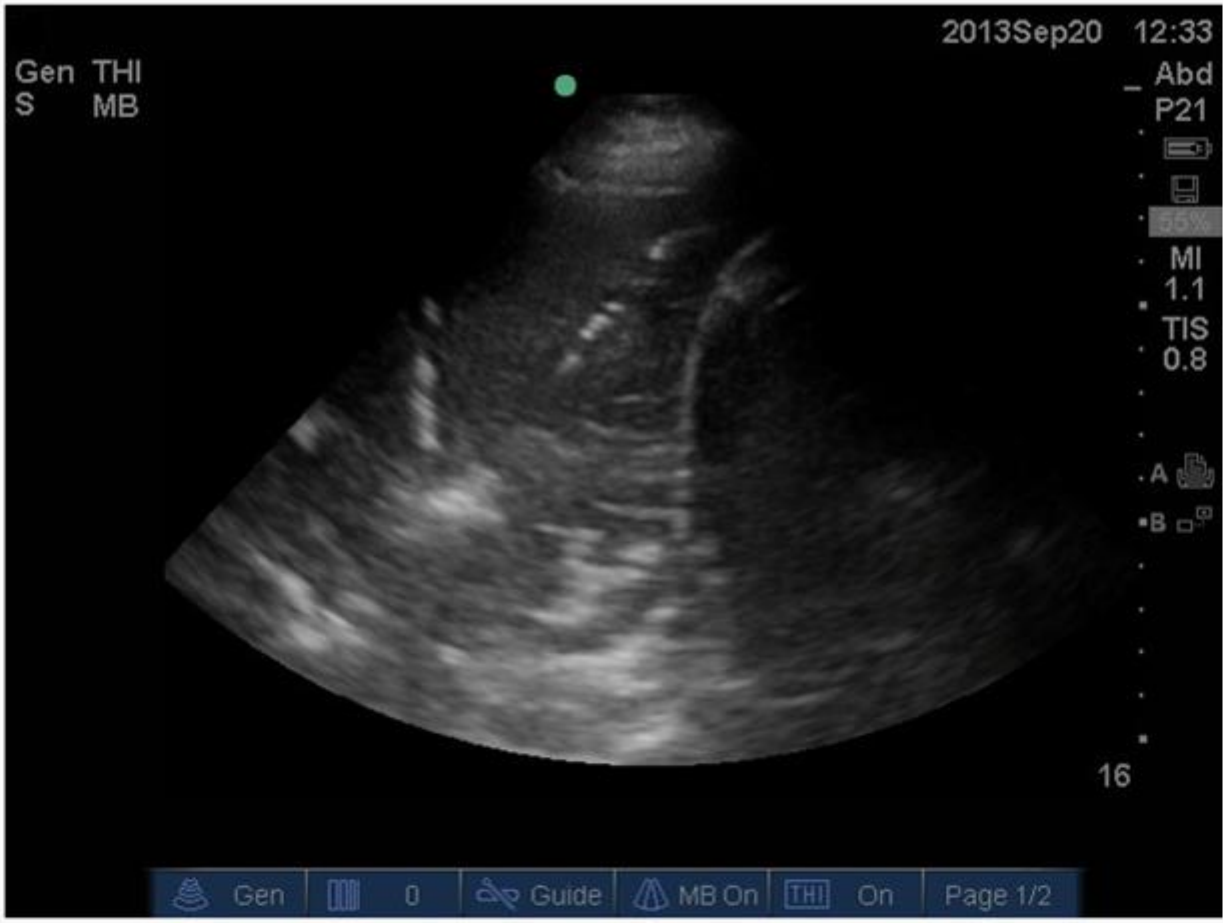

Air bronchograms and parenchymal changes consistent with pneumonia

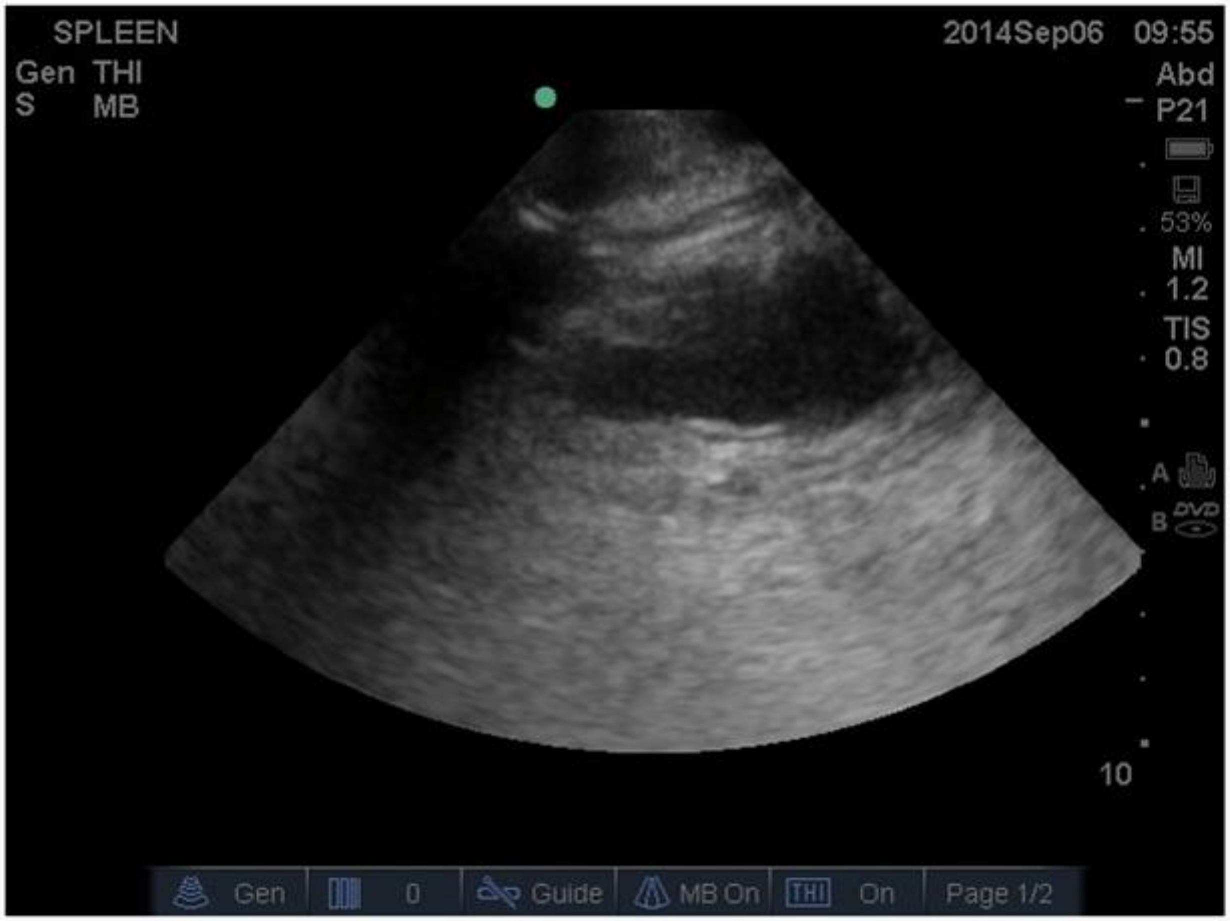

Walled off space consistent with abscess formation.

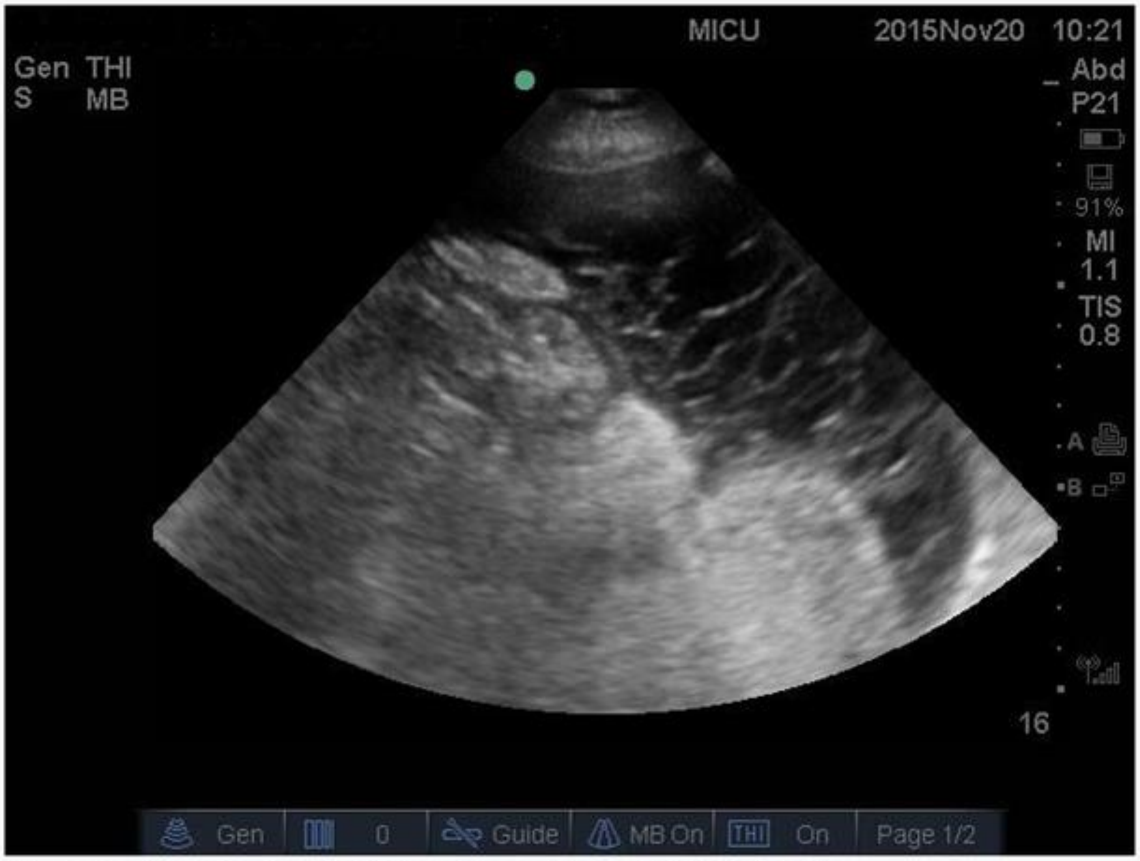

Fibrinous organization in abdominal cavity consistent with infected ascites

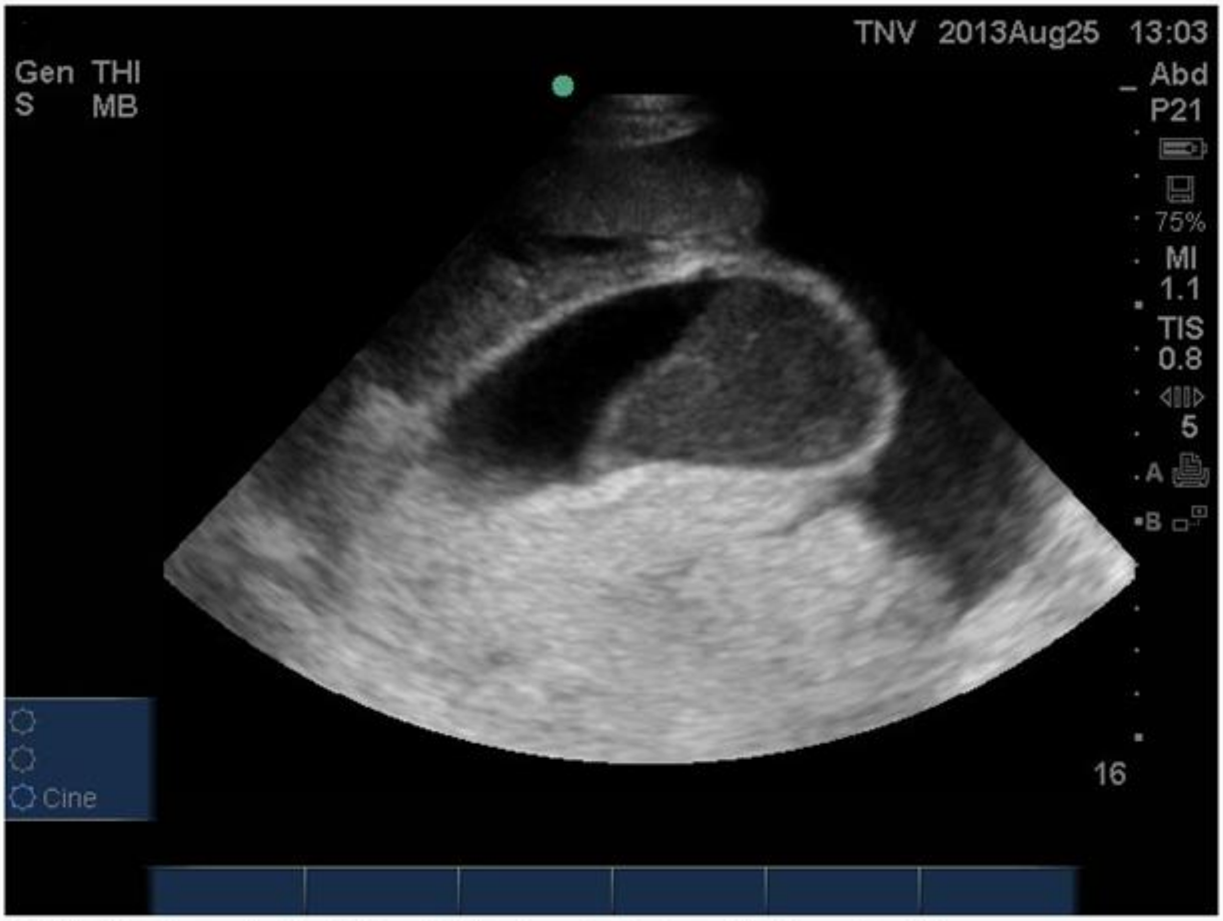

Gallbladder demonstrating biliary sludge, in acute cholecystitis there should be a corresponding increase in wall thickening and edema.

Ultrasound Approach To Undifferentiated Shock

We suggest the following protocol to aid in clinical judgment for the diagnosis and management of shock state

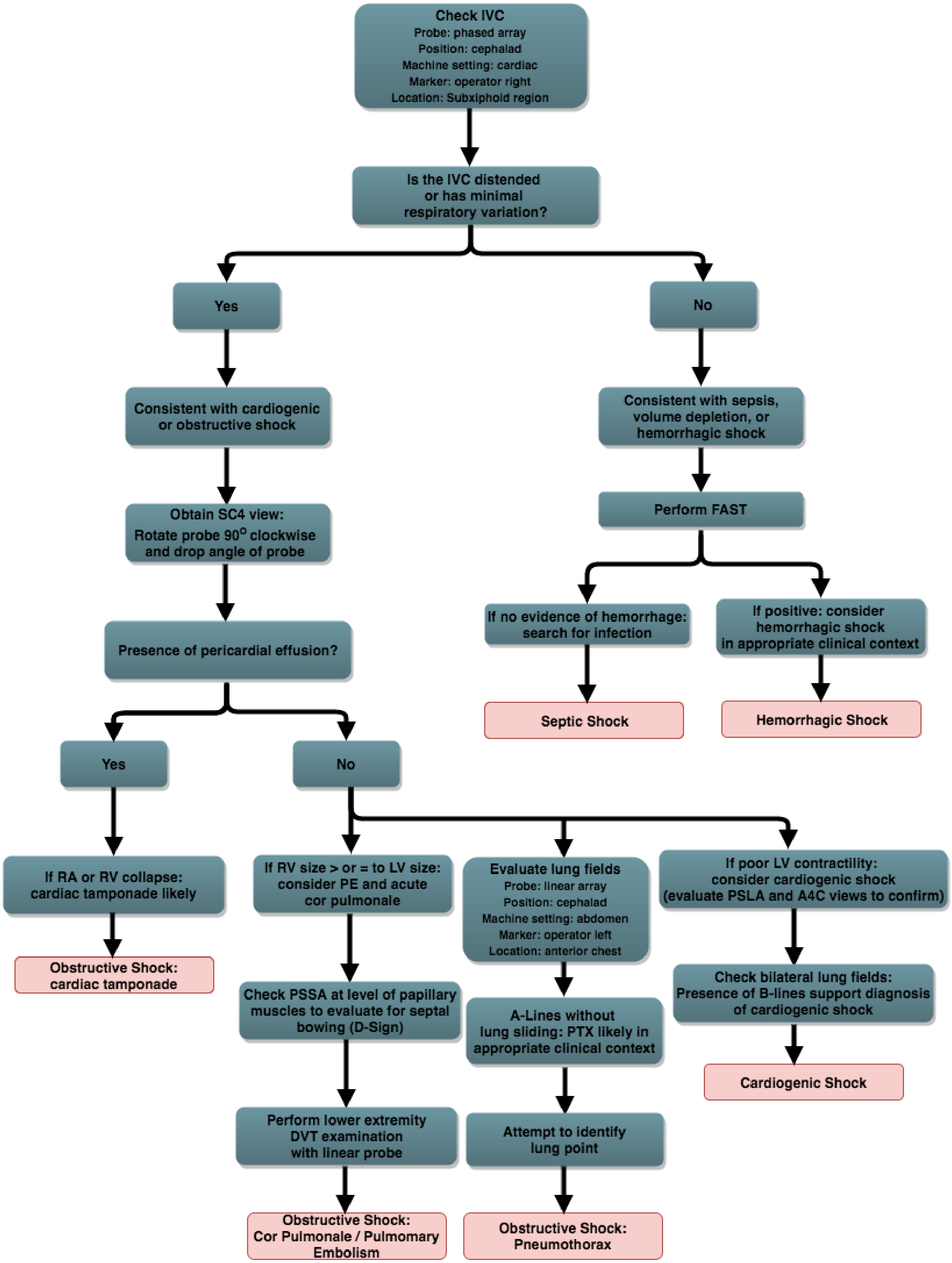
Check IVC
Probe: phased array
Position: cephalad
Machine setting: cardiac
Marker: operator right
Location: Subxiphoid region
Is the IVC distended or has minimal respiratory variation?
Yes
No
Consistent with cardiogenic or obstructive shock
Consistent with sepsis, volume depletion, or hemorrhagic shock
Obtain SC4 view: Rotate probe 90° clockwise and drop angle of probe
Perform FAST
If no evidence of hemorrhage: search for infection
If positive: consider hemorrhagic shock in appropriate clinical context
Septic Shock
Hemorrhagic Shock
Presence of pericardial effusion?
Yes
No
If RA or RV collapse: cardiac tamponade likely
Obstructive Shock: cardiac tamponade
If RV size > or = to LV size: consider PE and acute cor pulmonale
Check PSSA at level of papillary muscles to evaluate for septal bowing (D-Sign)
Perform lower extremity DVT examination with linear probe
Obstructive Shock: Cor Pulmonale / Pulmonary Embolism
Evaluate lung fields
Probe: linear array
Position: cephalad
Machine setting: abdomen
Marker: operator left
Location: anterior chest
A-Lines without lung sliding: PTX likely in appropriate clinical context
Attempt to identify lung point
Obstructive Shock: Pneumothorax
If poor LV contractility: consider cardiogenic shock (evaluate PSLA and A4C views to confirm)
Check bilateral lung fields: Presence of B-lines support diagnosis of cardiogenic shock
Cardiogenic Shock

Pearls and Pitfalls

- Clinical signs and symptoms as well as the scenario on presentation will be most helpful in differentiating septic from hypovolemic as US findings may be equivocal.
- Assessment of IVC is the best initial view to obtain as it can quickly differentiate major types of shock and guide management (e.g., choosing fluids versus vasopressors).
- When an IVC is difficult to image the internal jugular veins can act as a surrogate for determining central venous pressure.
- Be careful not to foreshorten your images when assessing the size of the right ventricle as this may give you a false impression of the ventricle's size.

<u>References</u>

1. Lichtenstein DA. Lung ultrasound in the critically ill. Ann Intensive Care. 2014 Jan 9; 4(1):1
2. Perera P, Mailhot T, Mailhot T, Mandavia D. The RUSH exam: Rapid Ultrasound in SHock in the evaluation of the critically ill. Emerg Med Clin North Am. 2010 Feb; 28(1):29-56.
3. Scalea TM, Rodriguez A, Chiu WC, et al. Focused Assessment with Sonography for Trauma (FAST): Results from an international consensus conference. J Trauma. 1999; 46:466–72.
4. Lichtenstein DA. How can the use of lung ultrasound in cardiac arrest make ultrasound a holistic discipline? The example of the SESAME-protocol. Medical Ultrasonography. 2014 Sep; 16 (3): 252-5.
5. Nagdev A, StoneMB. Point-of-care ultrasound evaluation of pericardial effusions: Does this patient have cardiac tamponade? Resuscitation. 2011 Sun; 82 (6):671-73.

Respiratory Failure- Ultrasound Protocol- Based Approach to Respiratory Failure

Sahar Ahmad, MD

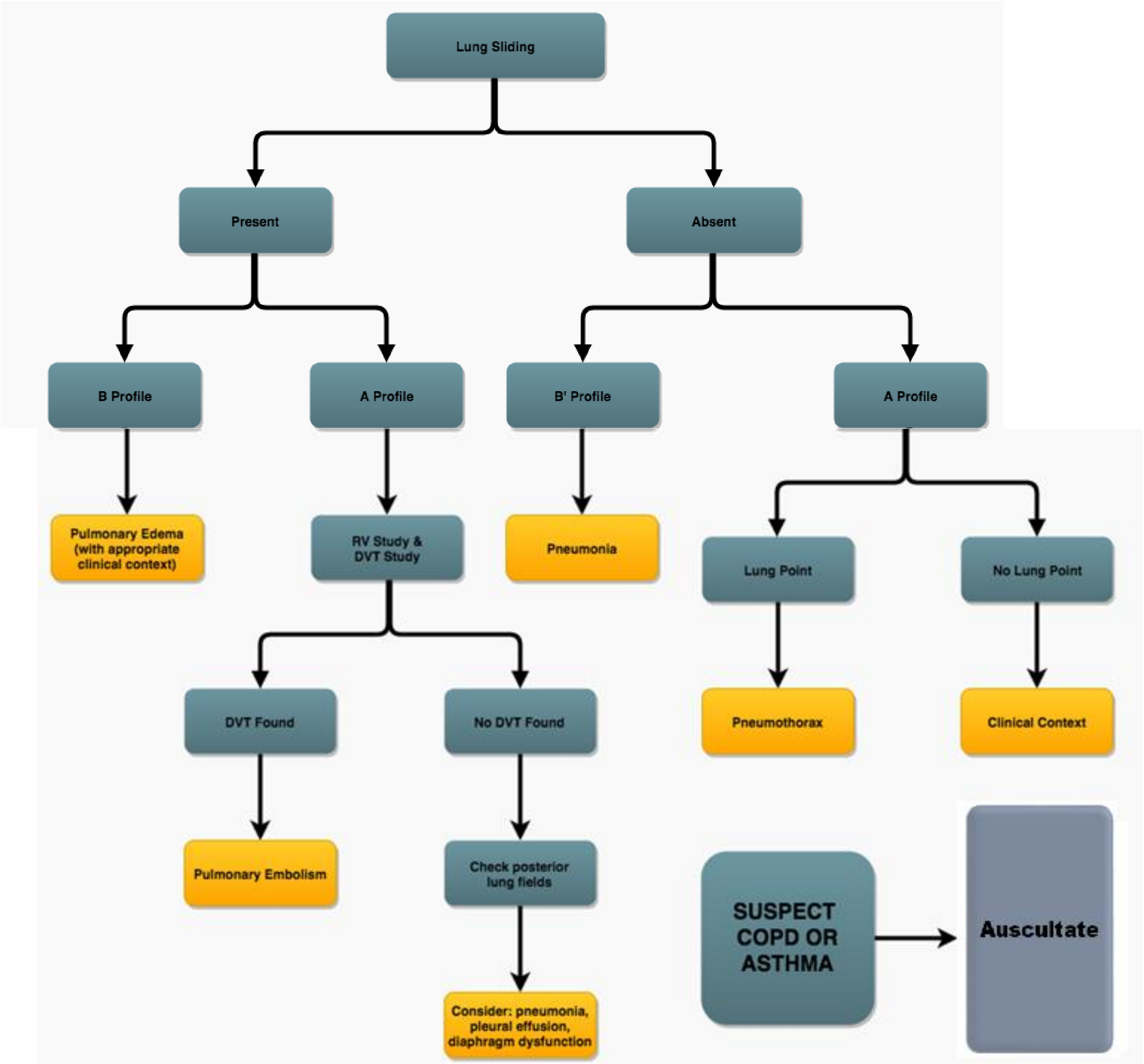

Supplement: Supplementary file 4 — Supplementary Material 4: Additional file 4 Ultrasound Manual [file 12909_2025_6802_MOESM4_ESM.pdf]
