# Supplementary material for: The design, performance and organizational impact of a point-of-care ultrasound (POCUS) elective for internal medicine residents
Source: BMC Med Educ. 2025 Feb 18;25:261. doi: 10.1186/s12909-025-06802-x (PMC11834687; doi:10.1186/s12909-025-06802-x)
Supplement: Supplementary file 5 — Supplementary Material 5: Additional file 5 SBUS Quick Guides [file 12909_2025_6802_MOESM5_ESM.pdf]

SBUS
Stony Brook Critical Care Ultrasound

Quick Guide to Vascular Access

All images are created by Dr. Sahar Ahmad

Technique:
- Transducer: linear array, high frequency; marker is operator left
- Machine setting: Vascular mode, screen marker: operator left

Find Optimal View

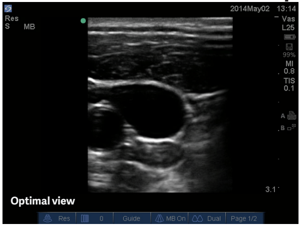

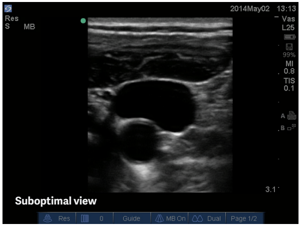

Use Tip Tracking

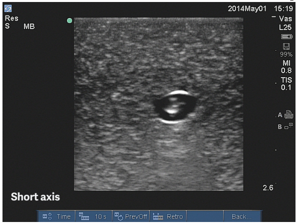

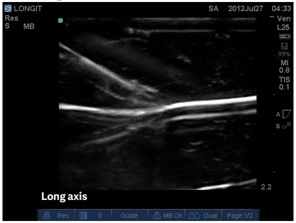

Confirm wire location

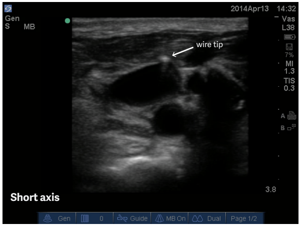

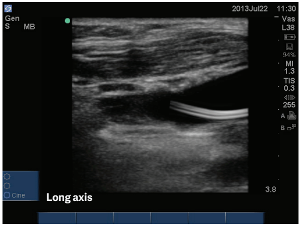

Review anatomy

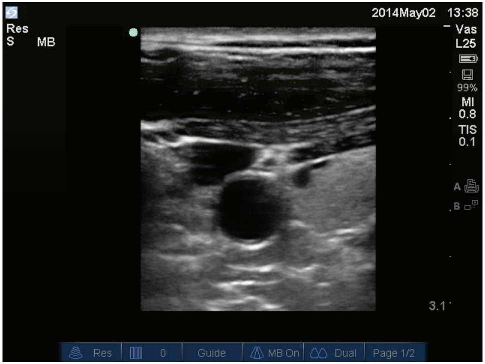

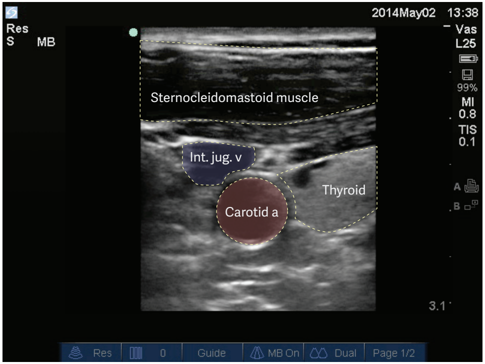

<Sahar.Ahmad@StonyBrookMedicine.edu>

SBUS
Stony Brook Critical Care Ultrasound

Quick Guide to Limited DVT Study

All images are created by Dr. Sahar Ahmad

Technique:
- Transducer: linear array, high frequency; marker is operator left
- Machine setting: Vascular mode, screen marker to operator left

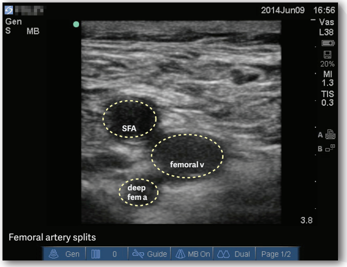

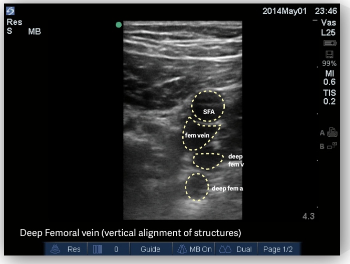

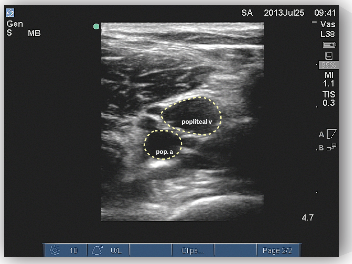

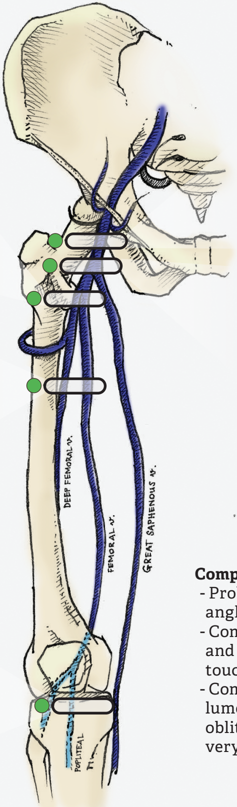

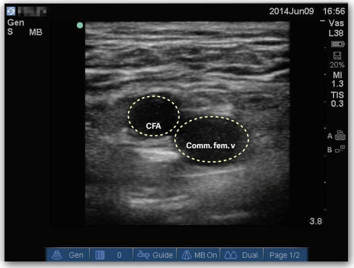

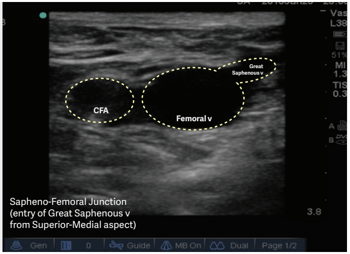

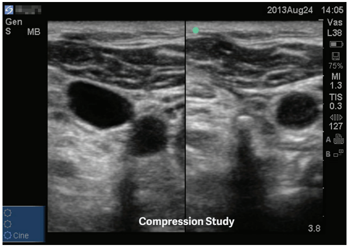

Compression Technique:
- Probe must be at 90˚ angle from vessel
- Compress until anterior and posterior edges touch.
- Compress until vein lumen is completely obliterated, or artery is very deformed.

Quick Guide to Chest Ultrasound

All images are created by Dr. Sahar Ahmad

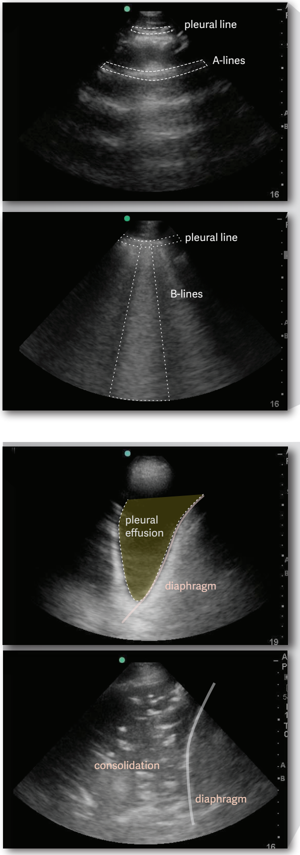

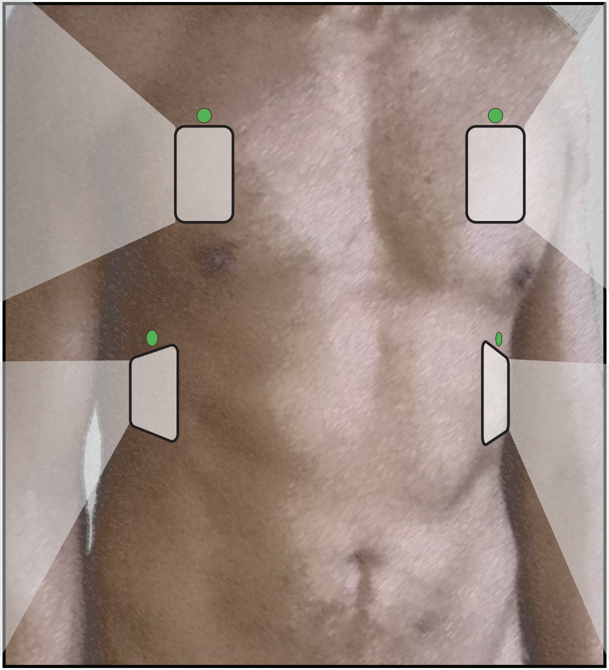

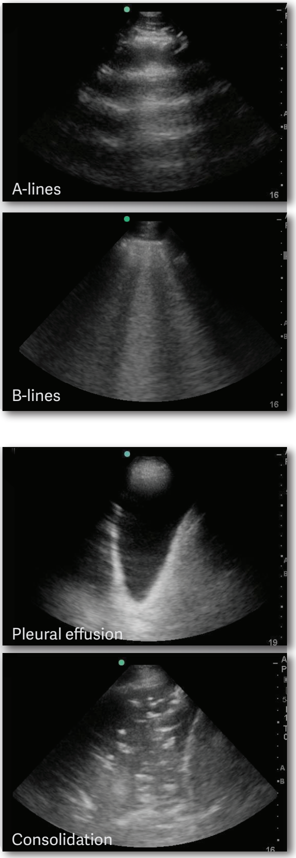

SBUS
Stony Brook Critical Care Ultrasound

Technique:
- Transducer: phased array, low frequency; marker is cephalad
- Machine setting: Abdominal mode, screen marker: operator left

<Sahar.Ahmad@StonyBrookMedicine.edu>

Quick Guide to Cardiac Ultrasound

Technique:
- Transducer: phased array, low frequency; marker is view-dependent
- Machine setting: cardiac, screen marker to operator right

All images created by Dr. Sahar Ahmad

Parasternal view: Long axis (PSLA)

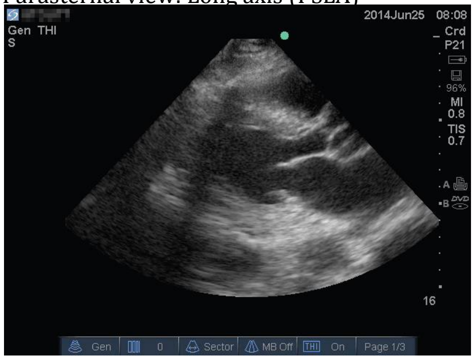

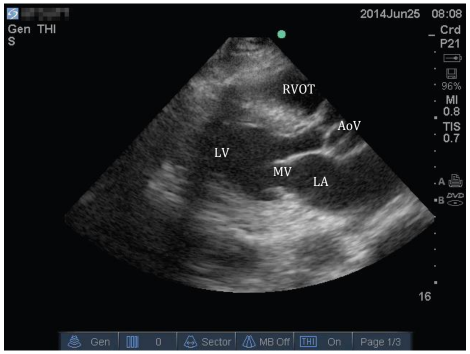

Parasternal view: Short axis: Level of Aortic Valve

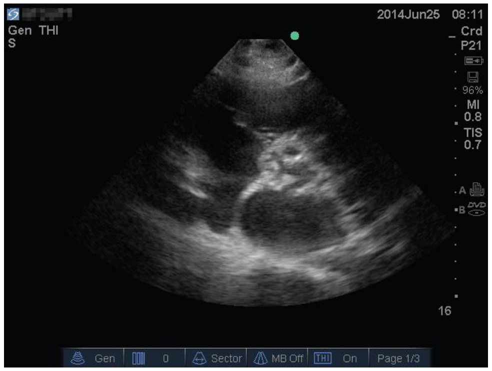

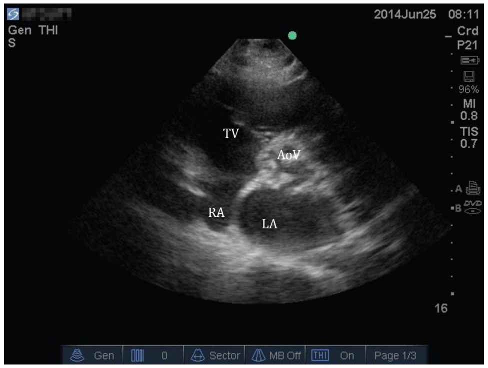

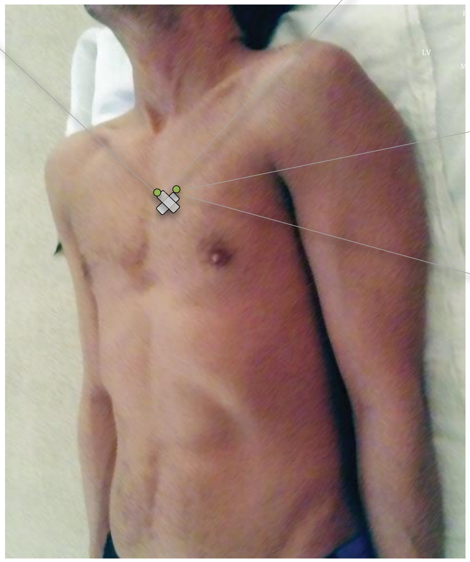

Parasternal view: Short axis: Level of Mitral Valve

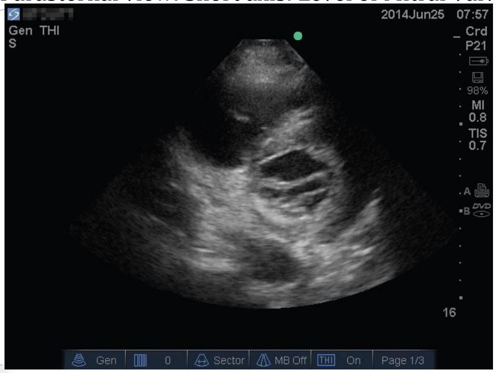

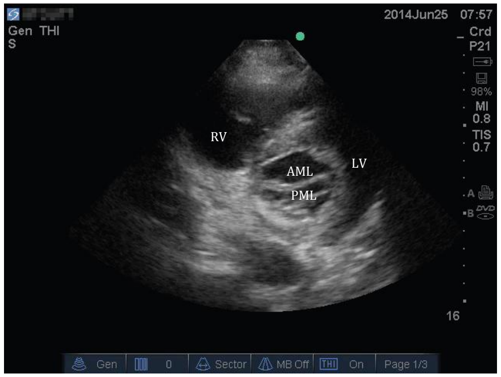

Parasternal view: Short axis: Level of Papillary Muscles

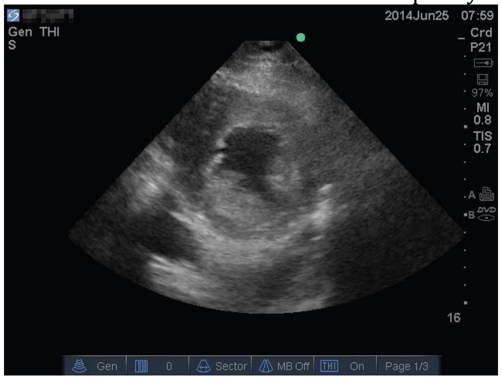

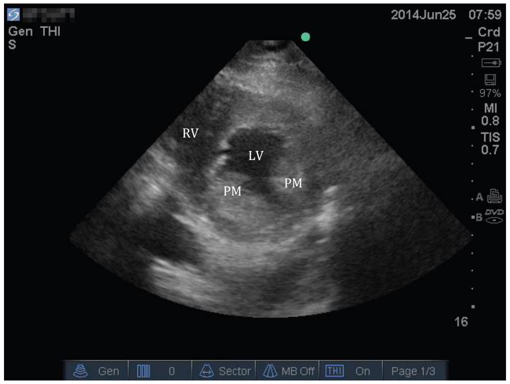

Abbreviations:

AoV: Aortic Valve
RVOT: Right Ventricular Outflow Tract
LV: Le_ Ventricle
RV: Right Ventricle
LA: Le_ Atrium
RA: Right Atrium
MV: Mitral Valve
AML: Anterior Mitral Leaflet
PML: Posterior Mitral

Quick Guide to IVC Ultrasound: Image Acquisition

All images created by Dr. Sahar Ahmad

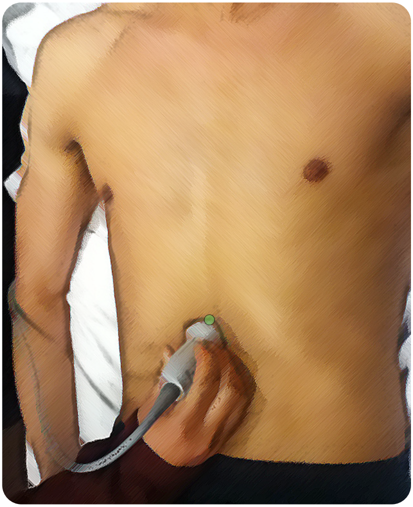

Machine setting: Echo (screen marker to operator right)

Transducer selection: Phased array, low-frequency transducer

Technique:

- probe marker cephalad
- place transducer at 90° angle to the patient at the sub-xiphoid region, slightly right-lateral, and slightly tilted cephalad
- visualize the cavo-atrial junction, diaphragm, liver, and IVC (in longitudinal plane)
- visualize the aorta prior to beginning measurements (slight angulation of the scan plane medially)

Measurements:

- set up M-Mode for anteroposterior diameter measurements of the IVC
- choose an M-Mode beam corresponding to a near-orthoganal section, 2–4cm distal to the cavo-atrial junction (just beyond visible hepatic vein)
- measurements should be taken using machine caliper, internal edge-to-internal edge

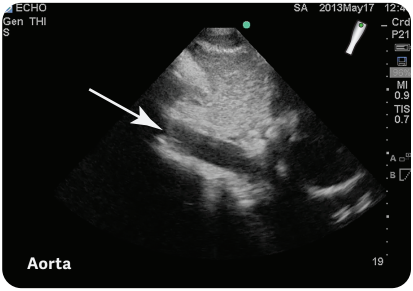

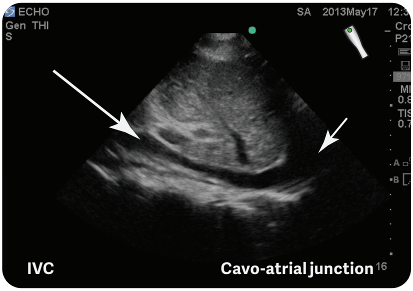

Dmax = Maximal IVC anteroposterior diameter

Dmin = Minimal IVC anteroposterior diameter

dIVC (Distensibility index of IVC) = (Dmax - Dmin) / Dmin, expressed as a percentage

cIVC (Collapsibility index of IVC) = (Dmax - Dmin) / Dmax, expressed as a percentage

Aorta:
- probe angled medially

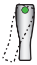

IVC:
- probe angled laterally

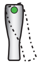

Quick Guide to IVC Ultrasound: **Image Interpretation**

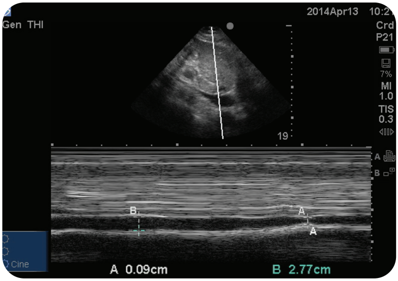

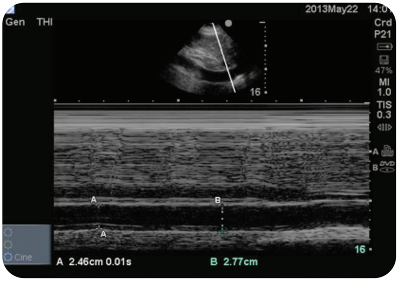

The Intubated Patient

Criteria: Vt >7cc/kg IBW, passive on vent, normal sinus rhythm (NSR)

Measurements: Dmax, Dmin

Calculation: dIVC

Interpretation: **dIVC >18%** across one respiratory cycle suggests that this patient's hypotension will be fluid responsive (90% Sn, 90% Sp).

Ref: Barbier, Intensive Care Med. 2004

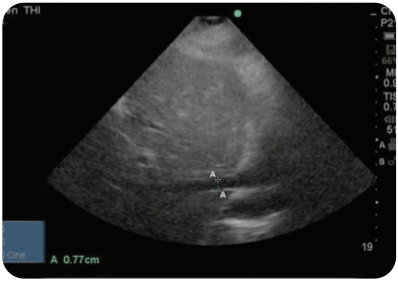

The Non-intubated Patient

Criteria: Calm tidal breathing in NSR

Measurements: Dmax (if using alternate method [cIVC], also measure Dmin)

Interpretation: **Dmax <1.0cm** suggests that hypotension will be fluid responsive.

Alt: cIVC >40% predicts CVP <7 (80% Sp), usually associated with fluid responsiveness; a low cIVC is poorly predictive.

Ref: Slama M, Amiens, France; Muller et. al. Critical Care 2012, 16:R188.

SBUS
Stony Brook Critical Care Ultrasound

<Sahar.Ahmad@StonyBrookMedicine.edu>

Quick Guide to Cardiac Ultrasound

Technique:
- Transducer: phased array, low frequency; marker is view-dependent
- Machine setting: cardiac, screen marker to operator right

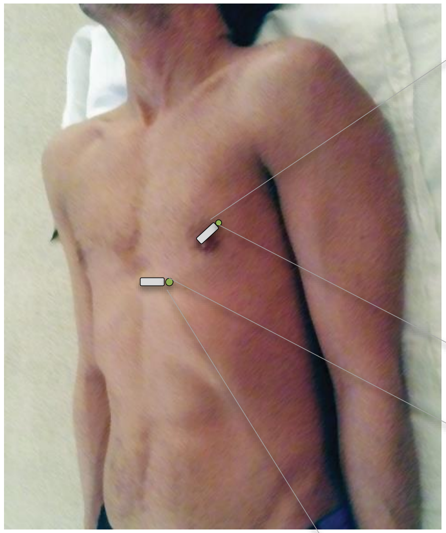

Apical view: Short axis: 4 Chamber

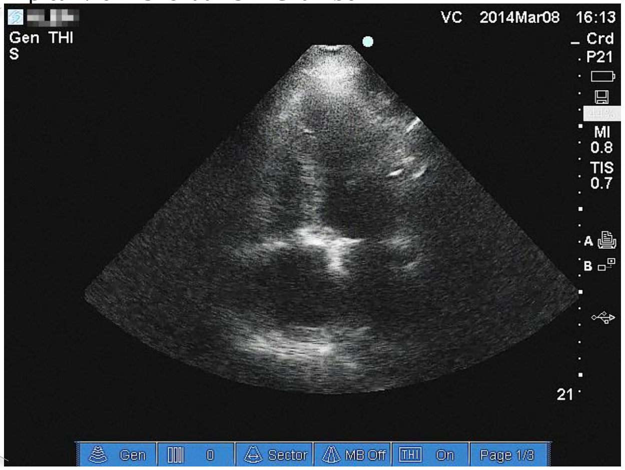

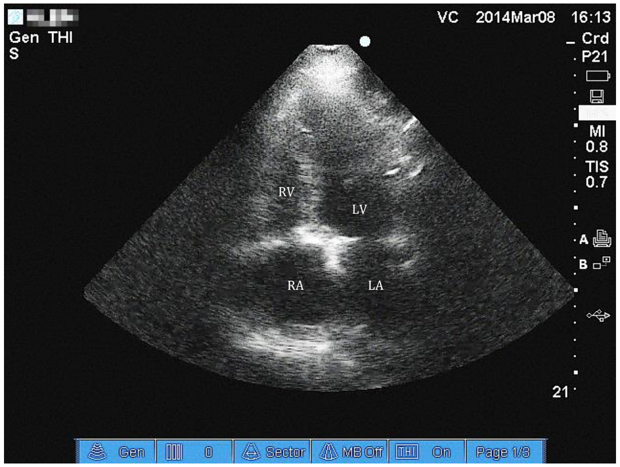

Subcostal view: 4 Chamber (SC4)

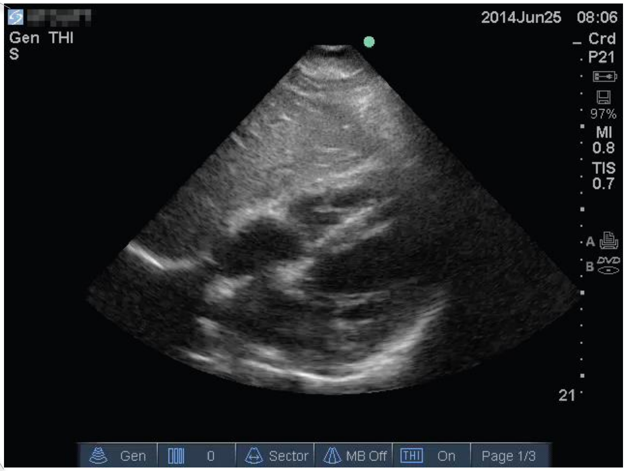

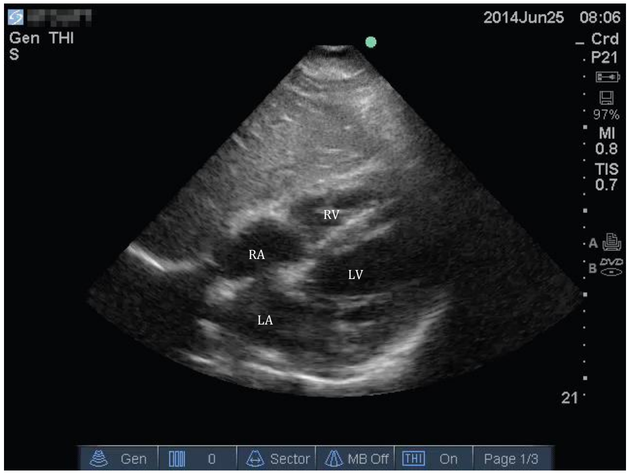

Abbreviations:

AoV: Aortic Valve
RVOT: Right Ventricular Outflow Tract
LV: Le_ Ventricle
RV: Right Ventricle
LA: Le_ Atrium
RA: Right Atrium
MV: Mitral Valve
AML: Anterior Mitral Leaflet
PML: Posterior Mitral Leaflet
PM: Papillary Muscles

Ultrasound-guided Assessment of Reversible Causes in Cardiac Arrest

All images created by Dr. Sahar Ahmad

Lung:
1. R/O PTX (absent lung sliding, or stratosphere sign)

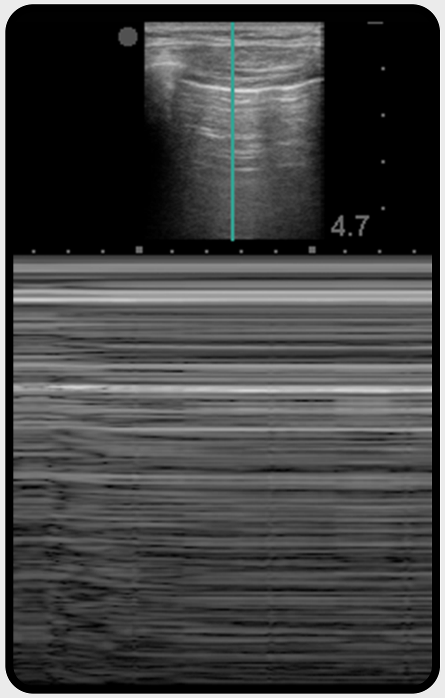

Abdomen:
1. R/O hemorrhage (collection at hepato-renal recess)
2. R/O hypovolemia (IVC <1cm)

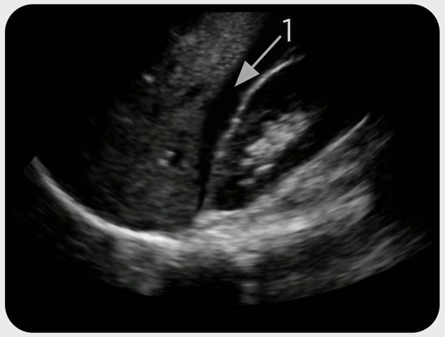

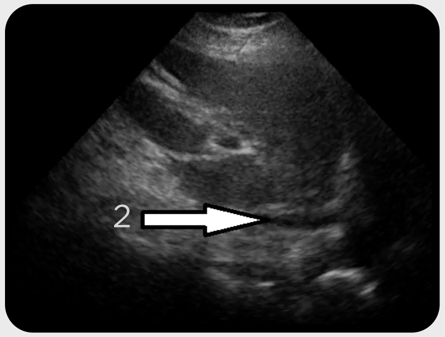

Cardiac (SC4)
1. R/O acute cor pulmonale (RV:LV >1)
2. R/O tamponade
3. Evaluate cardiac function (standstill, coarse/fine v-fib, LV dysfunction)

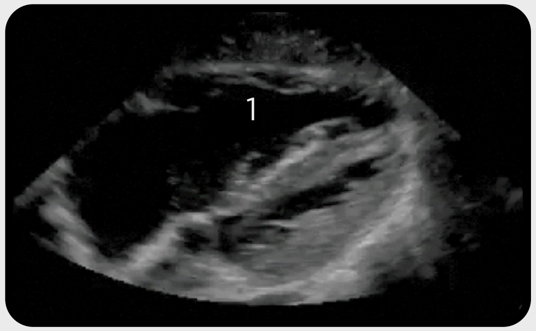

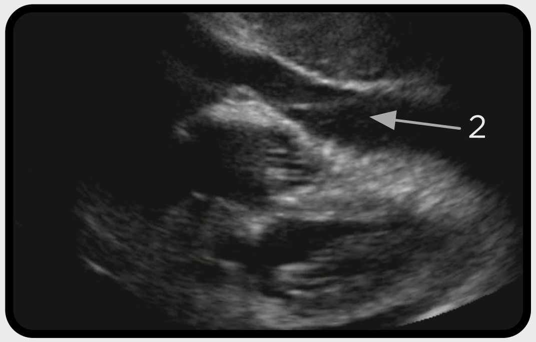

Post-intubation:
1. Verify ETT position (single air shadow at anterior neck)

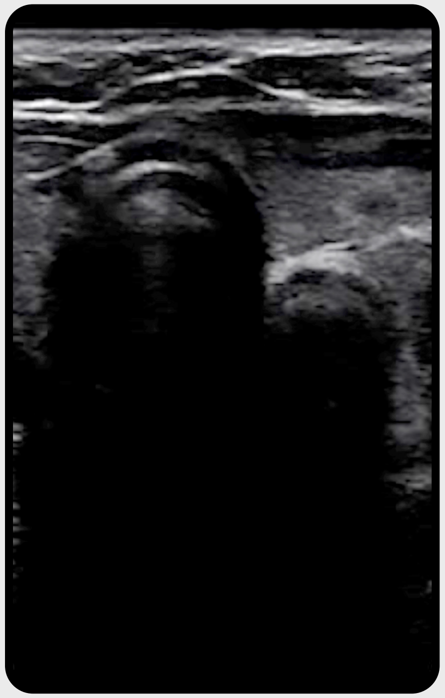

Post-ROSC Protocol

Cardiac views to assess overall post-ROSC function:
- PSLA
- PSSA
- A4C
- SC4

Lung views for respiratory failure protocol:
- bilateral anterior thorax to view pleura and parenchyma
- bilateral postero-lateral views

SBUS
Stony Brook Critical Care Ultrasound

<Sahar.Ahmad@StonyBrookMedicine.edu>
