# Supplementary material for: The design, performance and organizational impact of a point-of-care ultrasound (POCUS) elective for internal medicine residents
Source: BMC Med Educ. 2025 Feb 18;25:261. doi: 10.1186/s12909-025-06802-x (PMC11834687; doi:10.1186/s12909-025-06802-x)
Supplement: Supplementary file 6 — Supplementary Material 6: Additional file 6 Consult note [file 12909_2025_6802_MOESM6_ESM.pdf]

SBUS Ultrasound Elective: Consult Notes Required Image List

Submit minimum of one example of each of the following images:
Remember to optimize your image (depth and gain set to enhance and focus on the structure of interest).
Remember to name/describe your finding, state machine setting, state which transducer used, its position on the body.

Required (can be found in most/all patients)
1. Lung sliding in M-mode
2. A-lines
3. Hepato-renal recess (include visualization of kidney, liver, diaphragm).
4. Common femoral vein and femoral artery proximal to sapheno-femoral junction
5. Sapheno-femoral junction
6. Superficial femoral vein
7. Urinary bladder
8. Parasternal Long axis view of the heart
9. Parasternal short axis view, at the level of the papillary muscles (mid-ventricular view)
10. Subcostal 4 chamber view
11. IVC, longitudinal view, in M-mode
12. Aorta, longitudinal view, B-mode in still frame

As come across (can be found in many cases if look for it)
1. B-lines
2. Pleural effusion, from mid- axillary line
3. Bladder with Foley in place
4. Aorta in short axis, from anterior mid- abdomen
5. Ascites

Writing an EMR Ultrasound note:
Use Pulmonary Freetext note format

Chest Ultrasound note *(Also can title as applicable: Limited Abdomen Ultrasound; Limited Vascular ultrasound, etc.)*
Procedure:
Indication:
Operator: *(yourself)*
Supervisor:
Findings/Images: *(insert images here with brief labels)*
Interpretation: *(write out interpretation of the collection of images; if applicable include EBM citation.)*

Not Official Copy: Pulmonary/MICU Event
Date/Time of Service: 19 May 2015 21:01
Result Status: Final
Result Title: Chest Ultrasound Note
Performed By: AHMAD , SAHAR on 19 May 2015 21:02
Verified By: AHMAD , SAHAR on 19 May 2015 21:02
Encounter info: [redacted], Stony Brook University Hospital, IP Medically Justified, 05/16/2015 -

* Final Report *

Chest Ultrasound Note

Patient: [redacted] **MRN:**[redacted] **FIN:** [redacted]
Age: **22 years** Sex: **Male** DOB: [redacted]
Associated Diagnoses: **None**
Author: **AHMAD , SAHAR**

..
Chest Ultrasound note

Procedure: B scan ultrasound chest (left anterior, left posterior)
Indication: pleural effusion
Operator: S. Ahmad MD
Supervisor: n/a
Findings/Images:

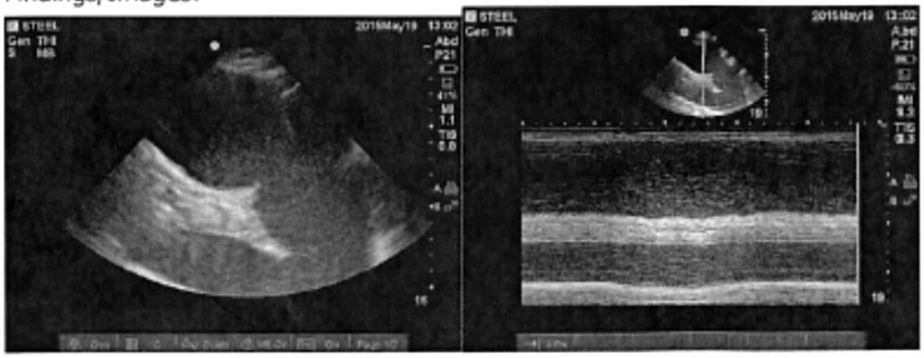

Above left: pleural effuson large surrounding atelectatic lung. Notable is the grainy appearance of the gravity dependent region of the fluid.
Above right: M- mode over atelectatic lung. Absent sinusoid sign.

Interpretation:
1) Large exudative effusion is definitely diagnosed
2) Absent sinusoid sign in my experience predicts a poorly expansile lung. of note other features of this lung not shown here suggest at least partial expasnion if fluid is removed.
