# Supplementary material for: The design, performance and organizational impact of a point-of-care ultrasound (POCUS) elective for internal medicine residents
Source: BMC Med Educ. 2025 Feb 18;25:261. doi: 10.1186/s12909-025-06802-x (PMC11834687; doi:10.1186/s12909-025-06802-x)
Supplement: Supplementary file 7 — Supplementary Material 7: Additional file 7 Resident evaluation and checklist [file 12909_2025_6802_MOESM7_ESM.pdf]

IM Resident US Elective: Evaluation Checklist

Day 1

Identifying a Safe Site for IJV Access

[ ] Positioning
- [ ] Patient positioned supine or in Trendelenburg
- [ ] Operator positioned at head of bed
- [ ] Machine positioned in line of vision at arm's length

[ ] Transducer and machine
- [ ] High frequency linear array transducer in use
- [ ] Transducer marker to operator left (or match screen)
- [ ] Transducer held in cross section
- [ ] Depth appropriately set
- [ ] Gain appropriately set

[ ]Anatomy
- [ ]Identifies IJV
- [ ]Identifies carotid artery
- [ ] Identifies the SCM muscle

Performing a DVT Study

[ ]Positioning
- [ ]Patient low extremity externally rotated
- [ ]Operator positioned at side of bed
- [ ]Machine positioned in line of vision at arm's length

[ ]Transducer and machine
- [ ]High frequency linear array transducer in use
- [ ]Transducer marker to operator left (or match screen)
- [ ]Transducer held in cross section
- [ ]Depth appropriately set and adjusted during majority of study
- [ ]Gain appropriately set and adjusted during majority of study

[ ]Anatomy
- [ ]Identifies CFV proximal to SFJ
- [ ]Identifies SFJ
- [ ]Identifies bifurcation of femoral artery
- [ ]Identifies CFV distal to the SFJ
- [ ]Identifies deep femoral vein; or verbalizes expected location
- [ ]Identifies popliteal vein

[ ]Evaluation and technique
- [ ]Compression at each of above points
- [ ]90 degree compression technique
- [ ]Obliteration of vessel lumen with juxtaposition of walls

Day 2

Performing a Chest Ultrasound

[ ]Positioning

- [ ]Patient positioned supine with exposed anterior thorax
- [ ]Operator positioned at side of bed
- [ ]Machine positioned in line of vision at arm's length

[ ]Transducer and machine

- [ ]Low frequency phased array transducer prepared for use
- [ ]Transducer marker cephalad
- [ ]Machine set for abdominal setting
- [ ]Depth appropriately set and adjusted during majority of study
- [ ]Gain appropriately set and adjusted during majority of study

[ ]Anatomy

- [ ]Identifies lung sliding in B-mode
- [ ]Identifies lung sliding in M-Mode
- [ ]Identifies A-lines
- [ ]Identifies Right hemidiaphragm

[ ]Evaluation and technique

- [ ]Decreases gain to emphasize the pleural line

<u>**Day 3**</u>

Performing an Abdominal Ultrasound

[ ]Positioning

- [ ]Patient positioned supine with exposed anterior thorax
- [ ]Operator positioned at side of bed
- [ ]Machine positioned in line of vision at arm’s length

[ ]Transducer and machine

- [ ]Low frequency phased array transducer prepared for use
- [ ]Transducer marker cephalad
- [ ]Machine set for abdominal setting
- [ ]Depth appropriately set and adjusted during majority of study
- [ ]Gain appropriately set and adjusted during majority of study

[ ]Anatomy

- [ ]Identifies kidney in long axis
- [ ]Identifies kidney in short axis
- [ ]Identifies inferior pole of kidney in short axis
- [ ]Identified bladder
- [ ]Identifies hepato- renal recess
- [ ]Identifies spleno- renal recess
- [ ]Appropriately positions for right paracolic gutter
- [ ]Appropriately positions for left paracolic gutter
- [ ]Attempts to perform subcostal cardiac view

<u>Day 4 and 5</u>

Performing a Bedside Echocardiography

[ ]Positioning
- [ ]Patient positioned supine with exposed anterior thorax
- [ ]Operator positioned at side of bed
- [ ]Machine positioned in line of vision at arm’s length

[ ]Transducer and machine
- [ ]Low frequency phased array transducer prepared for use
- [ ]Machine set for cardiac setting and dot at operator right
- [ ]Depth appropriately set and adjusted during majority of study
- [ ]Gain appropriately set and adjusted during majority of study
- [ ]Displays working knowledge of caliper functionality

[ ]Anatomy
- [ ]Accomplishes adequate PSLA view
- [ ]Identifies LV, LA, LVOT, RVOT
- [ ]Identifies Ao valve, mitral valve
- [ ]Identifies Descending Aorta
- [ ]Identifies pericardium
- [ ]Achieves adequate PSSA view at the level of the papillary muscles
- [ ]Identifies regions: septal, anterior, lateral, inferior
- [ ]Identifies RV
- [ ]Achieves adequate A4C view
- [ ]Identifies LV, LA, RV, RA, Mitral valve, Tricuspid valve
- [ ]Achieves adequate Subcostal 4 chamber view
- [ ]Identifies LV, LA, RV, RA, Mitral valve, Tricuspid valve
- [ ]Achieves adequate view of IVC in longitudinal section at mid-abdomen
- [ ]Identifies cavo-atrial junction
- [ ]Identifies Aorta in longitudinal section
- [ ]Measures at 2-4cm from cavo-atrial junction

[ ]Knowledge
- [ ]Interprets IVC measurements appropriately for PPV
- [ ]Interprets IVC measurements appropriately for non-PPV
- [ ]*Prompts:*
  - [ ] “How would you maneuver the transducer to move from PSSA view at level of papillary muscle to level of aortic valve”
  - [ ] “Which cardiac view would you use to evaluate RV function and size”
  - [ ] “What finding on PSSA would suggest RV volume or pressure overload?
  - [ ] “What is one alternative way to view the IVC if anterior- abdomen is unavailable”

Ultrasound Elective Resident Evaluation Form

Resident Name:

Please indicate your level of agreement with regards to resident performance in each category by placing an "X" in the box

**Overall Assessment**	**Cannot Perform**	**Can perform under direct supervision**	**Can perform with indirect supervision**	**Can perform independently**	**Can supervise junior trainees**
Vascular Access					
Vascular Diagnostics					
Chest-Lung					
Chest- Pleura					
Chest-Diaphragm					
Basic Echocardiography					
Abdomen/ Retroperitoneal					

**Overall Assessment**	**Cannot Perform**	**Can perform under direct supervision**	**Can perform with indirect supervision**	**Can perform independently**	**Can supervise junior trainees**
Overall Knowledge base in bedside ultrasound applications					
Image Acquisition Skills					
Image Interpretation Skills					
Ability to apply US knowledge, image acquisition and interpretation within clinical context					

1. Professionalism: patient interaction during ultrasound exams
    a. Unsatisfactory
    b. Improvement needed
    c. Meets expectations
    d. Exceeds expectations
    e. Exceptional

2. Professionalism: timeliness
    a. Unsatisfactory
    b. Improvement needed
    c. Meets expectations
    d. Exceeds expectations
    e. Exceptional

3. Quality of US consult notes
    a. Unsatisfactory
    b. Improvement needed
    c. Meets expectations
    d. Exceeds expectations

    e. Exceptional

4. Quality of US Journal Club Presentation
    a. Unsatisfactory
    b. Improvement needed
    c. Meets expectations
    d. Exceeds expectations
    e. Exceptional
