# Supplementary material for: The design, performance and organizational impact of a point-of-care ultrasound (POCUS) elective for internal medicine residents
Source: BMC Med Educ. 2025 Feb 18;25:261. doi: 10.1186/s12909-025-06802-x (PMC11834687; doi:10.1186/s12909-025-06802-x)
Supplement: Supplementary file 8 — Supplementary Material 8: Additional file 8 Knowledge assessment [file 12909_2025_6802_MOESM8_ESM.docx]

Trainee Name: ______________________

Date: ____________

**For the following test questions, please answer best choice available. If you are unaware of the correct answer, choose "Do Not Know" rather than placing a random guess.**

*All images and videos created by author, Dr. Sahar Ahmad.*

1

Attenuation of an ultrasound wave is lowest in which of the following mediums?

[]Water

[]Bone

[]Steel

[]Air

[]Do Not Know

2

Which of the following ratios best describes the typical ultrasound transducer’s percentage time spent emitting waves to the time spent listening for echoes? (Emitt : Listen)

[]10:90

[]30:70

[]50:50

[]70:30

[]90:10

[]Do Not Know

3

Which type of artifact is seen in this image?


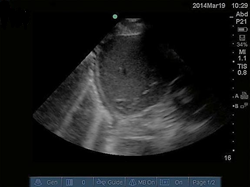


[]Reflection

[]Reverberation

[]Ring down

[]Doppler Shift

[]Nyquist limit

[]Do Not Know

4

The machine settings and transducer from this image are best described as:


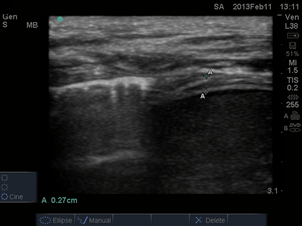


[]Linear Array, High frequency, B-Mode

[]Linear Array, High frequency, M- Mode

[]Phased Array, Low frequency, B- Mode

[]Phased Array, Low frequency, M- Mode

[]Linear Array, Low frequency, B- Mode

[]Phased Array, High frequency, M- mode

[]Do Not Know

5

Prior to starting the procedure, how would you optimize this image?


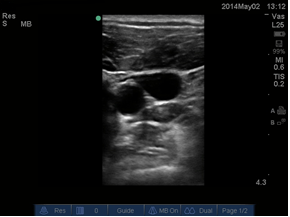


[]Reduce depth

[]Increase depth

[]Reduce gain

[]Increase gain

[]Rotate scan plane 90 degrees

[]Do Not Know

6

Which of the following probe maneuvers are being performed in this video:

<https://youtu.be/FGBQYxB6kCM>

[]Slide

[]Rotate

[]Angulate

[]Tilt

[]Do Not Know

7

Match the image obtained by this transducer position (see image).


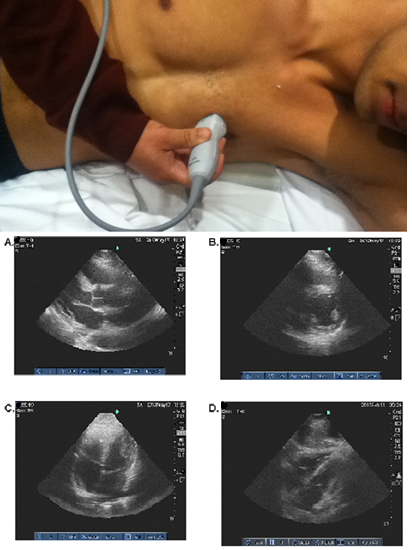


[]A

[]B

[]C

[]D

[]Do Not Know

8

Which structure is shown in this image?


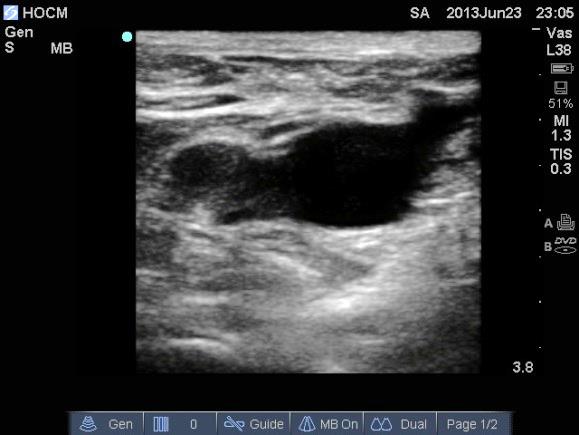


[]Internal Jugular Vein with Carotid Artery in short axis

[]Internal Jugular Vein in longitudinal axis

[]Common Femoral Vein with Saphenous Vein in short axis

[]Common Femoral Vein in longitudinal axis

[]Do Not Know

9

Identify the structure marked by the arrow:


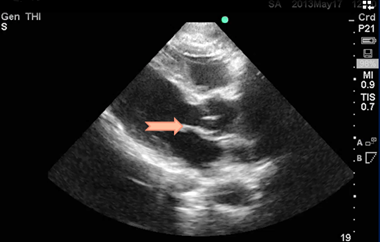


[]Anterior Leaflet of Mitral Valve

[]Posterior Leaflet of Mitral Valve

[]Right Coronary Cusp of Aortic Valve

[]Left Coronary Cusp of Aortic Valve

[]Non Coronary Cusp of Aortic Valve

[]Do Not Know

10

Identify the structure marked by the star:


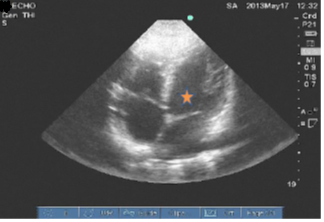


[]Left Ventricle

[]Right Ventricle

[]Left Atrium

[]Right Atrium

[]Do Not Know

11

Identify the structure marked by the arrow:


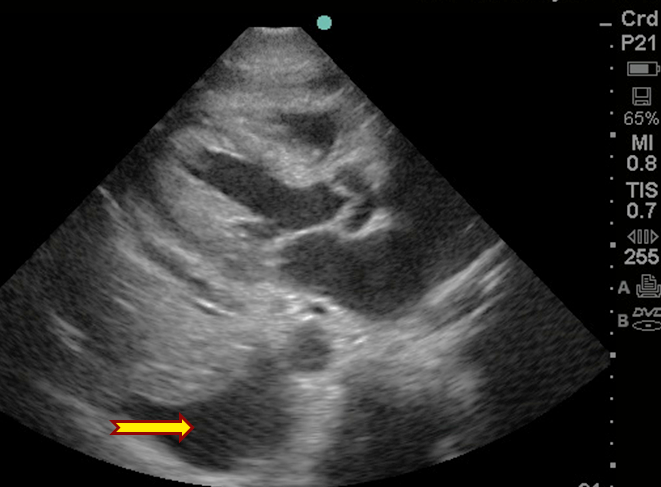


[]Pleural effusion

[]Pericardial effusion

[]Left Ventricle

[]Left Atrium

[]Descending thoracic Aorta

[]Do not know

12

Which of the following is diagnosed by this ultrasound video:

<https://youtu.be/OFWhqynAWJ8>

[]Complex pleural effusion

[]Diaphragm paradox

[]Aortic Aneurysm

[]Aortic Dissection

[]None of the above

[]Do Not Know

13

Identify this image:


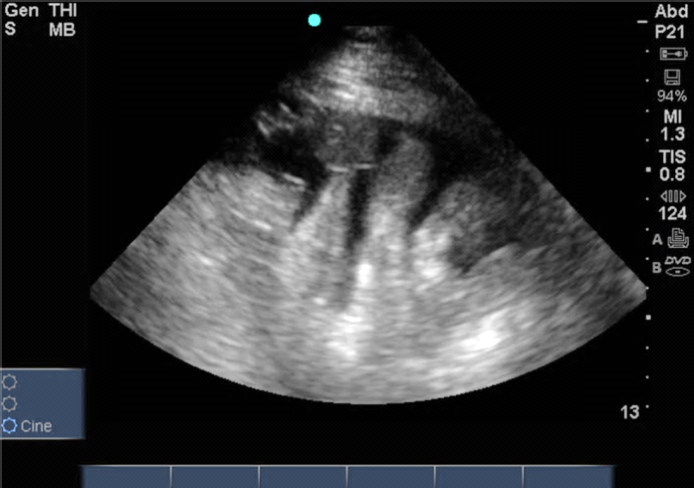


[]Liver

[]Bowel

[]Bladder

[]Kidney

[]Do Not Know

14

Identify this finding:


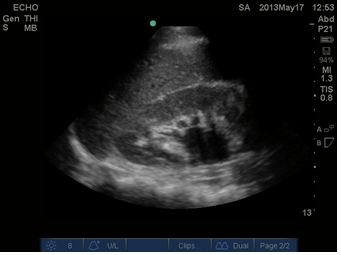


[]Liver cyst

[]Liver with ascites

[]Bowel with ascites

[]Kidney with hydronephrosis

[]Renal cyst

[]None of the above

[]Do Not Know

15

Identify the finding shown in this image:


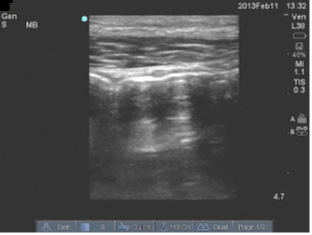


[]A-lines

[]B-lines

[]Lung Sliding

[]Reverberation artifact

[]None of the above

[]Do Not Know

16

This image is compatible with the following clinical diagnoses EXCEPT:
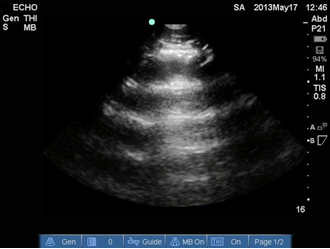


[]Acute pulmonary edema

[]Pulmonary Embolus

[]Pneumothorax

[]Right Main Stem intubation

[]None of the above

[]Do Not Know

17

This patient in shock, is intubated receiving 8 cc/kg Tidal Volume, is paralyzed, and in Normal Sinus Rhythm. What is the best management strategy according to this image?


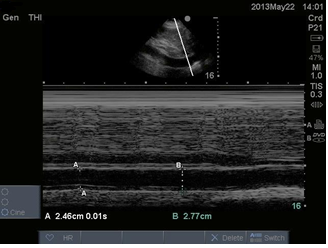


[]Start fluid resuscitation

[]Start vasopressor therapy

[]Diurese

[]All of the above

Exam Answers

1. Choice 1: Water
2. Choice 1: 10:90
3. Choice 3: Ring Down
4. Choice 1: Linear Array, High frequency, B-Mode
5. Choice 1: Reduce depth
6. Choice 3: Angulate
7. Choice 1: A
8. Choice 3: Common Femoral Vein with Saphenous Vein in short axis
9. Choice 1: Anterior Leaflet of Mitral Valve
10. Choice 1: Left Ventricle
11. Choice 1: Pleural Effusion
12. Choice 1: Complex Pleural Effusion
13. Choice 2: Bowel
14. Choice 4: Kidney with hydronephrosis
15. Choice 4: Reverberation artifact
16. Choice 1: Acute pulmonary edema
17. Choice 2: Start vasopressor therapy
