# Supplementary material for: The design, performance and organizational impact of a point-of-care ultrasound (POCUS) elective for internal medicine residents
Source: BMC Med Educ. 2025 Feb 18;25:261. doi: 10.1186/s12909-025-06802-x (PMC11834687; doi:10.1186/s12909-025-06802-x)
Supplement: Supplementary file 9 — Supplementary Material 9: Additional file 9 JC Literature list [file 12909_2025_6802_MOESM9_ESM.pdf]

Journal Club Literature List

1. Moore CL and Copel JA. Point-of-care ultrasonography. N Engl J Med 2011;364:749-57.

2. Mayo PH, Beaulieu Y, Doelken P, et al. American college of chest physicians/ la societe de reanimation de langue francaise statement on competence in critical care ultrasonography. CHEST 2009;135;1050-60.

3. Kory PD, Pellecchia CM, Shiloh AL, et al. Accuracy of ultrasonography performed by critical care physicians for the diagnosis of dVT. CHEST 2011; 139(3):538–42.

4. DiBello C and Koenig S. Diagnosis of deep venous thrombosis by critical care physicians using compression ultrasonography. The Open Critical Care Medicine Journal 2010; 3:43-7.

5. Chen SC, Yen ZS, Wang HP et al. Ultrasonography is superior to plain radiography in the diagnosis of penumoperitoneum. Brit J Surg 2002;89: 351-4.

6. Hoffmann B, Nurnberg D and Westergaard C. Focus on abnormal air: diagnostic ultrasonography for the acute abdomen. Eur J Emerg Med 2012; 00(00):1-8.

7. Lichtenstein DA, Meziere GA, Lagouyete J-F, et al. A-Lines and B-Lines. Lung ultrasound as a bedside tool for predicting pulmonary artery occlusion pressure in the critically ill. CHEST 2009; 136(4):1014-20.

8. Noble VE, Murray AF, Capp R, et al. Ultrasound assessment for extravascular lung water in patients undergoing hemodialysis. CHEST 2009; 135:1433–9.

9. Alrajhi K, Woo MY and Vaillancourt C. Test characteristics of ultrasonography for the detection of pneumothorax. A systematic review and meta-analysis. CHEST 2012; 141(3):703–8.

10. Zanforlin A, Gavelli G, Oboldi D, et al. Ultrasound-guided thoracenthesis: the V-point as a site for optimal drainage positioning. Eur Rev Med Pharmaco 2013. 17:25-8.

11. McCool FD and Tzelepis GE. Dysfunction of the diaphragm. N Engl J Med 2012. 366;(10):932-42.

12. Barbier C, Loubieres Y, Schmit C, et al. Respiratory changes in inferior vena cava diameter are helpful in predicting fluid responsiveness in ventilated septic patients. Intens Care Med 2004; 30:1740–6.

13. Yanagawa Y, Sakamoto T and Okada Y. Hypovolemic shock evaluated by sonographic measurement of the inferior vena cava during resuscitation in trauma patients. J Trauma 2007; 63(6):1245-8.

14. Muller L, Bobbia X, Toumi M, et al. Respiratory variations of inferior vena cava diameter to predict fluid responsiveness in spontaneously breathing patients with acute circulatory failure: need for a cautious use. Crit Care 2012; 16:R188.

15. Fremont B, Pacouret G, Jacobi D, et al. Prognostic value of echocardiographic right/left ventricular end-diastolic diameter ratio in patients with acute pulmonary embolism. CHEST 2008; 133:358–362.

Study Guide

Study Objective/Purpose
1. What are the research questions and purpose?
2. What is the hypothesis?

Methodology/Study Design
1. What is the study design? For example: a clinical trial, cohort, case-control, cross-sectional, or case-series?
2. What is the study population and sample size?
3. How were the subjects recruited?
4. What were the inclusion & exclusion criteria?
5. Was there a control group?

Outcome Measures
1. What are the study endpoints?

Statistical Analysis
1. What were the statistical methods and were they appropriate to answer the research questions?
2. What was the power calculations for sample size? Were these met?

Results & Discussion
1. What were the main results of the study?
2. Do the results speak to the research questions?
3. Are the conclusions justified from the data?

Limitations & Future Study
1. What are the main limitations of the study?
2. What suggestions are made for future work?

Applicability of Results
1. Is this study broadly applicable?
2. Would the learner consider changing practice based on this study?
