# Supplementary material for: The design, performance and organizational impact of a point-of-care ultrasound (POCUS) elective for internal medicine residents
Source: BMC Med Educ. 2025 Feb 18;25:261. doi: 10.1186/s12909-025-06802-x (PMC11834687; doi:10.1186/s12909-025-06802-x)
Supplement: Supplementary file 10 — Supplementary Material 10: Additional file 10 Evaluation survey [file 12909_2025_6802_MOESM10_ESM.docx]

IM US Elective Evaluation Survey

1. Your current training program
   1. Internal Medicine- Preliminary
   2. Internal Medicine- Categorical
   3. Med/Peds
   4. Med/Neuro
   5. Emergency Medicine
2. Your level of training
   1. PGY 1
   2. PGY 2
   3. PGY 3
   4. PGY 4- Resident
   5. Fellow
3. Organization of rotation
   1. Unsatisfactory (1)
   2. Below Average (2)
   3. Average (3)
   4. Above Average (4)
   5. Outstanding (5)
   6. N/A
4. Quality of resources

|  | Unsatisfactory (1) | Below  Average (2) | Average (3) | Above  Average (4) | Outstanding (5) | N/A |
| --- | --- | --- | --- | --- | --- | --- |
| Simulation  Resources |  |  |  |  |  |  |
| Resources: SBUS  Manual |  |  |  |  |  |  |
| Resources:  Other |  |  |  |  |  |  |
| US  Machines and Equipment |  |  |  |  |  |  |

1. Quality of teaching

|  | Unsatisfactory (1) | Below Average  (2) | Average (3) | Above Average  (4) | Outstanding (5) | N/A |
| --- | --- | --- | --- | --- | --- | --- |
| Materials  Provided |  |  |  |  |  |  |
| Didactic  Sessions |  |  |  |  |  |  |
| Hands on  Teaching |  |  |  |  |  |  |

1. Case diversity
   1. Unsatisfactory (1)
   2. Below Average (2)
   3. Average (3)
   4. Above Average (4)
   5. Outstanding (5)
   6. N/A
2. Faculty availability
   1. Unsatisfactory (1)
   2. Below Average (2)
   3. Average (3)
   4. Above Average (4)
   5. Outstanding (5)
   6. N/A
3. Balance of supervision and autonomy
   1. Unsatisfactory (1)
   2. Below Average (2)
   3. Average (3)
   4. Above Average (4)
   5. Outstanding (5)
   6. N/A
4. Meets curriculum goals and objectives
   1. Unsatisfactory (1)
   2. Below Average (2)
   3. Average (3)
   4. Above Average (4)
   5. Outstanding (5)
   6. N/A
